# Systematic review and meta-analysis of adverse health effects associated with mycotoxin exposure

**DOI:** 10.1016/j.envint.2026.110540

**Published:** 2026-10

**Authors:** Tess Goessens, Yasmine Bader, Febe Vertriest, Kokeb Tesfamariam, Sarah De Saeger, Carl Lachat, Marthe De Boevre

**Affiliations:** a Centre of Excellence in Mycotoxicology and Public Health, Faculty of Pharmaceutical Sciences, Ghent University, Ottergemsesteenweg 460, 9000 Ghent, Belgium; b Department of Internal Medicine and Pediatrics, Faculty of Medicine and Health Sciences, Ghent University, C. Heymanslaan 10, 9000 Gent, Belgium; c Department of Food Technology, Safety and Health, Faculty of Bioscience Engineering, Ghent University, Coupure Links 653, 9000 Ghent, Belgium; d Nutrition Epidemiology Group, International Agency for Research on Cancer, 150 Cours Albert Thomas, 69372 Lyon CEDEX 08, France; e Department of Biotechnology and Food Technology, Faculty of Science, University of Johannesburg, PO Box 17011, Doornfontein 2028, South Africa

**Keywords:** Mycotoxins, Biomarkers, Human health outcomes, Systematic review

## Abstract

**Background:**

Mycotoxins, toxic fungal metabolites present in food, are an increasing global health concern expected to intensify with climate change and shifting dietary patterns.

**Aim:**

This review systematically identified, appraised, and synthesized primary research on human health effects associated with exposure to single and multiple mycotoxins, aiming to clarify current evidence, uncertainties, and research gaps.

**Methods:**

Following the PECO framework, Embase, Cochrane Library, PubMed, Web of Science, and Scopus were searched from inception to May 2025 for studies reporting measured or modelled mycotoxin exposure and human health outcomes. Non-human, mechanistic, review, and grey literature were excluded. Three reviewers independently screened studies, with duplicate full-text assessment. Risk of bias was evaluated using OHAT and certainty of evidence with GRADE. Sensitivity analyses, publication bias assessment, and non-quantitative evidence were synthesized using Cochrane effect-direction plots. Random-effects *meta*-analyses assessed associations between aflatoxin exposure and seven health outcomes.

**Results:**

Of 55,342 records, 268 studies met inclusion criteria, covering 17 mycotoxin groups and 71 health outcomes. Liver disease, particularly hepatocellular carcinoma, and kidney disease were the most frequently investigated outcomes, with most studies conducted in Africa and Asia. Qualitative synthesis demonstrated predominantly positive associations between mycotoxin exposure and neurological, metabolic, oncological, renal, reproductive, and developmental disorders. Certainty of evidence was very low for all 21 assessed outcomes, reflecting the predominance of cross-sectional designs and substantial between-study inconsistency. Quantitative *meta*-analyses of 42 studies identified associations between aflatoxin exposure and hepatocellular carcinoma, liver cirrhosis, elevated ALT, and gallbladder cancer.

**Conclusion:**

Evidence supports associations between mycotoxin exposure and multiple health risks; however, heterogeneity in study design, exposure assessment, and outcome reporting highlights the need for more rigorous and harmonized research across diverse populations.

## 1 Introduction

Mycotoxins are toxic fungal metabolites that contaminate staple crops, nuts, fruits, and processed products worldwide, and can enter meat, eggs, and milk through contaminated animal feed (IARC, 2002). Mycotoxins, produced mainly by Aspergillus, Fusarium, and Penicillium species, often co-occur, making simultaneous exposures common (De Ruyck et al., 2015). Up to 80% of global crops are affected (Eskola et al., 2020), a burden expected to rise with climate change (Battilani et al., 2016, Moretti et al., 2019). Additionally, low- and middle-income countries face disproportionate risks due to reliance on vulnerable staples, limited diet diversity, and weaker food safety regulation (Global Panel on Agriculture and Food Systems for Nutrition Foresight Report 2.0, 2020).

Exposure to mycotoxins has been linked to a wide spectrum of adverse outcomes, including hepatocellular carcinoma (HCC) (Bressac et al., 1991), kidney disease (Khoi et al., 2021), metabolic and reproductive disorders, and immune dysfunctions (Malvandi et al., 2022). The International Agency for Research on Cancer classifies aflatoxins (AFs) as Group 1 carcinogens, while several other mycotoxins such as ochratoxin (OTA), fumonisins (FBs) and sterigmatocystin (STC) are considered possibly carcinogenic (List of Classifications). Despite these well-documented toxic and carcinogenic properties, there are currently no globally harmonized toxicological reference values or human biomonitoring guidelines that define internal exposure thresholds associated with adverse health effects. Most regulatory frameworks, including those of the European Commission, European Food Safety Authority (EFSA) and the Joint FAO/WHO Expert Committee on Food Additives (JECFA), focus primarily on setting maximum allowable concentrations of mycotoxins in food, feed, and agricultural commodities to control external exposure (Official Journal of the European Union 119, 103-157, 2023). However, globally consistent toxicological threshold values and corresponding biomonitoring reference levels for internal exposure in humans remain to be established.

Despite the global relevance of mycotoxin contamination, most existing reviews have examined single toxins or outcomes in isolation, overlooking co-exposure scenarios that better reflect real-world conditions and leaving key areas such as inflammatory bowel disease, certain cancers, and neurological disorders unexamined (De Ruyck et al., 2015). This systematic review (SR) and *meta*-analysis offers a comprehensive synthesis of major mycotoxins and their mixtures in relation to reported human health outcomes. By addressing fragmented evidence and considering sources of uncertainty, it seeks to inform more comprehensive risk assessments of a potentially underestimated global threat.

### 1.1 Objectives

This SR aims to systematically search for, appraise and synthesize primary research evidence to identify what is known about dietary mycotoxin-related health effects and what remains unknown, as well as the uncertainty around findings and the recommendations for the future. The work follows up on the call for SRs by the European Commission in the field of regulatory risk assessment of emerging chemicals (Goessens et al., 2024, European Commission - Horizon Europe Programme, 2021). Specific objectives are:

• To identify and critically appraise all available studies assessing associations between measured or modelled exposure to any of the 69 major mycotoxins listed in Supplementary Table 1, whether single or multiple − and adverse health outcomes in humans across all ages, genders, health statuses, and life stages.
• To characterize areas of uncertainty and major knowledge gaps regarding health human outcomes linked to mycotoxins and their co-exposure patterns.
• Provide recommendations for future research and regulatory risk assessment activities.

## 2 Methods

### 2.1 Method planning

This SR and *meta*-analysis has been conducted following PRISMA reporting guidance (Page et al., 2021) and COSTER recommendations (Whaley et al., 2020). The protocol was registered on PROSPERO (CRD42022366592) and OSF (https://osf.io/9zdpj/), and published in a peer-reviewed journal (Goessens et al., 2024). The following PECO aspects were addressed: Population − humans of all ages; Exposure – mycotoxins, single or multiple; Comparator − lower or no exposure; Outcome − all reported adverse health outcomes potentially related to mycotoxin exposure. These aspects informed the primary research question: In individuals of all ages and genders (Population), what impact does dietary mycotoxin exposure (Exposure) have on human health (Outcome), in comparison to either no exposure or a reduced level of exposure (Comparator)?

### 2.2 Protocol deviations

#### 2.2.1 Meta-analysis

The protocol’s original approach selects fixed- versus random-effects models based primarily on I2 thresholds, with *meta*-regression applied only when I2 exceeds 75%. This strategy proved to be methodologically limited. I2 is not a model-selection criterion and is influenced by study size and variance structure, which can lead to misleading interpretations. In addition, fixed-effects models are conceptually restrictive in global observational settings with heterogeneous populations, exposure definitions, and analytical methods, regardless of the magnitude of I2. As such, the statistical approach was therefore revised as follows:

- Heterogeneity was characterized using τ^2^ and 95% prediction intervals for all primary outcomes, providing a clearer representation of between-study variability and the expected range of effects across contexts.
- A uniform random-effects framework was applied to all outcomes.

Additionally, and in contrast to the protocol, effect sizes were not converted into a common metric, and *meta*-analyses were conducted only when at least three studies examined the same exposure-outcome pair using comparable measures, ensuring clinically consistent and directly comparable estimates. Studies were further stratified by design and biological matrix to reduce heterogeneity.

#### 2.2.2 Sensitivity analysis

The protocol initially specified sensitivity analyses restricted to studies at low RoB. However, to preserve adequate statistical power and avoid excessive loss of information, studies with high or uncertain RoB were retained in the primary analysis. Sensitivity analyses were then conducted by comparing results with and without these studies to assess the robustness of the findings.

#### 2.2.3 Presenting the evidence

Although the protocol specified that outcomes would primarily be assessed qualitatively, with *meta*-analysis performed where applicable, the final manuscript additionally incorporated Cochrane effect direction plots to systematically summarize the direction and consistency of effects across studies (Boon and Thomson, 2021). This was done to provide a clearer and more structured overview of heterogeneous findings that could not be meaningfully presented through narrative synthesis alone. Additionally, given the heterogeneity and uneven distribution of studies, heat maps were considered to add limited interpretive value and risk oversimplification. Instead, research gaps were addressed in detail within the Discussion section.

#### 2.2.4 Discussion

In contrast to the protocol’s plan to integrate mechanistic and animal evidence into the discussion, the final manuscript prioritized human evidence. Given the substantial heterogeneity, wide prediction intervals, and limited number of studies for several outcomes, a more extensive mechanistic narrative could have overstated the consistency of biological evidence. This deviation better aligns conclusions with the observed uncertainty and maintains focus on the strengths and limitations of the epidemiological data.

### 2.3 Search strategy and data sources

Embase, Cochrane Library, PubMed, Web of Science Core Collection, and Scopus were searched from inception to May 5, 2025 via detailed electronic search strings which have been published before (Goessens et al., 2024). All references were imported into Covidence, allowing for automatic deduplication. Primary human studies (cohort, case-control, case & outbreak reports, cross-sectional and clinical trials) reporting on any of 69 mycotoxins (Supplementary Table 1) and adverse health outcomes pertaining to cancer, non-carcinogenic diseases, and reproductive & developmental adverse outcomes, were included. Animal studies, mechanistic studies, reviews, opinions, and grey literature (conference abstracts, theses and book chapters) were excluded. No restrictions were applied for language or publication year. All inclusion and exclusion criteria are presented in Table 1.

**Table 1:** Inclusion and exclusion criteria of the SR according to the PECO framework.

**Inclusion criteria**	**Exclusion criteria**
***Population***	
Human − No restrictions on age, gender, health status or life stage at exposure or outcome assessment. Only whole organisms are considered.	Animals and human organs, tissues, cell lines or cellular components.
***Exposure***	
Dietary exposure of mycotoxins, either measured (*e.g.* environmental and consumption data, or biomonitoring studies) or modelled (*e.g.* physiologically based pharmacokinetic modelling).	Non-dietary routes (*e.g.* inhalation, dermal).
***Comparator***	
Human − in cohort studies and cross-sectional studies, health outcomes will be compared amongst groups having different levels of dietary exposure, assessed through environmental and consumption data, biomonitoring studies or physiologically based pharmacokinetic modelling. In randomized controlled trials and case-control studies, control groups will be defined as not receiving the tested oral mycotoxin(s).	Animals and human organs, tissues, cell lines or cellular components.
***Outcome***	
All adverse health outcomes (primary outcomes divided into 3 categories *i.e.* cancer, non-carcinogenic diseases and reproductive & developmental adverse outcomes, as well as secondary outcomes) measured in the exposed humans.	Effects on chromatin (*i.e.* DNA mutations, gene expression, RNA & histone modifications).
***Study design***	
Cohort studies, (non–)randomized controlled trials, (non–)case-control studies, cross-sectional studies and case reports.	*In vitro*, *ex vivo*, *in silico* and review studies, as well as expert’s opinions.

### 2.4 Study selection and data extraction

Titles and abstracts were screened independently by three reviewers; full texts were reviewed in duplicate (TG, YB, and FV). Data extraction from 268 eligible studies was performed independently by three reviewers (TG, YB, and FV), and discrepancies were resolved through discussion using a two-thirds majority vote. Extracted information included study design, population characteristics, mycotoxin type and concentration, exposure assessment, and reported effect measures (odds ratio (OR), relative risk (RR), mean difference (MD), standardized mean difference (SMD), correlation coefficients (CC) and β-values). The data extraction template related to this SR can be consulted via https://osf.io/n5etv/.

### 2.5 Quality of evidence

#### 2.5.1 Risk of bias for individual studies

The RoB for each individual study was assessed independently by two reviewers (TG and YB) using the Office of Health Assessment and Translation (OHAT) RoB tool (Ntp, 2019). Disagreements were resolved by consultation with a third reviewer (FV). Evaluation comprised six domains: appropriateness of comparison groups, control of confounding, completeness of outcome data, confidence in exposure characterisation, confidence in outcome measurement, and completeness of reporting. Each domain was rated as definitely low, probably low, probably high or definitely high RoB. Three domains were specified in greater detail than in the standard instrument, to make the criteria explicit and reproducible for this exposure and this set of outcomes. For confounding, up to three outcome-specific modifying variables were pre-specified per outcome on the basis of World Cancer Research Fund criteria (Interactive Cancer Risk Matrix | World Cancer Research Fund) and the wider literature (*e.g.,* diet, viral infection, alcohol consumption (Griswold et al., 2018); adjustment for at least one such reliable confounder was considered sufficient for the criterion to be fulfilled.

For exposure characterisation, a rating of definitely low RoB required both quantification of mycotoxins in biological samples and a study design appropriate to the temporality of the outcome: cohort or case–control designs for chronic disease, cross-sectional designs for acute disease − with biological measurement by liquid chromatography tandem mass spectrometry (LC-MS/MS) regarded as the analytical gold standard; studies quantifying biomarkers under a design inappropriate to the outcome were rated probably low. For outcome measurement, criteria distinguished diagnosis in a hospital setting against objective clinical gold standards (imaging or specific biomarkers) from ascertainment by trained personnel outside a hospital setting, and both from self-report or questionnaire-based diagnosis. Studies therefore received lower RoB ratings when comparison groups were matched for age, sex and source population; when at least one reliable confounder was accounted for by matching or adjustment; when outcome data were complete or non-measurement was explicitly verified; and when exposure and diagnosis were established through robust analytical methods and objective clinical criteria respectively. Higher ratings resulted from absent or poorly matched control groups, no adjustment for any reliable confounder, incomplete outcome capture − common in outbreak reports − questionnaire-based diagnosis, or missing methodological detail. The overall rating was derived from the profile of domain judgements, with domains 2, 4 and 5 (confounding, exposure characterisation and outcome measurement) treated as key domains. Studies were rated definitely low RoB when all key domains were low and most other applicable domains were also low; probably low when most domains, including at least two key domains, were low; and probably or definitely high as concerns in the key domains accumulated. All judgements and their underlying rationale were documented systematically in tabular format (Supplementary Table 28).

#### 2.5.2 Certainty of body of evidence

The certainty of evidence for each health outcome was evaluated independently by the same reviewers (TBG & YB) using the Grading of Recommendations Assessment, Development and Evaluation (GRADE) approach, as adapted for environmental and nutritional exposures by Morgan et al. (2016). Disagreements were resolved by consultation with a third reviewer (FV), and all co-authors reviewed the related decisions. Initial certainty was assigned per outcome on the basis of four study design features of the underlying body of evidence: whether exposure was controlled, whether exposure occurred prior to the outcome, whether outcome data were reported at the individual level, and whether a comparison group was used. Bodies of evidence meeting all four features started at high certainty, three at moderate, two at low, and one or none at very low. As all included studies were observational, controlled exposure was never met. Certainty was subsequently downgraded for risk of bias, inconsistency, indirectness, imprecision and publication bias. Each domain was appraised individually and rated as not serious, serious or very serious, corresponding to a downgrade of no level, one level or two levels respectively. Because study design and the presence of a comparison group are captured in the initial certainty rating, they were not reassessed under indirectness, which was evaluated for populations, exposure and outcome only, so as to avoid double counting (Guyatt et al., 2011). Upgrading factors − a large magnitude of effect, a clear dose–response gradient, and residual confounding likely to attenuate rather than create the observed association − were considered only for bodies of evidence in which no downgrading domain raised serious concerns, in line with GRADE guidance. Domain ratings were not converted into numerical scores and were not summed. Consistent with GRADE being a structured qualitative judgement rather than an additive scoring algorithm, the final certainty rating for each outcome was reached by consensus and expressed on the four-level scale (high, moderate, low, very low). All judgements and their underlying rationale were documented systematically in tabular format (Supplementary Tables 29–49) (Morgan et al., 2016). In order to make the evidential basis of each certainty rating explicit, it was additionally recorded for each outcome assessed with GRADE whether a study design appropriate to the time course of the outcome was available and whether a supporting biological mechanism has been described; this mapping was compiled after the certainty assessment had been completed and did not influence the ratings.

### 2.6 Data synthesis

#### 2.6.1 Meta-analysis

Effect estimates were pooled when ≥3 studies examined the same toxin-outcome pair, effect measure, and clinical outcome definition. The OR, RR and CC measures were analysed separately in the same primary *meta*-analysis. Primary analyses were stratified by study design (cohort, case–control, cross-sectional), and pooling was limited to studies assessing exposure in the same biological matrix (plasma/serum, urine, or food). For AF exposure detected in plasma or serum, pooled ORs were estimated separately for each specific outcome, including gallbladder cancer, stunting, pregnancy anaemia, wasting, underweight, liver cirrhosis, and HCC. For HCC in relation to AF exposure detected in urine, pooling was conducted using RR, which were analysed independently from OR-based estimates.

For liver function markers (AST, ALT, and total bilirubin), most studies reported CC, which were pooled after transformation to Fisher’s *z*. For each study, standard errors were derived from the reported CC and *p*-value, followed by Fisher’s *z*-transformation to obtain approximately normal effect size distributions suitable for *meta*-analysis (Michael Borenstein, 2009). Sensitivity analyses were performed at multiple levels using leave-one-out methods on non-transformed datasets (Baujat et al., 2002).

Between-study heterogeneity was quantified using the τ^2^ (between-study variance) and I^2^ statistic (total variance), and 95% prediction intervals were calculated to estimate the expected range of effects in comparable future studies (Choi and Kang, 2025). To account for methodological heterogeneity arising from geographic, analytical, and design differences, random-effects models were applied using the restricted maximum likelihood (REML) estimator with Hartung–Knapp–Sidik–Jonkman adjustment (Inthout et al., 2014, Viechtbauer, 2005).

Publication bias was assessed with funnel plots and Egger’s test for analyses including ≥10 studies (Nguyen et al., 2022). A trim-and-fill procedure was applied to these plots, and the adjusted pooled effect was compared with the original estimate (Duval and Tweedie, 2000). This was only performed for HCC as outcome in association with AF exposure detected in plasma or serum.

All analyses were conducted in R (version 4.5.0) using the *meta*, *metainf*, *metafor*, pROC and *dmetar* packages.

#### 2.6.2 Effect direction plots

For toxin-outcome pairs ineligible for *meta*-analysis, a qualitative syntheses was conducted using Cochrane effect direction plots (Boon and Thomson, 2021). Upward arrows denote positive health effects, downward arrows indicate adverse health impacts, and horizontal arrows represent no effect or conflicting findings; arrow size was scaled proportionally to total sample size (<50, 50–300, or >300 participants). Study quality was color-coded by RoB: green (low RoB/high quality), yellow (some concerns/moderate quality), and red (high RoB/low quality) (Boon and Thomson, 2021).

## 3 Results

All results, including the complete data extraction as well as quality assessment, can be consulted open-access via https://osf.io/n5etv/.

### 3.1 Study selection

After removing 34,527 duplicates, 55,342 references were screened by title and abstract, yielding 529 articles for full-text review. Of these, 529 met the inclusion criteria (Fig. 1). Full texts of 36 studies could not be retrieved. To enhance transparency, reproducibility, and validity − and to ensure consistency with PRISMA standards − these studies were excluded from the qualitative analysis (Supplementary Table 2). A total of 225 additional studies were excluded based on incorrect study design (*i.e.* review articles or studies reporting only regional food contamination data without exposure assessment, n = 131), incorrect exposure (*i.e.* non-dietary or non-mycotoxin exposure, n = 19), incorrect outcome (*i.e.* no relevant health outcome assessed or outcomes limited to chromatin effects, n = 41), incorrect population (*i.e.* animal studies, n = 2), being conference abstracts (n = 10), a purely pharmacokinetic study (n = 1), or duplicates not removed during automatic deduplication (n = 21).

**Fig. 1:**
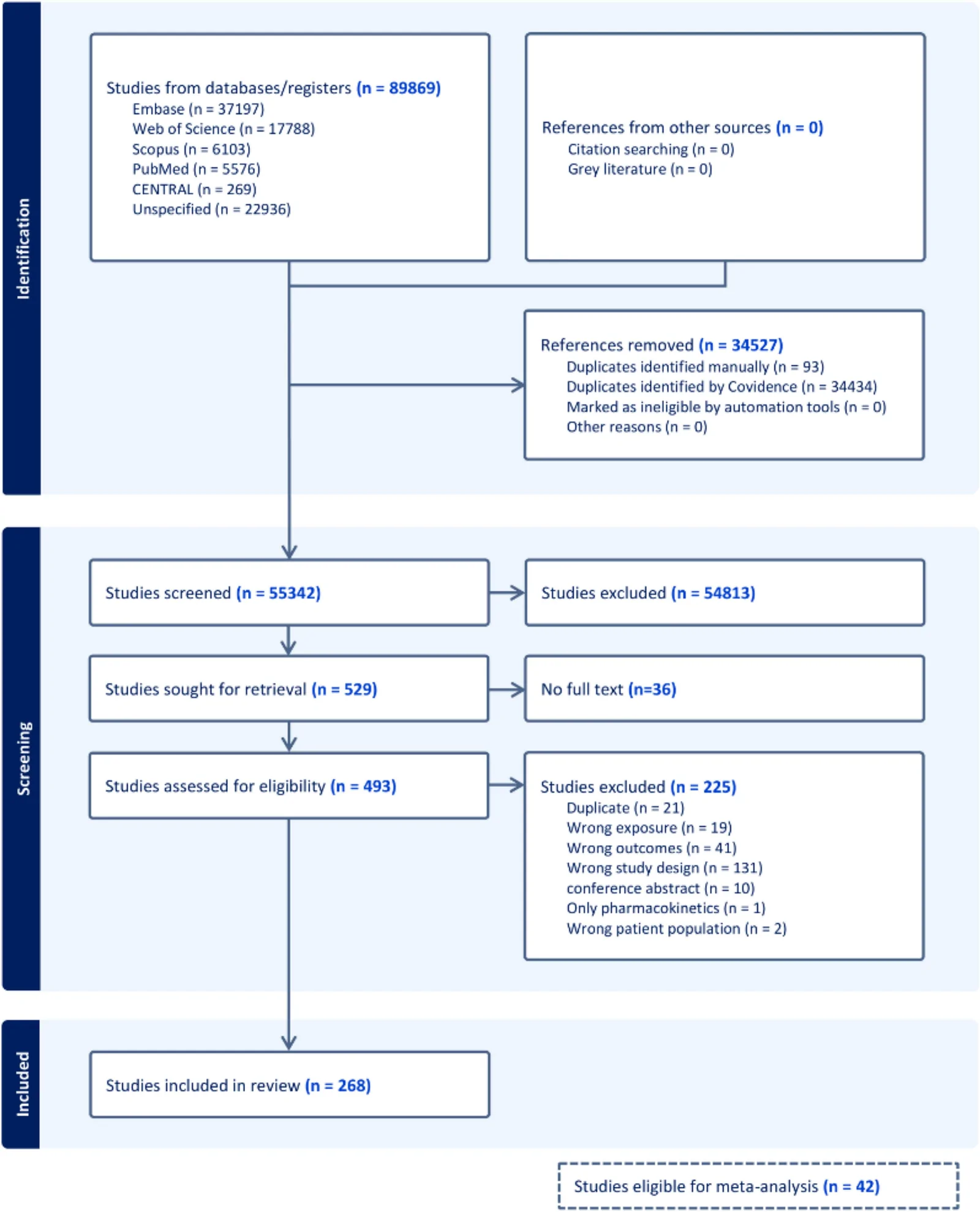
PRISMA flow diagram of study selection.

### 3.2 Study characteristics

Study-level characteristics of all included studies are presented in Supplementary Tables 3–27. Included studies spanned from 1967 to 2025, with sample sizes ranging from 1 to 450,112 participants, and largest sample sizes reported for studies conducted in China, South Africa, Egypt, Tanzania, Kenya and USA (Fig. 2). Geographically, most studies were conducted in China (n = 40), Egypt (n = 16), the United States (n = 16), Nigeria (n = 14), and South Africa (n = 12); 7 studies did not report population location. Participant ages ranged from newborns to 92 years; 39 studies included only females, 17 only males, 170 studies reported both, and 42 did not report sex.

**Fig. 2:**
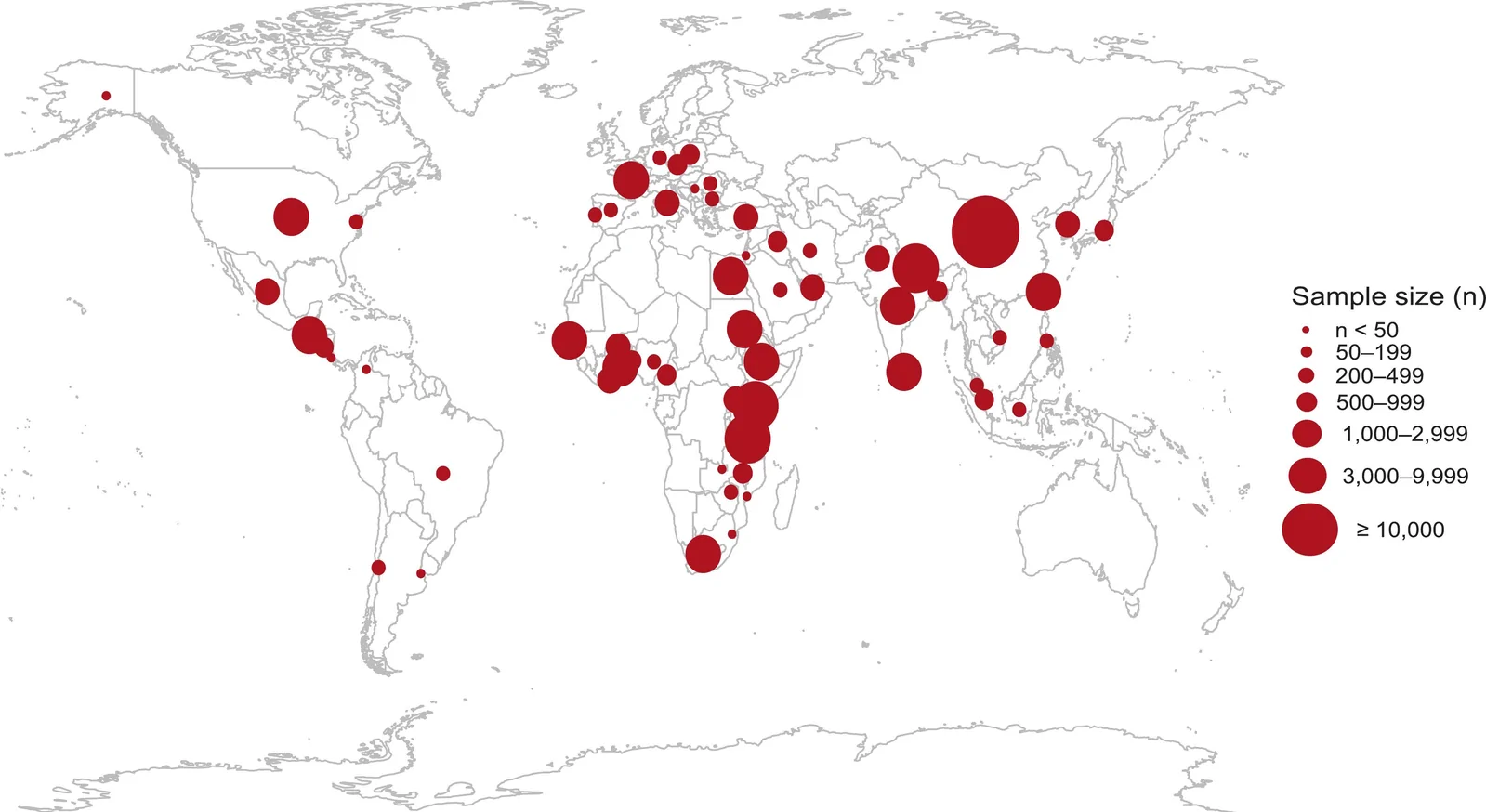
**Geographic distribution of included studies and sample sizes.** Red circles indicate the location of each study, with circle size proportional to the number of participants (n) from that country. Countries with larger sample sizes (*e.g.*, China, South Africa, Egypt, Tanzania, Kenya, USA) are represented by larger circles, whereas countries with smaller contributions (*e.g.*, Brazil, Chile, Czechia) are shown with smaller circles. Some studies could not be mapped due to missing location information. (For interpretation of the references to colour in this figure legend, the reader is referred to the web version of this article.)

### 3.3 Mycotoxin exposures

AFs were the most frequently studied toxin (n = 220, 82%), followed by OTA (n = 53, 20%), zearalenone (ZEN and derivatives, n = 31, 12%), multi-mycotoxin exposures (≥3 classes; n = 29, 11%), deoxynivalenol (DON and derivatives, n = 24, 9%) and FBs (n = 22, 8%) (see Supplementary Tables 3–27 for study-level details). Detection methods included enzyme-linked immunosorbent assay (ELISA (n = 73, 27%)), high-performance liquid chromatography with fluorescence detection (HPLC-FLD (n = 70, 26%)), liquid-chromatography tandem-mass spectrometry (LC-MS/MS (n = 56, 21%)), DNA sequencing (n = 17, 6%), thin-layer chromatography (TLC (n = 15, 6%)), polymerase chain reaction (PCR (n = 4, 2%)), dietary questionnaires (n = 3, 1%), and others (n = 30, 12%).

The reported exposure levels varied widely across studies, likely reflecting differences in matrices analysed, analytical methods used, and geographical contamination levels.

### 3.4 Quality of evidence

#### 3.4.1 Risk of bias for individual studies

A detailed overview of the RoB assessment (traffic light and summary plots) is shown in Supplementary Tables 1–17, with the rationale for individual judgements provided in Supplementary Table 28. Of the 268 assessed studies, 198 (74%) were rated as having low RoB − 65 definitely low and 133 probably low − indicating that they were generally well designed, well conducted, and likely to yield reliable findings. The remaining 70 studies (26%) were judged to have high RoB, comprising 53 probably high and 17 definitely high. High ratings were driven predominantly by the comparison-group and confounding domains: 192 studies (72%) were rated probably or definitely high for the appropriateness of comparison groups, most often because no control group was included, and 169 studies (63%) for confounding, reflecting an absence of matching or of adjustment for any reliable confounder. Incomplete outcome data contributed to a lesser extent (117 studies, 44%), as did selective reporting (74 studies, 28%) and outcome measurement (57 studies, 21%). Exposure characterisation was rated low in 255 studies (95%), since mycotoxins were nearly always quantified in biological matrices; under the stricter criterion applied here, however, only 78 studies (29%) reached a definitely low rating, the remainder falling to probably low because the study design was not appropriate to the temporality of the outcome. In accordance with OHAT handbook guidelines^15^, and recognising that excluding high RoB studies could result in incomplete evidence and reduced transparency, such studies were retained in the final synthesis, with their limitations carefully considered and their contribution to the overall conclusions interpreted cautiously (Handbook for Conducting Systematic Reviews for Health Effects Evaluations).

#### 3.4.2 Certainty of body of evidence

GRADE assessment indicated very low certainty of evidence for all adverse health outcomes evaluated (Table 2), with detailed judgements and rationales provided in Supplementary Tables 29–49. Initial certainty was assigned per outcome on the basis of study design features following the adapted GRADE approach of Morgan et al. (2016), rather than uniformly set to low for observational evidence. Only gallbladder cancer began at moderate certainty, reflecting appropriate study designs with matched control groups; eight outcomes began at low and the remaining twelve at very low certainty, predominantly because exposure was not established as preceding the outcome and because comparison groups were frequently absent. In every case, subsequent downgrading brought certainty to the lowest category. GRADE assessment was not feasible for pancreatic cancer, female infertility, musculoskeletal disease and mucous fibrosis, as only a single study was available for each.

**Table 2:** Overview of the GRADE assessment of the overall body of evidence of this SR, by health outcome.

Outcome	Initial certainty^a^	Risk of bias	Inconsistency	Indirectness	Imprecision	Publication bias	Overall certainty
Acute food intoxication	Low	Serious	Not serious	Not serious	Serious	Serious	**⊕○○○ Very low**
Liver disease	Very low	Not serious	Very serious	Very serious	Serious	Serious	**⊕○○○ Very low**
Gallbladder cancer	Moderate	Not serious	Very serious	Not serious	Serious	Not serious	**⊕○○○ Very low**
Metabolic disease	Very low	Not serious	Serious	Very serious	Not serious	Serious	**⊕○○○ Very low**
Neurological disease	Low	Not serious	Not serious	Very serious	Serious	Serious	**⊕○○○ Very low**
Male reproductive disease	Very low	Not serious	Serious	Very serious	Serious	Not serious	**⊕○○○ Very low**
Female reproductive disease	Very low	Not serious	Serious	Not serious	Serious	Not serious	**⊕○○○ Very low**
Premature puberty	Low	Not serious	Not serious	Not serious	Serious	Not serious	**⊕○○○ Very low**
Kidney disease	Low	Serious	Serious	Very serious	Not serious	Not serious	**⊕○○○ Very low**
Bladder cancer	Low	Serious	Serious	Serious	Not serious	Not serious	**⊕○○○ Very low**
Lung disease	Very low	Not serious	Serious	Very serious	Serious	Serious	**⊕○○○ Very low**
Immunomodulation	Low	Not serious	Serious	Not serious	Not serious	Not serious	**⊕○○○ Very low**
Kwashiorkor	Low	Not serious	Serious	Not serious	Serious	Serious	**⊕○○○ Very low**
Wasting	Very low	Not serious	Serious	Very serious	Not serious	Not serious	**⊕○○○ Very low**
Underweight	Very low	Not serious	Serious	Very serious	Not serious	Not serious	**⊕○○○ Very low**
Stunting	Very low	Not serious	Serious	Very serious	Not serious	Not serious	**⊕○○○ Very low**
Blood disease	Very low	Not serious	Serious	Very serious	Serious	Not serious	**⊕○○○ Very low**
Oesophageal disease	Low	Not serious	Not serious	Very serious	Not serious	Not serious	**⊕○○○ Very low**
Gastric disease	Very low	Not serious	Serious	Serious	Serious	Not serious	**⊕○○○ Very low**
Intestinal disease	Low	Not serious	Serious	Very serious	Serious	Not serious	**⊕○○○ Very low**
Adverse birth outcomes	Very low	Not serious	Serious	Very serious	Not serious	Not serious	**⊕○○○ Very low**

Note:^a^ Initial certainty was assigned on the basis of study design features rather than a uniform starting point for all observational evidence, following the adaptation of GRADE for observational exposure data by Morgan et al. (2016). Bodies of evidence meeting all four features (investigator-controlled exposure; exposure demonstrably preceding the outcome; outcome data at the individual level; presence of a comparison group) begin at high certainty, with one level deducted for each feature not met. Because exposure was not investigator-controlled in any included study, the maximum attainable initial certainty in this review was moderate.* = GRADE could not be performed for pancreatic cancer, female infertility, musculoskeletal disease and mucous fibrosis because only one study was included.

Downgrading was driven principally by inconsistency and indirectness. Effect estimates varied widely in magnitude across studies, with substantial to considerable statistical heterogeneity where *meta*-analysis was possible (I^2^ = 45.5–82.5%). Indirectness reflected differences in study populations (low-, middle- and high-income countries; children versus adults), in exposure assessment (different mycotoxins, biological matrices and analytical sensitivities), and in outcome ascertainment (hospital-based versus community-based settings). Imprecision was a serious concern for roughly half of the outcomes, chiefly where most studies had fewer than 100 participants and no sample size calculation was reported. Application of this domain was constrained by reporting practice: for six outcomes − male and female reproductive disease, kidney disease, bladder cancer, lung disease and kwashiorkor − no included study reported a confidence interval at all, and for neurological disease only a single study did so. Publication bias was judged serious for a minority of outcomes, most notably acute food intoxication, liver disease, metabolic disease, lung disease and kwashiorkor, reflecting underreporting of non-significant findings and, in outbreak settings in low-income countries, case ascertainment restricted to individuals able to access hospital care.

Upgrading factors were not applied to any outcome. In line with GRADE guidance, upgrading was considered only for bodies of evidence in which no downgrading domain raised serious concerns, and no outcome met this condition. Observations relevant to these factors are nonetheless retained in Supplementary Tables 29–49: a large magnitude of effect was apparent for several outcomes, including liver disease, gallbladder cancer, premature puberty and blood disease; a consistent dose–response gradient was rarely demonstrable, as concentrations varied widely both within and between biological matrices; and control of residual confounding was insufficient in most studies to establish the direction of any residual bias.

Taken together, these ratings indicate that the associations identified in this review should be regarded as hypothesis-generating rather than as establishing causal relationships, and they identify prospective study design with adequate comparison groups as the principal methodological gap to be addressed in future work.

Twenty of the 21 outcomes were supported by an appropriate study design, a described biological mechanism, or both (Supplementary Table 51) − premature puberty, for instance, rests on biological plausibility alone through the estrogenic activity of ZEN, whereas liver disease is supported both by cohort data on hepatocellular carcinoma and by the established mechanism of AFB1-induced TP53 mutation − while for metabolic disease neither criterion was clearly met, indicating that the uniformly very low certainty of evidence reflects genuine limitations in the underlying literature rather than the restricted applicability of the framework.

### 3.5 Meta-analysis of aflatoxin exposure

A *meta*-analysis was performed for AF exposure measured in serum or plasma (n = 38) in relation to seven outcomes **(**Fig. 3**)**: gallbladder cancer (4 studies), stunting (7 studies), pregnancy anaemia (3 studies), liver cirrhosis (6 studies), liver parameters (5 studies), underweight (4 studies) and HCC (18 studies). The pooled ORs [95% CI] based on random-effects models, stratified by study design, were as follows: gallbladder cancer, 3.82 [1.08–13.53]; stunting, 1.10 [0.98–1.24]; pregnancy anaemia, 1.83 [0.36–9.17]; liver cirrhosis, 3.98 [1.17–13.55]; underweight, 1.33 [0.33–5.35]; HCC and 2.31 [1.69–3.15]. For liver function biomarkers, pooled CCs [95% CI] indicated a moderate positive association for alanine aminotransferase (ALT: 0.55 [0.33–0.72]), while weaker and non-significant associations were observed for aspartate aminotransferase (AST: 0.11 [−0.68–0.78]) and total bilirubin (0.21 [−0.51–0.76]), based on random-effects models stratified by study design. A separate *meta*-analysis of AF exposure measured in urine (n = 4 studies) in relation to HCC yielded a pooled RR [95% CI] of 2.66 [1.80–3.93].

**Fig. 3:**
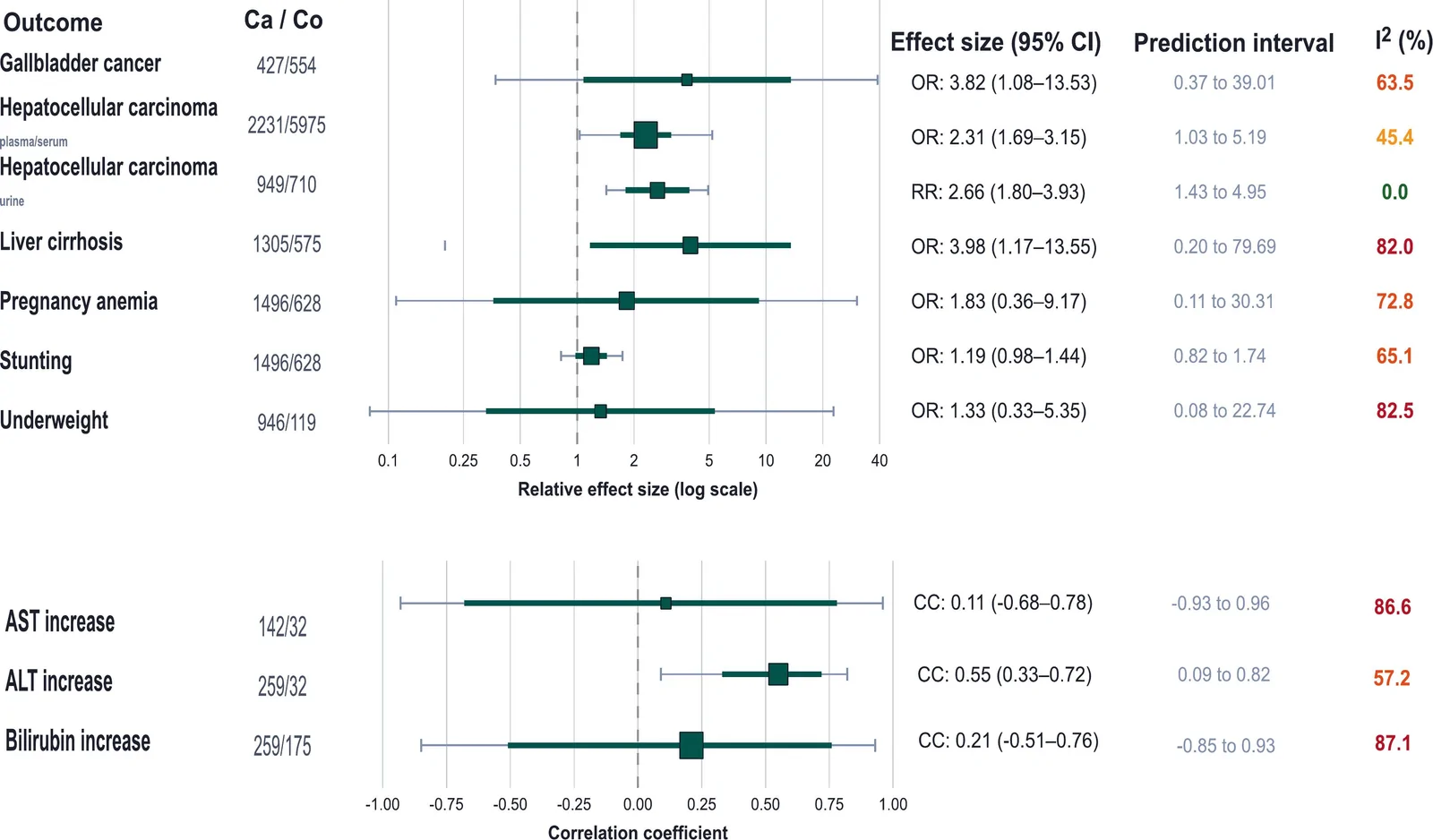
**Forest plot summarizing pooled effect estimates for gallbladder cancer, hepatocellular carcinoma, liver cirrhosis, pregnancy anaemia, stunting, underweight, and increases in serum or plasma liver biomarkers (AST, ALT, and bilirubin).** Pooled estimates are restricted to clinically comparable endpoints within each outcome (*e.g.* incident cancer cases and standardized definitions for anthropometric outcomes). CI: confidence interval; PI: prediction interval; AST: aspartate aminotransferase; ALT: alanine aminotransferase. Box size is proportional to the number of cases and controls included in each analysis. Colours of I^2^ indicate the degree of heterogeneity: low (green), moderate (yellow), substantial (orange), and high (red). (For interpretation of the references to colour in this figure legend, the reader is referred to the web version of this article.)

All included studies defined outcomes using clinically comparable criteria. For cancer outcomes, pooling was restricted to incident cases of HCC and gallbladder cancer, excluding studies reporting mortality or other non-equivalent endpoints. Similarly, standardized WHO definitions were used for outcomes such as stunting and underweight.

Between-study heterogeneity ranged from low to high (I^2^: 0%–87.1%; τ^2^: 0.00–1.12). Prediction intervals varied substantially across outcomes: gallbladder cancer [0.37–39.01], stunting [0.82–1.74], pregnancy anemia [0.11–30.31], liver cirrhosis [0.20–79.69], underweight [0.08–22.74], HCC (plasma/serum) [1.03–5.19], and HCC (urine) [1.43–4.95]. For liver function biomarkers, prediction intervals were wide and included the null: AST [−0.93–0.96], ALT [0.09–0.82], and total bilirubin [−0.85–0.93]. Notably, only the prediction intervals for HCC (both plasma/serum and urine) excluded the one value, indicating consistent positive associations across studies. Individual forest plots are provided in Supplementary Figs. 18–25.

### 3.6 Sensitivity analyses

Across the studies included in the *meta*-analyses, the RoB varied depending on the outcome assessed. To further assess robustness, a leave-one-out *meta*-analyses for each outcome was performed (see Supplementary Table 50).

For liver cirrhosis in relation to AF exposure, one study (Kirk et al., 2005) was classified as having a high RoB, while the remaining studies were of low RoB. Exclusion of this study reduced the pooled OR from 3.98 [95% CI: 1.17–13.55] to 2.77 [95% CI: 0.89–8.57], rendering the association non-significant. Additionally, heterogeneity decreased from I^2^ = 82% to 64%.

For HCC in relation to AF exposure measured in urine, one study (Lu, 2010) (Longitudinal study of aflatoxin exposure in the development of primary liver cancer in patients with chronic hepatitis) had a high RoB, while the remaining three studies were of low RoB. Excluding this study increased the pooled RR from 2.66 [95% CI: 1.80–3.93] to 3.26 [95% CI: 2.10–5.06], with the association remaining statistically significant. Heterogeneity remained absent (I^2^ = 0%). The change was less pronounced for HCC in relation to AF exposure measured in plasma or serum. Two studies were classified as high RoB and three as having uncertain RoB. Sensitivity analyses excluding high RoB studies (Kirk, 2005 and Lu, 1998) (Kirk et al., 2005, Lu, 1998) did not materially alter the pooled estimates. Similarly, exclusion of studies with uncertain RoB (Zheng et al., 2017, Mizrak et al., 2009, Kimbi et al., 2005) yielded stable results. For liver function outcomes, all included studies were classified as having a low risk of bias. However, sensitivity analyses revealed that specific studies contributed substantially to between-study heterogeneity. In particular, omission of Wild (1993) in the ALT analysis markedly reduced heterogeneity. A similar pattern was observed for AST, where exclusion of Farag et al. (2018) substantially decreased heterogeneity, and for bilirubin, where omission of Abdel-Wahab (2008) led to a notable reduction in heterogeneity. These findings suggest that, despite an overall low risk of bias, certain studies may disproportionately influence heterogeneity, likely due to differences in study design or sample size.

Similarly, for pregnancy-related anaemia, all three included studies were classified as low RoB. For gallbladder cancer, three studies were of low RoB and one study (Nogueira et al., 2015) had an uncertain RoB. Exclusion of this study reduced the pooled OR from 3.82 [95% CI: 1.08–13.53] to 2.64 [95% CI: 1.19–5.88]. Notably, the prediction interval narrowed substantially from 0.37 to 39.01 to 1.18–5.91, and heterogeneity decreased from I^2^ = 63.5% to 0%. However, exclusion of (Koshiol et al., 2017, Koshiol et al., 2024) or (Yadav et al., 2025) made the effect size statistically insignificant.

For underweight, three studies were of low RoB and one study (Ashraf, 2022) (Ashraf et al., 2022) had an uncertain RoB. Exclusion of this study reduced the pooled OR from 1.33 to 1.04, with the 95% CI remaining non-significant. Heterogeneity decreased from I^2^ = 82.5% to 56.8%. For stunting, among seven studies, one was classified as high RoB, one as uncertain RoB, and the remainder as low RoB. Exclusion of Ashraf, 2022 (Ashraf et al., 2022) resulted in a statistically significant pooled estimate (OR = 1.15; 95% CI: 1.01–1.31), compared to the original non-significant estimate (OR = 1.10; 95% CI: 0.98–1.24). A further model excluding Andrews-Trevino et al. (2019) yielded an OR of 1.13 (95% CI: 0.99–1.29), while heterogeneity increased to I^2^ = 75%.

Furthermore, funnel plots and Egger’s test (≥10 studies) indicated potential bias for the analysis of AF in plasma/serum in relation to HCC (Egger’s p = 0.0015), which was supported by Begg’s test (p = 0.0085) **(**Fig. 4**).** Smaller studies with negative or null results were likely underrepresented. Trim-and-fill analysis estimated 5 missing studies, yielding an adjusted OR of 1.94 [1.46–2.59], compared with the original pooled OR of 2.31 [1.76–3.03]. The modest attenuation of the effect estimate and the persistence of statistical significance suggest that the main findings are robust to potential publication bias.

**Fig. 4:**
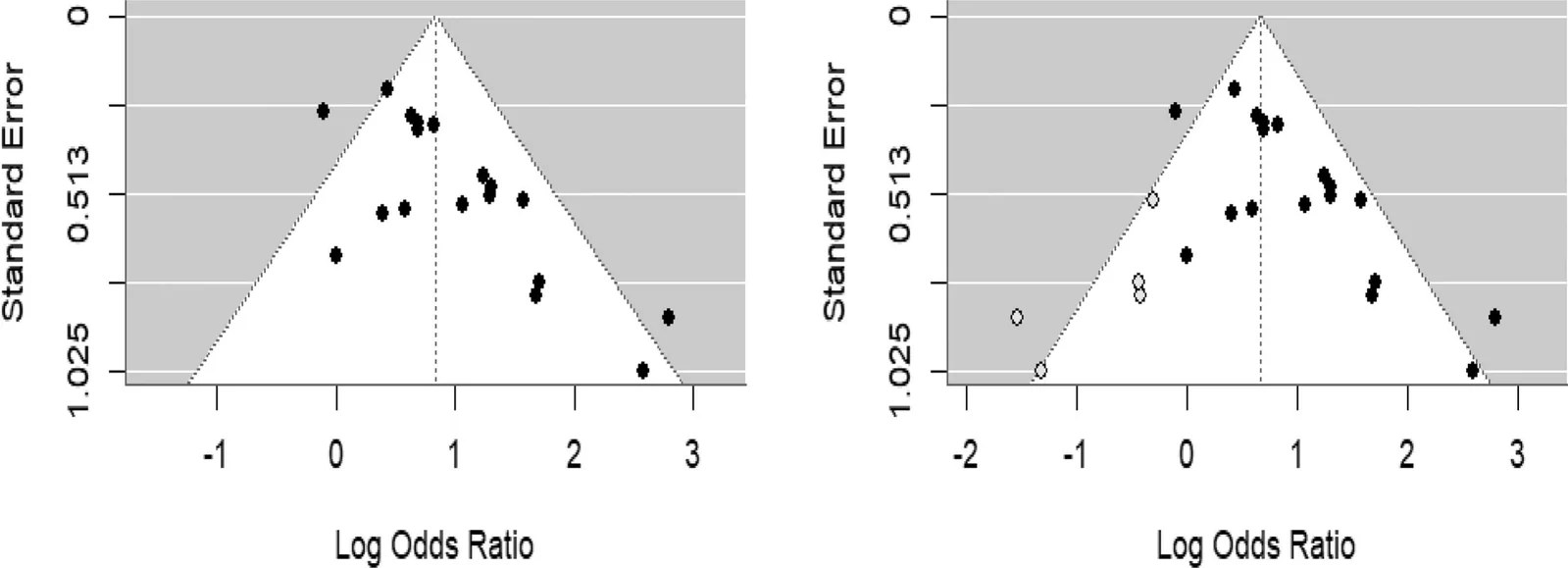
**Funnel plot investigating publication bias in AF analyzed in plasma or serum in relation to HCC.** Left plot represents the original data, while the right plot represent the adjusted funnel plot after employing the trim-and-fill method by adding 4 studies to the left of the plot.

### 3.7 Other mycotoxin-outcome associations

For toxin-outcome pairs excluded from the quantitative *meta*-analysis, associations were summarized using effect direction plots (Supplementary Figs. 26–45). A total of 17 distinct mycotoxin groups − including multiple mycotoxin exposure − have been identified as (potentially) linked to 71 adverse human health outcomes, a relationship that has not previously been comprehensively summarized.

Significant positive associations were observed between **AFs** and a range of neurological disorders, including chronic fatigue syndrome (CFS), acute encephalopathy, and autism, while a negative association was noted with brain function. AFs were also positively associated with acute food intoxication, metabolic disorders such as metabolic syndrome and type II diabetes, and a spectrum of cancers, including endometrial, oesophageal, gastric, and lung cancers, as well as oral submucous fibrosis. Furthermore, AFs were linked to nutritional disorders such as kwashiorkor and marasmus, chronic kidney disease and nephrotic syndrome, pregnancy-related complications including anaemia and haemorrhage, as well as immunomodulation, stunting, and underweight status. Positive associations were also identified between ***Alternaria*** mycotoxins and oesophageal cancer; citrinin (**CIT**) and male infertility; **DON** and autism, spastic paresis, low birth weight as well as acute vomiting; **FBs** and autism, oesophageal cancer, pregnancy anaemia, stunting, underweight status and microbiota alterations; nivalenol (**NIV**) and spastic paresis; **OTA** and various neurological diseases including Alzheimer’s disease, CSF, autism, Parkinson’s disease, as well as immunomodulation, male infertility, microcephaly, stunting, type II diabetes and kidney diseases including chronic kidney disease, renal failure and nephrotic syndrome; **STC** and Parkinson’s and Alzheimer’s disease; **(H)T-2 toxins** and acute vomiting, CSF and Keshan-Beck disease; **ZEN** and male infertility, premature puberty, and breast, endometrial and colorectal cancer; and **multiple mycotoxin** exposure (n ≥ 3) and stunting as well as oesophageal cancer.

## 4 Discussion

By integrating evidence from 268 studies spanning more than five decades, this study provides a comprehensive and up-to-date synthesis of the relationships between mycotoxin exposure and a broad spectrum of health outcomes. Our findings extend beyond the well-established associations with AF-induced HCC and liver cirrhosis, revealing additional potential outcomes such as gallbladder cancer. Moreover, qualitative evidence suggests that AFs and other mycotoxins are predominantly positively associated with a broad spectrum of adverse health outcomes − spanning neurological, metabolic, oncological, renal, reproductive, and developmental disorders. Together, these findings highlight both the expanding scope of mycotoxin-related health risks and the significant gaps that remain in the current evidence base. The findings aim to guide future research and broaden the focus beyond AFs to include other mycotoxins and co-exposures.

### 4.1 Climate and mycotoxin-related liver disease

Our *meta*-analyses focusing on AF exposure reinforced its established link with liver disease: pooled analyses indicated 3.98- and 2.31-fold higher odds of liver cirrhosis and HCC, respectively, among populations with detectable plasma or serum AF (Farag et al., 2018).

Notably, the prediction interval for HCC was the only outcome that did not include 1, suggesting a consistent effect of AF on HCC development. It should be noted, however, that this association reflects AF exposure as one of the several established risk factors for HCC, alongside hepatitis B/C virus infection, alcohol consumption, etc. Llovet et al. (2021), and while some included studies adjusted for these confounders, others did not. Furthermore, although the metric of HCC among populations with detectable AF in urine was higher (RR = 2.66) than in plasma or serum (OR = 2.31), with the trim-and-fill analysis for serum/plasma suggesting potential publication bias (adjusted OR = 1.94), the serum/plasma findings likely offer a more representative reflection of the chronic exposure patterns necessitated by the long latency period of HCC. In addition, studies focusing on AFB1-albumin adducts provide a 2–3 month integration of exposure, reducing the risk of exposure misclassification inherent with short-term urinary measurements (Armstrong, 1998).

Geographically, regions with high AF exposure largely overlap with areas of elevated HCC incidence-particularly Southeast Asia and sub-Saharan Africa. Evidence from this SR reflects this pattern, with most AF-related liver disease studies originating in these regions. However, climate projections indicate that a 2 °C global temperature rise could significantly increase AF contamination in maize across Southern and Eastern Europe ^4^, underscoring a critical research gap in Europe and the Americas and the need for more primary studies in these areas.

^4^In addition, our review found that the role of mycotoxins beyond AFs in liver disease has been relatively underexplored. Only two epidemiological studies examined OTA or FBs in relation to HCC, yielding inconsistent results: one study reported an association between OTA and HCC (Ibrahim et al., 2013), while the European Prospective Investigation Into Cancer and Nutrition cohort study did not; the latter observed a positive association with DON exposure (Huybrechts et al., 2024). Interestingly, climate change is altering the established geographic distribution of *Fusarium* mycotoxins in European wheat-where DON has historically been prevalent in western and northern regions and FB1 in the south- causing these toxins to appear in regions previously considered low-risk (Johns et al., 2022). These observations underscore the need to expand research beyond AF and to monitor emerging mycotoxins globally.

### 4.2 Gallbladder cancer

Although *meta*-analysis suggested a 3.82-fold higher risk of gallbladder cancer associated with serum or plasma AF exposure, this finding lacked robustness as the prediction interval was wide and included 1. The included studies (n = 4) were rated as having moderate certainty in the GRADE assessment but showed sensitivity to study omission. Despite 122,491 new cases reported globally in 2022 (Gallbladder cancer statistics | World Cancer Research Fund), the amount of conducted studies was low, with only one study from Chile and none from Bolivia, regions with high gallbladder cancer incidence and AF exposure potential based on their warm humid climate that favour *Aspergillus* growth and limited storage infrastructure (Gallbladder cancer statistics | World Cancer Research Fund, Foerster et al., 2021, Ramos-diaz et al., 2021). This pattern reflects a broader bias in the exposure map, which is heavily clustered in China, and East- and South-Africa, while regions in South-America remain underrepresented with weak regulation on mycotoxin exposure (Foerster et al., 2024). Such geographical gaps limit a comprehensive understanding of AF-related gallbladder carcinogenesis and highlight the need for large-scale cohort studies in underrepresented regions to enhance preliminary evidence and address potential reporting bias.

### 4.3 Other exposure-outcome pairs

Associations have been identified between exposure to various mycotoxins and a spectrum of adverse health outcomes, including neurological, metabolic, reproductive, and cancer-related disorders. Although findings indicate that mycotoxin exposure may affect multiple organ systems, much of the available evidence is constrained by methodological limitations, which is reflected in the certainty assessment − where most outcomes were rated as low to very low due to issues such as inconsistency, indirectness, and potential publication bias. Broader, well-controlled investigations are needed to confirm these associations and clarify the underlying biological mechanisms. Furthermore, inconsistent associations were observed across studies examining AFs and various health outcomes. Specifically, mixed results were reported between AF exposure and acute aflatoxicosis (3 studies (Jolly et al., 2007, Kamala et al., 2018, Krishnamachari et al., 1975) showing positive associations and 5 showing no effect (Hadidane et al., 1985, Samuel, 2009, An outbreak of acute hepatic encephalopathy due to severe aflatoxicosis in Malaysia - PubMed, Follow-up study of aflatoxic hepatitis in parts of western India - PubMed, Nyikal, 2004), chronic kidney disease (1 (Karamkhani et al., 2020) out of 5 studies reporting positive findings, with the remainder null), kwashiorkor (4 positive (Hendrickse, 1984, Hendrickse et al., 1982, McMillan et al., 2018, Lamplugh and Hendrickse, 1982) out of 17 studies, with the remainder showing null findings), marasmic kwashiorkor (2 positive (Hendrickse, 1984, Hendrickse et al., 1982) out of 6 studies, with the remainder showing null findings), marasmus (3 positive (Hendrickse, 1984, Hendrickse et al., 1982, McMillan et al., 2018) out of 11 studies, with the remainder showing null findings), as well as growth outcomes such as underweight (10 (Kamala et al., 2018, Andrews-Trevino et al., 2022, Gichohi-Wainaina et al., 2023, Gong et al., 2002, Magoha et al., 2014, Nasir et al., 2021, Shirima et al., 2015, Tesfamariam et al., 2022, Turner et al., 2007, Watson et al., 2018) positive out of 20 studies, with the remainder showing null findings) and stunting (19 (Andrews-Trevino et al., 2019, Andrews-Trevino et al., 2022, Gichohi-Wainaina et al., 2023, Gong et al., 2002, Magoha et al., 2014, Shirima et al., 2015, Turner et al., 2007, Watson et al., 2018, Voth-Gaeddert et al., 2018, Shouman et al., 2011, Mitchell et al., 2017, Matchado et al., 2023, Mahfuz et al., 2021, Mahdavi et al., 2008, Leroy et al., 2018, Kimanya et al., 2010, Huong et al., 2019, Gong et al., 2004, Andrews-Trevino et al., 2021) positive of 27 studies, with the remainder showing null findings)). Similarly, results for OTA and chronic kidney disease were inconsistent (2 (Hsieh et al., 2004, Abid et al., 2003) studies showing positive associations and remaining 6 reporting no effect (Thé et al., 2015, Schwerdt et al., 2001, Özçelik et al., 2001, Hassan et al., 2005, Desalegn et al., 2011, Dinis et al., 2007), indicating uncertain evidence for a causal relationship. For ZEN and breast cancer, only one (Belhassen et al., 2015) study reported a positive association while the rest found no effect, suggesting limited and weak evidence for this connection. Regarding FBs, available studies examining their relationship with stunting show mixed or inconclusive results (3 (Shirima et al., 2015, Kimanya et al., 2010, Huong et al., 2019) positive out of 6 studies, with the remaining showing null findings). Overall, these inconsistencies underscore the need for well-controlled longitudinal studies employing causal inference approaches to clarify the relationships between mycotoxin exposure and health outcomes. Ongoing work by our research team focuses on elucidating causal pathways linking stunting with AF exposure.

### 4.4 Multiple mycotoxin exposures

Co-occurrence of multiple mycotoxins is common due to shared fungal sources or contamination of the same food matrix (De Ruyck et al., 2015). Such mixtures may exert additive, synergistic, or antagonistic toxic effects (De Ruyck et al., 2015), yet only 11% of mycotoxin-health association studies embedded multiple mycotoxin exposure. This may be particularly important, as illustrated by one study on oesophageal cancer reporting stronger associations with multi-mycotoxin exposure than with individual toxins, highlighting potential synergistic effects (Mulisa et al., 2025). Future research must account for these real-world exposure scenarios to better estimate population-level risks.

### 4.5 Geographical and methodological gaps in mycotoxin research

Existing mycotoxin research is predominantly concentrated in Africa, Asia and the United States, with limited data from Europe, Oceania, and South America. This is concerning given high mycotoxin exposure potential in the Southern European and American regions (Battilani, 2016). European biomonitoring studies indicate intra- and inter-country variability, while climate change may exacerbate contamination in Europe and North America (Battilani et al., 2016, Moretti et al., 2019, Johns et al., 2022). Expanding geographical coverage is essential for accurate risk assessment and global food safety strategies. Furthermore, primary research is heavily focused on AFs, neglecting the potential synergistic effects of multiple mycotoxin co-exposure and limiting the scope of other relevant toxins like OTA, DON and FBs. Methodologically, the *meta*-analyses are impacted by study heterogeneity and variability in analytical methods, with older techniques (*i.e.* TLC and HPLC-UV) reporting larger associations than advanced methods like (UP)LC-MS/MS (Iqbal, 2021). Variability in biological sampling and analytical infrastructure across regions may also contribute to differences in observed associations. Furthermore, many of the included studies suffer from quality issues, reporting only prevalence or unadjusted comparisons, leading to inconsistent results for some outcomes (*e.g.* AF and kwashiorkor), and many newly highlighted associations rest on single studies, severely limiting their generalizability. Ultimately, the lack of harmonized epidemiological methods and limited adherence to FAIR (Findable, Accessible, Interoperable, Reusable) data principles restrict the integration of data and comprehensive risk assessment.

### 4.6 Strengths and limitations of this SR

A key strength of this SR lies in its comprehensive scope, integrating diverse data sources across multiple countries and encompassing all types of mycotoxin exposures, including both single compounds and mixtures. It offers the most complete overview to date of mycotoxins’ health effects, including noncommunicable and climate-sensitive diseases. By addressing major methodological and surveillance gaps, it advances understanding in an underexplored area of mycotoxin risk. Unlike previous reviews that typically focused on a single outcome or specific mycotoxin group (Claeys et al., 2020, Gong et al., 2016), this study offers a truly integrative overview of the epidemiological evidence to date.

Applying the GRADE framework clarifies what this assessment adds to the field. Although all mycotoxin-related health outcomes evaluated were rated as having very low certainty of evidence, the underlying reasons for these ratings differed substantially across outcomes. For premature puberty, the certainty of evidence was downgraded solely for imprecision, reflecting small sample sizes (<100 participants) and the absence of reported power calculations, and highlighting a clear need for larger, adequately powered studies. In contrast, the very low certainty for liver disease was driven primarily by inconsistency, characterized by widely varying effect estimates and moderate-to-high statistical heterogeneity, and indirectness arising from differences across the PECO elements, with additional downgrading for imprecision and publication bias. By distinguishing these different methodological limitations, the assessment goes beyond concluding that the overall evidence is weak: it identifies the specific gaps that future epidemiological research should address to strengthen the evidence base.

Nonetheless, several limitations should be noted. The lack of consistently reported effect sizes across studies hindered direct comparability and precluded *meta*-analyses for several outcomes. Meta-regression was not possible for outcomes such as pregnancy anemia and wasting due to insufficient study numbers (n < 10), despite high heterogeneity. In addition, although *meta*-analyses were restricted to studies reporting clinically comparable endpoints (*e.g.* incident cancer cases and standardized definitions for anthropometric outcomes), some residual heterogeneity in outcome definitions across studies cannot be entirely excluded. Differences in diagnostic criteria, outcome ascertainment, or reporting (*e.g.* hospital-based diagnosis versus registry-based identification) may have contributed to variability in effect estimates. While pooling fundamentally different endpoints such as incidence and mortality was avoided, these remaining differences should be considered when interpreting the pooled results. More fundamentally, the certainty of the overall evidence is limited by risk of bias and by indirectness arising from a frequent mismatch between study design and the temporal nature of the outcome. Chronic, long-latency conditions such as cancer require prospective cohort or well-conducted case–control designs establishing that exposure preceded disease onset, whereas cross-sectional designs are informative mainly for acute or short-latency outcomes. In this evidence base, design and outcome were appropriately aligned for only three endpoints − acute food intoxication, gallbladder cancer and immunomodulation − while most remaining outcomes, including several cancers, rely predominantly on cross-sectional studies in which exposure and disease were ascertained concurrently. This is particularly problematic because mycotoxin biomarkers reflect only recent intake and cannot be assumed to represent the aetiologically relevant exposure window, and because disease-related changes in diet or hepatic and renal function may themselves alter biomarker concentrations (reverse causation). Together with residual confounding, this design-related indirectness downgrades the certainty of evidence for every outcome assessed, which should therefore be regarded as largely hypothesis-generating. The same limitation precluded any formal assessment of time coherence. Only 10 of the 268 included studies used a prospective design with a documented interval between exposure assessment and diagnosis, and the timing of biomarker sampling relative to disease onset was rarely reported in a standardised way. Lagged sensitivity analyses are therefore currently infeasible in mycotoxin epidemiology, constrained not only by the small number of prospective studies but by the reporting conventions of the field. Standardised reporting of exposure–outcome timing would be a low-cost improvement with substantial analytical benefit. Furthermore, overall evidence remains concentrated on AFs and OTA, with limited data on emerging or modified mycotoxins, and many studies examined only single mycotoxins, restricting assessment of real-world co-exposure and synergistic effects (De Ruyck et al., 2015). Notably, exposure assessment was often based on one or two biological matrices, limiting the ability to capture the full spectrum of mycotoxin exposure, particularly given differences in biomarker pharmacokinetics (De Ruyck, 2020). Finally, variability in analytical sensitivity across studies may have further contributed to exposure misclassification, potentially influencing the observed associations with disease outcomes.

## 5 Conclusion

This SR highlights the potential significance of 17 mycotoxin classes as determinants of 71 human health outcomes. Evidence is most consistent for AF-associated HCC, the only outcome for which the prediction interval excluded the null, although this should be interpreted with caution given the limited adjustment for established co-risk factors across studies. For the remaining outcomes, associations are frequently characterized by wide prediction intervals, substantial heterogeneity, and few contributing studies; for gallbladder cancer, pregnancy anemia and growth outcomes, prediction intervals include the null.

Formal appraisal reinforces this picture. Although 74% of the 268 included studies were rated at low risk of bias at the individual study level, the certainty of the overall body of evidence was very low for all 21 outcomes assessed. This apparent discrepancy is informative: the limiting factor is not the internal conduct of individual studies but the design of the evidence base as a whole, in which cross-sectional biomarker measurement predominates and rarely establishes that exposure preceded disease. The associations reported here should therefore be regarded as hypothesis-generating rather than causal.

Addressing this will require prospective designs matched to the latency of the outcome under study, standardized exposure assessment and reporting of exposure–outcome timing, and strategies that account for co-exposure to multiple mycotoxins. The intersection of climate change, global food trade, and dietary transitions further underscores the relevance of these exposures for global population health, and closing the methodological, geographic and data-sharing gaps identified here will be critical to inform targeted interventions and protect vulnerable populations worldwide.

## CRediT authorship contribution statement

**Tess Goessens:** Writing – original draft, Validation, Supervision, Software, Project administration, Methodology, Investigation, Funding acquisition, Formal analysis, Data curation, Conceptualization. **Yasmine Bader:** Writing – review & editing, Visualization, Validation, Investigation, Formal analysis, Data curation. **Febe Vertriest:** Writing – review & editing, Validation, Investigation, Formal analysis, Data curation. **Kokeb Tesfamariam:** Writing – review & editing, Methodology, Formal analysis. **Sarah De Saeger:** Writing – review & editing, Supervision, Funding acquisition. **Carl Lachat:** Writing – review & editing, Supervision, Methodology, Funding acquisition, Formal analysis, Conceptualization. **Marthe De Boevre:** Writing – review & editing, Supervision, Funding acquisition, Conceptualization.

## Funding

This research was funded by the European research Council (ERC) under the European Union’s Horizon 2020 research and innovation program (grant agreement No 946192, HUMYCO), and the 10.13039/501100003130Research Foundation Flanders (FWO, Fellowship 1204324N, 11PRI24N and G064324N).

## Declaration of competing interest

The authors declare that they have no known competing financial interests or personal relationships that could have appeared to influence the work reported in this paper.

## Data Availability

All data related to this manuscript can be consulted open-access via https://osf.io/n5etv/.
