# Supplementary material for: Systematic review and meta-analysis of adverse health effects associated with mycotoxin exposure
Source: Environ Int. 2026 Oct;216:110540. doi: 10.1016/j.envint.2026.110540 (PMC13690690; doi:10.1016/j.envint.2026.110540)
Supplement: Supplementary file 1 [file mmc1.docx]

**Supplementary Materials – Systematic Review and Meta-analysis of Adverse Health Effects associated with Mycotoxin Exposure**

Tess Goessens^1^, Yasmine Bader^1,4^, Febe Vertriest^1,2^, Kokeb Tesfamariam^1^, Sarah De Saeger^1,5^, Carl Lachat^3^, Marthe De Boevre^1^.

^1^ Centre of Excellence in Mycotoxicology and Public Health, Faculty of Pharmaceutical Sciences, Ghent University, Ghent, Belgium.

^2^ Department of Internal Medicine and Pediatrics, Faculty of Medicine and Health Sciences, Ghent University, Ghent, Belgium.

^3^ Department of Food Technology, Safety and Health, Faculty of Bioscience Engineering, Ghent University, Ghent, Belgium.

^4^ Nutrition epidemiology group, International Agency for Research on Cancer, Lyon, Rhône Alpes, France.

^5^Department of Biotechnology and Food Technology, Faculty of Science, University of Johannesburg, Doornfontein Campus, Gauteng, Johannesburg, South Africa.

**Table of Contents**

[**Table 1 Dietary mycotoxins**	8](#_Toc229671835)

[**Table 2 Studies without full text (n = 36).**	10](#_Toc229671836)

[**Table 3 Acute food intoxication**	1](#_Toc229671837)

[**Table 4 Gallbladder cancer**	2](#_Toc229671838)

[**Table 5 Bladder cancer**	3](#_Toc229671839)

[**Table 6 Kidney disease**	4](#_Toc229671840)

[**Table 7 Immunomodulation**	8](#_Toc229671841)

[**Table 8 Liver disease**	9](#_Toc229671842)

[**Table 9 Neurologic disease**	20](#_Toc229671843)

[**Table 10 Metabolic disease**	24](#_Toc229671844)

[**Table 11 Female infertility**	25](#_Toc229671845)

[**Table 12 Premature puberty**	25](#_Toc229671846)

[**Table 13 Female reproductive disease (breast & endometrial cancer)**	27](#_Toc229671847)

[**Table 14 Male reproductive disease**	28](#_Toc229671848)

[**Table 15 Musculoskeletal disease**	29](#_Toc229671849)

[**Table 16 Mucous Fibrosis**	30](#_Toc229671850)

[**Table 17 Oesophageal disease**	30](#_Toc229671851)

[**Table 18 Gastric disease**	31](#_Toc229671852)

[**Table 19 Intestinal disease**	32](#_Toc229671853)

[**Table 20 Wasting**	34](#_Toc229671854)

[**Table 21 Underweight**	34](#_Toc229671855)

[**Table 22 Stunting**	37](#_Toc229671856)

[**Table 23 Adverse birth outcomes**	39](#_Toc229671857)

[**Table 24 Kwashiorkor**	42](#_Toc229671858)

[**Table 25 Blood diseases**	46](#_Toc229671859)

[**Table 26 Lung diseases**	47](#_Toc229671860)

[**Table 27 Pancreatic cancer**	48](#_Toc229671861)

[**Table 28 Detailed overview, including the methodology & rationale for decisions of the RoB assessment of the included eligible studies.**	49](#_Toc229671862)

[**Table 29 Detailed overview, including the criteria & rationale for decisions of the GRADE assessment of the overall body of evidence of this SR for acute food intoxication (Morgan et al., 2016).**	81](#_Toc229671863)

[**Table 30 Detailed overview, including the criteria & rationale for decisions of the GRADE assessment of the overall body of evidence of this SR for liver disease (Morgan et al., 2016).**	82](#_Toc229671864)

[**Table 31 Detailed overview, including the criteria & rationale for decisions of the GRADE assessment of the overall body of evidence of this SR for gallbladder cancer (Morgan et al., 2016).**	83](#_Toc229671865)

[**Table 32 Detailed overview, including the criteria & rationale for decisions of the GRADE assessment of the overall body of evidence of this SR for metabolic disease (Morgan et al., 2016).**	84](#_Toc229671866)

[**Table 33 Detailed overview, including the criteria & rationale for decisions of the GRADE assessment of the overall body of evidence of this SR for neurological disease (Morgan et al., 2016).**	85](#_Toc229671867)

[**Table 34 Detailed overview, including the criteria & rationale for decisions of the GRADE assessment of the overall body of evidence of this SR for male reproductive disease (Morgan et al., 2016).**	86](#_Toc229671868)

[**Table 35 Detailed overview, including the criteria & rationale for decisions of the GRADE assessment of the overall body of evidence of this SR for female reproductive disease (Morgan et al., 2016).**	87](#_Toc229671869)

[**Table 36 Detailed overview, including the criteria & rationale for decisions of the GRADE assessment of the overall body of evidence of this SR for premature puberty (Morgan et al., 2016).**	88](#_Toc229671870)

[**Table 37 Detailed overview, including the criteria & rationale for decisions of the GRADE assessment of the overall body of evidence of this SR for kidney disease (Morgan et al., 2016).**	89](#_Toc229671871)

[**Table 38 Detailed overview, including the criteria & rationale for decisions of the GRADE assessment of the overall body of evidence of this SR for bladder cancer (Morgan et al., 2016).**	90](#_Toc229671872)

[**Table 39 Detailed overview, including the criteria & rationale for decisions of the GRADE assessment of the overall body of evidence of this SR for lung disease (Morgan et al., 2016).**	91](#_Toc229671873)

[**Table 40 Detailed overview, including the criteria & rationale for decisions of the GRADE assessment of the overall body of evidence of this SR for immunomodulation (Morgan et al., 2016).**	92](#_Toc229671874)

[**Table 41 Detailed overview, including the criteria & rationale for decisions of the GRADE assessment of the overall body of evidence of this SR for kwashiorkor (Morgan et al., 2016).**	94](#_Toc229671875)

[**Table 42 Detailed overview, including the criteria & rationale for decisions of the GRADE assessment of the overall body of evidence of this SR for wasting (Morgan et al., 2016).**	95](#_Toc229671876)

[**Table 43 Detailed overview, including the criteria & rationale for decisions of the GRADE assessment of the overall body of evidence of this SR for underweight (Morgan et al., 2016).**	96](#_Toc229671877)

[**Table 44 Detailed overview, including the criteria & rationale for decisions of the GRADE assessment of the overall body of evidence of this SR for stunting (Morgan et al., 2016).**	97](#_Toc229671878)

[**Table 45 Detailed overview, including the criteria & rationale for decisions of the GRADE assessment of the overall body of evidence of this SR for blood disease (Morgan et al., 2016).**	98](#_Toc229671879)

[**Table 46 Detailed overview, including the criteria & rationale for decisions of the GRADE assessment of the overall body of evidence of this SR for oesophageal disease (Morgan et al., 2016).**	99](#_Toc229671880)

[**Table 47 Detailed overview, including the criteria & rationale for decisions of the GRADE assessment of the overall body of evidence of this SR for gastric disease (Morgan et al., 2016).**	100](#_Toc229671881)

[**Table 48 Detailed overview, including the criteria & rationale for decisions of the GRADE assessment of the overall body of evidence of this SR for intestinal disease (Morgan et al., 2016).**	101](#_Toc229671882)

[**Table 49 Detailed overview, including the criteria & rationale for decisions of the GRADE assessment of the overall body of evidence of this SR for adverse birth outcomes (Morgan et al., 2016).**	102](#_Toc229671883)

[**Table 50 Leave-one-out meta-analysis on all outcomes without metric transformation.**	103](#_Toc229671884)

[**Figure 1. Traffic light plot and Risk of bias (RoB) summary plot for adverse birth outcomes.**	106](#_Toc229671885)

[**Figure 2. Traffic light plot and Risk of bias (RoB) summary plot for acute food intoxication.**	107](#_Toc229671886)

[**Figure 3. Traffic light plot and Risk of bias (RoB) summary plot for female reproductive disease.**	108](#_Toc229671887)

[**Figure 4. Traffic light plot and Risk of bias (RoB) summary plot for oesophageal disease.**	109](#_Toc229671888)

[**Figure 5. Traffic light plot and Risk of bias (RoB) summary plot for gallbladder cancer.**	110](#_Toc229671889)

[**Figure 6. Traffic light plot and Risk of bias (RoB) summary plot for gastric disease.**	111](#_Toc229671890)

[**Figure 7. Traffic light plot and Risk of bias (RoB) summary plot for liver disease.**	112](#_Toc229671891)

[**Figure 8. Traffic light plot and Risk of bias (RoB) summary plot for immunomodulation.**	113](#_Toc229671892)

[**Figure 9. Traffic light plot and Risk of bias (RoB) summary plot for female infertility.**	114](#_Toc229671893)

[**Figure 10. Traffic light plot and Risk of bias (RoB) summary plot for intestinal disease.**	115](#_Toc229671894)

[**Figure 11. Traffic light plot and Risk of bias (RoB) summary plot for kidney disease and bladder cancer.**	116](#_Toc229671895)

[**Figure 12. Traffic light plot and Risk of bias (RoB) summary plot for kwashiorkor and marasmus.**	117](#_Toc229671896)

[**Figure 13. Traffic light plot and Risk of bias (RoB) summary plot for lung disease, muscuskeletaral disease, pancreatic cancer, male reproductive disease, and mucous fibrosis.**	118](#_Toc229671897)

[**Figure 14. Traffic light plot and Risk of bias (RoB) summary plot for neurological disease.**	119](#_Toc229671898)

[**Figure 15. Traffic light plot and Risk of bias (RoB) summary plot for blood disease.**	120](#_Toc229671899)

[**Figure 16. Traffic light plot and Risk of bias (RoB) summary plot for premature puberty and metabolic disease.**	121](#_Toc229671900)

[**Figure 17. Forest plot with random effects model of aflatoxin exposure detected in plasma or serum samples in relation to underweight in children**.	124](#_Toc229671901)

[**Figure 18. Forest plot with random effects model of aflatoxin exposure detected in plasma or serum samples in relation to liver cirrhosis.**	125](#_Toc229671902)

[**Figure 19. Forest plot with random effects model of aflatoxin exposure detected in plasma or serum samples in relation to gallbladder cancer.**	126](#_Toc229671903)

[**Figure 20. Forest plot with random effects model of aflatoxin exposure detected in plasma or serum samples in relation to stunting.**	127](#_Toc229671904)

[**Figure 21. Forest plot with random effects model of aflatoxin exposure detected in plasma or serum samples in relation to pregnancy anaemia.**	128](#_Toc229671905)

[**Figure 22. Forest plot with random effects model of aflatoxin exposure detected in plasma or serum samples in relation to hepatocellular carcinoma.**	129](#_Toc229671906)

[**Figure 23. Forest plot with random effects model of aflatoxin exposure detected in urine in relation to hepatocellular carcinoma.**	130](#_Toc229671907)

[**Figure 24. Forest plot with random effects model of aflatoxin exposure detected in plasma or serum samples in relation to AST/ALT/Bilirubin increase.**	131](#_Toc229671908)

[**Figure 25. Effect direction plot for acute food intoxication.**	132](#_Toc229671909)

[**Figure 26. Effect direction plot for metabolic disease.**	133](#_Toc229671910)

[**Figure 27. Effect direction plot for neurologic disease.**	135](#_Toc229671911)

[**Figure 28. Effect direction plot for female reproductive disease and cancer.**	136](#_Toc229671912)

[**Figure 29. Effect direction plot for male infertility.**	137](#_Toc229671913)

[**Figure 30. Effect direction plot for premature puberty.**	138](#_Toc229671914)

[**Figure 31. Effect direction plot for testicular and prostate cancer.**	139](#_Toc229671915)

[**Figure 32. Effect direction plot for testicular and oesophageal disease.**	140](#_Toc229671916)

[**Figure 33. Effect direction plot for gastro-intestinal disease.**	141](#_Toc229671917)

[**Figure 34. Effect direction plot for gallbladder, cholangiocarcinoma and pancreatic cancer.**	142](#_Toc229671918)

[**Figure 35. Effect direction plot for lung diseases.**	143](#_Toc229671919)

[**Figure 36. Effect direction plot for kwashiorkor and marasmus.**	144](#_Toc229671920)

[**Figure 37. Effect direction plot for kidney disease and bladder cancer.**	146](#_Toc229671921)

[**Figure 38. Effect direction plot for adverse birth outcomes.**	147](#_Toc229671922)

[**Figure 39. Effect direction plot for wasting.**	148](#_Toc229671923)

[**Figure 40. Effect direction plot for musculoskeletal and mucous fibrosis disease.**	149](#_Toc229671924)

[**Figure 41. Effect direction plot for immunomodulation.**	150](#_Toc229671925)

[**Figure 42. Effect direction plot for stunting and underweight.**	152](#_Toc229671926)

[**Figure 43. Effect direction plot for liver diseases.**	156](#_Toc229671927)

[**Figure 44. Effect direction plot for blood diseases.**	157](#_Toc229671928)

# **Table 1 Dietary mycotoxins**

| **Fungal species** | **Mycotoxins** | **Food products** |
| --- | --- | --- |
| *Aspergillus spp.* | Aflatoxins: AFB1, AFB2, AFG1, AFG2, AFM1  Aspertoxin  Aspergillic acid  Sterigmatocystin  Hydroxy-aflavinine  Ditryptophenaline  Flavacol  Hydroxyneoaspergillic acid  Leporin C  Methylcitreo-isocoumarin  Paspalinine  Speradine A  Versiconol | Aflatoxins, aspertoxin & aspergillic acid: Maize, rice, nuts, oilseeds, dried fruit and spices ^1,2^.  Sterigmatocystin: cereal products, pistachio nuts, pecan nuts & green coffee beans ^3^.  Hydroxy-aflavinine: tea & dried fruits ^4^.  Ditryptophenaline: peanuts ^5^.  Flavacol: maize ^4,6^.  Hydroxyneoaspergillic acid, leporin C, methylcitreo-isocoumarin, paspalinine, speradine A & versiconol: maize ^6^. |
| *Fusarium spp.* | Trichothecenes: T2, HT2, NIV, DON, 15-ADON, 3-ADON, fusarenon X, neosolaniol, NX toxin, verrucarin  Fumonisins: FB1, FB2 and FB3  Enniatins: ENNA, ENNA1, ENNB and ENNB1  BEA  Zearalenone and metabolites: ZEN, ZAN, α-ZAL, α-ZEL, β-ZAL and β-ZEL  DAS  Fusaric acid  Fusarin C  Moniliformin | Trichothecenes: cereal products, rice, peanuts & maize ^7^.  Fumonisins: cereal products, rice, asparagus, garlic, barley foods, beers, dried figs, milk, maize & black tea ^8^.  Enniatins & BEA: cereal products, nuts, dried fruits & bananas ^9^.  ZEN: Wheat, barley, maize, sorghum, rye, rice, corn silage, sesame seed, hay, flour, malt, soybeans, beer, and corn oil ^10^.  DAS: cereal products ^11^.  Fusaric acid: maize & tomatoes ^12–14^.  Fusarin C: maize ^14^.  Moniliformin: cereal products^15^. |
| *Penicillium spp.*  *Aspergillus spp.* | Ochratoxins: OTA A, B and C  Cyclopiazonic acid  Penicillic acid | Ochratoxins: cereal products, coffee, vegetables, liquorice, raisins, wine, nuts, pork & poultry ^16,17^.  Cyclopiazonic acid: maize, peanuts and cheese ^3^.  Penicillic acid: cereal products ^13^. |
| *Aspergillus spp.*  *Monascus spp.*  *Penicillium spp.* | Citrinin | Cereal products, rice, pomaceous fruits, black olives, roasted nuts, spices, cheese ^17^. |
| *Alternaria spp.* | Alternaria toxins: AME, AOH, TEA, ALT, ATX, TX | Small grain cereals, nuts, tomato fruits, olives, bell peppers, apples, berries, citrus fruits ^18^. |
| *Penicillium spp.* | Cyclochlorotine  Luteoskyrin  Rugulosin  Patulin  Roquefortine C | Cyclochlorotine: ereal products, soybeans, peanuts, beans ^19^.  Luteoskyrin & rugulosin: rice ^20^.  Patulin: apple and apple-based products, occasionally in other fruits such as pears, grapes, oranges and cereal products ^21^.  Roquefortine C: carbonated beverages, beer, wine, meats, blue cheese and bread ^22^. |
| *Claviceps spp.* | Ergot alkaloids: ergometrine, ergocornine, ergocristine, ergotamine, ergosine and ergocryptine | Cereal products ^23^. |
| *Phomopsis spp.* | Phomopsin | Lupin seeds and products ^24^. |

# **Table 2 Studies without full text (n = 36).**

| Reference | Title |
| --- | --- |
| Peers, 1980^25^ | **Aflatoxin in relation to the epidemiology of human liver cancer.** |
| Godin, 1997^26^ | **Is ochratoxin a nephrotoxic in human beings?** |
| Munsterer, 1955^27^ | **Ergot poisoning, a national epidemic in the Middle Ages.** |
| Dvorackova,1979^28^ | **Aflatoxin in the pathology of early childhood.** |
| Amla, 1970^29^ | **Role of aflatoxin in Indian childhood cirrhosis. (A preliminary communication).** |
| Ribarova, 2003^30^ | **Ochratoxin a - Risk factor for human health.** |
| Payet, 1975^31^ | **Aflatoxins and cancer of the liver.** |
| Butler, 1970^32^ | **Liver injury induced by aflatoxin.** |
| Naqvi,1980^33^ | **Aflatoxin and liver disease.** |
| Asgaonkar, 1987^34^ | **Ergot poisoning: a report of two cases.** |
| Huţanaşu, 2009^35^ | **High blood and urine concentration of hepatotoxic mycotoxins (aflatoxin B1, sterigmatocystin) in patients with liver cirrhosis.** |
| Nizami, 1986^36^ | **Urinary excretion of aflatoxin and liver cancer in Karachi.** |
| Olubuyide, 1993^37^ | **HBsAg, aflatoxins and primary hepatocellular carcinoma.** |
| Abulu, 1998^38^ | **Preliminary investigation on aflatoxin in cord blood of jaundiced neonates.** |
| Dhatt, 1982^39^ | **Aflatoxins and Indian childhood cirrhosis.** |
| Petkova-Bocharova, 1988^40^ | **Ochratoxin A in human blood in relation to Balkan endemic nephropathy and urinary system tumours in Bulgaria.** |
| Burguera, 1986^41^ | **Presence of aflatoxin B1 in human liver referred to as Reye's syndrome in Venezuela.** |
| Andrássy, 1986^42^ | **Outbreak of mass mycotoxicosis in Hajdú-Bihar County.** |
| Le Breton, 1970^43^ | **Hepatic cancer and mycotoxins.** |
| Breitholtz, 1994^44^ | **Ochratoxin A in human serum samples collected in southern Italy from healthy individuals and individuals suffering from different kidney disorders.** |
| Nguyen, 1999^45^ | **Risk of liver cancer due to peanut butter?** |
| Terao, 1974^46^ | **Mycotoxicosis caused by aflatoxin B1 and related compounds.** |
| De Vries, 1989^47^ | **Aflatoxins in liver biopsies from Kenya.** |
| Serck-Hanssen,1996^48^ | **Aflatoxin-induced fatal Hepatitis.** |
| Krishnamachari, 1977^49^ | **Problem of aflatoxic human disease in parts of India: epidemiological and ecological aspects.** |
| Dvorácková, 1972^50^ | **Aflatoxin and liver damage with encephalopathy.** |
| Dvorackova, 1974^51^ | **Aflatoxin - possible factor of liver damage in children (Czech).** |
| Karvonen, 1951^52^ | **Buerger's disease and ergotism.** |
| Plestina, 1990^53^ | **Human exposure to ochratoxin A in areas of Yugoslavia with endemic nephropathy.** |
| Nizami, 2004^36^ | **Aflatoxin and liver cancer in Karachi--a preliminary survey. 1977.** |
| Onyemelukwe, 1980^54^ | **Aflatoxin B1 in hepatocellular carcinoma.** |
| Alvarez, 1991^55^ | **Aflatoxin detection in urine in liver diseases in childhood.** |
| Aljicevic, 2010^56^ | **Aflatoxin in the urine of patients suffering from HCC and HBV.** |
| Peers, 1977^57^ | **Dietary aflatoxins and human primary liver cancer.** |
| Bhat, 1997^58^ | **A foodborne disease outbreak due to the consumption of moldy sorghum and maize containing fumonisin mycotoxins.** |
| Dvorackova, 1990^59^ | **Hepatocellular carcinoma (a 28-year necropsy review).** |

**Table 3 Acute food intoxication**

| **Reference** | **Population** | **Study period** | **Study design** | **N(Ca/Co)** | **Gender** | **Age (years)** | **Mycotoxin** | **Method** | **Matrix** | **LOD**  **(LOQ)**  **(µg/L)** | **Conc.**  **(µg/L)** | **OR/RR/CC/AR**  **(95% CI)** | **Prevalence**  **(%)** | **RoB** |
| --- | --- | --- | --- | --- | --- | --- | --- | --- | --- | --- | --- | --- | --- | --- |
| *Acute encephalopathy* | | | | | | | | | | | | | | |
| Shank, 1971^60^ | Thailand | 1971 | CaCo | 38(23/15) | M/F | 1-20 | AFB1 | TLC | Tissue; Urine | 10 | 0-132 | n.r. | Urine: 2-16  Tissue: 11-57 | Prob. High |
| Lye, 1995^61^ | Malaysia | 1988 | OuR | 13 | M/F | n.r. | AFB1 | n.r. | Tissue | n.r. | n.r. | n.r. | 100 | Def. High |
| *Acute gastrointestinal disease* | | | | | | | | | | | | | | |
| Wang, 1993^62^ | China | 1993 | OuR | 97 | n.r. | n.r. | T2 | n.r. | Food | n.r. | 420 | n.r. | n.r. | Def. High |
| Ruan, 2020^63^ | China | 2020 | OuR | 229(87/142) | M/F | 6-9 | DON | UPLC-MS/MS | Food | n.r. | 518-634 | 6^RR^ (2-16)  17^AR^ | n.r. | Def. High |
| Perduri, 2009^64^ | Italy | 2004-2007 | CaR | 1 | F | 38 | AFB1 | n.r. | Food | n.r. | n.r. | n.r. | 100 | Prob. High |
| *Acute liver failure* | | | | | | | | | | | | | | |
| Probst, 2007^65^ | Kenya | 2004 | OuR | 317 | M/F | n.r. | AFB1 | n.r. | Food | n.r. | 95,000 – 212,000 | n.r. | 100 | Def. High |
| Krishnamachari, 1975^66^ | India | 1974 | OuR | 7 | n.r. | n.r. | AFB1 | HPLC-FLD | Urine; Serum; Food | n.r. | n.r. | n.r. | Urine: 0  Serum: 29  Food: 0-100 | Prob. High |
| Hadidane, 1986^67^ | Tunesia | 1986 | Cs | 9 | n.r. | n.r. | AFB1, AFB2, AFG1, AFG2, CIT, OTA, DON, DAN, FUS-X, DON-M | TLC | Food | n.r. | n.r. | n.r. | n.r. | Def. High |
| Tzee-cheng, 1991^68^ | Malaysia | 1988 | OuR | 13 | M/F | 3-11 | AFB1, AFB2, AFM1, AFM2, AFG1, AFL | HPLC-  FLD | Tissue | n.r. | 0-35 | n.r. | n.r. | Prob. Low |
| Nyikal, 2004^69^ | Kenya | 2004 | OuR | 317 | M/F | n.r. | AFB1 | n.r. | Food | n.r. | 14 – 5,760 | 8^OR^  (1.7 – 37.1) | n.r. | Prob. Low |
| Jolly, 2007^70^ | Ghana | 2007 | Cs | 140 | M/F | 19-86 | AFB1-albumin | RIA | Plasma | n.r. | 2-54 | 2-6^OR^  (2-165) | n.r. | Prob. High |
| Samuel, 2009^71^ | Israel | 2009 | CaR | 1 | M | 28 | AFB1 | n.r. | Food | n.r. | n.r. | n.r. | n.r. | Prob. Low |
| Kamala, 2018^72^ | Tanzania | 2016 | OuR | 47(24/23) | M/F | <5-<49 | AFB1, AFB2, AFM1, AFM2, AFG1, FB1, FB2, FB3, ENNB, STG,OTA,AOH,DON, ZEN, T2, HT2, ROQ-C, AME, NEO,FUSX,3ADON, 15ADON | UPLC-MS/MS | Food | 1.2-100 | <LOD – 50,000 | n.r. | 28 - 100 | Prob. High |
| *Alimentary toxic aleukemia* | | | | | | | | | | | | | | |
| Hadidane, 1986^67^ | Tunesia | 1986 | Cs | 9 | n.r. | n.r. | AFB1, AFB2, AFG1, AFG2, CIT, OTA, DON, DAN, FUS-X, DON-M | TLC | Food | n.r. | n.r. | n.r. | n.r. | Def. High |

**Note: N = sample size; Ca = cases; Co = controls; LOD = limit of detection; LOQ = limit of quantification; Conc. = concentration; OR = odds ratio; RR = relative risk; CC = correlation coefficient; AR = attack rate; CI = confidence interval; RoB = Risk of Bias according to OHAT; OuR = outbreak report; Cs = cross-sectional study; CaCo = case-control study; NCC = nested case-control; CaR = case report; n.r. = not reported; TLC = thin layered chromatography; U(H)PLC-MS/MS = ultra(high) performance liquid chromatography - tandem mass spectrometry; HPLC-FLD = High-performance Liquid chromatography fluorescence light detection; RIA = radioimmunoassay;**	**ELISA = Enzyme-linked Immunosorbent Assay; M/F = Male/Female .**

# **Table 4 Gallbladder cancer**

| **Reference** | **Population** | **Study period** | **Study design** | **N(Ca/Co)** | **Gender** | **Age (years)** | **Mycotoxin** | **Method** | **Matrix** | **LOD**  **(LOQ)**  **(µg/L)** | **Conc.**  **(µg/L)** | **OR/RR/CC/AR/HR**  **(95% CI)** | **Prevalence** | **RoB** |
| --- | --- | --- | --- | --- | --- | --- | --- | --- | --- | --- | --- | --- | --- | --- |
| *Gallbladder cancer with gallstones* | | | | | | | | | | | | | | |
| Koshiol, 2024^73^ | China | 1986 | NCC | 250(84/168) | M | 42-66 | AFB1-lysine | LC-MS/MS | Serum | n.r. | 0.02 – 0.07  0.134 | 1.8^OR^  (0.75 – 4.30)  2.2^OR^  (0.91 – 5.6) | n.r. | Def. Low |
| Yadav, 2025^74^ | India | 2022 | CaCo | 151(51/100) | M/F | 41-53 | AFB1-lysine | ELISA | Serum | n.r | <17.3   ≥ 17.3 | 10.6^OR^  (0.60 – 194)  36.6^OR^  (2.1 – 653) | n.r | Def. Low |
| Koshiol, 2017^75^ | China | 1997 | NCC | 459(209/250) | M/F | 37-74 | AFB1-lysine | LC-MS/MS | Plasma | 0.5 | 0.216 | 2.71^OR^  (1.70 –4.33) | Plasma:32% | Prob. Low |
| Nogueira, 2015^76^ | Chile | 2012 | CaCo | 65(36/29) | M/F | 41-77 | AFB1-lysine | HPLC-FLD | Plasma | 0.5 | 0.304 | 9.4^OR^  (2.80 –37.2) | n.r. | Def. Low |
| *Gallbladder cancer without gallstones* | | | | | | | | | | | | | | |
| Nogueira, 2015^76^ | Chile | 2012 | CaCo | 83(36/47) | M/F | 37-79 | AFB1-lysine | HPLC-FLD | Plasma | 0.5 | 0.304 | 13.2^OR^  (4.3 –47.9) | n.r. | Def. Low |

**Note: N = sample size; Ca = cases; Co = controls; LOD = limit of detection; LOQ = limit of quantification; Conc. = concentration; OR = odds ratio; RR = relative risk; CC = correlation coefficient; AR = attack rate; HR= hazard ratio; CI = confidence interval; RoB = Risk of Bias according to OHAT; Cs = cross-sectional study; CaCo = case-control study; NCC = nested case-control; n.r. = not reported; U(H)PLC-MS = ultra(high) performance liquid chromatography - mass spectrometry; FLD = fluorescence light detection ;**	**ELISA = Enzyme-linked Immunosorbent Assay; M/F = Male/Female.**

# **Table 5 Bladder cancer**

| **Reference** | **Population** | **Study period** | **Study design** | **N(Ca/Co)** | **Gender** | **Age (years)** | **Mycotoxin** | **Method** | **Matrix** | **LOD (LOQ)**  **(µg/L)** | **Conc.**  **(µg/L)** | **OR/RR/CC/AR/HR**  **(95% CI)** | **Prevalence** | **RoB** |
| --- | --- | --- | --- | --- | --- | --- | --- | --- | --- | --- | --- | --- | --- | --- |
| *Bladder cancer* | | | | | | | | | | | | | | |
| Grosso, 2003^77^ | Tunisia | 1996 | CaCo | 83(21/62) | M/F | 32-84 | OTA | IAC-HPLC | Serum | 0.1 | 0.26 – 0.99 | n.r. | Serum:40.4% | Prob. high |
| Thé, 2015^78^ | Ivory Coast | 2005 | CaCo | 240(120/120) | M/F | 40-75 | OTA | HPLC-FLD | Serum | 0.05(0.2) | 0.07 – 8.06 | n.r. | Serum:76.67% | Prob. Low |
| Özçelik, 2001^79^ | Turkey | 2001 | CaCo | 55(15/40) | M/F | 56.8 | OTA | HPLC-FLD | Serum | 0.2 | 0.3 - 1.58 | n.r. | n.r. | Prob. High |
| Aslam, 2005^80^ | Pakistan | 2005 | CaCo | 127(96/31) | M/F | n.r. | OTA | HPLC-FLD | Whole blood | ≤0.03 | 0.03 – 3.41 | n.r. | n.r. | Prob. low |
| Aslam, 2012^81^ | Pakistan | 2012 | CaCo | 127(96/31) | M/F | 24 - 82 | OTA | HPLC-FLD | Plasma | 0.05 | 0.03 – 3.41 | n.r. | n.r. | Prob. High |

**Note: N = sample size; Ca = cases; Co = controls; LOD = limit of detection; LOQ = limit of quantification; Conc. = concentration; OR = odds ratio; RR = relative risk; CC = correlation coefficient; AR = attack rate; HR= hazard ratio; CI = confidence interval; RoB = Risk of Bias according to OHAT; Cs = cross-sectional study; CaCo = case-control study; NCC = nested case-control; n.r. = not reported; U(H)PLC-MS = ultra(high) performance liquid chromatography - mass spectrometry; FLD = fluorescence light detection ; ELISA = Enzyme-linked Immunosorbent; M/F = Male/Female.**

# **Table 6 Kidney disease**

| **Reference** | **Population** | **Study period** | **Study design** | **N(Ca/Co)** | **Gender** | **Age (years)** | **Mycotoxin** | **Method** | **Matrix** | **LOD**  **(LOQ)**  **(µg/L)** | **Conc.**  **(µg/L)** | **OR/RR/CC/AR/HR**  **(95% CI or p val)** | **Prevalence** | **RoB** |
| --- | --- | --- | --- | --- | --- | --- | --- | --- | --- | --- | --- | --- | --- | --- |
| *Renal cell carcinoma* | | | | | | | | | | | | | | |
| Fahmy, 2014^82^ | USA | 2014 | Coh | 52 | n.r. | n.r. | OTA | ELISA | Tissue; Plasma | n.r. | 0.004 – 0.25 | n.r. | n.r. | Def. Low |
| Claeys, 2022^83^ | EPIC | 1992 | CaCo | 450112(911/449201) | M/F | 35-70 | Multiple (62) | Food  Questionnaire | n.r. | n.r. | n.r. | 0.81^HR^  (0.62 – 1.07) | n.r. | Prob. High |
| Malir, 2019^84^ | Czech Republic | 2015 | CaCo | 286(50/236) | M/F | 40-81 | OTA, CIT, DH-CIT | HPLC-FLD; LC-MS/MS | Plasma;  Urine | OTA^plasma^  40 (100)  OTA^urine^  1 (2)  CIT^plasma^  70 (150)  CIT^urine^  20 (50)  DHCIT^plasma^  150 (300)  DHCIT^urine^  50 (100) | 0.040 – 0.870  0.001 – 0.0278  0.021 – 0.182  0.002 – 0.087  0  0.002 – 0.160 | n.r. | 48%  62%  98%  90%  0%  100% | Prob. Low |
| *Nephrotic syndrome* | | | | | | | | | | | | | | |
| Oyelami, 1995^85^ | Nigeria | 1995 | Cs | 25 | M/F | 3 | AFM2 | HPLC-FLD | Tissue | n.r. | 0.0075 | n.r. | n.r. | Prob. High |
| Wafa, 1998^86^ | Egypt | 1998 | CaCo | 55(15/40) | M/F | 5 - 50 | OTA | HPLC-FLD | Serum;  Urine | n.r. | 0 – 10.5 (Serum)  0 – 8.19  (Urine) | n.r. | 53% | Prob. high |
| *Balkan endemic nephropathy* | | | | | | | | | | | | | | |
| Yordanova, 2010^87^ | Bulgaria | 2005 | CaCo | 40(18/22) | M/F | 35 - 79 | OTA | HPLC-FLD | Serum | 0.1 | 0.4 – 3.9 | n.r. | 100% | Def. High |
| *Chronic kidney disease* | | | | | | | | | | | | | | |
| Oyelami, 1995^88^ | Nigeria | 1995 | Cs | 3 | M/F | 3-14 | AFL, AFB1,  AFG1 | HPLC-FLD | Tissue | 0.005 | 0.028 (AFL)  13.04  (AFB1)  25.58 – 75.33  (AFG1) | n.r. | 50%  (AFL)  50%(AFB1)  100% (AFG1) | Prob.  High |
| Oyelami, 1995^85^ | Nigeria | 1995 | Cs | 4 | M/F | 3-14 | AFG2, AFG1, AFM1, AFL,  AFB1, AFM2 | HPLC-FLD | Tissue | 0.005 | 0.168 – 0.220  (AFG2)  0.376  (AFG1)  0.638  (AFM1)  5.13  (AFL)  0.790  (AFB1)  1.313  (AFM2) | n.r. | n.r. | Prob. High |
| Kareem, 2021^89^ | Iraq | 2021 | CaCo | 136(86/50) | M/F | 52-64 | AFB1 | HPLC-MS/MS | Serum | n.r. | 0.86-8.33 | n.r. | 100% | Def. Low |
| MohdRedzwan, 2014^90^ | Malaysia | 2014 | Cs | 71 | M/F | 23-57 | AFB1-lysine | HPLC-MS/MS | Serum | 0.05(0.17) | 0.045-0.754 | n.r. | n.r. | Prob. High |
| Schwerdt, 2001^91^ | Germany | 2001 | CaCo | 39(18/31) | n.r. | n.r. | OTA | HPLC | Serum | 0.2 | 351 | n.r. | n.r. | Prob. Low |
| Desalegn, 2011^92^ | Sri Lanka | 2009 | Cs | 31 | M/F | 9 - 65 | AFB1, OTA  FB1 | ELISA | Urine | 0.005 | 0.005 – 0.80 (AFB1)  0.005 – 0.360 (OTA)  0.035 – 0.13 (FB1) | n.r. | AFB1 (61.29%)  OTA  (93.50%)  FB1  (19.40%) | Prob. High |
| Lin, 2016^93^ | China | 2013 | Cs | 5493 | M/F | 58.76 | AFB1 | ELISA | Food | 0.01 | 0.012 – 1.461 | n.r. | 42% | Prob. Low |
| Al-Mhanaa, 2020^94^ | Iraq | 2019 | CaCo | 109(94/15) | M/F | 18 –70 | OTA | HPLC;  TLC | Serum | n.r. | 0.59 – 2.61 | n.r. | 31% | Prob. High |
| Özçelik, 2001^79^ | Turkey | 2001 | CaCo | 75(35/40) | M/F | 26.5 | OTA | HPLC-FLD | Serum | 0.2 | 0.6 – 5.5 | n.r. | n.r. | Prob. High |
| Oyelami, 1997^95^ | Nigeria | 1997 | CaR | 20 | M/F | 0.5 – 14 | AFM2; AFG1 | HPLC-FLD | Tissue | 0.01 | 0.458 – 0.610 (AFM2)  1.70  (AFG1) | n.r. | n.r. | Prob. Low |
| Kosicki, 2021^96^ | Polen | 2021 | CaCo | 104(88/16) | M/F | 23 - 85 | OTA | HPLC-FLD | Serum | 0.03(0.1) | 0.1 -2.78 | n.r. | n.r. | Prob. Low |
| *Renal stones* | | | | | | | | | | | | | | |
| Özçelik, 2001^79^ | Turkey | 2001 | CaCo | 75(35/40) | M/F | 26.5 | OTA | HPLC-FLD | Serum | 0.2 | 0.4 - 2.5 | n.r. | n.r. | Prob. High |
| *Urothelial cancer* | | | | | | | | | | | | | | |
| Wafa, 1998^86^ | Egypt | 1998 | CaCo | 55(15/40) | M/F | 38 - 70 | OTA | HPLC-FLD | Serum;  Urine;  Tissue | n.r. | 0 – 5.57  0 – 4.64  0 – 1.28 | n.r. | Serum:20%  Urine:6%  Tissue:21% | Prob. High |
| *Interstitial nephritis* | | | | | | | | | | | | | | |
| Schwerdt, 2001^91^ | Germany | 2001 | CaCo | 47(16/31) | n.r. | n.r. | OTA | HPLC | Serum | 0.2 | 558 | n.r. | 87%. | Prob. Low |
| *Nephropathy* | | | | | | | | | | | | | | |
| Hassen, 2004^97^ | Tunisia | 2004 | CaCo | 140(100/40) | M/F | n.r. | OTA | HPLC-FLD | Serum | n.r. | 0 – 17125 | n.r. | n.r. | Prob. Low |
| Karamkhani, 2020^98^ | Iran | 2020 | CaCo | 40(20/20) | M | 32.21 | AFB-lysine | HPLC-FLD | Serum | 22(75) | 160 000 | n.r. | n.r. | Prob. High |
| Zaied, 2011^99^ | Tunisia | 2007 | CaCo | 221(83/138) | M/F | 18 - 88 | OTA | HPLC | Serum | 0.05(0.15) | 1 - 65 | n.r. | 76% | Prob. Low |
| Sangare-Tigori, 2006^100^ | Ivory Coast | 2001 | CaCo | 102(39/63) | M/F | 21-60 | OTA | HPLC-FLD | Serum | n.r. | 0.167 – 2.42 | n.r. | 21% | Prob. Low |
| Abid, 2003^101^ | Tunisia | 1991 | CaCo | 1159(954/205) | n.r. | n.r. | OTA | HPLC-FLD | Serum;  Urine | n.r. | 0 – 62.8  (Serum)  50.4  (Urine) | n.r. | Serum:95%  Urine:93% | Prob. Low |
| Maaroufi, 1995^102^ | Tunisia | 1995 | CaCo | 721(581/140) | M/F | 30 - 67 | OTA | HPLC-FLD | Whole blood | 1 | 12 - 55 | n.r. | n.r. | Prob. High |
| Hmaissia Khalifa, 2008^103^ | Tunisia | 2008 | CaCo | 236(131/105) | M/F | 54.33 | OTA | HPLC-FLD | Serum | 0.125 | 0.12 – 6.1 | n.r. | 61% | Prob. Low |
| Hmaissia Khalifa, 2012^104^ | Tunisia | 2012 | CaCo | 68(24/44) | M/F | 59.33 | OTA | HPLC-FLD | Serum | n.r. | 0.12 – 3.8 | n.r. | 80% | Prob. High |
| Dinis, 2007^105^ | Portugal | 2002 | Coh | 95 | M/F | 66 | OTA | HPLC-FLD | Serum | (0.05) | 0.12 – 1.52 | n.r. | 100% | Prob. High |
| Hsieh, 2004^106^ | Taiwan | 2004 | Coh | 47 | M/F | 20-80 | OTA | HPLC-FLD | Urine | n.r. | n.r. | 0.303^CC^  (0.006) | n.r. | Prob. High |
| DíazdeLeón-Martínez, 2019^107^ | Mexico | 2017 | Cs | 34 | F | 18 - 50 | AFB1-lysine | HPLC-FLD | Serum | 0.0035(0.0047) | 0.0432 – 4.104 | 0.231^cc^  (0.203) | n.r. | Def. High |
| Maaroufi, 1995^108^ | Tunisia | 1995 | CaCo | 101(84/17) | n.r. | n.r. | OTA | HPLC-FLD | Plasma;  Food | n.r. | 0.7 – 1136 (plasma)  0.3 – 46830 (Food) | n.r. | 100% | Prob. High |
| Smpokou, 2019^109^ | Nicaragua | 2019 | NCC | 350(28/29) | M/F | 18 - 30 | OTA; CIT | LC-MS/MS | Urine | n.r. | 0.331 (OTA)  0.205  (CIT) | n.r. | n.r. | Prob. Low |
| Hassan, 2006^110^ | Egypt | 2006 | CaCo | 50(36/14) | M/F child  F mother | 0.45  27.11 | OTA | HPLC-FLD | Serum;  Mother milk | 0.001 | 1.26 – 4.28 (Serum)  1.89  (Milk) | n.r. | 72% | Prob. Low |
| *Mesangial proliferative glomerulonephritis* | | | | | | | | | | | | | | |
| Schwerdt, 2001^91^ | Germany | 2001 | CaCo | 44(13/31) | n.r. | n.r. | OTA | HPLC | Serum | 0.2 | 404 | n.r. | n.r. | Prob. Low |
| *Rapidly progressive glomerulonephritis* | | | | | | | | | | | | | | |
| Schwerdt, 2001^91^ | Germany | 2001 | CaCo | 39(8/31) | n.r. | n.r. | OTA | HPLC | Serum | 0.2 | 294.9 | n.r. | n.r. | Prob. Low |
| *Membranous glomerulonephritis* | | | | | | | | | | | | | | |
| Schwerdt, 2001^91^ | Germany | 2001 | CaCo | 41(10/31) | n.r. | n.r. | OTA | HPLC | Serum | 0.2 | 165.64 | n.r. | n.r. | Prob. Low |
| *Focal segmental glomerulosclerosis* | | | | | | | | | | | | | | |
| Schwerdt, 2001^91^ | Germany | 2001 | CaCo | 40(9/31) | n.r. | n.r. | OTA | HPLC | Serum | 0.2 | 177.76 | n.r. | n.r. | Prob. Low |
| *Chronic glomerulonephritis* | | | | | | | | | | | | | | |
| Schwerdt, 2001^91^ | Germany | 2001 | CaCo | 39(8/31) | n.r. | n.r. | OTA | HPLC | Serum | 0.2 | 525.2 | n.r. | n.r. | Prob. Low |
| *End stage renal disease* | | | | | | | | | | | | | | |
| Wafa, 1998^86^ | Egypt | 1998 | CaCo | 55(15/40) | M/F | 10 - 58 | OTA | HPLC-FLD | Serum;  Urine | n.r. | 0 – 3.75 (Serum)  0 – 6.70  (Urine) | n.r. | 26% | Prob. high |
| *Granulomatosis with polyangiitis* | | | | | | | | | | | | | | |
| Schwerdt, 2001^91^ | Germany | 2001 | CaCo | 47(16/31) | n.r. | n.r. | OTA | HPLC | Serum | 0.2 | 456.5 | n.r. | 87% | Prob. Low |

**Note: N = sample size; Ca = cases; Co = controls; LOD = limit of detection; LOQ = limit of quantification; Conc. = concentration; OR = odds ratio; RR = relative risk; CC = correlation coefficient; AR = attack rate; HR= hazard ratio; CI = confidence interval; RoB = Risk of Bias according to OHAT; Cs = cross-sectional study; CaCo = case-control study; NCC = nested case-control; Coh = cohort study; n.r. = not reported; U(H)PLC-MS = ultra(high) performance liquid chromatography - mass spectrometry; FLD = fluorescence light detection; ELISA = Enzyme-linked Immunosorbent Assay; EPIC = European Prospective Investigation Into Cancer and Nutrition (Spain, Italy, France, Greece, Germany, Netherlands, United Kingdom, Denmark, Sweden and Norway); M/F = Male/Female.**

# **Table 7 Immunomodulation**

| **Reference** | **Population** | **Study period** | **Study design** | **N(Ca/Co)** | **Gender** | **Age (years)** | **Mycotoxin** | **Method** | **Matrix** | **LOD (LOQ)**  **(µg/L)** | **Conc.**  **(µg/L)** | **OR/RR/CC/AR/HR**  **(95% CI or p val)** | **Prevalence** | **RoB** |
| --- | --- | --- | --- | --- | --- | --- | --- | --- | --- | --- | --- | --- | --- | --- |
| *Impaired thymic size* | | | | | | | | | | | | | | |
| Xu, 2021^111^ | Gambia | 2011 | Coh | 374 | M/F | 0-1.1 | AFB1 | ELISA | Serum | 0.003 | 1.02 | -0.052^CC^ (0.001) | n.r. | Def. Low |
| *T cell activation* | | | | | | | | | | | | | | |
| Wood, 2017^112^ | South-Africa | 2010 | CaCo | 84(58/26) | M/F | 0 – 0.23 | OTA | ELISA | Plasma | 0.08 | 0.13 – 0.25 | 0.36^CC^ (0.02) HLADR  0.58^CC^(0.002)  CXCL10 | n.r. | Prob. Low |
| Jiang, 2005^113^ | Ghana | 2005 | Cs | 64 | M/F | 19-86 | AFB1-lysine | RIA | Plasma | n.r. | 6.08 – 41.5 | -0.57^CC^ (0.001) CD3+CD69+  -0.33^CC^(0.032)  CD19+CD69+ | n.r. | Prob. High |
| Jolly, 2011^114^ | Ghana | 2011 | CaCo | 314(155/159) | M/F | 35 | AFB1-lysine | RIA | Plasma | n.r. | 0-0.16 | 2.84^OR^  (1.17-7.78) | n.r. | Prob. low |
| *Cell mediated immunity response* | | | | | | | | | | | | | | |
| Turner, 2003^115^ | Gambia | 1998 | Cs | 472 | M/F | 6-9 | AFB1-lysine | ELISA | Serum | 0.005 | 0.2-18.84 | n.r. | n.r. | Prob. Low |
| *Antibody response* | | | | | | | | | | | | | | |
| Turner, 2003^115^ | Gambia | 1998 | Cs | 472 | M/F | 6-9 | AFB1-lysine | ELISA | Serum | 0.005 | 0.2-18.84 | n.r. | n.r. | Prob. Low |

**Note: N = sample size; Ca = cases; Co = controls; LOD = limit of detection; LOQ = limit of quantification; Conc. = concentration; OR = odds ratio; RR = relative risk; CC = correlation coefficient; AR = attack rate; HR= hazard ratio; CI = confidence interval; RoB = Risk of Bias according to OHAT; Cs = cross-sectional study; CaCo = case-control study; NCC = nested case-control; Coh = cohort study; n.r. = not reported; RIA = radioimmunoassay; ELISA = Enzyme-linked Immunosorbent Assay; M/F = Male/Female.**

# **Table 8 Liver disease**

| **Reference** | **Population** | **Study period** | **Study design** | **N(Ca/Co)** | **Gender** | **Age (years)** | **Mycotoxin** | **Method** | **Matrix** | **LOD (LOQ)**  **(µg/L)** | **Conc.**  **(µg/L)** | **OR/RR/CC/AR/HR**  **(95% CI or p val)** | **Prevalence** | **RoB** |
| --- | --- | --- | --- | --- | --- | --- | --- | --- | --- | --- | --- | --- | --- | --- |
| *Hepatocellular carcinoma* | | | | | | | | | | | | | | |
| Huybrechts, 2024^116^ | EPIC | 1992 | NCC | 450,112(255/449,857) | M/F | 35 - 70 | DON;OTA;PAT;FB;ENN;TeA;CIT;DAS;NIV;MON;ZEN;AF;T2;HT2; Multiple-mycotoxins | Dietary questionnaires | Food | n.r. | n.r. | 1.90^HR^  (1.18 – 3.05) | n.r. | Def. Low |
| Stora, 1981^117^ | Czechoslovakia | 1981 | Cs | 15 | M/F | 30-80 | AFB1 | TLC-UV | Tissue | n.r. | 0.315 -5.46 | n.r. | 30%. | Def. High |
| El-Kafrawy, 2005^118^ | Egypt | 2000 | OuR | 41 | M/F | 17-69 | R249 TP53 | Direct sequencing | Tissue | n.r. | n.r. | n.r. | 20% | Prob. Low |
| Huţanaşu, 2011^119^ | Romania | 2011 | CaCo | 108(53/55) | M/F | n.r. | STC | HPLC-FLD | Serum;  Urine | n.r. | 2.02;  9.39 | 0.15^CC^  (0.0001)  0.15^CC^  (0.003) | 26% | Prob. Low |
| Onyemelukwe,1982^120^ | Nigeria | 1982 | Cs | 20 | M/F | 20-65 | AFB1, AFB2,  AFG1, AFG2 | VFM | Serum | n.r. | 9-331 | n.r. | 100% | Prob. Low |
| Lunn, 1997^121^ | Taiwan | 1984 | CaCo | 142(105/37) | M/F | 54 | R249 TP53 | Direct sequencing | Tissue | n.r. | n.r. | 1.4^OR^  (<0.05) | 13% | Prob. Low |
| Nogueira, 2009^122^ | Brazil | 1998 | CoH | 74 | M/F | 54 | R249 TP53 | PCR-RFLP | Tissue | n.r. | n.r. | 2.45^OR^  (<0.05) | 28% | Prob. High |
| Abedi-Ardekani, 2011^123^ | Iran | 2011 | OuR | 21 | M/F | 56 | R249 TP53 | Direct sequencing | Tissue | n.r. | n.r. | n.r. | 0% | Prob. Low |
| Jiang, 1998^124^ | China | 1998 | OuR | 65 | n.r. | n.r. | R249 TP53 | PCR-RFLP | Tissue | n.r. | n.r. | n.r. | 58% | Prob. High |
| Zhou, 1996^125^ | China | 1992 | Cs | 31 | n.r. | n.r. | AFB1 | HPLC | Tissue | n.r. | n.r. | n.r. | 68% | Prob. High |
| Yu, 1997^126^ | Taiwan | 1988 | NCC | 129(43/86)  93(30/63) | n.r. | 51 | AFM1;AFB1;AFG1;  AFP1;AFB1-lysine | HPLC-FLD,  ELISA | Urine;  Serum | 0.05 | 0.68 – 2.90 | 6^OR^  (1.2-29)  2^OR^  (0.7-5.8)  0.4^OR^  (0.1-2.6)  2^OR^  (0.5-8)  10^OR^  (0.007) | 97%;  40%;  9%;  97%;  56% | Def. Low |
| Yu, 1992^127^ | China | 1992 | Cs | 51 | n.r. | n.r. | AFB1,  AFM1 | HPLC-FLD,  ELISA | Urine,  Food | n.r. | 0.750 | 0.58^CC^  (<0.01) | n.r. | Prob. Low |
| El-Shanawani, 2006^128^ | Egypt | 2006 | CaCo | 45(30/15) | M/F | 57 | AFB1; RP249 TP53 | ELISA | Serum | n.r. | 1.3 – 4.6 | 0.37^CC^  (0.041) | 80% | Prob. Low |
| DeCarvalho, 2013^129^ | Brazil | 1997 | OuR | 41 | M/F | 35-85 | R249 TP53 | Sequencing | Plasma;  Tissue | n.r. | n.r. | n.r. | 15% | Prob. Low |
| Kimbi, 2005^130^ | South-Africa | 2005 | Cs | 158 | M/F | 15-78 | RP249 TP53 | PCR | Serum | n.r. | n.r. | 13.3^OR^  (1.8 -100) | 18% | Prob. Low |
| Hollstein, 1993^131^ | Thailand | 1987 | OuR | 15 | M/F | 17-73 | AFB1-lysine;  RP249 TP53 | ELISA;  Direct  Sequencing | Serum;  Tissue | 0.005 | n.r. | n.r. | 50% | Prob. High |
| Hoque, 1999^132^ | USA | 1999 | Cs | 5 | M/F | n.r. | AFB1;  RP249 TP53 | ELISA;  Whole Genome Sequencing | Serum;  Tissue | n.r. | n.r. | n.r. | n.r. | Prob. Low |
| Bessard, 1990^133^ | Cote d'ivoire | 1990 | CaR | 1;  78 | n.r. | n.r. | AFB1; AFB2;  AFG1; AFG2 | LC-MS/MS | Tissue;  Food | n.r. | n.r. | n.r. | 100%;  36% | Def. High |
| Buetow, 1992^134^ | USA;  Alaska;  South-Africa;  Japan;  China | 1992 | OuR | 22;7;8;1;57 | n.r. | 14-79 | R249 TP53 | PCR | Tissue | n.r. | n.r. | n.r. | 1% | Prob. Low |
| Shirabe, 2011^135^ | Japan | 2004 | OuR | 279 | M/F | 17-91 | R249 TP53 | Sequencing | Tissue | n.r. | n.r. | n.r. | 1% | Prob. Low |
| Sun, 1999^136^ | China | 1987 | Coh | 145 | M | 25-60 | AFM1 | HPLC-FLD | Urine | 3.6 | 3.6-243 | 3.33^RR^  (0.017) | 55% | Prob. Low |
| Szymañska , 2009^137^ | China | 2009 | CaCo | 249(123/126) | M | 33-62 | RP249 TP53 | ELISA | Plasma | 0.005(0.01) | n.r. | 0.9^OR^  (0.52-1.56) | 60% | Def. Low |
| Lino-Silva, 2022^138^ | Mexico | 2005 | Cs | 100 | M/F | 40-59 | AFB1-lysine;RP249 TP53 | UPLC-MS/MS | Serum;  Tissue | 0.01 | n.r. | n.r. | n.r. | Prob. Low |
| Manda, 2018^139^ | Ivory-coast | 2018 | CaCo | 99(33/66) | M/F | 27-77 | AFB1-lysine | HPLC-FLD | Serum | n.r. | 15.63 - 92.74 | n.r. | 82% | Prob. High |
| Yeh, 2010^140^ | USA | 2001 | OuR | 18 | M/F | 45-68 | RP249 TP53 | Sequencing | Tissue | n.r. | n.r. | n.r. | n.r. | Prob. Low |
| Long, 2008^141^ | China | 2004 | OuR | 501 | M/F | 18-86 | RP249 TP53 | Sequencing | Tissue | n.r. | n.r. | n.r. | 59% | Def. Low |
| Shimizu, 1999^142^ | China | 1999 | OuR | 31 | M/F | 35-67 | RP249 TP53 | Sequencing | Tissue | n.r. | n.r. | n.r. | 32% | Prob. High |
| Wu, 2022^143^ | USA | 2008 | Cs | 155 | M/F | 61.5 | AFB1 | ELISA | Serum | 4.5 | 0.19 | n.r. | 46% | Prob. Low |
| Hosny, 2008^144^ | Egypt | 2005 | CaCo | 255(76/179) | M/F | 27-70 | R249 TP53 | WGS | Serum;  Tissue | n.r. | n.r. | n.r. | 10% | Prob. Low |
| Ross, 1992^145^ | China | 1986 | CaCo | 162(22/140) | M | 45-65 | AFM1, AFP1,  COMBI | HPLC-UV | Urine | n.r. | n.r. | 3^RR^(AFM1)  6.2^RR^(AFP1)  2.4^RR^(COMBI) | n.r. | Def. Low |
| Ibrahim, 2013^146^ | Egypt | 2010 | CaCo | 61(39/22) | M/F | 40-70 | OTA | HPLC | Serum | 0.005 | 0.13 – 10.9 | 9.78^OR^  (0.0002) | n.r. | Prob. Low |
| Bulatao-Jayme, 1982^147^ | Philippines | 1982 | CaCo | 165(76/89) | M/F | 47 | AFB1, AFM1 | TLC | Urine | n.r. | n.r. | 13.9^OR^ (<0.05) for <7µg/day  17^OR^(<0.05) for >7µg/day | 51% | Prob. Low |
| Ramalho, 2018^148^ | Brazil | 2011 | CaCo | 18(10/8) | M/F | 48 - 63 | AFB1 | HPLC-MS/MS | Tissue | 0.004(0.01) | 0.05 – 0.07 | n.r. | 37.5% | Def. Low |
| Wray, 1980^149^ | USA | 1980 | CaR | 1 | F | 31 | AFB1 | HPLC | Serum | n.r. | 3.39 | n.r. | n.r. | Prob. Low |
| Olubuyide, 1992^150^ | Nigeria | 1987 | Cs | 89 | M/F | 15 –78 | AFB1 | HPLC-FLD | Serum | n.r. | 0.017 | n.r. | 23% | Prob. Low |
| Srivatanakul, 1991^151^ | Thailand | 1991 | CaCo | 99(46/53) | M/F | 15-74 | AFB1-lysine | ELISA | Serum | n.r. | 0.30 | 1^OR^ | 46% | Def. Low |
| Chen,1996^152^ | Taiwan | 1996 | Coh | 106(20/86) | M/F | 30-65 | AFB1-lysine | ELISA | Serum | 0.01 | n.r. | 5.5^OR^  (<0.05) | 65% | Prob. Low |
| Rahman El-Zayadi, 2001^153^ | Egypt | 1992 | CaCo | 320(200/120) | M/F | 8-88 | AFB1 | TLC | Serum | 10 | 225.6 | 2^RR^ | 17% | Prob. Low |
| Murugavel, 2007^154^ | India | 1994 | CaCo | 53(38/15)  68(38/30) | M/F | 18 - 61 | AFB1 | ELISA;  Immuno-  peroxide text | Serum;  Tissue | n.r. | n.r. | n.r. | 60% and 90% | Prob. Low |
| Asim, 2011^155^ | India | 2005 | CaCo | 517(266/251) | M/F | 45 | AFB1 | HPLC-UV | Urine | 0.04 | n.r. | 4.53^OR^ | 53% | Def. Low |
| Qian, 1994^156^ | China | 1986 | CaCo | 398(22/267) | n.r | n.r | AFB1, AFP1,  AFM1, AFQ1, AFG1 | HPLC-UV | Urine | 0.07 | 0.17-16 | 3.5^RR^(AFB1)  5.1^RR^(AFP1)  5.8^RR^(AFM1)  7.6^RR^(AFQ1)  3.8^RR^(AFG1) | 30%;28%;  36%;14%;  18% | Prob. Low |
| Peers, 1973^157^ | Kenya | 1967 | OuR | 46 | M/F | 16 | AFB1 | n.r. | Plasma | n.r. | n.r. | n.r. | n.r. | Prob. High |
| Kirk, 2005^158^ | Gambia | 1997 | CaCo | 624(216/408) | M/F | 49 | R249 T953 | DNA  Sequencing | Plasma | n.r. | n.r. | 20.3^OR^  (>0.05) | 40% | Def. Low |
| Ozdemir, 2010^159^ | Turkey | 2003 | CaCo | 100(50/50) | M/F | 65 | R249 T953 | DNA  Sequencing | Plasma | n.r. | n.r. | n.r. | 2% | Prob. Low |
| McGlynn, 1995^160^ | China | 1991 | CaCo | 168(52/116) | M/F | 30-70 | R249 T953 | DNA  Sequencing | Tissue | n.r. | n.r. | n.r. | 20% | Prob. Low |
| Afum, 2016^161^ | Ghana | 2012 | CaCo | 174(38/136) | M/F | 37 | AFM1 | ELISA | Urine | 0.5 | n.r. | n.r. | n.r. | Prob. Low |
| Deng, 1998^162^ | China | 1998 | Cs | 62 | n.r. | n.r. | R249 T953 | DNA  Sequencing | Tissue | n.r. | n.r. | n.r. | 60% | Prob. Low |
| Bessard, 1990^133^ | Ivory Coast | 1990 | CaR | 1 | n.r. | n.r. | AFB1; AFB2;  AFG2 | HPLC | Tissue | n.r. | 0.1-11.8 | n.r. | n.r. | Def. High |
| Ng, 1994^163^ | China | 1994 | Cs | 31 | M/F | 38-72 | R249 T953 | DNA  Sequencing | Tissue | n.r. | n.r. | n.r. | 7.7% | Def. Low |
| Ozturk, 1991^164^ | Mozambique  Vietnam  China  South Africa  South Korea  Japan  USA  Germany  Spain  Turkey  Saudi Arabia  Israel  Italy  India | 1991 | Cs | 15  3  30  24  5  12  27  20  12  8  4  3  3  1 | M/F | 23-50 | R249 T953 | DNA  Sequencing | Tissue | n.r. | n.r. | n.r. | 8%  53%  33%  7%  4.17%  0%  0%  0%  0%  0%  0%  0%  0%  0% | Prob. High |
| Wu, 2009^165^ | Taiwan | 1991 | CaCo | 1282(230/1052)  1250(198/1052) | M/F | 30-64 | AFB1; AFB1-lysine | ELISA | Plasma;  Urine | n.r. | 0.47-1.3 | 1.54^OR^  (<0.01)  1.76^OR^  (<0.01) | n.r. | Def. Low |
| Zeng, 2017^166^ | China | 2013 | CaCo | 428(214/214) | M/F | 51 | AFB1-lysine | ELISA | Serum | n.r. | 5.8 | 1.9^OR^  (1.1 -3.4) | n.r. | Prob. Low |
| Peers, 1987^167^ | Swaziland | 1982 | Cs | 2583 | n.r. | n.r. | AFB1; AFB2;  AFG1; AFG2 | ELISA | Food | 1 | 0-420 | n.r. | 20% | Def. High |
| Lu, 1998^168^ | China | 1998 | CaCo | 180(30/150) | M/F | 29-66 | AFB1-lysine | ELISA | Serum | n.r. | 18 | 3.47^OR^  (0.01) | 80% | Prob. High |
| Tian, 1995^169^ | China | 1995 | CaCo | 30(20/10° | n.r. | n.r. | STC | ELISA | Plasma;  Urine | n.r. | 0.65-0.113 | n.r. | 30% | Prob. Low |
| Yu, 1992^127^ | China | 1987 | Cs | 81  81 | M/F | n.r. | AFM1;  AFB1 | ELISA | Urine;  Food | n.r. | n.r. | 0.0403^CC^  (0.0592)  0.58^CC^  (<0.01) | n.r. | Prob. Low |
| Chan, 1990^170^ | Costa Rica | 1979 | CaCo | 39(13/26) | n.r. | n.r. | AFB1 | n.r. | Corn | n.r. | n.r. | n.r. | n.r. | Def. High |
| Mizrak, 2009^171^ | Turkey | 2006 | CaCo | 265(203/62) | M/F | n.r. | AFB1; AFB2;  AFG1; AFG2 | HPLC-MS/MS | Plasma | n.r. | 0.04 | 3.7^OR^  (0.005) | 20% | Prob. Low |
| Ming, 2002^171^ | China | 1994 | Cs | 181 | M/F | 27-74 | AFM1 | HPLC-UV | Urine | n.r. | 0.008 -0.037 | n.r. | n.r. | Prob. Low |
| Wild, 1993^172^ | Gambia | 1991 | Coh | 117 | M/F | 3-4 | AFB1-lysine | ELISA | Serum | n.r. | 0.09 - 10 | 0.4^CC^  (0.001) | n.r. | Prob. Low |
| Persson, 2012^173^ | China | 1991 | CaCo | 551(271/280);  219(72/147) | M/F | 25-64  40-69 | FB1 | HPLC-MS/MS | Toenail | 0.025(0.06) | 0.375;  1.96 | 1.1^OR^  (0.64-1.9);  1.5^OR^  (0.7-3.07) | n.r. | Prob. Low |
| Lu, 2010^174^ | China | 1988 | Cs | 150 | M | 38 | AFM1 | ELISA | Urine | n.r. | 5.7- 243 | 2,23^RR^  (0,008) | 53% | Prob. Low |
| Liu, 2008^175^ | China | 2005 | Cs | BD290 | M/F | 18 - 37 | AFB1-lysine | ELISA | Plasma | 0.005 | 0.015-6.9 | n.r. | n.r. | Def. Low |
| Aydin, 2015^176^ | Turkey | 2013 | CaCo | 87(38/49) | M/F | 44 | AFB1 | HPLC-MS/MS | Serum | 0.02 | 0.063 | 1.49^OR^  (0.483) | 35% | Def. Low |
| Sun, 2001^177^ | Taiwan | 1991 | CaCo | 228(79/149) | M/F | 30-64 | AFB1-lysine | ELISA | Serum | 0.003 | n.r. | 2^OR^  (1.1 – 3.7) | 63% | Def. Low |
| DeVries, 1989^47^ | Kenya | 1989 | Coh | 5 | n.r. | n.r. | AFB1 | n.r. | Tissue;  Serum | n.r. | n.r. | n.r. | 60% | Prob. High |
| Wu, 2007^178^ | Taiwan | 1990 | CaCo | 950(174/776) | M/F | 30-64 | AFB1-lysine | ELISA | Serum | n.r. | n.r. | 1.7^OR^  (0.22) | n.r. | Def. Low |
| Stora, 1987^179^ | Czechia | 1981 | OuR | 38 | M/F | 38-88 | AFB1 | ELISA | Tissue | n.r. | 0.014-0.15 | n.r. | 80% | Def; Low |
| Chu, 2018^180^ | Taiwan | 1991 | CaCo | 3142(506/2636) | M/F | 30-65 | AFB1-lysine | ELISA | Serum | 7 | n.r. | 1^OR^  (0.54) | 80% | Def. Low |
| Tan, 1995^181^ | Singapore | 1991 | CaCo | 482(59/423) | M/F | 59 | AFB1; AFB2;  AFG1; AFG2 | HPLC-FLD | Whole blood;  Tissue | 0.003; 0.01 | 0-0.358 | n.r. | 10% | Prob. High |
| Abdel-Wahab,2008 | Egypt | 2006 | CaCo | 100(80/20) | M/F | 40-70 | AFB1 | ELISA | Serum | n.r. | 32.5 | 0.47^CC^  (0.015) | n.r. | Def. Low |
| Ilić, 2016^182^ | Serbia | 2016 | CaR | 1 | F | 37 | AFB1 | ELISA | Serum | n.r. | n.r. | n.r. | 100% | Prob. Low |
| El-Shahat, 2012^183^ | Egypt | 2012 | CaCo | 70(40/30) | M/F | 30-58 | AFM1 | HPLC | Serum | n.r. | 0.021 – 0.47 | n.r. | n.r. | Prob. High |
| Campbell, 1990^184^ | China | 1983 | Cs | 48 | M/F | 35-64 | AFB1 | ELISA | Urine | n.r. | n.r. | -0.17^CC^  (0.01) | n.r. | Def. High |
| Lleonart, 2005^185^ | Gambia | 2005 | CaCo | 220(89/131) | M/F | 19-85 | R249 TP53 | HPLC-MS/MS | Plasma | 10 copies | 2,800 copies | 15^OR^  (1.6-140) | 52% | Def. Low |
| Tchana, 2010^186^ | Cameroon | 1991 | Coh | 78(42/36) | M/F | 10-80 | AFB1 | HPLC-FLD | Serum | n.r. | 0.45 - 1.56 | n.r. | 60% | Prob. Low |
| Tomerak, 2011^187^ | Egypt | 2008 | Cs | 150 | F | 16-36 | AFM1 | ELISA | Breast milk | 0.05 | 0.2-19 | n.r. | n.r. | Prob. High |
| Ortiz-Cuaran, 2013^188^ | Thailand | 2008 | CaCo | 183(50/133) | M/F | 42-74 | R249 TP53 | HPLC-MS/MS | Serum | 149 copies | n.r. | n.r. | 56% | Def. Low |
| Soini,1996^189^ | Mexico | 1985 | OuR | 21 | M/F | 22-79 | AFB1-lysine | ELISA | Serum | n.r. | 0.54-4.6 | n.r. | 19% | Prob. Low |
| Mokhles, 2007^190^ | Egypt | 2004 | CaCo | 58(46/12) | M/F | 56 | AFM1 | HPLC-MS/MS | Serum;  Urine | 0.04 | 3.8;  5.6 | n.r. | n.r. | Prob. Low |
| Kirk, 2000^191^ | Gambia | 1997 | CaCo | 106(53/53) | M/F | 19-87 | R249 TP53 | Direct sequencing | Plasma | n.r. | n.r | 16.4^OR^  (0.4-26.1) | 36% | Def. Low |
| Lu, 2010^174^ | China | 1988 | Cs | 150 | M | 38 | AFM1 | ELISA | Urine | n.r. | 5.7 - 243 | 2.23^RR^  (0.008) | n.r. | Prob. Low |
| Chu, 2017^192^ | Taiwan | 1991 | NCC | 643(66/577) | M/F | 30-65 | AFB1 | ELISA | Serum | 0.02 | ≤0.39 | 5.39^OR^(0.0368) | n.r. | Def. Low |
| Chu, 2017^192^ | Taiwan | 1991 | NCC | 643(66/577) | M/F | 30-65 | AFB1 | ELISA | Serum | 0.02 | <0.39 | 2.38^OR^(0.3166) | n.r. | Def. Low |
| Mandishona, 1998^193^ | South-Africa | 1990 | CaCo | 24/48 | M/F | 11-80 | AFB1-lysine | ELISA | Serum | n.r. | n.r. | n.r. | n.r. | Def. Low |
| *Cirrhotic hepatocellular carcinoma* | | | | | | | | | | | | | | |
| Chu, 2017^192^ | Taiwan | 1991 | NCC | 691(114/577) | M/F | 30-65 | AFB1 | ELISA | Serum | 0.02 | ≥0.39 | 5.47^OR^(0.0003) | n.r. | Def. Low |
| Chu, 2017^192^ | Taiwan | 1991 | NCC | 691(114/577) | M/F | 30-65 | AFB1 | ELISA | Serum | 0.02 | ≥0.39 | 2.53^OR^(0.06) | n.r. | Def. Low |
| Ortiz-Cuaran, 2013^188^ | Thailand | 2008 | CaCo | 167(34/133) | M/F | 33-71 | R249 TP53 | HPLC-MS/MS | Serum | 149 copies | n.r. | n.r. | 80% | Def. Low |
| Ngindu, 1982^194^ | Kenya | 1981 | Coh | 20 | M/F | 2.5-45 | AFB1 | TLC | Tissue | n.r. | 39-89 | n.r. | n.r. | Prob. Low |
| López, 2002^195^ | Argentina | 2002 | CaCo | 45(20/35) | M/F | 42-64 | AFB1 | HPLC-FLD | Serum | n.r. | n.r. | n.r. | 1% | Def. Low |
| *Neonatal jaundice* | | | | | | | | | | | | | | |
| Ahmed, 1995^196^ | Nigeria | 1987 | CaCo | 78(38/40)  124(64/60) | n.r. | 0 – 0.07 | AFB1; AFB2; AFM1;  AFM2; AFL; AFG1. AFG2 | HPLC-MS/MS | Serum;  Cord Blood | n.r. | 0-10.3 | n.r. | 40%;  30% | Prob. Low |
| Raafat, 2021^197^ | Egypt | 2018 | Cs | 24 | M | 0–0.08 | AFB1 | HPLC-FLD | Serum | 0.003(0.009) | 4-20 | 5.9^OR^  (1.3 – 27) | 58% | Prob. Low |
| Sodeinde, 1995^198^ | Nigeria | 1995 | CaCo | 330(270/60) | M/F | 0-0.08 | AFB1; AFB2; AFM1;  AFM2; AFL; AFG1; AFG2 | HPLC | Plasma | n.r. | 0.005-165.07 | 2.68^OR^  (0.005) | 27.4%  aflatoxins | Def. Low |
| *Cholangiocarcinoma* | | | | | | | | | | | | | | |
| Parkin, 1991^199^ | Thailand | 1987 | CaCo | 206(106/103) | M/F | 28-75 | AFB1-lysine | ELISA | Serum | n.r. | n.r. | 1.4^OR^  (0.8-2.7) | n.r. | Def. Low |
| *Miscellaneous liver disease* | | | | | | | | | | | | | | |
| Coulter, 1986^200^ | Sudan | 1986 | Cs | 16 | n.r. | 1.6 | AFB1; AFB2; AFG1;  AFG2; AFM1; AFM2  AFL | HPLC-FLD | Urine;  Serum;  Tissue | n.r. | 0.67 | n.r. | n.r. | Def. Low |
| *AST impairment* | | | | | | | | | | | | | | |
| Abdel-Wahab, 2008^201^ | Egypt | 2006 | CaCo | 100(80/20) | M/F | 40-70 | AFB1 | ELISA | Serum | n.r. | 32.5 | -0.1^CC^  (0.36) | n.r. | Def. Low |
| Ankrah, 1994^202^ | Ghana | 1994 | Cs | 40 | M/F | 30-73 | AFB1; AFG1; AFQ1;  AFM1; COMBI | HPLC-MS/MS | Serum;  Urine;  Faeces | n.r. | 7.5 – 12.5 | n.r. | 30%;  10%;  20% | Prob. Low |
| Nasir, 2021^203^ | Pakistan | 2019 | Cs | 150 | M/F | 3-5 | AFM1 | ELISA | Urine | 0.001(0.03) | 0.13-2.5 | 0.28^CC^  (<0.01) | 10% | Prob. Low |
| Farag, 2018^204^ | Saudia Arabia | 2013 | CaCo | 50(38/12) | n.r. | n.r. | AFB1 | ELISA | Serum | (0.5) | 0.5 – 1.7 | 0.503^CC^  (0.001) | n.r. | Def. Low |
| Raafat, 2021^197^ | Egypt | 2018 | Cs | 24 | M | 0-0.08 | AFB1 | HPLC-FLD | Serum | 0.003(0.009) | 4-20 | -0.106^CC^  (>0.05) | 58% | Prob. Low |
| Liu, 2017^205^ | China | 2013 | Cs | 5493 | M/F | 30-94 | AFB1 | ELISA | Food | (0.01)^LLOQ^ | n.r. | 1.13^OR^  (0.56) | n.r | Prob. Low |
| Karamkhani, 2020^98^ | Iran | 2020 | CaCo | 40(20/20) | M | 32 | AFB1-lysine | HPLC-FLD | Serum | 22/75 | 228 | n.r. | n.r | Prob. High |
| Jolly, 2011^114^ | Ghana | 2011 | CaCo | 314(155/159) | M/F | 35 | AFB1-lysine | ELISA | Plasma | n.r | 0-0.1 | 1.55^OR^  (0.44) | n.r | Prob. Low |
| *ALT impairment* | | | | | | | | | | | | | | |
| Farag, 2018^204^ | Saudia Arabia | 2013 | CaCo | 50(38/12) | n.r. | n.r. | AFB1 | ELISA | Serum | (0.5) | 0.5 – 1.7 | 0.678^CC^  (0.001) | n.r. | Def. Low |
| Ankrah, 1994^202^ | Ghana | 1994 | Cs | 40 | M/F | 30-73 | AFB1; AFG1; AFQ1;  AFM1; COMBI | HPLC-MS/MS | Serum;  Urine;  Faeces | n.r. | 7.5 – 12.5 | n.r. | 30%;  10%;  20% | Prob. Low |
| Nasir, 2021^203^ | Pakistan | 2019 | Cs | 150 | M/F | 3-5 | AFM1 | ELISA | Urine | 0.001(0.03) | 0.13-2.5 | 0.21^CC^  (<0.01) | 10% | Prob. Low |
| Raafat, 2021^197^ | Egypt | 2018 | Cs | 24 | M | 0-0.08 | AFB1 | HPLC-FLD | Serum | 0.003(0.009) | 4-20 | 0.536^CC^  (<0.05) | 58% | Prob. Low |
| Liu, 2017^205^ | China | 2013 | Cs | 5493 | M/F | 30-94 | AFB1 | ELISA | Food | (0.01)^LLOQ^ | n.r. | 1.16^OR^  (0.6) | n.r | Prob. Low |
| Karamkhani, 2020^98^ | Iran | 2020 | CaCo | 40(20/20) | M | 32 | AFB1-lysine | HPLC-FLD | Serum | 22(75) | 240 | n.r. | n.r | Prob. High |
| Jolly, 2011^114^ | Ghana | 2011 | CaCo | 314(155/159) | M/F | 35 | AFB1-lysine | ELISA | Plasma | n.r | 0-0.1 | 0.72^OR^  (0.67) | n.r | Prob. Low |
| *Bilirubin impairment* | | | | | | | | | | | | | | |
| Abdel-Wahab, 2008^201^ | Egypt | 2006 | CaCo | 100(80/20) | M/F | 40-70 | AFB1 | ELISA | Serum | n.r. | 32.5 | -0.13^CC^  (0.26) | n.r. | Def. Low |
| Mohd Redzwan, 2014^90^ | Malaysia | 2014 | Cs | 71 | M/F | 23-57 | AFB1-lysine | HPLC-MS/MS | Serum | 0.05(0.17) | 0.07 – 1.14 | 0.35^CC^  (0.003) | n.r. | Prob. High |
| Nasir, 2021^203^ | Pakistan | 2019 | Cs | 150 | M/F | 3-5 | AFM1 | ELISA | Urine | 0.001(0.03) | 0.13-2.5 | 0.08^CC^  (0.32) | 10% | Prob. Low |
| Wu, 2022^143^ | USA | 2008 | Cs | 155 | M/F | 61.5 | AFB1 | ELISA | Serum | 4.5 | 0.19 | 0.32^CC^  (0.0001) | 46% | Prob. Low |
| Raafat, 2021^197^ | Egypt | 2018 | Cs | 24 | M | 0-0.08 | AFB1 | HPLC-FLD | Serum | 0.003(0.009) | 4-20 | 0.44^CC^  (>0.05) | 58% | Prob. Low |
| Jolly, 2011^114^ | Ghana | 2011 | CaCo | 314(155/159) | M/F | 35 | AFB1-lysine | ELISA | Plasma | n.r | 0-0.1 | 1.53^OR^  (0.81) | n.r | Prob. Low |
| *Liver cirrhosis* | | | | | | | | | | | | | | |
| DeCarvalho, 2013^129^ | Brazil | 1997 | OuR | 74 | M/F | 16-81 | R249 TP53 | Sequencing | Plasma;  Tissue | n.r. | n.r. | n.r. | 15% | Prob. Low |
| Amla, 1971^206^ | India | 1971 | OuR | 20 | M/F | 1.5-5 | AFB1 | TLC | Urine | n.r. | n.r. | n.r. | 7% | Prob. Low |
| El-Shahat, 2012^183^ | Egypt | 2012 | CaCo | 70(40/30) | M/F | 30-58 | AFM1 | HPLC | Serum | n.r. | 0.021 – 0.47 | n.r. | n.r. | Prob. High |
| Kirk, 2000^191^ | Gambia | 1997 | CaCo | 66(13/53) | M/F | 19-87 | R249 TP53 | Direct sequencing | Plasma | n.r. | n.r | 3.2^OR^  (0.4-26.1) | 15% | Def. Low |
| Alvarez, 2020^207^ | Guatemala | 2015 | CaCo | 300(100/200) | M/F | 47-64 | AFB1-lysine | IDMS | Serum | n.r. | 0.03 - 10 | 11.5^OR^  (4.05 – 32.9) | n.r. | Def. Low |
| Ramalho, 2018^148^ | Brazil | 2011 | CaCo | 18(8/10) | M/F | 48-63 | AFB | UPLC-MS/MS | Tissue | 0.004(0.10) | 0.01-0.42 | n.r. | 35% | Def. Low |
| Kirk, 2005^158^ | Gambia | 1997 | CaCo | 624(216/408) | M/F | 49 | R249 T953 | DNA  Sequencing | Plasma | n.r. | n.r. | 4.83^OR^  (>0.05) | 20% | Def. Low |
| Ozdemir, 2010^159^ | Turkey | 2003 | CaCo | 100(50/50) | M/F | 61 | R249 T953 | DNA  Sequencing | Plasma | n.r. | n.r. | n.r. | 0% | Prob. Low |
| Chen, 2007^208^ | Taiwan | 2002 | CaCo | C314(85/229) | M/F | 67 | AFB1-lysine | ELISA | Serum | 2.4 | 0 – 3.3 | 2.29^OR^  (0.009) | 85% | Prob. Low |
| Chu, 2017^192^ | Taiwan | 1991 | NCC | 691(114/577) | M/F | 30-65 | AFB1 | ELISA | Serum | 0.02 | ≥0.39 | 2.45^OR^(0.0003) | n.r. | Def. Low |
| Chu, 2017^192^ | Taiwan | 1991 | NCC | 691(114/577) | M/F | 30-65 | AFB1 | ELISA | Serum | 0.02 | <0.39 | 1.47^OR^(0.15) | n.r. | Def. Low |
| Mizrak, 2009^171^ | Turkey | 2006 | CaCo | 265(203/62) | M/F | n.r. | AFB1; AFB2;  AFG1; AFG2 | HPLC-MS/MS | Plasma | n.r. | 0.02 - 0.09 | 6.6^OR^  (0.004) | 20% | Prob. Low |
| Aydin, 2015^176^ | Turkey | 2013 | CaCo | 87(38/49) | M/F | 44 | AFB1 | HPLC-MS/MS | Serum | 0.02 | 0.063 | 1.02^OR^  (0.971) | 30% | Def. Low |
| Huţanaşu, 2011^119^ | Romenia | 2011 | CaCo | 113(58/55) | M/F | n.r. | STC | HPLC-FLD | Serum;  Urine | n.r. | 0.626;  1.05 | 0.48^CC^  (0.003)  0.48^CC^  (0.049) | 26% | Prob. Low |
| Lleonart, 2005^185^ | Gambia | 2005 | CaCo | 173(42/131) | M/F | 21-71 | R249 TP53 | HPLC-MS/MS | Plasma | 10 copies | 500 copies | n.r. | 52% | Def. Low |
| Yadgiri, 1970^209^ | India | 1970 | CaCo | 16(11/6) | n.r. | n.r. | AFB1 | Spectro-photometry | Urine | n.r. | n.r. | n.r. | 60% | Prob. Low |
| Mokhles, 2007^190^ | Egypt | 2004 | CaCo | 24(12/12) | M/F | 48 | AFM1 | HPLC-MS/MS | Serum;  Urine | 0.04 | 19.2;  43.2 | n.r. | n.r. | Prob. Low |
| Anitha, 2014^210^ | India | 2009 | CaCo | 238(130/108) | M/F | 36-61 | AFB1-lysine | ELISA | Serum | n.r. | 0.15-41 | n.r. | 22% | Prob. Low |
| El-Shanawani, 2006^128^ | Egypt | 2006 | CaCo | 40(25/15) | M/F | 51 | AFB1; RP249 TP53 | ELISA | Serum | n.r. | 1.3 – 4.6 | 0.37^CC^  (0.041) | 80% | Prob. Low |
| *Chronic Hepatomegaly* | | | | | | | | | | | | | | |
| Gong, 2012^211^ | Kenya | 2002 | CaCo | 218(104/114) | M/F | 6-17 | AFB1-lysine | ELISA | Plasma | 0.18 | 8 - 41 | 1.43^OR^  (0.03) | n.r. | Prob. Low |
| *Fatty liver* | | | | | | | | | | | | | | |
| Sayed, 2005^212^ | Egypt | 2005 | Cs | 84 | M/F | 5-73 | AFB1 | ELISA | Serum | 0.1 | 0-0.16 | 0.2^OR^  (0.04-0.9) | 95% | Def. High |
| *Bilharzial liver* | | | | | | | | | | | | | | |
| Sayed, 2005^212^ | Egypt | 2005 | Cs | 84 | M/F | 5-73 | AFB1 | ELISA | Serum | 0.1 | 0-0.16 | n.r. | 95% | Def. High |

**Note: N = sample size; Ca = cases; Co = controls; LOD = limit of detection; LOQ = limit of quantification; Conc. = concentration; OR = odds ratio; RR = relative risk; CC = correlation coefficient; AR = attack rate; HR= hazard ratio; CI = confidence interval; RoB = Risk of Bias according to OHAT; Cs = cross-sectional study; CaCo = case-control study; NCC = nested case-control; Coh = cohort study; CaR = case report; n.r. = not reported; TLC = thin layered chromatography; U(H)PLC-MS = ultra(high) performance liquid chromatography - mass spectrometry; ELISA = Enzyme-linked immunosorbent assay; IDMS = Isotope Dilution Mass Spectrometry; FLD = fluorescence light detection; WGS = whole genome sequencing; PCR = polymerase chain reaction; = European Prospective Investigation Into Cancer and Nutrition (Spain, Italy, France, Greece, Germany, Netherlands, United Kingdom, Denmark, Sweden and Norway); VFM = Velasco fluorotoxin meter ; M/F : Male / Female .**

**Table 9 Neurologic disease**

| **Reference** | **Population** | | **Study period** | | **Study design** | | **N(Ca/Co)** | | **Gender** | | **Age (years)** | | **Mycotoxin** | | **Method** | | | | **Matrix** | | | **LOD (LOQ)**  **(µg/L)** | | | **Conc.**  **(µg/L)** | | | **OR/RR/CC/AR**  **(95% CI)** | | | **Prevalence**  **(%)** | | **RoB** |
| --- | --- | --- | --- | --- | --- | --- | --- | --- | --- | --- | --- | --- | --- | --- | --- | --- | --- | --- | --- | --- | --- | --- | --- | --- | --- | --- | --- | --- | --- | --- | --- | --- | --- |
| *Autism* | | | | | | | | | | | | | | | | | | | | | | | | | | | | | | | | | |
| De Santis, 2017^213^ | Italy | | 2017 | | CaCo | | 233(172/61) | | M/F | | 2-12 | | AFB1, AFM1, DON, DOM-1, FB1, GLIO, ZEN, OTA | | UPLC-MS/MS | | | | Urine;  Serum | | | (2)^urine^  (5)^serum^ | | | 0-83^urine^  0-28^serum^ | | | n.r. | | | 32-70^urine^  5-85^serum^ | | Prob. Low |
| Duringer, 2016^214^ | USA | | 2016 | | CaCo | | 54(25/29) | | M/F | | 12^mean^ | | ZEN, ZEN-4-glucoside, 3-ADON, ALT | | LC-MS/MS | | | | Urine | | | (50) | | | n.r. | | | n.r. | | | 0-12 | | Prob. Low |
| *Chronic Idiopathic Spastic Paraparesis* | | | | | | | | | | | | | | | | | | | | | | | | | | | | | | | | | |
| León, 1998^215^ | Colombia | | 1998 | | CaCo | | 40(15/25) | | M/F | | 34-85 | | AFB1, AFB2, AFG1, AFG2, OTA, NIV, DON, FB1 | | HPLC-MS/MS | | | | Urine | | | n.r. | | | 0-19,300 | | | n.r. | | | 0-87 | | Prob. Low |
| *Cognitive score* | | | | | | | | | | | | | | | | | | | | | | | | | | | | | | | | | |
| Mahfuz, 2022^216^ | Bangladesh | | 2022 | | Coh | | 163-194 | | M/F | | 1-3 | | AFB1-lysin | | HPLC-MS/MS | | | | Plasma | | | 0.0005 | | | n.r. | | | -0.7^CC^ (-1 to 0) | | | 17-61 | | Def. Low |
| *Expressive language score* | | | | | | | | | | | | | | | | | | | | | | | | | | | | | | | | | |
| Mahfuz, 2022^216^ | Bangladesh | | 2022 | | Coh | | 163-194 | | M/F | | 1-3 | | AFB1-lysin | | HPLC-MS/MS | | | | Plasma | | | 0.0005 | | | n.r. | | | -1^CC^ (-2 to 0) | | | 17-61 | | Def. Low |
| *Functionality of brain* | | | | | | | | | | | | | | | | | | | | | | | | | | | | | | | | | |
| Simon, 2003^217^ | n.r. | | 2003 | | CaCo | | 36(30/6) | | M/F | | 5-67 | | n.r. | | TH-SPECT | | | | Tissue (brain) | | | n.r. | | | n.r. | | | n.r. | | | n.r. | | Prob. Low |
| *Miscellaneous disease (Meningitis)* | | | | | | | | | | | | | | | | | | | | | | | | | | | | | | | | | |
| Oyelami, 1997^95^ | Nigeria | | 1997 | | OuR | | 20 | | M/F | 1-14 | | AFL, AFB1, AFB2, AFM1, AFM2, AFG1, AFG2 | | HPLC-FLD | | | | Tissue  (lung) | | | 0.005  0.01^AFB1,AFG1^ | | | 30 | | n.r. | | | n.r. | | | Prob. Low | |
| *Myalgic Encephalomyelitis, chronic fatigue syndrome* | | | | | | | | | | | | | | | | | | | | | | | | | | | | | | | | | |
| Wu, 2022^218^ | USA | | 2019-2021 | | Cs | | 236 | | M/F | 14-88 | | AFB1; OTA | | ELISA; LC-MS | | | | Urine | | | n.r. | | | 12; 1 | | n.r. | | | 30; 80 | | | Prob. Low | |
| Brewer, 2013^219^ | USA | | 2012 | | CaCo | | 167(102-55) | | M/F | 15-72 | | OTA, AFB1, MT, OTA+MT, AFB1+MT, COMBI | | ELISA | | | | Urine | | | 2 | | | 0-15 | | n.r. | | | 4-88 | | | Prob. Low | |
| *Neurodegenerative disease risk (Alzheimer or Parkinson)* | | | | | | | | | | | | | | | | | | | | | | | | | | | | | | | | | |
| Arce-López, 2021^220^ | Spain | | 2021 | | CaCo | | 93(68/25) | | M/F | 43-85 | | AFB1, AFB2, AFG1, AFG2, AFM1, T-2, HT-2, OTA, OTB, ZEN, STER, NIV, DON, 3-A-DON, 15-A-DON, DOM-1, NEO, DAS, FUS-X | | HPLC-MS/MS | | | | Plasma | | | 0-9 | | | 0-851 | | n.r. | | | n.r. | | | Prob. Low | |
| *Nodding syndrome* | | | | | | | | | | | | | | | | | | | | | | | | | | | | | | | | | |
| Duringer, 2021^221^ | Uganda | | 2014 | | CaCo | | 100(50/50) | | n.r. | 5-18 | | a-ZEL, T2, AFM1, ZEN, β-ZEL, HT2, FB1, FB2, OTA | | LC-MS/MS | | | | Urine | | | 0.1-25(1-50) | | | 0-317 | | n.r. | | | 12-70 | | | Prob. Low | |
| *Receptive language score* | | | | | | | | | | | | | | | | | | | | | | | | | | | | | | | | | |
| Mahfuz, 2022^216^ | Bangladesh | | 2022 | | Coh | | 163-194 | | M/F | 1-3 | | AFB1-lysin | | HPLC-MS/MS | | | | Plasma | | | 0.0005 | | | n.r. | | -1^CC^ (-2 to 0) | | | 17-61 | | | Def. Low | |
| *Reye's syndrome* | | | | | | | | | | | | | | | | | | | | | | | | | | | | | | | | | |
| Stora, 1983^222^ | | n.r. | | n.r. | | Cs | | 4 | M/F | 0.25-1.8 | | | AFB1 | | | TLC-UV | Tissue | | | n.r. | | | n.r. | | | | n.r. | | | 100 | | | Prob. High |
| Nelson, 1980^223^ | | Georgia, Tennessee and Alabama | | 1977 | | CaCo | | 65(17-48) | M/F | <1 - >14 | | | AFB1, AFM1 | | | n.r.;  HPLC + RIA | Serum;  Urine | | | n.r. | | | 0; 0-14 | | | | n.r. | | | n.r. | | | Prob. Low |
| Berry, 1986^224^ | | Tunesia | | 1986 | | Cs | | 9 | n.r. | n.r. | | | AFB1, AFB2, AFG1, AFG2, CIT, OTA, DON, DAN, FUS-X, DON-monoacetate, DAN | | | TLC | Food | | | n.r. | | | n.r. | | | | n.r. | | | n.r. | | | Prob. Low |
| Rogan, 1985^225^ | | USA | | 1982 | | CaCo | | 13(12/1) | M/F | 0-12 | | | AFB1, AFM1 | | | RIA | Tissue (liver) | | | 0.01 | | | n.r. | | | | n.r. | | | n.r. | | | Prob. Low |
| Becroft, 1972^226^ | | Auckland | | 1972 | | CaR | | 2 | M/F | 0-1 | | | AFB1 | | | HPLC-FLD | Tissue | | | n.r. | | | n.r. | | | | n.r. | | | n.r. | | | Prob. Low |
| Dementia | | | | | | | | | | | | | | | | | | | | | | | | | | | | | | | | | |
| Lefèvre-Arbogast, 2024^227^ | | France | | 1999 | | Coh | | 1,288 | M/F | 76 | | | MON, OTB | | | Food  questionnaires | Food | | | n.r. | | | n.r. | | | | n.r. | | | n.r. | | | Def. Low |

**Note: N = sample size; Ca = cases; Co = controls; LOD = limit of detection; LOQ = limit of quantification; Conc. = concentration; OR = odds ratio; RR = relative risk; CC = correlation coefficient; CI = confidence interval; AR = attack rate; RoB = Risk of Bias according to OHAT; Cs = cross-sectional study; Coh = cohort study; CaCo = case-control study; NCC = nested case-control; CaR = case report; n.r. = not reported; U(H)PLC-MS = ultra(high) performance liquid chromatography - mass spectrometry; FLD = fluorescence light detection; RIA = radioimmunoassay; TH-SPECT = triple-head single-photon emission computed tomography; ELISA = Enzyme-linked immunosorbent assay; TLC = thin layered chromatography;** **M/F = Male/Female.**

**Table 10 Metabolic disease**

| **Reference** | **Population** | **Study period** | **Study design** | **N(Ca/Co)** | **Gender** | | **Age (years)** | | **Mycotoxin** | | **Method** | | **Matrix** | | **LOD (LOQ)**  **(µg/L)** | | **Conc.**  **(µg/L)** | | **OR/RR/CC/AR**  **(95% CI)** | | **Prevalence (%)** | | **RoB** |
| --- | --- | --- | --- | --- | --- | --- | --- | --- | --- | --- | --- | --- | --- | --- | --- | --- | --- | --- | --- | --- | --- | --- | --- |
| *Diabetes Mellitus* | | | | | | | | | | | | | | | | | | | | | | | |
| Wu, 2022^143^ | USA | 2008-2014 | Cs | 155 | M/F | | ≥18-62 | | AFB1 | | ELISA | | Serum | | 5 | | 0.2 | | n.r. | | n.r. | | Prob. Low |
| Hamid Akash, 2021^228^ | Pakistan | 2021 | CaCo | 480(302/178) | M/F | | 15 - >55 | | AFM1 | | ELISA | | Serum;  Urine | | n.r. | | n.r.;  2 ± 0.3 | | 0.5(HbA1c)^CC^  0.4(insulin)^CC^  (0.4 to 0.7)  (0.3 to 0.6) | | n.r. | | Prob. Low |
| Alvarez, 2022^229^ | Guatemala | 2016 | Cs | 423 | M/F | | 40-60 | | AFB1-albumine | | IDMS | | Serum | | n.r. | | 0.4 | | 4^OR^  (2-8) | | n.r. | | Def. Low |
| Hsieh, 2004^106^ | Taiwan | 2004 | Coh | 7 | M/F | | 20-80 | | OTA | | HPLC-FLD | | Urine | | n.r. | | n.r. | | n.r. | | n.r. | | Prob. High |
| *Elevated AST/ALT* | | | | | | | | | | | | | | | | | | | | | | | |
| Alvarez, 2022^229^ | Guatemala | 2016 | Cs | 423 | M/F | | 40-60 | | AFB1-albumine | | IDMS | | Serum | | n.r. | | 0.3 | | 1^OR^  (0.5-2) | | n.r. | | Def. Low |
| *Elevated fatty liver index* | | | | | | | | | | | | | | | | | | | | | | | |
| Alvarez, 2022^229^ | Guatemala | 2016 | Cs | 423 | M/F | | 40-60 | | AFB1-albumine | | IDMS | | Serum | | n.r. | | 0.3 | | 0.7^OR^  (0-2) | | n.r. | | Def. Low |
| *Elevated hepatic steatosis index* | | | | | | | | | | | | | | | | | | | | | | | |
| Alvarez, 2022^229^ | Guatemala | 2016 | Cs | 423 | M/F | | 40-60 | | AFB1-albumine | | IDMS | | Serum | | n.r. | | 0.3 | | 1.4^OR^  (1-4) | | n.r. | | Def. Low |
| *Gestational diabetes* | | | | | | | | | | | | | | | | | | | | | | | |
| Xie, 2018^230^ | China | 2018 | CaCo | 6(3/3) | n.r. | | 31^mean^ | | AFB1 | | ELISA | | Cord blood | | n.r. | | 6±2 | | n.r. | | n.r. | | Prob. Low |
| *Metabolic syndrome* | | | | | | | | | | | | | | | | | | | | | | | |
| Alvarez, 2022^229^ | Guatemala | 2016 | Cs | 423 | M/F | 40-60 | | AFB1-albumine | | IDMS | | Serum | | n.r. | | 0.3 | | 1.7^OR^  (1-4) | | n.r. | | Def. Low | |
| Zeng, 2017^166^ | China | 2013 | CaCo | 5493(1747/3746) | M/F | 30-85 | | AFB1 | | ELISA | | Food | | n.r. | | n.r. | | 0.6^OR^  (0.5 - 0.7) | | n.r. | | Prob. Low | |

**Note: N = sample size; Ca = cases; Co = controls; LOD = limit of detection; LOQ = limit of quantification; Conc. = concentration; OR = odds ratio; RR = relative risk; CC = correlation coefficient; CI = confidence interval; AR = attack rate; RoB = Risk of Bias according to OHAT; OuR = outbreak report; Cs = cross-sectional study; Coh = cohort study; NCC = nested case-control; CaR = case report; n.r. = not reported; TLC = thin layered chromatography; U(H)PLC-MS = ultra(high) performance liquid chromatography - mass spectrometry; ELISA = Enzyme-linked immunosorbent assay; IDMS = Isotope Dilution Mass Spectrometry; M/F = Male/Female.**

**Table 11 Female infertility**

| **Reference** | **Population** | **Study period** | **Study design** | **N(Ca/Co)** | **Gender** | **Age (years)** | **Mycotoxin** | **Method** | **Matrix** | **LOD (LOQ)**  **(µg/L)** | **Conc.**  **(µg/L)** | **OR/RR/CC/AR**  **(95% CI)** | **Prevalence (%)** | **RoB** |
| --- | --- | --- | --- | --- | --- | --- | --- | --- | --- | --- | --- | --- | --- | --- |
| *Adiopathic anovulation* | | | | | | | | | | | | | | |
| El-Azab, 2009^231^ | Egypt | 2010 | CaCo | 27(7/20) | F | >18 | AFB1 | TLC-UV | Serum | n.r. | n.r. | n.r. | n.r. | Prob. Low |
| *Polycystic ovary syndrome* | | | | | | | | | | | | | | |
| El-Azab, 2009^231^ | Egypt | 2010 | CaCo | 63(43/20) | F | >18 | AFB1 | TLC-UV | Serum | n.r. | n.r. | n.r. | n.r. | Prob. Low |

**Note: N = sample size; Ca = cases; Co = controls; LOD = limit of detection; LOQ = limit of quantification; Conc. = concentration; OR = odds ratio; RR = relative risk; CC = correlation coefficient; CI = confidence interval; AR = attack rate; RoB = Risk of Bias according to OHAT; Cs = cross-sectional study; NCC = nested case-control; n.r. = not reported; TLC = thin layered chromatography; UV = Ultraviolet detection; F = Female.**

**Table 12 Premature puberty**

| **Reference** | **Population** | **Study period** | **Study design** | **N(Ca/Co)** | **Gender** | **Age (years)** | **Mycotoxin** | **Method** | **Matrix** | **LOD (LOQ)**  **(µg/L)** | **Conc.**  **(µg/L)** | **OR/RR/CC/AR**  **(95% CI)** | **Prevalence (%)** | **RoB** |
| --- | --- | --- | --- | --- | --- | --- | --- | --- | --- | --- | --- | --- | --- | --- |
| *Central ideopathic precocious puberty* | | | | | | | | | | | | | | |
| Asci, 2024^232^ | Turkey | 2024 | CaCo | 50(25/25) | F | 4-8 | ZEN | ELISA | Urine | 0.05 | 0-1 | n.r. | n.r. | Def. Low |
| *Central precocious puberty* | | | | | | | | | | | | | | |
| Massart, 2008^233^ | Italy | 2001-2005 | CaCo | 63(32/31) | F | n.r. | ZEN, α -ZEN, β -ZEN, α -ZAL, β -ZAL | HPLC-FLD | Serum | 0.025-0.25(0.05-0.5) | 0-1 | 0.9 (correlation with height) and 0.9(correlation with weight) ^CC^ | 19 | Prob. Low |
| *Early maternal menarche* | | | | | | | | | | | | | | |
| Deng, 2012^234^ | China | 2009-2010 | Cs | n.r. | n.r. | n.r. | ZEN | ELISA | Serum | n.r. | n.r. | 6^OR^ | n.r. | Def. Low |
| *Height z-score at menarche* | | | | | | | | | | | | | | |
| Rivera-Núñez, 2019^235^ | USA | 2006-2017 | Coh | 163 | F | 9-until event | ZEN, zearalanone, α-ZAL, α-ZEL, β-ZAL, β-ZEL | HPLC-MS/MS | Urine | 0.05 | 0-1 | n.r. | n.r. | Prob. Low |
| Idiopathic precocious puberty | | | | | | | | | | | | | | |
| Deng, 2012^234^ | China | 2009-2010 | CaCo | 178(78/100) | M/F | n.r. | ZEN | ELISA | Serum | n.r. | n.r. | 9^OR^ | n.r. | Def. Low |
| *Premature telarch* | | | | | | | | | | | | | | |
| Asci, 2024^232^ | Turkey | 2024 | CaCo | 52(28/25) | F | 4-8 | ZEN | ELISA | Urine | n.r. | 0-1 | n.r. | n.r. | Def. Low |
| *Time to menarche* | | | | | | | | | | | | | | |
| Rivera-Núñez, 2019^235^ | USA | 2006-2017 | Coh | 163 | F | 9-until event | ZEN, zearalanone, α-ZAL, α-ZEL, β-ZAL, β-ZEL | HPLC-MS/MS | Urine | 0.05 | 0-1 | n.r. | n.r. | Prob. Low |
| *Weight z-score at menarche* | | | | | | | | | | | | | | |
| Rivera-Núñez, 2019^235^ | USA | 2006-2017 | Coh | 163 | F | 9-until event | ZEN, zearalanone, α-ZAL, α-ZEL, β-ZAL, β-ZEL | HPLC-MS/MS | Urine | 0.05 | 0-1 | n.r. | n.r. | Prob. Low |

**Note: N = sample size; Ca = cases; Co = controls; LOD = limit of detection; LOQ = limit of quantification; Conc. = concentration; OR = odds ratio; RR = relative risk; CC = correlation coefficient; CI = confidence interval; AR = attack rate; RoB = Risk of Bias according to OHAT; Cs = cross-sectional study; CaCo = case-control study; NCC = nested case-control; Coh = cohort study; n.r. = not reported; U(H)PLC-MS = ultra(high) performance liquid chromatography - mass spectrometry; FLD = fluorescence light detection; ELISA = Enzyme-linked immunosorbent assay; UV = Ultraviolet detection; F = Female.**

**Table 13 Female reproductive disease (breast & endometrial cancer)**

| **Reference** | **Population** | **Study period** | **Study design** | **N(Ca/Co)** | **Gender** | **Age (years)** | **Mycotoxin** | **Method** | **Matrix** | **LOD(LOQ)**  **(µg/L)** | **Conc.**  **(µg/L)** | **OR/RR/CC/AR**  **(95% CI)** | **Prevalence (%)** | **RoB** |
| --- | --- | --- | --- | --- | --- | --- | --- | --- | --- | --- | --- | --- | --- | --- |
| *Begine ovarian cyst* | | | | | | | | | | | | | | |
| Gajecki, 2004^236^ | Poland | 2001-2002 | Cs | 74 | F | n.r. | ZEN | HPLC-UV | Plasma | n.r. | 87 | n.r. | 10 | Prob. High |
| *Benigne tumours* | | | | | | | | | | | | | | |
| Kuciel-Lisieska, 2008^237^ | Poland | 2004 | Cs | 200 | F | n.r. | ZEN | HPLC-FLD | Plasma | n.r. | 0-183 | n.r. | n.r. | Prob. Low |
| *Benigne uterus myoma* | | | | | | | | | | | | | | |
| Gajecki, 2004^236^ | Poland | 2001-2002 | Cs | 74 | F | n.r. | ZEN | HPLC-UV | Plasma | n.r. | 137 | n.r. | 10 | Prob. High |
| *Breast cancer* | | | | | | | | | | | | | | |
| Pillay, 2002^238^ | South Africa | 2002 | CaCo | 78(28/50) | F | n.r. | ZEN, α-ZEL, β-ZEL | HPLC, GC-MS | Plasma | 25-40 | 193-457 | n.r. | n.r. | Prob. Low |
| Belhassen, 2014^239^ | Tunisia | 2012 | CaCo | 110(69/41) | F | 29-79 | α-ZAL | UHPLC-MS/MS | Urine | 0.3(0-1) | 5 | 2^OR^  (1-3) | n.r. | Def. Low |
| *Endometrial cancer* | | | | | | | | | | | | | | |
| Unicsovics, 2024^240^ | Hungary | 2023 | NCC | 52(25/27) | F | n.r. | AFs, DON,  OTA, ZEN,  FB1, T2,  HT2 | ELISA | Serum;  Tissue (endometrial) | n.r. | 0.05 – 10.7  0.04 – 3.43 | n.r. | n.r. | Prob. Low |
| Gadzała-Kopciuch, 2011^241^ | Poland | 2011 | Coh | 7 | F | 66-76 | ZEN, α-ZEL | UE-MISPE-LC | Tissue (uterus) | 0.01(0.02-0.04) | 134-1,748 | n.r. | n.r. | Def. High |
| Gajecki, 2004^236^ | Poland | 2001-2002 | Cs | 74 | F | n.r. | ZEN | HPLC-UV | Plasma | n.r. | <5-26 | n.r. | 60 | Prob. High |
| *Malignant tumours* | | | | | | | | | | | | | | |
| Kuciel-Lisieska, 2008^237^ | Poland | 2004 | Cs | 200 | F | n.r. | ZEN | HPLC-FLD | Plasma | n.r. | 0-73 | n.r. | 37 | Prob. Low |
| *Ovarian cancer* | | | | | | | | | | | | | | |
| Gajecki, 2004^236^ | Poland | 2001-2002 | Cs | 74 | F | n.r. | α -ZEL | HPLC-UV | Plasma | n.r. | 5 | n.r. | 20 | Prob. High |

**Note: N = sample size; Ca = cases; Co = controls; LOD = limit of detection; LOQ = limit of quantification; Conc. = concentration; OR = odds ratio; RR = relative risk; CC = correlation coefficient; CI = confidence interval; AR = attack rate; RoB = Risk of Bias according to OHAT; Cs = cross-sectional study; Coh = cohort study; CaCo = case-control study; NCC = nested case-control; n.r. = not reported; TLC = thin layered chromatography; U(H)PLC-MS = ultra(high) performance liquid chromatography - mass spectrometry; FLD = fluorescence light detection; UV = Ultraviolet detection; GC = Gas Chromatography; UE-MISPE-LC = Ultrasound Extraction - Molecularly Imprinted Solid-Phase Extraction - Liquid Chromatography ;**	**ELISA = Enzyme-linked Immunosorbent Assay; F = Female.**

**Table 14 Male reproductive disease**

| **Reference** | **Population** | **Study period** | **Study design** | **N(Ca/Co)** | **Gender** | **Age (years)** | **Mycotoxin** | **Method** | **Matrix** | **LOD (LOQ)**  **(µg/L)** | **Conc.**  **(µg/L)** | **OR/RR/CC/AR**  **(95% CI)** | **Prevalence (%)** | **RoB** |
| --- | --- | --- | --- | --- | --- | --- | --- | --- | --- | --- | --- | --- | --- | --- |
| *Prostatic carcinoma* | | | | | | | | | | | | | | |
| Fry, 2022^242^ | USA | 2022 | Coh | 12 | M | 64^Mean^ | OTA | FM | Tissue (prostate) | n.r. | n.r. | n.r. | 100 | Prob. Low |
| *Testicular disease* | | | | | | | | | | | | | | |
| Fahmy, 2014^82^ | n.r. | 2014 | Coh | 4 | n.r. | n.r. | OTA | ELISA;  IHC | Plasma;  Urine | n.r. | n.r. | n.r. | 0 | Def. Low |
| *Infertility* | | | | | | | | | | | | | | |
| Ibeh, 1994^243^ | Nigeria | 1994 | CaCo | 100(50/50) | M | n.r. | AFG1, AFM1  AFM2 | n.r. | Semen | n.r. | 0.0016 | n.r. | 24 | Prob. High |
| Ning, 2024^244^ | China | 2014 | CaCo | 184(89/95) | M | 21-49 | 67 biomarkers | UPLC-MS/MS | Plasma | 0.001-0.5^LOD^  0.002-1^LLOQ^ | 0.002^CIT^  1.07^OTA^ | n.r. | 100^OTA^  79^CIT^  77^ENNB^  68^BEAU^  45^CPA^ | Prob. Low |
| Yang, 2024^245^ | China | 2014 | Cs | 192 | M | 21-49 | 67 biomarkers | UPLC-MS/MS | Plasma | 0.001-0.5^LOD^  0.002-1^LLOQ^ | 0.106^BEA^  0.003^ENNB^  0.885^OTA^  0.214^CIT^ | 1.35^OR^  (1.12-1.62)  0.9^OR^  (>0.05)  1.1^OR^  (>0.05)  1.37^OR^  (1.10-1.70) | n.r. | Prob. Low |
| Okonofua, 2024^246^ | Nigeria | 2024 | CaCo | 290(154/136) | M | 18- 59 | ZEN;OTA;  DON | UPLC-MS/MS | Urine | 0.16-0.61^LOD^  0.48-1.48^LLOQ^ | 466.8^ZEN^  149.6^OTA^  544.4^DON^ | n.r. | n.r. | Prob. Low |

**Note: N = sample size; Ca = cases; Co = controls; LOD = limit of detection; LOQ = limit of quantification; Conc. = concentration; OR = odds ratio; RR = relative risk; CC = correlation coefficient; CI = confidence interval; AR = attack rate; RoB = Risk of Bias according to OHAT; Coh = cohort study; CaCo = case-control study; NCC = nested case-control; n.r. = not reported; TLC = thin layered chromatography; ELISA = Enzyme-linked immunosorbent assay; FM = Fluorescence Microscopy; IHC = immunohistochemy ; ELISA = Enzyme-linked Immunosorbent Assay; M = Male.**

**Table 15 Musculoskeletal disease**

| **Reference** | **Population** | **Study period** | **Study design** | **N(Ca/Co)** | **Gender** | **Age (years)** | **Mycotoxin** | **Method** | **Matrix** | **LOD (LOQ)**  **(µg/L)** | **Conc.**  **(µg/L)** | **OR/RR/CC/AR**  **(95% CI)** | **Prevalence**  **(%)** | **RoB** |
| --- | --- | --- | --- | --- | --- | --- | --- | --- | --- | --- | --- | --- | --- | --- |
| *Chronic osteochondral disease* | | | | | | | | | | | | | | |
| Ning, 2023^247^ | China | 2017-2019 | CaCo | 153(30/123) | M/F | 1-9 | HT2 | UPLC-HRMS | Urine | n.r. | n.r. | n.r. | n.r. | Prob. Low |
| Ning, 2023^247^ | China | 2017-2019 | CaCo | 58(39/19) | M/F | 8-61 | HT2 | UPLC-HRMS | Urine | n.r. | n.r. | n.r. | n.r. | Prob. Low |

**Note: N = sample size; Ca = cases; Co = controls; LOD = limit of detection; LOQ = limit of quantification; Conc. = concentration; OR = odds ratio; RR = relative risk; CC = correlation coefficient; CI = confidence interval; AR = attack rate; RoB = Risk of Bias according to OHAT; Cs = cross-sectional study; CaCo = case-control study; NCC = nested case-control; n.r. = not reported; TLC = thin layered chromatography; U(H)PLC-MS = ultra(high) performance liquid chromatography - mass spectrometry; HRMS = High-Resolution Mass Spectrometry; M/F = Male/Female.**

**Table 16 Mucous Fibrosis**

| **Reference** | **Population** | **Study period** | **Study design** | **N(Ca/Co)** | **Gender** | **Age (years)** | **Mycotoxin** | **Method** | **Matrix** | **LOD (LOQ)**  **(µg/L)** | **Conc.**  **(µg/L)** | **OR/RR/CC/AR**  **(95% CI)** | **Prevalence**  **(%)** | **RoB** |
| --- | --- | --- | --- | --- | --- | --- | --- | --- | --- | --- | --- | --- | --- | --- |
| *Oral submucous fibrosis* | | | | | | | | | | | | | | |
| Bhowate, 2021^248^ | India | 2021 | CaCo | 128(88/40) | M/F | 21-40 | AFB1 antibody titer | ELISA | Serum | n.r. | n.r. | 0.6^CC^ | n.r. | Prob. Low |

**Note: N = sample size; Ca = cases; Co = controls; LOD = limit of detection; LOQ = limit of quantification; Conc. = concentration; OR = odds ratio; RR = relative risk; CC = correlation coefficient; CI = confidence interval; AR = attack rate; RoB = Risk of Bias according to OHAT; CaCo = case-control study; NCC = nested case-control; Cs = cross-sectional study; ELISA = Enzyme-linked immunosorbent assay; M/F = Male/Female.**

**Table 17 Oesophageal disease**

| **Reference** | **Population** | **Study period** | **Study design** | **N(Ca/Co)** | **Gender** | **Age (years)** | **Mycotoxin** | **Method** | **Matrix** | **LOD (LOQ)**  **(µg/L)** | **Conc.**  **(µg/L)** | **OR/RR/CC/AR**  **(95% CI)** | **Prevalence**  **(%)** | **RoB** |
| --- | --- | --- | --- | --- | --- | --- | --- | --- | --- | --- | --- | --- | --- | --- |
| *Achalasia* | | | | | | | | | | | | | | |
| Lin, 2020^249^ | China | 2016 | CaCo | 105(75/30) | M/F | 14-81 | AFB1, AFB2, AFG1, AFG2 | ELISA | oesophageal content | n.r. | n.r. | n.r. | n.r. | Prob. Low |
| *Oesophageal cancer* | | | | | | | | | | | | | | |
| Niknejad, 2021^250^ | Iran | 2021 | CaCo | 27(17/10) | M/F | 20-92 | DON, NEO, T2, HT2, 3-ADON, 15-ADON, FUS-X, DAS, NIV, ZON, ZAN | GC-MS/MS | Urine | 0.1-4(0.3-8) | 10.2-22 | n.r. | n.r. | Def. High |
| Mulisa, 2025^251^ | Ethiopia | n.r. | CaCo | 332(166/166) | M/F | 18-105 | AFB2, CIT, CPA, DON, ENNB, NIV, OTA, TA, ZAN, α-ZEL | UPLC-MS/MS | Plasma | 0,018-5 (0,024-10) | <LOD-0,73 | TA: 1,88^OR^ (1.68-2.11)  COMBI: 2.54^OR^ (2.1-3.07) | n.r. | Prob. Low |
| Xue, 2019^252^ | China | 2019 | CaCo | 570(190/380) | M/F | n.r. | AFB1, FB1;  FB1  COMBI | HPLC-FLD | Serum;  Urine;  COMBI | 0.04(0.2) | 0.04-0.2;  2.8-13.5;  n.r. | 2.6^OR^;  3.7^OR^:  4-7.1^OR^ | n.r. | Def. Low |
| *Oesophageal precancerous lesions* | | | | | | | | | | | | | | |
| Wang, 2020^253^ | China | 2015-2017 | CaCo | 200(100/100) | M/F | 35-75 | AFB1-lysine;  AFB1 | ELISA | Serum;  Food (corn flour) | n.r. | 0.02-0.4;  n.r. | 1-25^OR^;  1-1.4^OR^ | 14-50;  31-35 | Def. Low |

**Note: N = sample size; Ca = cases; Co = controls; LOD = limit of detection; LOQ = limit of quantification; Conc. = concentration; OR = odds ratio; RR = relative risk; CC = correlation coefficient; CI = confidence interval; AR = attack rate; RoB = Risk of Bias according to OHAT; Cs = cross-sectional study; CaCo = case-control study; NCC = nested case-control; n.r. = not reported; U(H)PLC-MS = ultra(high) performance liquid chromatography - mass spectrometry; FLD = fluorescence light detection; ELISA = Enzyme-linked immunosorbent assay; M/F = Male/Female.**

**Table 18 Gastric disease**

| **Reference** | **Population** | **Study period** | **Study design** | **N(Ca/Co)** | **Gender** | **Age (years)** | **Mycotoxin** | **Method** | **Matrix** | **LOD (LOQ)**  **(µg/L)** | **Conc.**  **(µg/L)** | **OR/RR/CC/AR**  **(95% CI)** | **Prevalence**  **(%)** | **RoB** |
| --- | --- | --- | --- | --- | --- | --- | --- | --- | --- | --- | --- | --- | --- | --- |
| *Gastric cancer* | | | | | | | | | | | | | | |
| Liu, 2022^254^ | China | 2002-2018 | Coh | 186 | M/F | 20-75 | AFB1 | WGS | Tissue | n.r. | n.r. | n.r. | n.r. | Prob. Low |
| Eom, 2013^255^ | Korea | 2013 | CaCo | 954(477/477) | M/F | n.r. | AFB1 | ELISA | Food | n.r. | n.r. | 1-2^OR^  (1.4 - 2.6) | 39-61 | Def. Low |
| *Pyloric stenosis* | | | | | | | | | | | | | | |
| Oyelami, 1995^88^ | Nigeria | 1995 | Cs | 1 | M | 0.125 | AFB1, AFB2, AFG1, AFG2, AFL, AFM1, AFM4 | HPLC-FLD | Tissue  (brain) | n.r. | 0.5 | n.r. | n.r. | Prob. High |
| Oyelami, 1998^85^ | Nigeria | 1995 | Cs | 1 | M | 0.125 | AFB1, AFB2, AFG1, AFG2, AFM1 AFM2, AFL | HPLC-FLD | Tissue  (kidney) | 0.005-0.01 | n.r. | n.r. | n.r. | Prob. High |

**Note: N = sample size; Ca = cases; Co = controls; LOD = limit of detection; LOQ = limit of quantification; Conc. = concentration; OR = odds ratio; RR = relative risk; CC = correlation coefficient; CI = confidence interval; AR = attack rate; RoB = Risk of Bias according to OHAT; CaCo = case-control study; NCC = nested case-control; Cs = cross-sectional study; Coh = cohort study; n.r. = not reported; U(H)PLC-MS = ultra(high) performance liquid chromatography - mass spectrometry; FLD = fluorescence light detection; RIA = radioimmunoassay; TH-SPECT = triple-head single-photon emission computed tomography; ELISA = Enzyme-linked immunosorbent assay; WGS = Whole Genome Sequencing; M/F = Male/Female.**

**Table 19 Intestinal disease**

| **Reference** | **Population** | **Study period** | **Study design** | **N(Ca/Co)** | **Gender** | **Age (years)** | **Mycotoxin** | **Method** | **Matrix** | **LOD (LOQ)**  **(µg/L)** | **Conc.**  **(µg/L)** | **OR/RR/CC/AR**  **(95% CI)** | **Prevalence**  **(%)** | **RoB** |
| --- | --- | --- | --- | --- | --- | --- | --- | --- | --- | --- | --- | --- | --- | --- |
| *Celiac disease (Coeliac disease)* | | | | | | | | | | | | | | |
| Cirlini, 2016^256^ | Italy | 2012-2014 | CaCo | 105(55/50) | M/F | 18-70 | DON, DON-GlcA, DOM-1, ZEN, α-ZEL, β-ZEL, ZEN14-Sulf, ZEN14-GlcA, FB1 | UPLC-MS/MS | Urine | 0.003-0.3 (0.009-1.1) | 0-9 | n.r. | 25 ZEN,  9 DON-Glca,  7 DON,  9 ZEL,  0 FB1,  0 ZEN14Glca | Prob. Low |
| Brera, 2013^257^ | Italy | 2011-2013 | CaCo | 65(35/30) | F | n.r. | AFM1, ZEN, OTA | HPLC-FLD | Breast milk | 0.007 | 0-2 | n.r. | 44 AFM1,  14 ZEN,  0 OTA | Prob. High |
| Roy, 1992^258^ | n.r. | 1992 | CaCo | 2-23 | n.r. | n.r. | AFB1 | ELISA | Serum;  Urine | n.r. | 172,000,000 – 600,000,000 | n.r. | 100 | Prob. High |
| *Colorectal cancer risk* | | | | | | | | | | | | | | |
| Ouhibi, 2020^259^ | Tunisia | 2020 | CaCo | 100(50/50) | M/F | 25-80 | PAT, CIT | UPLC-MS/MS | Urine;  Plasma | 0.1-1.7;  0.04-1.1 | 0-1;  0.5-12; | n.r. | 25 PAT, 36-70 CIT | Def. Low |
| Lisieska-Żołnierczyk, 2024^260^ | n.r. | n.r. | CaCo | 33(16/17) | M/F | 65,1^mean^ | ZEN, α-ZEL, β-ZEL | LC-MS/MS | Plasma | n.r. | 214,61-289,14 | n.r. | 48 | Prob. Low |
| *Crohn's disease* | | | | | | | | | | | | | | |
| Roy, 1992^258^ | n.r. | 1992 | CaCo | 6/23 | n.r. | n.r. | AFB1 | ELISA | Serum;  Urine | n.r. | 0-152,000,000 | n.r. | 53 | Prob. High |
| *Hirschsprung's disease* | | | | | | | | | | | | | | |
| Oyelami, 1995^88^ | Nigeria | 1995 | Cs | 1 | M | 1.5 | AFB1, AFB2, AFG1, AFG2, AFL, AFM1, AFM7 | HPLC-FLD | Tissue (brain) | n.r. | 1.6 | n.r. | 81 | Prob. High |
| Oyelami, 1995^85^ | Nigeria | 1998 | Cs | 1 | M | 1.5 | AFB1, AFB2, AFG1, AFG2, AFM1 AFM2, AFL | HPLC-FLD | Tissue (kidney) | 0.005 – 0.01 | 1.3 | n.r. | 38 | Prob. High |
| *(Intestinal) enteropathy* | | | | | | | | | | | | | | |
| Chen, 2023^261^ | Tanzania | 2009-2011 | NCC | 196(48/148)  196(87/109) | M/F | 2 | AFB1-lys;  FB1 | LC/MS | Plasma;  Urine | 0.4;  0.01 | 0.01-1;  0-17 | n.r. | n.r. | Prob. Low |
| *Typhoid enteritis (miscellaneous disease)* | | | | | | | | | | | | | | |
| Oyelami, 1997^95^ | Nigeria | 1997 | OuR | 20 | M/F | 0.5-14 | AFL, AFB1, AFB2, AFM1, AFM2, AFG1, AFG2 | HPLC-FLD | Tissue (lung) | 0.005  0.01^AFB1,AFG1^ | 0.001-1.2 | n.r. | 78 | Prob. Low |
| *Ulcerative colitis* | | | | | | | | | | | | | | |
| Roy, 1992^258^ | n.r. | 1992 | CaCo | 2/23 | n.r. | n.r. | AFB1 | ELISA | Serum;  Urine | n.r. | 12,000,000-112,000,000 | n.r. | 50 | Prob. High |
| *Microbial changes* | | | | | | | | | | | | | | |
| Ayeni, 2024^262^ | Nigeria | 2019-2021 | Cs | 14 | M/F | 0-1,5 | AFB1, AFB2, AFM1, AME, BEA, CIT, DHC, DON, DOM, ENNA, ENNB, ENNB1, FB1, FB2, OTA, OTα, OTB, STG, ZEN, α-ZEL, β-ZEL | LC-MS/MS | Breast milk, faeces, urine | 0,0001-0,024^breast milk^  0,00003-0,0049^faeces^  0,005-0,7^urine^ | <LOQ-779^breast milk^  <LOQ-197^faeces^  <LOQ-2743^urine^ | n.r. | Urine; AFM1 20, FB1 40, CIT 13, DHC 20, DON 67, OTA 73  Plasma: BEA 87, OTA 74, AME 96  Breast milk: 87 OTA | Prob. High |
| Oesterle, 2024^263^ | Nigeria | 2019-2021 | Cs | 11 | M/F | 0-1 | FB1, NIV | HPLC-HRMS | Breast milk, infant stool | n.r. | 55,35-462,4^infant stool^ | n.r. | n.r. | Prob. Low |

**Note: N = sample size; Ca = cases; Co = controls; LOD = limit of detection; LOQ = limit of quantification; Conc. = concentration; OR = odds ratio; RR = relative risk; CC = correlation coefficient; CI = confidence interval; AR = attack rate; RoB = Risk of Bias according to OHAT; CaCo = case-control study; NCC = nested case-control; Cs = cross-sectional study; Coh = cohort study; CaR = case report; n.r. = not reported; U(H)PLC-MS = ultra(high) performance liquid chromatography - mass spectrometry; FLD = fluorescence light detection; ELISA = Enzyme-linked immunosorbent assay; HRMS = High-Resolution Mass Spectrometry; M/F = Male/Female.**

**Table 20 Wasting**

| **Reference** | **Population** | **Study period** | **Study design** | **N(Ca/Co)** | **Gender** | **Age (years)** | **Mycotoxin** | **Method** | **Matrix** | **LOD (LOQ)**  **(µg/L)** | **Conc.**  **(µg/L)** | **OR/RR/CC/AR**  **(95% CI)** | **Prevalence**  **(%)** | **RoB** |
| --- | --- | --- | --- | --- | --- | --- | --- | --- | --- | --- | --- | --- | --- | --- |
| *Wasting* | | | | | | | | | | | | | | |
| Ashraf, 2022^264^ | Pakistan | 2020 | Cs | 238 | M/F | 1-11 | AFB1-lysine | HPLC-FLD | Serum | 0.04 | 0.03 – 10.73 | 1.02^OR^  (0.99 -1.05) | 100 | Prob. Low |
| Alamu, 2020^265^ | Zambia | 2020 | Cs | 19 | M/F | 0.5-2 | AFB1 | HPLC+LC-MS/MS | Serum | 0.03 | 0.03-13 | 0.64^OR^  (<0.0001) | n.r. | Def. Low |
| Bastos-Moreira, 2025^266^ | Burkina Faso | 2024 | Ch | 255 | F | 24.26 | Multiple mycotoxins | UPLC-MS/MS | VAMS | 0.09  (0.17) | 0.09 – 3.61 | -0.02^Bèta-value^  (-0.28 – 0.4) | 38 | Prob. Low |
| Nasir, 2021^203^ | Pakistan | 2019 | Cs | 150 | M/F | 3-5 | AFM1 | ELISA | Urine | 0.001 (0.03) | 0.13-2.5 | -0.15^CC^  (0.07) | 10% | Prob. Low |
| Magoha, 2014^267^ | Tanzania | 2011-2012 | Cs | 143 | F | 28^mean^ | AFM1 | HPLC-FLD | Human milk | 0.005 | 0-0.6 | 0.02^bèta-value^  (-0.028; 0.068) | n.r. | Prob. Low |
| Matchado, 2023^268^ | Malawi | 2011-2015 | Cs | 241 | M/F | 0.5-2.5 | AFB1-lysine | IDMS | Plasma | 0.01  (0.005) | n.r. | -0.07^Bèta-value^  (-0.34 – 0.2) | n.r. | Def. Low |

**Note: N = sample size; Ca = cases; Co = controls; LOD = limit of detection; LOQ = limit of quantification; Conc. = concentration; OR = odds ratio; RR = relative risk; CC = correlation coefficient; CI = confidence interval; AR = attack rate; RoB = Risk of Bias according to OHAT; CaCo = case-control study; NCC = nested case-control; Cs = cross-sectional study; n.r. = not reported; U(H)PLC-MS = ultra(high) performance liquid chromatography - mass spectrometry; FLD = fluorescence light detection; ELISA = Enzyme-linked immunosorbent assay; IDMS = Isotope Dilution Mass Spectrometry; M/F = Male/Female.**

**Table 21 Underweight**

| **Reference** | **Population** | **Study period** | **Study design** | **N(Ca/Co)** | **Gender** | **Age (years)** | **Mycotoxin** | **Method** | **Matrix** | **LOD (LOQ)**  **(µg/L)** | **Conc.**  **(µg/L)** | **OR/RR/CC/AR**  **(95% CI)** | **Prevalence**  **(%)** | **RoB** |
| --- | --- | --- | --- | --- | --- | --- | --- | --- | --- | --- | --- | --- | --- | --- |
| *Underweight* | | | | | | | | | | | | | | |
| Ashraf, 2022^264^ | Pakistan | 2020 | Cs | 238 | M/F | 1-11 | AFB1-lysine | HPLC-FLD | Serum | 0.04 | 0.03 – 10.73 | 0.99  (0.98 – 1.00) | 100 | Prob. Low |
| Andrews-Trevino, 2022^269^ | Nepal | 2015-2016 | Coh | 699 | M/F | 1.5-2.2 | AFB1, OTA, FB1;  OTA, FB1, DON | HPLC-FLD | Serum;  Urine | 0.4-20;  10-40 | 0-5-295 | 1.1-1.2^OR^  0.9-1^OR^ | n.r. | Def. Low |
| Gong, 2002^270^ | Savannah | 2002 | CaCo | 460(129/331) | M/F | 0.8-5 | AFB1 | ELISA | Serum | 2 | n.r. | < 0^CC^ | n.r. | Prob. Low |
| Mahdavi, 2010^271^ | Iran | 2007 | Cs | 91 | F | 27^mean^ | AFM1 | ELISA | Breast milk | 5 | 0.005-0.008 | -0.13^B^  (-0.36 – 0.1) | 22 | Def. Low |
| Shouman, 2012^272^ | Egypt | 2012 | Cs | 23 | M/F | 0.09-4.5 | AFB1 | TLC + UV detection | Serum | n.r. | 30,565-84,930 | -0.2  (0.185) | 37 | Prob. Low |
| Alamu, 2020^265^ | Zambia | 2020 | Cs | 19 | M/F | 0.5-2 | AFB1 | HPLC+LC-MS/MS | Serum | 0.03 | 0.03-13 | 1.19^OR^  (0.79- 1.78) | n.r. | Def. Low |
| Nasir, 2021^203^ | Pakistan | 2019 | Cs | 150 | M/F | 3-5 | AFM1 | ELISA | Urine | 0.001 (0.03) | 0.13-2.5 | -0.05^CC^  (0.54) | 10% | Prob. Low |
| Magoha, 2014^267^ | Tanzania | 2011-2012 | Cs | 143 | F | 28^mean^ | AFM1 | HPLC-FLD | Breast  milk | 0.005 | 0-0.6 | -0.009 ^bèta-value^  (-0.016; -0.001) | n.r. | Prob. Low |
| Turner, 2007^273^ | Gambia | 1999-2000 | Cs | 119;  99;  118 | n.r. | n.r. | AFB1-albumin | ELISA | Maternal blood;  Cord blood;  Infant blood | 0.005 | 0.2-10;  0.2-3.6;  0.2- 1.2 | -0.355^Bèta-value^  (0.727 – 0.018) | 100;  49;  11 | Prob. Low |
| Matchado, 2023^268^ | Malawi | 2011-2015 | Cs | 241 | M/F | 0.5-2.5 | AFB1-lysine | IDMS | Plasma | 0.005(0.01) | n.r. | -0.15 ^Bèta-value^  (-0.43 – 0.12) | n.r. | Def. Low |
| Ramjee, 1992^274^ | South Arica | 1992 | CaCo | 51(16/35) | n.r. | 0.5-2 | AFB1, AFB2, AFG1, AFG2, AFM1 | TLC + HPLC-FD | Serum | 1-50(0.001-0.05) | 0.1-0.3 | n.r. | 0-25 | Prob. Low |
| Chen, 2018^275^ | Tanzania, Haydom | 2009-2011 | CaCo | 60(10/50);  94(70/24) | M/F | 2-3 | AFB1;  FB1 | UPLC-MS/MS + LC-MS;  LC-MS | Plasma;  Urine | 0.01(0.4) | 0.01-1;  <LOD – 17 | n.r. | 72;  80 | Def. Low |
| Lauer, 2020^276^ | Uganda | 2012-2013 | Coh | 403 | F | 25^mean^ | AFB1 | HPLC-FLD | Serum | 0.4 | 0-16 | n.r. | 98 | Prob. Low |
| Kamala, 2018^72^ | Tanzania | 2013-2014 | RCT | 249(128/121) | M/F | 4^mean^ | AFB1, AFB2, AFG1, AFG2;  FB1, FB2 | HPLC-FLD | Food (maize) | 0.01-0.5(47-53) | 3.5-292 | n.r. | n.r. | Prob. High |
| Njumbe Ediage, 2013^277^ | Cameroon | 2012 | Cs | 220 | M/F | 1.5-4.5 | OTA, AFM1, DON, FB1, ZEN, B-ZOL, A-ZOL | LC-MS/MS | Urine | 0.01-0.3 | 0-77 | n.r. | n.r. | Prob. Low |
| Wangia-Dixon, 2020^278^ | Kenya | 2018 | Cs | 746 | M/F | 6-12 | AFB1-lysine | HPLC-FLD | Serum | 0.4 | 0.03-21 | n.r. | n.r. | Def. Low |
| Watson, 2018^279^ | Gambia | 2011-2012 | Cs | 374 | M/F | 0-2 | Aflatoxin | HPLC-FLD | Plasma | 3 | n.r. | n.r. | n.r. | Prob. Low |
| Andrews-Trevino 2019^280^ | Nepal | 2015-2017 | Coh | 1621 | M/F | 0 | AFB1-lysine | HPLC-FLD | Serum | 0.4 | <0.4-6 | 1.1^OR^ | n.r. | Prob. High |
| Lauer, 2019^281^ | Uganda | 2017 | Cs | 220 | M/F | n.r. | AFB1-lysine | HPLC-FLD | Maternal serum | 0.2 | 0.03-4 | n.r. | n.r. | Prob. Low |
| Gichohi-Wainaina, 2023^282^ | Tanzania | 2023 | Cs | 165 | M/F | 0.5-2 | AFB1 | ELISA | Food | 1 | 1.2-1,693 | 1^OR^ | n.r. | Prob. High |

**Note: N = sample size; Ca = cases; Co = controls; LOD = limit of detection; LOQ = limit of quantification; Conc. = concentration; OR = odds ratio; RR = relative risk; CC = correlation coefficient; AR = attack rate; RoB = Risk of Bias according to OHAT; OuR = outbreak report; Cs = cross-sectional study; CaCo = case-control study; NCC = nested case-control; Coh = cohort study; CaR = case report; RCT = randomised controlled trial; n.r. = not reported; TLC = thin layered chromatography; U(H)PLC-MS = ultra(high) performance liquid chromatography - mass spectrometry; FLD = fluorescence light detection; RIA = radioimmunoassay; ELISA = Enzyme-linked immunosorbent assay; IDMS = Isotope Dilution Mass Spectrometry; UV = Ultraviolet detection; M/F = Male/Female.**

# **Table 22 Stunting**

| **Reference** | **Population** | **Study period** | **Study design** | **N(Ca/Co)** | **Gender** | **Age (years)** | **Mycotoxin** | **Method** | **Matrix** | **LOD (LOQ)**  **(µg/L)** | **Conc.**  **(µg/L)** | **OR/RR/CC/AR**  **(95% CI)** | **Prevalence**  **(%)** | **RoB** |  |
| --- | --- | --- | --- | --- | --- | --- | --- | --- | --- | --- | --- | --- | --- | --- | --- |
| *Stunting* | | | | | | | | | | | | | | | |
| Ashraf, 2022^264^ | Pakistan | 2020 | Cs | 238 | M/F | 1-11 | AFB1-lysine | HPLC-FLD | Serum | 0.04 | 0.03 – 10.73 | 0.99  (0.99 -1.01) | 100 | Prob. Low |  |
| Andrews-Trevino, 2022^269^ | Nepal | 2015-2016 | Coh | 699 | M/F | 2-2.2 | AFB1, OTA;  FB1, DON | HPLC-FLD | Serum:  Urine | 0.4-20;  10-40 | 0-44  0-5,295 | 1-1.3^OR^;  1-1.1^OR^ | n.r. | Def. Low |  |
| Gong, 2002^270^ | Savannah | 2002 | CaCo | 455 (148/307) | M/F | 0.8-5 | AFB1 | ELISA | Serum | 2 | n.r. | <0^CC^ | n.r. | Prob. Low |  |
| Mahdavi, 2010^271^ | Iran | 2007 | Cs | 91 | F | 27^mean^ | AFM1 | ELISA | Breast milk | 5 | 0.005-0.008 | n.r. | 22 | Def. Low |  |
| Leroy, 2018^283^ | Mexico | 2005-2006 | Coh | 347 | M/F | 1^mean^ | AFB1 | HPLC-FLD | Serum | 0.2 | 0.03 | n.r. | n.r. | Prob. Low |  |
| Alamu, 2020^265^ | Zambia | 2020 | Cs | 19 | M/F | 0.5-2 | AFB1 | HPLC+LC-MS/MS | Serum | 0.03 | 0.03-13 | 1.3^OR^  (0.91 -1.85) | n.r. | Def. Low |  |
| Shirima, 2015^284^ | Tanzania, Nyabula | 2015 | NCC | 56(40/16) | M/F | 0.5-1.2 | AFB1;  FB1 | ELISA;  HPLC-MS | Serum;  Urine | 3;  3-20 | 0.2-1;  0.2-0.6 | n.r. | 67-100;  96-100 | Prob. Low |  |
| Shirima, 2015^284^ | Tanzania,  Kigwa | 2015 | NCC | 37(17/20) | M/F | 0.5-1.2 | AFB1;  FB1 | ELISA;  HPLC-MS | Serum;  Urine | 3;  3-20 | 0.2-1;  0.2-0.6 | n.r. | 67-100;  96-100 | Prob. Low |  |
| Shirima, 2015^284^ | Tanzania,  Kikelelwa | 2015 | NCC | 55(22/33) | M/F | 0.5-1.2 | AFB1;  FB1 | ELISA;  HPLC-MS | Serum;  Urine | 3;  3-20 | 0.2-1;  0.2-0.6 | n.r. | 67-100;  96-100 | Prob. Low |  |
| Shouman, 2012^272^ | Egypt | 2012 | Cs | 23 | M/F | 0.09-4.5 | AFB1 | TLC + UV detection | Serum | n.r. | 30,565-84,930 | n.r. | 37 | Prob. Low |  |
| Chen, 2018^275^ | Tanzania, Haydom | 2009-2011 | CaCo | 60(41/19);  94(70/24) | M/F | 2-3 | AFB1;  FB1 | UPLC-MS/MS + LC-MS;  LC-MS | Plasma;  Urine | 0.4;  0.01 | 0.01-1;  <0.4 – 17 | n.r. | 72;  80 | Def. Low |  |
| Mahfuz, 2021^285^ | Bangladesh | 2010-2012 | Coh | 228 | M/F | 0.6-3 | AFB1-lysine | UPLC-MS/MS | Plasma | n.r. | 0-5 | 2.2^OR^ | n.r. | Def. Low |  |
| Njumbe Ediage, 2013^277^ | Cameroon | 2012 | Cs | 220 | M/F | 1.5-4.5 | OTA, AFM1, DON, FB1, ZEN, B-ZOL, A-ZOL | LC-MS/MS | Urine | 0.01-0.3 | 0-77 | n.r. | n.r. | Prob. Low |  |
| Huong, 2019^286^ | Vietnam | 2014 | CaCo | 119(60/59) | M/F | 1-5 | AFB1, FB1, OTA | ELISA | Food | 0.2-2 | n.r. | -3 - (-0.7)^CC^ | n.r. | Prob. High |  |
| Watson, 2018^279^ | Gambia | 2011-2012 | Cs | 374 | M/F | 0-2 | Aflatoxin | HPLC-FLD | Plasma | 3 | n.r. | n.r. | n.r. | Prob. Low |  |
| Voth-Gaeddert, 2018^287^ | Guatemala | 2016-2017 | Cs | 320 | M/F | 0.5-5 | AFB1, AFB2, AFG1, AFG2 | ELISA | Food | 2 | n.r. | n.r. | n.r. | Prob. High |  |
| Gong, 2004^288^ | Benin | 2001 | Coh | 200 | M/F | 1-2 | AFB-albumin | ELISA | Serum | 3 | 3.5 | n.r. | n.r. | Prob. Low |  |
| Wangia-Dixon 2020^278^ | Kenya | 2018 | Cs | 746 | M/F | 6-12 | AFB1-lysine | HPLC-FLD | Serum | 0.4 | 0.03-21 | n.r. | n.r. | Def. Low |  |
| Andrews-Trevino 2019^280^ | Nepal | 2015-2017 | Coh | 1621 | M/F | 0 | AFB1-lysine | HPLC-FLD | Serum | 0.4 | <0.4-6 | 1-1.1^OR^ | n.r. | Prob. High |  |
| Mitchell, 2017^289^ | Nepal | 2010-2012 | Coh | 85 | M/F | 1.6-3 | AFB1-lysine | IDMS | Plasma | 0.02 | 0.1-0.16 | n.r. | n.r. | Prob. Low |  |
| Nasir, 2021^203^ | Pakistan | 2019 | Cs | 150 | M/F | 3-5 | AFM1 | ELISA | Urine | 0.001(0.03) | 0.1-2.5 | 0.13^CC^  (0.11) | 11 | Prob. low |  |
| Memiş, 2020^290^ | Turkey | 2017-2018 | Cs | 79 | M/F | 15-120^days^ | OTA, AFM1, DON, ZEN | ELISA | Breast milk | 0.002 | 0-13 | 0.1-1^OR^ | n.r. | Def. Low |  |
| Magoha, 2014^267^ | Tanzania | 2011-2012 | Cs | 143 | F | 28^mean^ | AFM1 | HPLC-FLD | Breast milk | 0.005 | 0-0.6 | 0.02^bèta-value^  (-0.03 – 0.07) | n.r. | Prob. Low |  |
| Turner, 2007^273^ | Gambia | 1999-2000 | Cs | 119;  99;  118 | n.r. | n.r. | AFB1-albumin | ELISA | Maternal blood;  Cord blood;  Infant blood | 0.005 | 0.2-10;  0.2-3.6;  0.2- 1.2 | -0.558^bèta-value^  (-0.906 - -0.216) | 100;  49;  11 | Prob. Low |  |
| Tessema, 2021^291^ | Ethiopia | 2015-2016 | CaCo | 102(50/52) | M/F | 0.5-3 | AFB1-lysine, AFB1, AFB2, AFG1, AFG2, AFM1, FB1, FB2, FB3 | LC-MS/MS | Serum | n.r. | <LOD – 0.3 | -58 – 47^OR^ | n.r. | Prob. Low |  |
| Kimanya, 2010^292^ | Tanzania | 2006 | Cs | 215 | M/F | 0.5 | FB1, FB2, FB3 | n.r. | Food (maize) | n.r. | 13-2,305 | -1.4 – (-0.3) | 31-69 | Prob. High |  |
| Matchado, 2023^268^ | Malawi | 2011-2015 | Cs | 241 | M/F | 0.5-2.5 | AFB1-lysine | IDMS | Plasma | 0.01  0.005 | n.r. | -0.2 ^Bèta-value^  (-0.3 – 0.1) | n.r. | Def. Low |  |
| Andrews-Trevino, 2021^293^ | Nepal | 2015-2019 | Coh | 1675 | n.r. | 0.3-2 | AFB1-lysine, AFM1 | HPLC-FLD | Serum;  Breast milk | 0.0004 | 0.02-6;  0.04-316 | 1.2^OR^ | 75-94;  94 | Prob. Low |  |
| Hoffmann, 2018^294^ | Kenya | 2013-2016 | CaCo | 798(436/362)  524(279/245) | M/F | 0 | AFB1-lysine | HPLC-FLD | Serum | n.r. | 0.4-3 | n.r. | n.r. | Prob. Low |  |
| Lauer, 2019^281^ | Uganda | 2017 | Cs | 220 | M/F | n.r. | AFB1-lysine | HPLC-FLD | Maternal serum | 0.2 | 0.03-4 | n.r. | n.r. | Def. Low |  |
| Gichohi-Wainaina, 2023^282^ | Tanzania | 2023 | Cs | 165 | M/F | 0.5-2 | AFB1 | ELISA | Food | 1 | 1.2-1,693 | 1^OR^ | n.r. | Prob. High |  |

**Note: N = sample size; Ca = cases; Co = controls; LOD = limit of detection; LOQ = limit of quantification; Conc. = concentration; OR = odds ratio; RR = relative risk; CC = correlation coefficient; CI = confidence interval; AR = attack rate; RoB = Risk of Bias according to OHAT; Cs = cross-sectional study; CaCo = case-control study; NCC = nested case-control; Coh = cohort study; n.r. = not reported; TLC = thin layered chromatography; U(H)PLC-MS = ultra(high) performance liquid chromatography - mass spectrometry; FLD = fluorescence light detection; ELISA = Enzyme-linked immunosorbent assay; IDMS = Isotope Dilution Mass Spectrometry; UV = Ultraviolet detection; M/F = Male/Female.**

# **Table 23 Adverse birth outcomes**

| **Reference** | **Population** | **Study period** | **Study design** | **N(Ca/Co)** | **Gender** | **Age (years)** | **Mycotoxin** | **Method** | **Matrix** | **LOD (LOQ)**  **(µg/L)** | **Conc.**  **(µg/L)** | **OR/RR/CC/AR**  **(95% CI)** | **Prevalence**  **(%)** | **RoB** |
| --- | --- | --- | --- | --- | --- | --- | --- | --- | --- | --- | --- | --- | --- | --- |
| *Preterm birth* | | | | | | | | | | | | | | |
| Tesfamariam, 2022^295^ | Ethiopia | 2017-2020 | Cs | 483 | F | 15-49 | AFB1, AFB2, AFG1, AFG2, AFM1, AFB1-lysine, FB1, FB2, FB3, HFB1, 3-ADON, DON, DON-3G, FUS-X, NIV, DOM-1, NEO, DAS, HT-2, T-2, ZEN, ZAN, α-ZEL, β-ZEL, β-ZAL, OTA, OTα, AOH, AME, TeA, Enn B, STG, CIT | UPLC-MS/MS | Serum | 0.03-6 | <0.03-34 | n.r. | n.r. | Prob. Low |
| Andrews-Trevino 2019^280^ | Nepal | 2015-2017 | Coh | 1621 | M/F | 0 | AFB1-lysine | HPLC-FLD | Serum | 0.4 | <0.4-6 | 1^OR^  (0.75 – 2.27)_ | n.r. | Prob. High |
| Shuaib, 2010^296^ | Ghana | 2006 | Cs | 785 | F | 14-48 | AFB1-lysin | HPLC-FLD | Serum | 0.0005 | 0.02-11 | 1.3^OR^  (0.75-2.27) | n.r. | Def. Low |
| *Small for gestational age* | | | | | | | | | | | | | | |
| Tesfamariam, 2022^295^ | Ethiopia | 2017-2020 | Cs | 483 | F | 15-49 | AFB1, AFB2, AFG1, AFG2, AFM1, AFB1-lysine, FB1, FB2, FB3, HFB1, 3-ADON, DON, DON-3G, FUS-X, NIV, DOM-1, NEO, DAS, HT-2, T-2, ZEN, ZAN, α-ZEL, β-ZEL, β-ZAL, OTA, OTα, AOH, AME, TeA, Enn B, STG, CIT | UPLC-MS/MS | Serum | 0.03-6 | <0.03-34 | n.r. | n.r. | Prob. Low |
| Shuaib, 2010^296^ | Ghana | 2006 | Cs | 785 | F | 14-48 | AFB1-lysin | HPLC-FLD | Serum | 0.0005 | 0.02-11 | 0.6-2.1^OR^  (0.2-3.7) | n.r. | Def. Low |
| Andrews-Trevino, 2019^280^ | Nepal | 2015-2017 | Coh | 1621 | M/F | 0 | AFB1-lysine | HPLC-FLD | Serum | 0.4 | <0.4-6 | 1.13^OR^  (1 – 1.27) | n.r. | Prob. High |
| Kinkade, 2024^297^ | USA | 2024 | Coh | 286 | F | 29 | ZEN and derivates | LC-MS/MS | Urine | n.r. | n.r. | n.r. | n.r. | Prob. Low |
| Bastos-Moreira 2025^266^ | Burkina Faso | 2024 | Coh | 255 | M/F | 25 | AFB1, AFB2, AFG1, AFG2, AFM1, AFB1-lysine, FB1, FB2, FB3, HFB1, 3-ADON, DON, DON-3G, FUS-X, NIV, DOM-1, NEO, DAS, HT-2, T-2, ZEN, ZAN, α-ZEL, β-ZEL, β-ZAL, OTA, OTα, AOH, AME, TeA, Enn B, STG, CIT | LC-MS/MS | VAMS | 0.09^LOD^  0.17^LLOQ^ | n.r. | 6.63^B^  (-3.52 – 16.7) | n.r. | Prob. Low |
| Osoro, 2024^298^ | Kenya | 2017 | NCC | 375(125/250) | F | 29 | AFB1-lysine | LC-MS/MS | Maternal  Plasma | 0.02^LOD^ | 0.061-0.18 | 1.01^OR^  (0.59 – 1.73) | 38.7 | Def. Low |
| *Low birthweight* | | | | | | | | | | | | | | |
| Bastos-Moreira, 2025^266^ | Burkina Faso | 2024 | Coh | 305 | M/F | 30 | AFB1, AFB2, AFG1, AFG2, AFM1, AFB1-lysine, FB1, FB2, FB3, HFB1, 3-ADON, DON, DON-3G, FUS-X, NIV, DOM-1, NEO, DAS, HT-2, T-2, ZEN, ZAN, α-ZEL, β-ZEL, β-ZAL, OTA, OTα, AOH, AME, TeA, Enn B, STG, CIT | LC-MS/MS | VAMS | 0.09^LOD^  0.17^LLOQ^ | n.r. | 7.80^B^  (-3.33 – 18.9) | n.r. | Prob. Low |
| Tan, 2023^299^ | China | 2023 | Coh | 1,538 | F | 28 | DON and derivates | LC-MS/MS | Urine | 0.5 | 5.1-12.1 | 1.48^OR^  (1.02 – 2.15) | n.r. | Def. Low |
| Zhong, 2024^300^ | Chine | 2021 | Coh | 126 | F | 30.1 | AFB1-lysine | ELISA | Serum;  Cord blood | n.r. | 0.03 – 8.50  0.05 – 8.42 | n.r. | n.r. | Def. Low |
| Raafat, 2021^197^ | Egypt | 2018-2020 | Cs | 24 | M | 0-0.1 | AFB1 | HPLC-FLD | Serum | 0.003(0.01) | 4-20 | -0.60^CC^ | n.r. | Prob. Low |
| Abdulrazzaq, 2004^301^ | United Arab Emirates | 1995-1998 | CaCo | 166(43/123) | M/F | 0 | AFM1 | HPLC-FLD | Serum | 0.1 | 4.3 | 0.056^CC^  (0.001) | n.r. | Prob. Low |
| Abdulrazzaq,  2002^302^ | United Arab Emirates | 1995-1999 | Cs | 201 | M/F | 0 | AFB1,AFM1, AFM2 | HPLC-FLD | Cord blood | 0.5 | 0.027 – 4.06 | n.r. | 71 | Prob. Low |
| DeVries, 1989^303^ | Kenya | 1985 | Cs | 208;  101 | F;  M/F | n.r. | AFB1, AFB2, AFG1, AFM1, AFM2, AFL | HPLC-FLD | Maternal blood;  Cord blood | n.r. | 0.01-12;  0.01-7 | n.r. | n.r. | Prob. High |
| *Microcephaly* | | | | | | | | | | | | | | |
| Andrews-Trevino, 2022^269^ | Nepal | 2015-2016 | Coh | 699 | M/F | 1.5-2.2 | AFB1, OTA,  FB1, DON | HPLC-FLD | Serum;  Urine | 0-20;  10-40 | 0-44  0-5,295 | 0.8-1.1^OR^;  1-1.1^OR^ | n.r. | Prob. Low |
| Matchado, 2023^268^ | Malawi | 2011-2015 | Cs | 241 | M/F | 0.5-2.5 | AFB1-lysine | IDMS | Plasma | 0.005(0.01) | n.r. | -0.33 ^Bèta-value^  (-0.63, -0.05) | n.r. | Def. Low |
| Tesfamariam, 2022^304^ | Ethiopia | 2017-2020 | Cs | 492 | F | 26^mean^ | AFB1-lysine | HPLC-FLD | Serum | 0.03 | 0.04-4 | -0.1- (-1)^CC^ | n.r. | Prob. Low |

**Note: N = sample size; Ca = cases; Co = controls; LOD = limit of detection; LOQ = limit of quantification; Conc. = concentration; OR = odds ratio; RR = relative risk; CC = correlation coefficient; CI = confidence interval; AR = attack rate; RoB = Risk of Bias according to OHAT; Cs = cross-sectional study; CaCo = case-control study; NCC = nested case-control; Coh = cohort study; n.r. = not reported; U(H)PLC-MS = ultra(high) performance liquid chromatography - mass spectrometry; FLD = fluorescence light detection; IDMS = Isotope Dilution Mass Spectrometry; M/F = Male/Female.**

# **Table 24 Kwashiorkor**

| **Reference** | **Population** | **Study period** | **Study design** | **N(Ca/Co)** | **Gender** | **Age (years)** | **Mycotoxin** | **Method** | **Matrix** | **LOD (LOQ)**  **(µg/L)** | **Conc.**  **(µg/L)** | **OR/RR/CC/AR**  **(95% CI)** | **Prevalence**  **(%)** | **RoB** |
| --- | --- | --- | --- | --- | --- | --- | --- | --- | --- | --- | --- | --- | --- | --- |
| *Kwashiorkor* | | | | | | | | | | | | | | |
| Hendrickse, 1982^305^ | Sudan | 1981 | CaCo | 88(44/44) | M/F | n.r. | AFB1, AFL | HPLC-FLD | Serum;  Urine | n.r. | 0.7;  0.1 | n.r. | n.r. | Prob. Low |
| De Vries, 1990^306^ | Kenya | 1986 | Cs | 1 | F | n.r. | AFB1, AFB2, AFM1, AFM2, AFG1, AFG2, AFL | HPLC-FLD | Serum;  Urine;  Faeces | n.r. | n.r. | n.r. | n.r. | Prob. Low |
| Coulter, 1986^200^ | Sudan | 1986 | CaCo | 40(16/24) | n.r. | 1.6^mean^ | AFB1, AFB2, AFG1, AFG2, AFM1, AFM2, AFL | HPLC-FLD;  HPLC-FLD;  Light Microscopy | Urine;  Serum;  Tissue (liver) | n.r. | 0-0.5;  0-1;  0-35 | n.r. | n.r. | Prob. Low |
| Hatem, 2005^307^ | Egypt | 2005 | CaCo | 40(30/10) | M/F | 0.6-1.7 | AFB1, AFB2, AFG1, AFG2, AFM1, AFM2, AFB2a, AFG2a, R0, AFB3, AFGM1 | 2D-TLC | Serum;  Urine | n.r. | 0-0.3;  0-0.08 | n.r. | 47-80;  46-80 | Def. Low |
| Coulter, 1986^308^ | Sudan | 1981-1983 | CaCo | 241(138/103)  269(114/155) | n.r. | 0.3-3.4 | AFB1, AFB2, AFG1, AFG2, AFM1, AFM2, AFL | HPLC-FLD + 2D TLC | Serum;  Urine | 1-50;  5-25 | 0.2;  0.3 | n.r. | 7-13;  4-6 | Def. Low |
| Apeagyei, 1986^309^ | Ghana | 1986 | OuR | 22 | M/F | 0.4-4 | AFB1, AFL | 2D-TLC & HPLC-FLD | Tissue | n.r. | 0.01-4.6 | n.r. | 9-91 | Prob. High |
| Adhikari, 1994^310^ | South Africa | 1994 | Cs | 36 | M/F | 0.5-2 | AFB1 | HPLC-FLD | Serum | n.r. | n.r. | n.r. | 58 | Prob. Low |
| Oyelami, 1995^88^ | Nigeria | 1995 | Cs | 18 | M/F | 0.5-3 | AFB1, AFG1, AFL, AFM1, AFM2, AFB2, AFG2 | HPLC-FLD | Tissue (brain) | n.r. | 0.02-113 | n.r. | 5-39 | Prob. Low |
| De Vries, 1987^311^ | Kenya | 1984-1985 | CaCo | 24(14/10) | n.r. | 4.5^mean^ | AFB1 | HPLC-FLD | Serum;  Urine | n.r. | 0.02-67;  0.04-1.4 | n.r. | 42-64 | Prob. high |
| Ramjee, 1992^274^ | South Arica | 1992 | CaCo | 80(45/35);  59(24/35) | n.r. | 0.5-2 | AFB1, AFB2, AFG1, AFG2, AFM1 | TLC + HPLC-FD | Serum;  Urine | 0.001-0.05 (1-50); 0.005-0.03(5-25) | 0.05-2.2;  n.r. | n.r. | 6-27;  0-19 | Prob. Low |
| Househam, 1991^312^ | South-Africa | 1991 | CaCo | 367(47/320) | M/F | 1-5 | AFB1, AFB2, AFG1, AFG2, AFL | HPLC-FLD | Urine | 20 | n.r. | n.r. | 0 | Prob. Low |
| Oyelami, 1995^85^ | Nigeria | 1995 | Cs | 24 | M/F | 0.5-6 | AFL, AB2, AG2, AM2, AFG1 | HPLC-FLD | Tissue (kidney) | 0.005-0.01 | 0.006-45 | n.r. | 8-29 | Prob. High |
| Lamplugh, 1982^313^ | Nigeria and South-Africa | 1982 | Coh | 3 | n.r. | n.r. | AFB1, AFL, AFM1 | HPLC-MS/MS | Tissue (liver) | n.r. | n.r. | n.r. | n.r. | Def. High |
| Tchana, 2010^186^ | Cameroon | 1991-1995 | CaCo | 78(42/36) | M/F | 1-12 | AFB1 | HPLC-FLD | Urine | n.r. | 0.1-3 | n.r. | 36 | Prob. Low |
| McMillan, 2018^314^ | Nigeria | 2012 | CaCo | 37(26/11) | n.r. | 0.5-4 | AFB1-lysine | LC-MS/MS | Plasma | 0.01-1.1 | n.r. | n.r. | n.r. | Prob. Low |
| Hendrickse, 1983^315^ | Nigeria and South-Africa | 1983 | Cs | 8 | n.r. | n.r. | AFB1, AFL, AFM1, AF | HPLC-FLD | Tissue (liver) | n.r. | 0.2-9 | n.r. | 0-88 | Prob. High |
| Hendrickse 1984^316^ | Sudan | 1984 | Cs | 468;  468;  469 | M/F | n.r. | AFB2, AFG1, AFG2, AFM1, AFM2, AFB1, AFL;  AFG1, AFG2, AFB2, AFM1, AFM2, AFB1, AFL;  AFB1,AFB2, AFM1, AFM2, AFL | HPLC-FLD | Faeces;  Urine;  Serum | n.r. | 0.15;  0.04-7.6;  0.003-3.4 | n.r. | 12 | Prob. Low |
| Oyelami, 1997^95^ | Nigeria | 1997 | OuR | 20 | M/F | 0.3-6 | AFB1, AFB2, AFM1, AFM2, AFG1, AFG2, AFL | HPLC-FLD | Tissue (lung) | 0.005  0.01^AFB1,AFG1^ | 0-55 | n.r. | 90 | Prob. Low |
| Okoth, 2004^317^ | Kenya | 2000-2002 | Cs | 242 | M/F |  | AFB1, AFB2, AFG1, AFG2 | TLC | Food | n.r | 10-82 | n.r. | 2-17 | Def. High |
| *Marasmus* | | | | | | | | | | | | | | |
| Hendrickse, 1982^305^ | Sudan | 1981 | CaCo | 101(57/44) | M/F | n.r. | AFB1, AFL | HPLC-FLD | Serum;  Urine | n.r. | 0.08;  0.2 | n.r. | n.r. | Prob. Low |
| Coulter, 1986^200^ | Sudan | 1986 | CaCo | 40(11/29) | n.r. | 1.7^mean^ | AFB1, AFB2, AFG1, AFG2, AFM1, AFM2, AFL | HPLC-FLD;  HPLC-FLD;  Light Microscopy | Urine;  Serum;  Tissue (liver) | n.r. | 0-0.8;  0;  0 | n.r. | n.r. | Prob. Low |
| Hatem, 2005^307^ | Egypt | 2005 | CaCo | 40(30/10) | M/F | 0.5-1 | AFB1, AFB2, AFG1, AFG2, AFM1, AFM2, AFB2a, AFG2a, R0, AFB3, AFGM1 | 2D-TLC | Serum;  Urine | n.r. | 0-0.1;  0-0.08 | n.r. | 47-80;  46-80 | Def. Low |
| Coulter, 1986^308^ | Sudan | 1981-1983 | CaCo | 221(118/103)  274(119/155) | n.r. | 0.6-4 | AFB1, AFB2, AFG1, AFG2, AFM1, AFM2, AFL | HPLC-FLD + 2D TLC | Serum;  Urine | 1-50;  5-25 | 0.08;  0.4 | n.r. | 0.8-7;  0.8-11 | Def. Low |
| Oyelami, 1995^88^ | Nigeria | 1995 | Cs | 1 | M | 0.3-0.3 | Total of aflatoxins | HPLC-FLD | Tissue (brain) | n.r. | 0 | n.r. | n.r. | Prob. Low |
| Househam, 1991^312^ | South Africa | 1991 | CaCo | 320(64/256) | M/F | 1-5 | AFB1, AFB2, AFG1, AFL | HPLC-FLD | Urine | n.r. | n.r. | n.r. | n.r. | Prob. Low |
| De Vries, 1987^311^ | Kenya | 1984-1985 | CaCo | 21(11/10) | n.r. | 5^mean^ | AFB1 | HPLC-FLD | Serum;  Urine | n.r. | 0.1-6.6;  0.006-1 | n.r. | 36-45 | Prob. high |
| Ramjee, 1992^274^ | South Arica | 1992 | Caco | 48(13/35)  45(10/35) | n.r. | 0.5-2 | AFB1, AFB2, AFG1, AFG2, AFM1 | TLC + HPLC-FD | Serum;  Urine | 0.001-0.05 (1-50); 0.005-0.03(5-25) | 0.1-0.2;  n.r. | n.r. | 0-23;  0-19 | Prob. Low |
| Househam, 1991^312^ | South-Africa | 1991 | CaCo | 337(17/320) | M/F | 1-5 | AFB1, AFB2, AFG1, AFG2, AFL | HPLC-FLD | Urine | 20 | n.r. | n.r. | 0 | Prob. Low |
| Oyelami, 1995^85^ | Nigeria | 1995 | Cs | 1 | M | 0.3 | AFM2 | HPLC-FLD | Tissue (kidney) | 0.005-0.01 | 1 | n.r. | n.r. | Prob. High |
| Lamplugh, 1982^313^ | Nigeria and South-Africa | 1982 | Coh | 1 | n.r. | n.r. | AFB1, AFL, AFM1 | HPLC-MS/MS | Tissue (liver) | n.r. | n.r. | n.r. | n.r. | Def. High |
| McMillan, 2018^314^ | Nigeria | 2012 | CaCo | 32(21/11) | n.r. | 0.5-4 | AFB1-lysine | LC-MS/MS | Plasma | n.r. | 0.008-0.6 | n.r. | n.r. | Prob. Low |
| Hendrickse, 1983^315^ | Nigeria and South-Africa | 1983 | Cs | 3 | n.r. | n.r. | AFB1, AFL, AFM1, AF | HPLC-FLD | Tissue (liver) | n.r. | n.r. | n.r. | 0-19 | Prob. High |
| Hendrickse 1984^316^ | Sudan | 1984 | Cs | 468;  469;  468 | M/F | n.r. | AFG1, AFG2, AFB2, AFM1, AFM2, AFB1, AFL | HPLC-FLD | Faeces;  Serum;  Urine | n.r. | 0.2-9;  0.2-9;  0.03-0.4 | n.r. | 0 | Prob. Low |
| *Marasmic kwashiorkor* | | | | | | | | | | | | | | |
| Hendrickse, 1982^305^ | Sudan | 1981 | CaCo | 76(32/44) | M/F | n.r. | AFB1, AFL | HPLC-FLD | Serum;  Urine | n.r. | 0.4;  0.7 | n.r. | n.r. | Prob. Low |
| Coulter, 1986^308^ | Sudan | 1981-1983 | CaCo | 201(98/103)  232(77/155) | n.r. | 0.6-5 | AFB1, AFB2, AFG1, AFG2, AFM1, AFM2, AFL | HPLC-FLD + 2D TLC | Serum;  Urine | 1-50;  5-25 | 0.08;  0.5 | n.r. | 6-7;  5-9 | Prob. Low |
| De Vries, 1987^311^ | Kenya | 1984-1985 | CaCo | 16(6/10) | n.r. | 4^mean^ | AFB1 | HPLC-FLD | Serum;  Urine | n.r. | 0.04-1;  0.06-4.4 | n.r. | 50-60 | Prob. High |
| Lamplugh, 1982^313^ | Nigeria and South-Africa | 1982 | Coh | 3 | n.r. | n.r. | AFB1, AFL, AFM1 | HPLC-MS/MS | Tissue (liver) | n.r. | n.r. | n.r. | n.r. | Def. High |
| Tchana, 2010^186^ | Cameroon | 1991-1995 | CaCo | 78(42/36) | M/F | 1-12 | AFB1 | HPLC-FLD | Urine | n.r. | 0.1-1 | n.r. | 46 | Prob. Low |
| Hendrickse, 1983^315^ | Nigeria and South-Africa | 1983 | Cs | 6 | n.r. | n.r. | AFB1, AFL, AFM1, AF | HPLC-FLD | Tissue (liver) | n.r. | 0.02-9 | n.r. | 0-67 | Prob. High |
| Hendrickse 1984^316^ | Sudan | 1984 | Cs | 468;  468;  469 | M/F | n.r. | AFG1, AFG2, AFB2, AFM1, AFM2, AFB1, AFL | HPLC-FLD | Faeces;  Urine;  Serum | n.r. | 0.15;  0.04-7.6;  0.003-3.4 | n.r. | 6 | Prob. Low |

**Note: N = sample size; Ca = cases; Co = controls; LOD = limit of detection; LOQ = limit of quantification; Conc. = concentration; OR = odds ratio; RR = relative risk; CC = correlation coefficient; CI = confidence interval; AR = attack rate; RoB = Risk of Bias according to OHAT; Cs = cross-sectional study; Coh = cohort study; CaCo = case-control study; NCC = nested case-control; CaR = case report; n.r. = not reported; TLC = thin layered chromatography; U(H)PLC-MS = ultra(high) performance liquid chromatography - mass spectrometry; FLD = fluorescence light detection; M/F = Male/Female.**

# **Table 25 Blood diseases**

| **Reference** | **Population** | **Study period** | **Study design** | **N(Ca/Co)** | **Gender** | **Age (years)** | **Mycotoxin** | **Method** | **Matrix** | **LOD (LOQ)**  **(µg/L)** | **Conc.**  **(µg/L)** | **OR/RR/CC/AR**  **(95% CI)** | **Prevalence**  **(%)** | **RoB** |
| --- | --- | --- | --- | --- | --- | --- | --- | --- | --- | --- | --- | --- | --- | --- |
| *Anemia* | | | | | | | | | | | | | | |
| Lei, 2021^318^ | China | 2016-2017 | Coh | 616 | F | 29^mean^ | AFB1-albumin | ELISA | Serum | n.r. | 0.6 | 4-14^OR^ | n.r. | Def. Low |
| Shuaib, 2010^319^ | Ghana | 2006 | Cs | 755 | F | 20-30 | AFB1-lysine | HPLC-FLD | Serum | 0.0005 | 0.02-11 | 1.2-1.8^OR^ | n.r. | Def. Low |
| Oyelami,1997^95^ | Nigeria | 1997 | OuR | 20 | M/F | 0.5-14 | AFL, AFB1, AFB2, AFM1, AFM2, AFG1, AFG2 | HPLC-FLD | Tissue (lung) | 0.005  0.01^AFB1,AFG1^ | 0.1-1.7 | n.r. | n.r. | Prob. Low |
| Pratiwi, 2024^320^ | Indonesia | n.r. | Cs | 104 | F | n.r. | AFB1 | ELISA | Plasma | n.r. | 0.148 | n.r. | n.r. | Prob. High |
| Murashiki, 2025^321^ | Zimbabwe | 2020 | Coh | 129 | F | 31 | AFB1;  FB1 | ELISA | Urine | n.r. | 0.04 – 1.06^AFB1^  0.17 – 6.16^FB1^ | 13.72^OR^  (0.92 – 203.6)  10.68^OR^  (1.02 – 112.34) | n.r. | Prob. Low |
| *Burkitt’s lymphoma* | | | | | | | | | | | | | | |
| Oyelami, 1995^88^ | Nigeria | 1995 | Cs | 1 | F | 7 | AFB1, AFB2, AFG1, AFG2, AFL, AFM1, AFM3 | HPLC-FLD | Tissue (brain) | n.r. | 0 | n.r. | n.r. | Prob. Low |
| Oyelami, 1995^85^ | Nigeria | 1998 | Cs | 1 | F | 7 | AFB1, AFB2, AFG1, AFG2, AFM1 AFM2, AFL | HPLC-FLD | Tissue (kidney) | 0.005-0.01 | 0 | n.r. | n.r. | Prob. High |
| *postpartum hemorrhage* | | | | | | | | | | | | | | |
| Chen, 2022^322^ | China | 2016-2017 | Cs | 379 | F | 28^mean^ | AFB1-albumin | ELISA | Serum | n.r. | 0.4-0.8 | 1-5^OR^ | n.r. | Prob. Low |
| Pre-eclampsia | | | | | | | | | | | | | | |
| Moodley, 2001^323^ | South-Africa | n.r. | CaCo | 51(35/16) | F | 27.5 | FB1 | HPLC-FLD | Serum | n.r. | 0.0045 | n.r. | n.r. | Prob. Low |

**Note: N = sample size; Ca = cases; Co = controls; LOD = limit of detection; LOQ = limit of quantification; Conc. = concentration; OR = odds ratio; RR = relative risk; CC = correlation coefficient; CI = confidence interval; AR = attack rate; RoB = Risk of Bias according to OHAT; Cs = cross-sectional study; Coh = cohort study; NCC = nested case-control; CaR = case report; n.r. = not reported; U(H)PLC-MS = ultra(high) performance liquid chromatography - mass spectrometry; FLD = fluorescence light detection; ELISA = Enzyme-linked immunosorbent assay; M/F = Male/Female**

# **Table 26 Lung diseases**

| **Reference** | **Population** | **Study period** | **Study design** | **N(Ca/Co)** | **Gender** | **Age (years)** | **Mycotoxin** | **Method** | **Matrix** | **LOD (LOQ)**  **(µg/L)** | **Conc.**  **(µg/L)** | **OR/RR/CC/AR**  **(95% CI)** | **Prevalence**  **(%)** | **RoB** |
| --- | --- | --- | --- | --- | --- | --- | --- | --- | --- | --- | --- | --- | --- | --- |
| *Bronchopneumia* | | | | | | | | | | | | | | |
| Ali, 2013^324^ | India | 2011-2012 | CaCo | 65(40/25) | M/F | 45-60 | AFB1-lysin | ELISA | Serum | n.r. | 192 - 500 | n.r. | 25 | Prob. Low |
| Oyelami, 1997^95^ | Nigeria | 1997 | OuR | 20 | M/F | 0.5-14 | AFL, AFB1, AFB2, AFM1, AFM2, AFG1, AFG2 | HPLC-FLD | Tissue (lung) | 0.005  0.01^AFB1,AFG1^ | 0.6-90 | n.r. | n.r. | Prob. Low |
| *Lung cancer* | | | | | | | | | | | | | | |
| Dvorackova, 1981^325^ | n.r. | 1981 | CaR | 2 | M | 59-68 | AFB1 | TLC-UV | Tissue (lung) | n.r. | n.r. | n.r. | n.r. | Prob. Low |
| Cusumano, 1991^326^ | n.r. | 1991 | NCC | 250(100/150) | M/F | 40-75 | AFB1, AFB2, AFG1, AFG2 | HPLC | Serum | n.r. | 0.4-1.2 | n.r. | 0.7-7 | Prob. Low |
| Lauer, 2019^281^ | Uganda | 2017 | Cs | 220 | M/F | n.r. | AFB1-lysin | HPLC-FLD | Maternal serum | 0.2 | 0.03-4 | n.r. | n.r. | Def. Low |
| *Tuberculous* | | | | | | | | | | | | | | |
| Oyelami, 1997^95^ | Nigeria | 1997 | OuR | 20 | M/F | 0.5-14 | AFL, AFB1, AFB2, AFM1, AFM2, AFG1, AFG2 | HPLC-FLD | Tissue (lung) | 0.005  0.01^AFB1,AFG1^ | 0-4.5 | n.r. | n.r. | Prob. Low |

**Note: N = sample size; Ca = cases; Co = controls; LOD = limit of detection; LOQ = limit of quantification; Conc. = concentration; OR = odds ratio; RR = relative risk; CC = correlation coefficient; CI = confidence interval; AR = attack rate; RoB = Risk of Bias according to OHAT; Cs = cross-sectional study; Coh = cohort study; CaCo = case-control study; NCC = nested case-control; CaR = case report; n.r. = not reported; TLC = thin layered chromatography; U(H)PLC-MS = ultra(high) performance liquid chromatography - mass spectrometry; FLD = fluorescence light detection; UV = Ultraviolet detection . ELISA = Enzyme-linked immunosorbent; M/F = Male/Female.**

# **Table 27 Pancreatic cancer**

| **Reference** | **Population** | **Study period** | **Study design** | **N(Ca/Co)** | **Gender** | **Age (years)** | **Mycotoxin** | **Method** | **Matrix** | **LOD (LOQ)**  **(µg/L)** | **Conc.**  **(µg/L)** | **OR/RR/CC/AR**  **(95% CI)** | **Prevalence**  **(%)** | **RoB** |
| --- | --- | --- | --- | --- | --- | --- | --- | --- | --- | --- | --- | --- | --- | --- |
| *Pancreatic cancer* | | | | | | | | | | | | | | |
| Rodriguez, 2025^327^ | USA | Coh | CaCo | 40(20/20) | M/F | 45-82 | OTA;CIT  AFM1;CIT;  ZEN | LC-MS/MS | Urine | n.r. | n.r. | n.r. | n.r. | Prob. Low |

**Note: N = sample size; Ca = cases; Co = controls; LOD = limit of detection; LOQ = limit of quantification; Conc. = concentration; OR = odds ratio; RR = relative risk; CC = correlation coefficient; CI = confidence interval; AR = attack rate; RoB = Risk of Bias according to OHAT; Cs = cross-sectional study; Coh = cohort study; CaCo = case-control study; NCC = nested case-control; CaR = case report; n.r. = not reported; TLC = thin layered chromatography; U(H)PLC-MS = ultra(high) performance liquid chromatography - mass spectrometry; FLD = fluorescence light detection; UV = Ultraviolet detection . ELISA = Enzyme-linked immunosorbent; M/F = Male/Female.**

# **Table 28 Detailed overview, including the methodology & rationale for decisions of the RoB assessment of the included eligible studies.**

| Source | 1. Did selection of study participants result in appropriate comparison groups? | 2. Did the study design or analysis account for important confounding and modifying variables? | 3. Were outcome data complete without attrition or exclusion from analysis? | 4. Can we be confident in the exposure characterization? | 5. Can we be confident in the outcome measure? | 6. Were all measured outcomes reported? | Overall RoB |
| --- | --- | --- | --- | --- | --- | --- | --- |
| CRITERIA | - Definitely low if 3 criteria fulfilled: controls of same age, sex and population - Probably low: 2 out of 3 fulfilled - Probably high: 1 out of 3 fulfilled - Definitely high: none fulfilled or no information | - Definitely low if 3 criteria fulfilled: matching of groups^328^, taking into account at least one reliable influencing factor during selection, adjusted for at least one reliable confounder (WCRF matrix for cancer, literature for others)^329^ - Probably low: 2 out of 3 criteria fulfilled - Probably high: 1 out of 3 criteria fulfilled - Definitely high: no criteria fulfilled | - Definitely low: all included cases were measured or non-measures are verified - Probably low: presumably all was measured (no reports of non-measures) - Probably high: indirect evidence that not all was measured - Direct evidence that not all cases were measured without further explanation | - Definitely low: MYCs measured in biological samples ^331^ AND appropriate study design (i.e. cohort study for chronic disease and Cs for acute diseases) - Probably low: MYCs measured in food ^331^ OR exposure measured in biological samples but utilizing a non-reliable study design for the disease type (e.g., Cs study for a chronic disease) - Probably high: very little info on the exposure measurement, regardless of the study design - Definitely high: no info on exposure measurement | - Definitely low: diagnosed in a hospital setting using objective clinical gold standards (radiology or specific biomarkers) - Probably low: diagnosed via trained personnel outside hospital setting or general medical records but lacking explicit radiology/biomarker confirmation. - Probably high: diagnosed via questionnaire, self-report, or indirect clinical proxies - Definitely high: no info on the diagnosis method provided | - Definitely low: direct proof that all outcomes were documented (*i.e.* hospital records) - Probably low: indirect proof that all outcomes were documented (*i.e.* medical records) - Probably high: indirect proof that not all outcomes have been documented (*e.g*. loss of follow-up, outbreak reports) - Definitely high: direct proof that not all outcomes have been documented (*e.g.* biased inclusion) | - Definitely low: All key domains (2, 4, 5) are low, and the majority of other applicable domains are also low - Probably low: Most domains (including at least two of the key domains 2, 4, 5) are low, but there is some uncertainty due to one or more domains being high. - Probably high: Most domains (including at least two of the key domains 2, 4, 5) are high, but there is some uncertainty due to one or more domains being low. - Definitely high: All key domains (2, 4, 5) are high, and the majority of other applicable domains are also high. |
| Gong, 2002 (underweight) | Definitely low RoB | Definitely low RoB | Definitely low RoB | Probably low RoB | Probably low RoB | Probably low RoB | Probably low |
| Rationale | *Multivariable model adjusting for age, sex, socioeconomic status, and agroecological zone* | *Matched, taking into account reliable influencing factors during selection, adjusted for confounding.* | *449 of the 450 samples are measured and the one that is not measured is stated missing* | *AFB1 measured in biological samples in a cross-sectional study (not an appropriate study design)* | *Trained personnel* | *Medical records via trained personnel* |  |
| El-azab, 2009 (neurologic disease) | Probably low RoB | Probably high RoB | Definitely low RoB | Probably low RoB | Definitely low RoB | Probably low RoB | Probably low |
| Rationale | *Age, sex of controls is known and similar to the cases – no info on population origin* | *Unmatched, reliable influencing factors were taken into account, but no confounders* | *All included persons were measured* | *AFB1 measured in biological samples (i.e. serum), but no appropriate study design (CaCo)* | *Diagnosed in hospital setting* | *Medical records* |  |
| Ibrahim, 2013 (liver disease) | Definitely low RoB | Probably high RoB | Definitely low RoB | Probably low RoB | Probably low RoB | Definitely low RoB | Probably low |
| Rationale | *Matched for age, sex and ethnicity* | *Matched, reliable measurement, not taken into account at least one reliable confounder, no information regarding other influencing factors* | *39 HCC patients were enrolled; 3 excluded due to refusal or insufficient samples, with no differences in key characteristics* | *OTA measured in biological samples (i.e. serum), but no appropriate study design (CaCo without nested design)* | *Diagnosed in hospital setting, medical records* | *Hospital records* |  |
| Hatem, 2005 (kwashiorkor) | Probably low RoB | Definitely low RoB | Definitely low RoB | Definitely low RoB | Definitely low RoB | Definitely low RoB | Definitely low |
| Rationale | *Matched for age and gender* | *Matched, taking into account other influencing factors (liver disease measurements) and confounders* | *No exclusions from analysis* | *AFs measured in biological samples (i.e. urine & serum), case-control study design* | *Diagnosed in hospital setting, according to WHO criteria* | *Hospital records of all cases* |  |
| Zaied, 2011 (kidney disease) | Probably high RoB | Probably low RoB | Definitely low RoB | Probably low RoB | Definitely low RoB | Definitely low RoB | Probably low |
| Rationale | *Matched for age, but not for sex and population origin* | *Not fully matched, taking into account other influencing factors* | *No exclusion from analysis* | *OTA was measured in serum, study design not appropriate for disease type (CaCo without nested design)* | *Diagnosed in hospital setting (biomarkers)* | *Hospital records* |  |
| Samuel, 2009 (acute food intoxication) | Definitely high RoB | Probably high RoB | Definitely low RoB | Probably low RoB | Probably low RoB | Probably low RoB | Probably low |
| Rationale | *Outbreak reports – no controls* | *Unmatched, measurement only in food, recognition influencing factors, no confounders were taken into account* | *No exclusions from analysis (there was only 1 analysis)* | *Measurement in food,* | *Diagnosed in medical centre, but lacking specific biomarker confirmation* | *Medical records* |  |
| Bulatao-Jayme, 1982 (liver disease) | Definitely low RoB | Probably high RoB | Probably high RoB | Probably low RoB | Definitely low RoB | Definitely high RoB | Probably low |
| Rationale | *Matched by age, gender and same population within hospital setting, not matched for socio-economic status* | *Matched, not taking into account other influencing factors, no confounders taken into account* | *Urine samples are collected from 76 out of 90 cases and 89 out of 90 controls -> reason why not from everyone is unknown* | *MYCs measured in urine and food* | *Diagnosed in hospital setting, biomarker analysis* | *Records not reported for all included cases* |  |
| Alvarez, 2020 (liver disease) | Definitely low RoB | Definitely low RoB | Probably high RoB | Definitely low RoB | Definitely low RoB | Definitely low RoB | Definitely low |
| Rationale | *Matched for age, gender and population origin* | *Matched, taking into account influencing factors and confounding factors (i.e. heavy alcohol consumption, viral infections, occupation, etc.)* | *Exclusion of 1 person from analysis without more information* | *AFB1-lysine measurement in serum, in a case-control study* | *Diagnosed in hospital setting, ultrasonography with scoring system was used* | *All-reporting hospital record* |  |
| Ramalho, 2018 (liver disease) | Probably low RoB | Probably low RoB | Definitely low RoB | Probably low RoB | Probably low RoB | Definitely low RoB | Definitely low |
| Rationale | *Matched for age and within the same hospital* | *Not fully matched, influencing factors taken into account (i.e. HIV, auto-immune, drug-induced hepatitis, endocrine disorders), also confounding factors taken into account (i. e. alcohol, viral hepatitis)* | *No exclusion from analysis* | *Tissue measurements (post-mortem case-control study not appropriate for longitudinal disease)* | *Diagnosed in hospital setting, but not specified how patients were diagnosed* | *Hospital records* |  |
| Lopez, 2002 (liver disease) | Probably high RoB | Probably low RoB | Definitely low RoB | Probably low RoB | Probably low RoB | Definitely low RoB | Definitely low |
| Rationale | *No info about the population of the controls, not from the same hospital, different characteristics* | *No matching, taking influencing factors and confounders into account (i. e. residence, hepatitis B antigen status, smoking habits, alcohol consumption)* | *No exclusion from analysis* | *Serum measurement, but not an appropriate design for hepatic longitudinal disease (CaCo without nested design)* | *Diagnosed in hospital setting, but no information on how diagnosis took place* | *Hospital records* |  |
| Coulter, 1986 (liver disease) | Probably low RoB | Probably low RoB | Probably high RoB | Probably low RoB | Probably low RoB | Definitely low RoB |  |
| Rationale | *Age and population origin matched* | *Not fully matched, taking influencing factors into account, but no confounding factors* | *Indirect evidence that not all persons per group were analyzed* | *Serum measurement but not an appropriate study design for longitudinal disease* | *Diagnosed in hospital setting, but no information on disease criteria for diagnosis* | *Hospital records* | Definitely low |
| Grosso, 2003(bladder cancer) | Definitely high RoB | Definitely high RoB | Definitely low RoB | Probably low RoB | Probably low RoB | Definitely low RoB | Probably high |
| Rationale | *Not matched* | *Not matched, not taking into account other influencing factors, or confounding factors* | *No exclusion from analysis* | *Serum measurement, but not an appropriate study design for longitudinal disease* | *Diagnosed in hospital setting, but no explicit diagnostic criteria* | *Hospital records* |  |
| Al-Mhanaa, 2020 (kidney disease) | Definitely high RoB | Definitely high RoB | Probably high RoB | Probably low RoB | Probably low RoB | Definitely low RoB | Probably high |
| Rationale | *No info on controls* | *No matching, no influencing factors or confounders taken into account* | *Indirect evidence of non-measures* | *Serum measurement, but case-control study without nested design* | *Diagnosed in hospital setting, but no information on diagnostic criteria* | *Hospital records* |  |
| Apeagyei, 1986 (kwashiorkor) | Definitely high RoB | Probably high RoB | Probably low RoB | Probably low RoB | Probably high RoB | Probably high RoB | Probably high |
| Rationale | *No controls – case series* | *No matching, taking influencing factors into account, no confounding factors (because of study design)* | *No reports of non-measurements but little information* | *Tissue measurement (post-mortem), appropriate design for* | *No info on diagnosis, but autopsy was performed so presumable medical setting* | *No info diagnosis* |  |
| Koshiol, 2017 (gallbladder cancer) | Probably low RoB | Probably low RoB | Definitely low RoB | Probably low RoB | Probably high RoB | Probably low RoB | Probably low |
| Rationale | *Matched for age, gender* | *Not fully matching, relevant influencing factor taken into account (gallstones), no confounding factors taken into account* | *All included patients were analysed* | *Plasma measurement (with golden standard), but case-control study without nested design* | *Forty patients with cancer did not have gallstones indicated through questionnaire, medical record abstraction, ultrasound, specimen collection, or histology, or a combination of these, and 1 patient with cancer had missing gallstone status* | *Medical records* |  |
| Thé, 2015 (kidney disease) | Definitely high RoB | Definitely high RoB | Definitely low RoB | Probably low RoB | Definitely low RoB | Probably low RoB | Probably low |
| Rationale | *No info on age, gender, ethnicity* | *No matching, no other influencing factors taken into account, not adjusted* | *All included patients/controls were measured* | *Serum measurement but non-reliable study design for chronic outcome* | *Diagnosed in hospital (imaging/biochemistry)* | *Hospital records but little info on controls originating from the same department in urinary disease* |  |
| Pillay, 2002 (female reproductive disease) | Definitely high RoB | Probably high RoB | Definitely low RoB | Probably low RoB | Definitely low RoB | Probably low RoB | Probably low |
| Rationale | *No info on age, gender, ethnicity* | *Age matched, no influencing factors, not adjusted for confounding* | *All included patients/controls were measured* | *Plasma measurement but non-reliable study design for chronic outcome* | *Diagnosed in hospital (imaging/biochemistry)* | *Hospital records but little info on controls originating from the same department in gynaecology* |  |
| Chen, 2023 (intestinal disease) | Definitely high RoB | Probably low RoB | Definitely high RoB | Definitely low RoB | Probably low RoB | Definitely high RoB | Probably low |
| Rationale | *No controls* | *No matching, taking into account influencing factors, adjusted for confounding* | *48 out of 196 were measured, no further explanation* | *Serum measurement (golden standard) and reliable study design* | *Trained personnel* | *Medical records but missing for 48* |  |
| Hassan, 2006 (kidney disease) | Definitely high RoB | Probably high RoB | Probably high RoB | Probably low RoB | Definitely low RoB | Definitely low RoB | Probably low |
| Rationale | *No controls* | *No matching, not taking into account reliable influencing factors for selection, adjusted for confounding* | *Only reported for 36 out of 50 children* | *Serum measurement and reliable study design* | *Diagnosed in hospital (imaging/biochemistry)* | *Hospital records* |  |
| Abid, 2003 (kidney disease) | Probably high RoB | Definitely high RoB | Probably low RoB | Definitely low RoB | Probably low RoB | Probably low RoB | Probably low |
| Rationale | *No matching age, gender, but from same region* | *No matching, not taking into account reliable influencing factors for selection, although variables noted (dietary habits, antecedents, etc) not matched for in statistics.* | *Presumably all samples analysed but no individual data* | *Blood/urine measurement and reliable study design* | *For some in hospital (imaging/biochemistry), for others no info on diagnosis* | *Medical reports for all, but not always in a hospital setting* |  |
| Mahdavi, 2010 (stunting & underweight) | Definitely high RoB | Probably low RoB | Definitely low RoB | Probably low RoB | Probably low RoB | Probably low RoB | Definitely low |
| Rationale | *No controls* | *Not matched, taking into account influencing factors, adjusted for confounding* | *All included patients were analyzed* | *Breastmilk measurement but suboptimal study design* | *Trained personnel* | *Medical records* |  |
| Özçelik, 2001 (bladder cancer) | Probably high RoB | Definitely high RoB | Definitely low RoB | Probably low RoB | Probably high RoB | Probably high RoB | Probably high |
| Rationale | *Matched for age but not gender or origin* | *No full matching, not taking into account reliable influencing factors for selection, not adjusted for confounding* | *All was analyzed* | *Serum measurement but suboptimal study design* | *No info on diagnosis, but treated with dialysis at a presumable centre* | *Little information on reports* |  |
| Malir, 2019 (kidney disease) | Definitely high RoB | Probably high RoB | Definitely low RoB | Probably low RoB | Definitely low RoB | Definitely low RoB | Probably low |
| Rationale | *Completely different population* | *No matching, influencing factors accounted for, not adjusted for reliable confounding* | *All was analyzed* | *Serum measurement but non-reliable study design for chronic outcome* | *Diagnosed in hospital (biochemistry/imaging)* | *Hospital records* |  |
| Wray, 1980 (liver disease) | Definitely high RoB | Probably high RoB | Definitely low RoB | Probably low RoB | Definitely low RoB | Probably low RoB | Probably low |
| Rationale | *Case report* | *No matching, taking into account influencing factors (e.g. alcohol misuse), not adjusted for reliable confounding* | *All was analyzed* | *Serum measurement but non-reliable study design* | *Diagnosed in hospital (biochemistry/imaging)* | *Medical records* |  |
| Belhassen, 2014 (female reproductive disease) | Probably low RoB | Probably low RoB | Definitely low RoB | Probably low RoB | Definitely low RoB | Definitely low RoB | Definitely low |
| Rationale | *Matched for age, sex, etchnicity* | *Matching, no influencing factors accounted for during selection, however adjusted for reliable confounding* | *All was analyzed* | *Serum measurement (golden standard), non-reliable study design* | *Diagnosed in hospital (biochemistry/imaging)* | *Hospital records* |  |
| Cusumano, 1991 (lung disease) | Probably low RoB | Probably high RoB | Definitely low RoB | Probably low RoB | Definitely low RoB | Definitely low RoB | Probably low |
| Rationale | *Matched for age, gender, not etchnicity* | *Matching, no influencing factors accounted for during selection, not although smoking history noted, not adjusted for in analysis* | *All was analyzed* | *Serum measurement & appropriate study design* | *Diagnosed in Hospital (biochemistry/imaging/biopsies)* | *Hospital records* |  |
| Nyikal, 2004 (acute food intoxication) | Definitely high RoB | Probably high RoB | Probably high RoB | Probably low | Probably low RoB | Probably high RoB | Probably low |
| Rationale | *Outbreak report* | *No matching, influencing factors accounted for, not adjusted for reliable confounding* | *Discontinuation of follow-up in health centres + inherent to outbreaks: less severe cases not admitted* | *Food measurement + appropriate study design* | *Medical reports for all, but not always in a hospital setting* | *Medical record review & patient interviews, however discontinuation of follow-up + less severe cases not admitted to health facility* |  |
| Maaroufi, 1995 (kidney disease) | Probably high RoB | Probably high RoB | Probably low RoB | Probably low | Definitely low RoB | Definitely low RoB | Probably low |
| Rationale | *Only matched for ethnicity* | *Not fully matched, not taking into account influencing factors, not adjusted for reliable confounding* | *No reports of non-measurements* | *Biological matrix, suboptimal study design* | *Diagnosed in hospital (biochemistry/imaging)* | *Hospital records* |  |
| Hmaissia Khalifa, 2008 (kidney disease) | Probably low RoB | Probably low RoB | Probably low RoB | Probably low RoB | Probably low RoB | Probably low RoB | Probably low |
| Rationale | *Matched for age, gender, not ethnicity* | *Matched design, influence factor considered, lacks info on covariates* | *No reports of non-measures* | *Biological matrix but no appropriate study design (CaCo but not nested)* | *Medical centres but lacks info on diagnosis* | *Medical records* |  |
| Abdulrazzaq, 2004 (adverse birth outcomes) | Definitely high RoB | Definitely high RoB | Definitely high RoB | Definitely low RoB | Definitely low RoB | Probably low RoB | Probably low |
| Rationale | *No controls* | *Not matched, no influencing factors taken into account, not adjusted for confounding* | *Only 250 out of 1600 samples, no further explanation* | *Serum measurement & appropriate study design* | *Hospital setting (biochemistry)* | *Hospital records* |  |
| Asci, 2024 (premature puberty) | Definitely low RoB | Probably low RoB | Definitely low RoB | Probably low RoB | Definitely low RoB | Definitely low RoB | Definitely low |
| Rationale | *Matched for age, gender, ethnicity* | *Matched, influencing factors accounted for, not adjusted for reliable confounding* | *All was analyzed* | *Biological matrix, suboptimal study design* | *Hospital setting (biochem/imaging)* | *Hospital records* |  |
| Tian, 1995 (liver disease) | Definitely high RoB | Definitely high RoB | Probably high RoB | Probably low RoB | Probably low RoB | Probably low RoB | Probably low |
| Rationale | *No info on age, gender or ethnicity* | *No matching, no influencing factors accounted for, not adjusted for confounding* | *Indirect evidence that not all was reported* | *Biological matrix, inappropriate study design* | *Hospital and medical centre setting* | *Hospital and medical records* |  |
| Sodeinde, 1995 (liver disease) | Definitely low RoB | Probably low RoB | Definitely high RoB | Definitely low RoB | Definitely low RoB | Definitely low RoB | Definitely low |
| Rationale | *Matched for age, gender, ethnicity* | *Matched, no influencing factors accounted for, adjusted for potential confounding* | *Only 270 out of 327 tested* | *Biological matrix, appropriate study design* | *Hospital setting (biochemistry/imaging)* | *Hospital records* |  |
| Adhikari, 1994 (kwashiorkor) | Definitely high RoB | Definitely high RoB | Definitely low RoB | Probably low RoB | Probably low RoB | Definitely low RoB | Probably low |
| Rationale | *No controls* | *No matching, no influencing factors taken into account, no adjustment for reliable confounding* | *All was analyzed* | *Serum measurement, suboptimal study design* | *although in hospital setting, diagnosis mainly based on clinical features (no biochemistry/imaging)* | *Hospital records* |  |
| Olubuyide, 1992 (liver disease) | Definitely high RoB | Defenitely high RoB | Definitely low RoB | Probably low RoB | Definitely low RoB | Definitely low RoB | Probably low |
| Rationale | *No controls* | *No matching, no influencing factors accounted for, although noted, not adjusted for reliable confounding (HBV, smoking, alcohol, etc.)* | *All was analyzed* | *Serum measurement, inappropriate study design* | *Hospital setting (biopsies, imaging, biochemistry)* | *Hospital records* |  |
| Stora, 1983 (neurologic disease) | Definitely high RoB | Definitely high RoB | Probably high RoB | Definitely low RoB | Probably high RoB | Probably high RoB | Probably high |
| Rationale | *No controls* | *No matching, no influencing factors accounted for, no adjustment for reliable confounding* | *No info on selection – less severe cases likely omitted* | *Tissue measurement, appropriate study design* | *No info on diagnosis but autopsy indicated cerebral edema* | *Indirect proof that not all has been documented* |  |
| Oyelami, 1995 (gastric disease) | Probably low | Probably high RoB | Probably high RoB | Probably low RoB | Probably high RoB | Probably high RoB | Probably high |
| Rationale | *Matched for age and ethnicity* | *matched, no influencing factors taken into account, no adjustment for reliable confounding* | *No info on selection* | *Biological matrix, inappropriate study design* | *although in hospital setting very little info on diagnosis* | *Hospital records* |  |
| Manda, 2018 (liver disease) | Definitely high RoB | Probably high RoB | Probably high RoB | Probably low | Probably high RoB | Probably high RoB | Probably high |
| Rationale | *No matching* | *No matching, no influencing factors taken into account, adjustment for at least one reliable confounding* | *No info on selection* | *Biological matrix, inappropriate study design* | *No info on diagnosis* | *No info on diagnosis or other clinical variables* |  |
| Coulter, 1986 (liver disease) | Definitely high RoB | Definitely high RoB | Probably high RoB | Probably low RoB | Definitely low RoB | Probably low RoB | Probably low |
| Rationale | *No controls* | *no matching, no influencing factors taken into account, not adjusted for confounding* | *No info on selection* | *Biological matrix, inappropriate study design* | *Hospital setting (biochemistry/biopsies/imaging)* | *Hospital medical records* |  |
| Roy, 1992 (intestinal disease) | Definitely high RoB | Definitely high RoB | Probably high RoB | Probably low RoB | Definitely high RoB | Definitely high RoB | Probably high |
| Rationale | *No controls* | *no matching, no influencing factors taken into account, not adjusted for confounding* | *No info on selection* | *Biological matrix, inappropriate study design* | *No info on diagnosis* | *No info on diagnosis* |  |
| Aslam, 2005 (bladder cancer) | Definitely high RoB | Definitely high RoB | Probably high RoB | Probably low RoB | Definitely low RoB | Probably low RoB | Probably low |
| Rationale | *No info on age, gender, ethnicity* | *No matching, no influencing factors accounted for, no adjustment for reliable confounding* | *No info on selection* | *Biological matrix, inappropriate study design* | *Histology confirmed in hospital setting* | *Confirmed histology but no other reports* |  |
| Arce-Lopez, 2021 (neurologic disease) | Probably low RoB | Probably high RoB | Definitely high RoB | Probably low RoB | Definitely low RoB | Definitely low RoB | Probably low |
| Rationale | *Controls same age, healthy companions, same population* | *Matched, no influencing factors accounted for, no adjustment for reliable confounding* | *Direct evidence (not enough sample volume) that not all was analyzed* | *Biological matrix, inappropriate study design* | *Histology confirmed in hospital setting* | *Confirmed histology but no other reports* |  |
| Turner, 2003 (immunomodulation) | Definitely high RoB | Probably high RoB | Definitely high RoB | Definitely low RoB | Probably low RoB | Probably low RoB | Probably low |
| Rationale | *No controls* | *No matching, no influencing factors accounted for, adjusted for reliable confounding* | *Direct evidence (not enough sample volume) that not all was analyzed* | *Biological matrix, appropriate study design* | *Trained personnel, immunity testing* | *Reports by medical trained persons on-site* |  |
| Househam, 1991 (kwashiorkor) | Probably high RoB | Probably low RoB | Probably high RoB | Probably low RoB | Probably high RoB | Probably low RoB | Probably low |
| Rationale | *No info on age and gender* | *little matching, no influencing factors accounted for, adjusted for reliable confounding* | *23 urine specimens selected at random were analysed in the laboratory* | *Biological matrix, inappropriate study design* | *Community centre on-site, very little info on diagnosis* | *Recorded by district managers* |  |
| Nogueira, 2016 (gallbladder cancer) | Definitely low RoB | Definitely low RoB | Probably high RoB | Probably low | Definitely low RoB | Definitely low RoB | Definitely low |
| Rationale | *Age, gender & ethnicity matched* | *Matching, influencing factors taken into account, adjusted for reliable confounding* | *Different participation rates* | *Biological matrix, inappropriate study design* | *Hospital setting (biochemistry/imaging)* | *Hospital records* |  |
| Leroy, 2018 (stunting) | Definitely high RoB | Probably low RoB | Probably high RoB | Definitely low RoB | Probably low RoB | Probably low RoB | Probably low |
| Rationale | *no controls* | *no matching, not taken into account influencing factors, adjusted for reliable confounding* | *indirect evidence that not all samples were analyzed* | *Biological matrix, appropriate study design* | *Trained personel* | *Reports by medical personnel on site* |  |
| De Vries, 1987 (kwashiorkor) | Probably high RoB | Probably high RoB | Probably low RoB | Probably low RoB | probably high RoB | Definitely low RoB | Probably high |
| Rationale | *Similar age, no info on sex, other diseases* | *very little matched, no influencing factors taken into account, not adjusted for confounding* | *No reports of non-measures* | *Biological matrix, inappropriate study design* | *although in hospital setting very little info on diagnosis* | *Hospital records* |  |
| Duringer, 2021 (neurologic disease) | Probably low RoB | Probably high RoB | Definitely low RoB | probably low RoB | Probably low RoB | Probably low RoB | Probably low |
| Rationale | *Age and gender matched, no additional info* | *partially matched, no influencing factors taken into account, not adjusted for confounding* | *All was analyzed* | *Biological matrix, inappropriate study design* | *Rsearch study centre* | *Reports by research personnel on site* |  |
| Moodley, 2001 (blood diseases) | Probably low RoB | Probably high RoB | Probably low RoB | definitely low RoB | Probably low RoB | Probably low RoB | Probably low |
| Rationale | *Age and parity matched* | *Matched, no influencing factors, no confound adjustment* | *No reports of non-measured* | *Biological matrix, appropriate study design* | *Research study centre* | *Reports research personnel on site* |  |
| Ali, 2013 (lung diseases) | Probably low RoB | Probably high RoB | Definitely low RoB | Probably low RoB | Probably low RoB | Probably low RoB | Probably low |
| Rationale | *Age & gender matched without lung disease* | *Matched, not taking into account influencing factors, although reported not adjusted for confounding* | *All was analyzed* | *Biological matrix, inappropriate study design* | *Medical research centre* | *Medical reports* |  |
| Srivatanakul, 1991 (liver disease) | Probably low RoB | definitely low RoB | Definitely high RoB | Probably low RoB | Definitely low RoB | Definitely low RoB | Definitely low |
| Rationale | *Age & gender matched* | *Matched, influencing factors accounted for, adjusted for confounding* | *Direct evidence that not all patients were measured* | *Biological matrix, inappropriate study design* | *Hospital setting (biochemistry/imaging/biopsies)* | *Hospital records* |  |
| Wu, 2022 (liver disease & neurologic disease & metabolic disease) | Definitely high RoB | Probably high RoB | Probably low RoB | Probably low RoB | Definitely low RoB | Definitely low RoB | Probably low |
| Rationale | *No controls* | *Not matched, no influencing factors accounted for during selection, adjusted for confounding* | *No reports of non-measures* | *Biological matrix, inappropriate study design* | *Hospital setting (biochemistry/imaging)* | *Hospital records* |  |
| Ibeh, 1994 (male reproductive disease) | definitely high RoB | Definitely high RoB | Probably low RoB | Definitely high RoB | Definitely low RoB | Definitely low RoB | Probably high |
| Rationale | *No info on age or any other (only male)* | *No matching, no influencing factors accounted for, no adjusted for confounding* | *No reports of non-measures* | *No info on exposure identification method, inappropriate study* | *Diagnosed in hospital* | *Hospital records* |  |
| Chen, 1996 (liver disease) | Probably low RoB | Probably low RoB | Definitely high RoB | definitely low RoB | Probably low RoB | Probably low RoB | Probably low |
| Rationale | *Matched for age and gender* | *Matched, no influencing factors accounted for, adjusted for confounding* | *Not all cases were analysed* | *Biological matrix, appropriate study design* | *Medical research centre* | *Medical reports* |  |
| Schwerdt, 2001 (kidney disease) | Definitely high RoB | definitely high RoB | Probably low RoB | Probably low RoB | Definitely low RoB | Definitely low RoB | Probably low |
| Rationale | *No info on controls* | *No matching, no influencing factors accounted for, not adjusted for confounding* | *No reports of non-measures* | *Biological matrix, inappropriate study design* | *Hospital setting (biochemistry/imaging)* | *Hospital records* |  |
| Kuciel-Lisieska, 2008 (female reproductive disease) | Definitely high RoB | definitely high RoB | Probably low RoB | Probably low RoB | definitely low RoB | Probably low RoB | Probably low |
| Rationale | *No controls* | *not matched, no influencing factors accounted for, not adjusted for confounding* | *No reports of non-measures* | *Biological matrix, inappropriate study design* | *Oncology centre (imaging/biopsies)* | *Medical records* |  |
| RahmanEl-Zayadi, 2001 (liver disease) | Probably high RoB | Probably high RoB | Definitely high RoB | Probably low RoB | definitely low RoB | Probably low RoB | Probably low |
| Rationale | *No info* | *Not matched, no influencing factors accounted for, adjusted for confounding* | *Direct evidence that not all patients were measured* | *Biological matrix, suboptimal study design* | *Medical research centre (biopsies/imaging)* | *Medical records* |  |
| Murugavel, 2007 (liver disease) | Probably low RoB | Probably high RoB | Probably low RoB | Probably low RoB | Definitely low RoB | Definitely low RoB | Probably low |
| Rationale | *Little info on controls, age > 18 years* | *Not fully matched, influencing factors not taken into account, adjusted for confounding* | *No reports of non-measures* | *Biological matrix, inappropriate study design* | *Hospital setting* | *Hospital records* |  |
| Alamu, 2020 (wasting & underweight & stunting) | Definitely high RoB | Probably low RoB | Probably low RoB | probably low Rob | Definitely low RoB | Definitely low RoB | Definitely low |
| Rationale | *No controls* | *No matching, no* | *No reports of non-measures* | *Biological matrix, inappropriate study design* | *Hospital setting* | *Hospital records* |  |
| Asim, 2011 (liver disease) | definitely low RoB | Probably low RoB | Probably low RoB | Probably low RoB | Definitely low RoB | Definitely low RoB | *Definitely low* |
| Rationale | *Matched for age, gender, etchnicity* | *Matched, no influencing factors however adjusted for confounding* | *No reports of non-measures* | *Biological matrix, inappropriate study design* | *Hospital setting (biochemistry/biopsies)* | *Hospital records* |  |
| Perduri, 2009 (acute food intoxication) | Definitely high RoB | probably high RoB | Definitely low RoB | Definitely high RoB | probably high RoB | Probably low RoB | Probably high |
| Rationale | *Case report* | *No matching, no influencing factors, adjusted for confounding* | *Case report* | *No measurement in any human matrix nor food (feed of co-exposed animals), inappropriate study design* | *Although in hospital, diagnosed by exclusion* | *Hospital records of all parameters and symptoms – but by exclusion* |  |
| Qian, 1994 (liver disease) | Definitely high RoB | definitely high RoB | Definitely high RoB | definitely low RoB | Probably low RoB | Probably low RoB | Probably low |
| Rationale | *No info on controls* | *Not matched, no influencing factors, no adjustment for confounding* | *Direct evidence that not all was measured* | *Biological matrix, appropriate study design* | *Medical research center* | *Medical research records* |  |
| Shirima, 2015 (stunting) | Definitely high RoB | Probably high RoB | Probably low RoB | definitely low RoB | Probably low RoB | Probably low RoB | Probably low |
| Rationale | *No controls* | *Not matched, no influencing factors accounted for, adjusted for confounding* | *No reports of non-measures* | *biological matrix, appropriate study design* | *Trained personnel* | *Medical reports* |  |
| Ramjee, 1992 (underweight & kwashiorkor) | probably high RoB | Probably high RoB | Probably low RoB | Probably low RoB | Probably low RoB | Definitely low RoB | Probably low |
| Rationale | *Matched for age* | *Not fully matched, no influencing factors, not adjusted for confounding* | *No reports of non-measures* | *Biological matrix, inappropriate study design* | *Diagnosed in hospital, but little info on diagnosis* | *Hospital records* |  |
| Shouman, 2012 (underweight & stunting) | Definitely high RoB | Probably high RoB | Probably low RoB | Probably low RoB | Probably low RoB | Definitely low RoB | Probably low |
| Rationale | *No info on controls* | *Not matched, no influencing factors, adjusted for confounding* | *No reports of non-measures* | *Biological matrix, inappropriate study design* | *Diagnosed in hospital, but little info on diagnosis* | *Hospital records* |  |
| Peers, 1973 (liver disease) | Definitely high RoB | Definitely high RoB | Probably low RoB | Definitely high RoB | Probably low RoB | Definitely low RoB | Probably high |
| Rationale | *Case report* | *No matching, no influencing factors, not adjusted for confounding* | *No reports of non-measures* | *No info on diagnosis, inappropriate study design* | *Diagnosed in hospital but little info on diagnosis* | *Hospital records* |  |
| Chen, 2018 (underweight & stunting) | Definitely high RoB | Probably low RoB | Probably low RoB | probably low RoB | Definitely low RoB | Definitely low RoB | Definitely low |
| Rationale | *No controls* | *No matching, influencing factors accounted for, adjusted for confounding* | *No reports of non-measures* | *Biological matrix, inappropriate study design* | *Diagnosed in hospital* | *Hospital records* |  |
| Farag, 2018 (liver disease) | Definitely high RoB | Probably low RoB | Probably low RoB | Probably low RoB | Definitely low RoB | Definitely low RoB | Definitely low |
| Rationale | *No info on controls* | *No matching, accounted for influencing factors, no adjustment for confounding* | *No reports of non-measures* | *Biological matrix, inappropriate study design* | *Hospital setting (biochemistry/biopsies)* | *Hospital records* |  |
| Andrews-trevino, 2022 (underweight & stunting & adverse birth outcomes) | Definitely high RoB | Probably low RoB | Definitely high RoB | Probably low RoB | Probably low RoB | Probably low RoB | Definitely low |
| Rationale | *no controls* | *Not matched, influencing factors accounted for, adjusted for confounding* | *Direct evidence that not all patients were measured* | *Biological matrix, appropriate study design* | *Medical research centre* | *Medical reports* |  |
| Xue, 2019 (oesophageal disease) | Definitely low RoB | Probably low RoB | Probably high RoB | Probably low RoB | Probably low RoB | Probably low RoB | Definitely low |
| Rationale | *Controls of same age, sexes, and population* | *Matched, not accounted for influencing factors, adjust for confounding* | *No info on selection* | *Biological matrix, inappropriate study design* | *Malignant tumor registration record* | *Malignant tumor registration record* |  |
| Dvorackova, 1981 (lung diseases) | Definitely high RoB | definitely high RoB | Definitely low RoB | Probably low RoB | Probably low RoB | Probably low RoB | Probably low |
| Rationale | *Case report* | *No matching, , no influencing factors, not adjusted for confounding* | *Case report* | *Biological matrix, inappropriate study design* | *Malignant tumor registration record* | *Malignant tumor registration record* |  |
| Liu, 2022 (gastric disease) | Definitely high RoB | Probably high RoB | Probably low RoB | definitely low RoB | Definitely low RoB | Definitely low RoB | Probably low |
| Rationale | *No controls* | *No matching, influencing factors accounted for, no adjustment for confounding* | *No reports of non-measures* | *Biological matrix, appropriate study design* | *Hospital setting (biopsies/imaging)* | *Hospital records* |  |
| Stora, 1981 (liver disease) | Definitely high RoB | Definitely high RoB | Probably low RoB | Probably high RoB | Definitely high | Probably high | Definitely high |
| Rationale | *No controls* | *Not matched, no influencing factors, no confounding factors taken into account* | *No reports of non-measures* | *Biological matrix, but not a reliable study design* | *No info on the diagnosis method provided* | *No information* |  |
| Onyemelukwe, 1982 (liver disease) | Definitely high RoB | Probably highRoB | Probably low RoB | Probably low RoB | Probably low RoB | Definitely low RoB | Probably low |
| Rationale | *No controls, therefore, no matching* | *No matching, influencing factors (hepatitis B) was taken into account, although not in in- or exclusion criteriad and no confounders taken into account* | *No reports of non-meausres* | *Biological matrix, but not a reliable study design for liver cancer* | *Hospital setting, but not reported how patients were diagnosed* | *Hospital records* |  |
| Liu, 2017 (liver disease) | Probably high | Probably low RoB | Probably high RoB | Probably high Rob | Probably low RoB | Probably low RoB | Probably low |
| Rationale | *No controls, so no matching (community-based)* | *No matching, influencing factors (parasitology & virology) taken into account, also confounding factors taken into account (hepatitis B)* | *Indirect evidence that not all samples were analyzed* | *ELISA in food (+ questionnaires) and a cross-sectional study design for a longitudinal disease* | *Community-based, lacking explicit biomarkers for diagnosis* | *Medical reports* |  |
| Hoque, 1999 (liver disease) | Definitely high | Probably high RoB | Probably low RoB | Probably low RoB | Definitely low RoB | Definitely low RoB | Probably low |
| Rationale | *No controls* | *No matching, ifluencing factors considered (hepatitis b), no confounding factors taken into account in statistics* | *No reports of non-measures* | *Biological matrix, but cross-sectional design* | *Hospital setting* | *Hospital records* |  |
| Hendrickse, 1982 (kwashiorkor) | Probably high RoB | Probably high RoB | Probably high RoB | Probably low RoB | Definitely low RoB | Definitely low RoB | Probably low |
| Rationale | *Age-matched controls* | *Age matched, no other influencing or confounding factors taken into account* | *No info on selections* | *Biological matrix, cross-sectional study* | *Hospital setting (diagnosis criteria well explained)* | *Hospital records* |  |
| Wu, 2022 (liver disease & neurologic disease & metabolic disease) | Definitely high RoB | Probably high RoB | Probably low RoB | Probably low RoB | Probably low RoB | Definitely low RoB | Probably low |
| Rationale | *No controls, case only* | *No matching, no influencing factors, confounding factors (e.g. smoking, diabetes...) were taken into account* | *No reports of non-measures* | *Biological matrix, but cross-sectional study is not appropriate for HCC* | *Hospital setting, no explicit diagnosis criteria* | *Hospital records* |  |
| Hassen, 2004 (kidney disease) | Probably low RoB | Probably high RoB | Probably low RoB | Probably low RoB | Definitely low RoB | Definitely low RoB | Probably low |
| Rationale | *Controls of same age and same region* | *Age-matched, influencing factors (clear distinction between the different groups/disease types), no confounding factors considered* | *No reports of non-measures* | *Biological matrix, but not appropriate study design for chronic disease* | *Hospital setting, nephropathy was diagnosed via glomerular filtration* | *Hospital records* |  |
| Hosny, 2008 (liver disease) | Definitely high RoB | Definitely high RoB | Probably high RoB | Probably low RoB | Definitely low RoB | Definitely low RoB | Probably low |
| Rationale | *No matching* | *Not matched, no influencing factors considered, no confounding factors* | *No info on selection* | *Biological matrix, case-control study without nested design* | *Hospital setting, but no biomarker measurement for diagnosis* | *Hospital records* |  |
| Raafat, 2021 (liver disease & adverse birth outcomes) | Definitely high RoB | Probably high RoB | Definitely high RoB | Definitely low RoB | Definitely low RoB | Definitely low RoB | Probably low |
| Rationale | *No matching (although all neonates)* | *Not matched (although all neonates), influencing factors taken into account, no confounding factors* | *Direct evidence that not all patients were measured* | *Biological matrix and an appropriate study design for an acute disease* | *Hospital setting and biomarker analysis for diagnosis* | *Hospital records* |  |
| Ross, 1992 (liver disease) | Definitely low RoB | Probably low RoB | Probably low RoB | Definitely low RoB | Definitely low RoB | Definitely low RoB | Definitely low |
| Rationale | *Matched for age, gender, disease, ethnicity, etc* | *Matched, influencing factors taken into account, as well as confounders (smoking, hepatitis B, education level, alcohol consumption)* | *No reports of non-measures* | *Biological matrix and nested case-control design (appropriate for HCC)* | *Hospital setting and diagnosed via biopsy/serum alpha-fetoprotein/ultrasonography* | *Hospital records* |  |
| Ruan, 2020 (acute food intoxication) | Definitely low RoB | Probably high RoB | Definitely low RoB | Probably high Rob | Probably high RoB | probably high rob | Definitely high |
| Rationale | *Controls matched for age, gender, other* | *Matched, , no other influencing factors, no confounding factors* | *Case report* | *Food analysis* | *Less severe cases not admitted, not always hospital* | *Only the severe cases with severe symptoms* |  |
| Ouhibi, 2020 (intestinal disease) | Definitely low RoB | Probably low RoB | Probably low RoB | Probably low RoB | Probably low RoB | Definitely low RoB | Definitely low |
| Rationale | *Matched for age, gender & ethnicity* | *Matched, no influencing factors, no confounding factors* | *No reports of non-measures* | *Golden standard, but case-control study without nested design* | *Hospital setting, but no diagnosis criteria mentioned* | *Hospital setting* |  |
| Ilić, 2016 (liver disease) | Definitely high RoB | Probably high RoB | Definitely low RoB | Probably low RoB | Definitely low RoB | Definitely low RoB | Probably low |
| Rationale | *No controls, so no matching* | *Not matched, influencing factorstaken into account (HBV, HCV, liver cirrhosis), no confounding factors* | *Case report* | *Biological matrix, cross-sectional, not appropriate for HCC* | *Hospital setting, HCC was diagnosed via radiography* | *Hospital setting* |  |
| Serck-Hanssen, 1970 (liver disease) | Definitely high RoB | Definitely high RoB | Definitely low RoB | Probably high Rob | Definitely low RoB | Definitely low RoB | Probably high |
| Rationale | *No controls, no matching* | *No matching, no influencing factors, no confounding factors* | *Case report* | *Food analysis* | *Hospital setting, full examination* | *Hospital records* |  |
| Bessard, 1990 (liver disease) | Definitely high RoB | Definitely high RoB | Definitely low RoB | Probably low RoB | Definitely high RoB | Definitely high RoB | Definitely high |
| Rationale | *No controls, no matching* | *No matching, influencing factors taken into account, no confounding factors* | *Case report* | *Biological matrix* | *No info on diagnosis* | *No info on diagnosis* |  |
| Chao, 1991 (outcome not found - check) | Definitely high RoB | Probably high RoB | Definitely low RoB | Definitely low RoB | Probably low RoB | Probably low RoB | Probably low |
| Rationale | *Outbreak report* | *No matching, s, influencing factors (explicit exclusion criteria), no confounding factors* | *Outbreak report* | *Biological matrix, appropriate study design for aflatoxicosis* | *Less severe cases not admitted, not always hospital, but explicit diagnostic criteria* | *Only the severe cases with severe symptoms but comprehensive recording* |  |
| Wang, 1993 (acute food intoxication) | Definitely high RoB | Probably high RoB | Definitely low RoB | Definitely high Rob | Definitely high RoB | Probably high RoB | Definitely high |
| Rationale | *No controls, so no matching (no information as well)* | *No info on measurement* | *Case report* | *No info on measurement technique and only in molded food* | *No info on diagnosis* | *Only recording of symptoms* |  |
| Stora, 1987 (liver disease) | Definitely high RoB | Probably low RoB | Definitely low RoB | Probably low RoB | Definitely low RoB | Definitely low RoB | Definitely low |
| Rationale | *Controls, but not matched* | *Not matched, influencing factors accounted (hepatitis, cirrhosis...), confounding factors also taken into account* | *Case report* | *Biological matrix, but not appropriate study design for liver cancer* | *Hospital setting* | *Hospital records* |  |
| Lye, 1995 (acute food intoxication) | Definitely high RoB | Definitely high RoB | Definitely low RoB | Probably high Rob | Definitely high RoB | Definitely high RoB | Definitely high |
| Rationale | *No information on the controls* | *,Not matched, no influencing or confounding factors accounted* | *Case report* | *Biological matrix, but very little info* | *No info on diagnosis* | *No recording* |  |
| Long, 2008 (liver disease) | Definitely high RoB | Probably low RoB | Probably low RoB | Probably low RoB | Definitely low RoB | Definitely low RoB | Definitely low |
| Rationale | *No controls, so no matching* | *Not matched, influencing factors accounted, confounding factors as well (age, sex, ethnicity, HBsAg, anti-HCV, AFB1-adducts)* | *Patients excluded with documented reason* | *Biological matrix* | *Hospital setting, histopathological diagnosis* | *Hospital records* |  |
| NjumbeEdiage, 2013 (underweight & stunting) | Probably high RoB | Probably high RoB | Probably low RoB | Definitely low RoB | Probably low RoB | probably low RoB | Probably low |
| Rationale | *No controls (but age and population are comparable in this exposure assessment)* | *No matching, influencing factors accounted (clear exclusion critera), no confounders taken into account* | *Patients excluded with documented reason* | *Biological matrix, cross sectional study* | *Nurses personal on-site, clear criteria for diagnosis* | *Medical records* |  |
| Mandishona, 1998 (liver disease) | Definitely low RoB | Probably low RoB | Definitely low RoB | Probably low RoB | Definitely low RoB | Definitely low RoB | Definitely low |
| Rationale | *Age, gender and etchnicity matched* | *Matching, taken into account influencing factor, and confounding factors (e.g. alcohol consumption)* | *All included cases were measured* | *Biological matrix, cross-sectional study design for HCC not appropriate* | *Hospital setting, liver biopsies for diagnosis* | *Hospital records* |  |
| Desalegn, 2011 (kidney disease) | Definitely high RoB | Probably high RoB | Probably high RoB | Probably low RoB | Probably high RoB | Definitely high RoB | Probably high |
| Rationale | *No matching* | *No matching, influencing factors were taken into account (diabetes, hypertension...), no confounding factors* | *Not all cases were measured, random selection* | *Biological matrix, but cross-sectional design for a chronic disease is not appropriate* | *Medical records (with criteria following KDOQI guidelines, but lacking explicit confirmation* | *No info on recording* |  |
| Xu, 2021 (immunomodulation) | Definitely low RoB | Probably low RoB | Probably low RoB | Definitely low RoB | Probably low RoB | Probably low RoB | Definitely low |
| Rationale | *Matched for age, gender, ethnicity, medication use* | *Matching, influencing factors taken into account and adjusted for confounding factors* | *No reports of non-measures* | *Biological matrix, and appropriate longitudinal study design* | *Trained personnel, laboratory results, but also questionnaires on-site* | *Indirect proof that all outcomes were documented* |  |
| Persson, 2012 (liver disease) | Definitely low RoB | Definitely low RoB | Probably low RoB | Definitely low RoB | Definitely low RoB | Probably low RoB | Definitely low |
| Rationale | *Matched for age, gender, origin* | *Matching, influencing factors taken into account, adjusted for confounding factors (hepatitis B virus, alcohol consumption)* | *Patients excluded with documented reason* | *Biological matrix + appropriate study design* | *Hospital records, and HCC diagnosis via liver imaging, or alpha-fetoprotein elevation* | *Indirect proof that all outcomes were documented* |  |
| Duringer, 2016 (neurologic disease) | Probably low RoB | Definitely low RoB | Probably low RoB | Probably low RoB | Definitely low RoB | Probably low RoB | Definitely low |
| Rationale | *Matched for age and region* | *Matched, influencing factors considered, adjusted for confounders (e.g. medication use)* | *No report of non-measured samples* | *Biological matrix, but case-control without nested design* | *Autism clinical program at rehabilitation centre, diagnosis via DSM-IV criteria* | *Indirect proof that all outcomes were documented* |  |
| Sun, 2001 (liver disease) | Definitely low RoB | Probably low RoB | Definitely low RoB | Definitely low RoB | Definitely low RoB | Probably low RoB | Definitely low |
| Rationale | *Matched for age, gender, and residence* | *Matched, influencing factors taken into account and adjusted for confounding factors* | *Missing samples are explained* | *Biological measurement and nested case-control study (appropriate design)* | *Diagnosis was done in medical centres by tomography/angiogram/cytology* | *indirect proof that all outcomes were documented* |  |
| MohdRedzwan, 2014 (kidney disease & liver disease) | Definitely high RoB | Definitely high RoB | Probably high RoB | Definitely low RoB | Probably high RoB | Probably high RoB | Probably high |
| Rationale | *No controls, so no matching* | *No matching, noinfluencing factors taken into account, no confounding factors taken into account* | *Indirect evidence of non-measures* | *Biological measurement , cross-sectional design appropriate* | *Non-medical personel on-site* | *Self-questionnaire* |  |
| Kimbi, 2005 (liver disease) | Definitely high RoB | Probably high RoB | Probably high RoB | Probably low RoB | Definitely low RoB | Probably low RoB | Probably low |
| Rationale | *No matching* | *No matching, no influencing factors taken into account, but adjusted for confounding factors (e.g. age, HBsAg status)* | *No info on selection* | *Aflatoxin is not measured (genetic mutation is measured in DNA), but cross-sectional design for HCC not appropriate* | *Hospital setting* | *Medical records* |  |
| Lunn, 1997 (liver disease) | Probably low RoB | Probably high RoB | Probably high RoB | Probably low RoB | Definitely low RoB | Definitly low RoB | probably low |
| Rationale | *Matching of age and gender* | *Matching, no influencing factors taken into account, not adjusted for confounding* | *No info on selection* | *Biological measurement, inappropriate study design* | *Hospital setting (biochemistry/biopsies/imaging)* | *Hospital records* |  |
| Aydin, 2015 (liver disease) | Probably low RoB | Probably low RoB | Definitely low RoB | Probably low RoB | Definitely low RoB | Definitely low RoB | Definitely low |
| Rationale | *same population, in same age range, not matched for gender* | *not fully matched, influencing factors accounted for during selection, adjusted for confounding (smoking/alcohol)* | *Selection criteria well-documented* | *Biological measurement, inappropriate study design* | *Hospital setting (biochemistry/imaging)* | *Hospital records* |  |
| Claeys, 2022 (kidney disease) | Definitely high RoB | Probably high RoB | Definitely low RoB | Probably low RoB | Definitely high RoB | Probably low RoB | probably high |
| Rationale | *Direct evidence that controls and cases were not similar* | *No matching, no influencing factors taken into account, adjusted for confounding (alcohol, BMI, etc)* | *Selection criteria well-documented* | *Food questionnaires, appropriate study design* | *No info on diagnosis* | *Questionnaires by trained personel* |  |
| Dinis, 2007 (kidney disease) | definitely high RoB | definitely high RoB | Probably high RoB | defnitely low RoB | Definitely high RoB | Probably high RoB | Probably high |
| Rationale | *no controls* | *not matched, no influencing factors accounted for, not adjusted for confounding* | *Indirect evidence that exclusion was not addressed* | *Biological measurement, appropriate study design* | *No info on diagnosis* | *Indirect proof that not all outcomes were documented* |  |
| Huong, 2019 (stunting) | definitely high RoB | Probably high RoB | Probably high RoB | Probably low RoB | Definitely high RoB | Probably high RoB | Probably high |
| Rationale | *No controls* | *no matching, no influencing factors accounted for, adjusted for confounding* | *Indirect evidence that exclusion was not addressed* | *Biological measurement, inappropriate study design* | *No info on diagnosis* | *Only recording of symptoms* |  |
| Hollstein, 1993 (liver disease) | definitely high RoB | Probably high RoB | Probably low RoB | Probably low RoB | Definitely high RoB | Definitely high RoB | probably high |
| Rationale | *Case report – no controls* | *no matching, no influencing factors accounted for, adjusted for hepatitis B antigen* | *Case report* | *Biological measurement, inappropriate study design* | *No info on diagnosis* | *No info recorded* |  |
| Oyelami, 1998 (gastric disease) | definitely high RoB | definitely high RoB | Probably high RoB | Probably low RoB | Definitely low RoB | Definitely high RoB | Definitely high |
| Rationale | *No controls* | *no matching, no influencing factors, not adjusted* | *Selection not mentioned* | *Biological measurement, inappropriate study design* | *Hospital setting (biochemistry/imaging)* | *No info on diagnosis recorded* |  |
| Ankrah, 1994 (liver disease) | definitely high RoB | probably low RoB | Probably high RoB | probably low RoB | Definitely high RoB | Definitely high RoB | probably low |
| Rationale | *No controls* | *no matching, influencing factors accounted for during selection (alcohol consumption), adjusted for confounding* | *Selection not mentioned* | *biological measurement, inappropriate study design* | *No info on diagnosis* | *No info on diagnosis* |  |
| Xie, 2018 (metabolic disease) | Definitely high RoB | Probably high RoB | Probably low RoB | Definitely low RoB | Definitely low RoB | Definitely low RoB | Probably low |
| Rationale | *Not matched* | *Not matched, influencing factors considered but not adjustment for covairates* | *No info on non-measures* | *Biological measurement and appropriate study design* | *Hospital setting with clinical paramteres mentioned* | *Hospital records* |  |
| Akash, 2021 (metabolic disease) | Defintely high RoB | Probably high RoB | Definitely low RoB | Probably low RoB | Definitely low RoB | Definitely low RoB | Probably low |
| Rationale | *Not matching* | *Not matched, influencing factors considered but no matching of covariates* | *non-measures adequately addressed* | *Biological measurement but no appropriate study design* | *Hospital setting with preliminary screening test performed by medical doctors* | *Hospital records and questionnaires* |  |
| Abdel-Wahab, 2008 (liver disease) | Definitely low RoB | Probably low RoB | Probably low RoB | Probably low RoB | Definitely low RoB | Definitely low RoB | Definitely low |
| Rationale | *Matched for age, gender, ethnicity, etc.* | *Matched, influencing factors considered but not adjusted for covariates* | *Selection explained* | *Biological measurement but no appropriate study design* | *Hospital setting with clinical parameters, ultrasound diagnosis and biomeasure analysis* | *Hospital records* |  |
| Kirk, 2005 (liver disease) | Probably low RoB | Definitely lowRoB | Probably low RoB | Probably low RoB | Definitely low RoB | Probably high RoB | Definitely low |
| Rationale | *Matched for age, gender* | *Matched, influencing factors accounted for, and adjusted for appropriate covariates (e.g., groundnut intake)* | *No info on non-measures* | *Biological measurement but no appropriate study design* | *Hospital setting + diagnosis via ultrasound and alfa-fetoprotein* | *Missing info* |  |
| Bercroft, 1972 (neurologic disease) | Definitely high RoB | Definitely high RoB | Definitely low RoB | Definitely low RoB | Definitely low RoB | Definitely low RoB | Probably low |
| Rationale | *No controls* | *No controls, no influencing factors nor covariates adjusted for* | *Case report* | *Biological measurement and appropriate study design* | *Hospital setting with clinical symptoms* | *Hospital records* |  |
| Deng, 2012 (premature puberty) | Definitely low RoB | Probably low RoB | Probably low RoB | Probably low RoB | Definitely low RoB | Definitely low RoB | Definitely low |
| Rationale | *Matched for age, gender with no secondary sex characteristics recruited at controls* | *Matched controls, influencing factors but no covariates adjusted* | *No info on non-measures* | *Biological measurement but no appropriate study design* | *Within the hospital and all patients underwent abdominal ultrasonography* | *Hospital record* |  |
| Kamala, 2018 (acute food intoxication & underweight) | Definitely high RoB | Definitely high RoB | Probably low RoB | Probably low RoB | Definitely high RoB | Definitely high RoB | Probably high |
| Rationale | *Unmatched* | *Unmatched, no influencing factors and not adjusted for covariates* | *No info on non-measures* | *Food but RCT* | *No info on diagnosis* | *Nothing recorded* |  |
| Lauer, 2020 (underweight) | Definitely high RoB | Probably low RoB | Probably low RoB | Defintely low RoB | Definitely low RoB | Probably high RoB | Probably low |
| Rationale | *No controls* | *No controls, influencing factors accounted for, adjusted for appropriate covariates (gestational weight)* | *No info on non-measures* | *Biological measurement and appropriate study design* | *Hospital setting using WHO standards* | *Although in hospital, little recorded* |  |
| Chen, 2007 (liver disease) | Probably low RoB | Probably high RoB | Definitely high RoB | Probably low RoB | Definitely low RoB | Definitely low RoB | Probably low |
| Rationale | *Matched for age and gender* | *Not fully matched, influencing factors considered and no adjustment of covariates* | *Direct evidence that non-measures were not addressed* | *Biological measurement but no appropriate study design (CaCo but not nested)* | *Hospital setting using a scoring system of liver parenchyma and ultrasonography* | *Hospital records + clinical features* |  |
| Mitchell, 2017 (stunting) | Definitely high RoB | Probably low RoB | Probably low RoB | Definitely low RoB | Definitely low RoB | Probably high RoB | Probably low |
| Rationale | *No controls* | *No matched controls, influencing factors accounted and adjusted for appropriate covariates (e.g., total energy)* | *No info on non-measures* | *Biological measurement and appropriate study design* | *Hospital setting and using WHO standards* | *Although in hospital, very little recorded* |  |
| Probst, 2007 (acute food intoxication) | Definitely high RoB | Probably high RoB | Definitely low RoB | Probably high RoB | Definitely high RoB | Definitely high RoB | Definitely high |
| Rationale | *Case report* | *No controls, influencing factors included but no covariate adjustment* | *Case report* | *Food analysis and appropriate study design for acute aflatoxicosis* | *No info on diagnosis* | *No info recorded* |  |
| McGlynn, 1995 (liver disease) | Probably high RoB | Probably high RoB | Probably low RoB | Probably low RoB | Probably low RoB | Probably low RoB | Probably low |
| Rationale | *Not matched for age, gender* | *Not fully matched, some influencing factors accounted for, but no covariates adjusted* | *No info on non-measures* | *Biological measurement but no appropriate study design* | *Hospital setting with genotyping* | *Hospital records* |  |
| Ozdemir, 2010 (liver disease) | Probably high RoB | Probably high RoB | Probably low RoB | Probably low RoB | Definitely low RoB | Definitely low RoB | Probably low |
| Rationale | *Not matched for age, gender* | *Not fully matched, some influencing factors but no adjustment of covariates* | *No info on non-measures* | *Biological measurement but no appropriate study design (CaCo but not nested)* | *Hospital setting and* cases of HCC included compatible clinical and radiologic findings, serum AFP; Alpha-fetoprotein (a-fetoprotein) levels, and finally, biopsy. The diagnosis of cirrhosis and chronic hepatitis B was also based on compatible clinical history, serologic markers, and liver biopsy findings. Fifty cases of controls were recruited from blood donors | *Diagnosis info and hospital record* |  |
| Deng, 1998 (liver disease) | Definitely high RoB | Definitely high RoB | Probably low RoB | Definitely low RoB | Definitely low RoB | Definitely low RoB | Probably low |
| Rationale | *No controls* | *No matched controls, no influencing factors and no covariate adjustment* | *No info on non-measures* | *Biological measurement and appropriate study design* | *Hospital setting with questionnaires, clinical parameters and lab analyses* | *Medical reports* |  |
| Kareem, 2021 (kidney disease) | Definitely high RoB | Probably low RoB | Probably low RoB | Probably low RoB | Definitely low RoB | Definitely low RoB | Definitely low |
| Rationale | *No matching* | *Not matching, influencing factors considered and adjusted for covariates (e.g., BMI, diabetes)* | *No info on non-measures* | *Biological measurement (golden standard) but no appropriate study design (CaCo but not nested)* | *Hospital setting with medical examination* | *Hospital records* |  |
| Ng, 1994 (liver disease) | Definitely high RoB | Probably low RoB | Probably low RoB | Probably low RoB | Definitely low RoB | Definitely low RoB | Definitely low |
| Rationale | *No controls* | *No matched controls, influencing factors considered and no adjustment of covariates* | *One post-mortem sample, otherwise no info on non-measures* | *Biological measurement and appropriate study design* | *Hospital setting with lab analyses* | *Hospital records* |  |
| Ozturk, 1991 (liver disease) | Defintely high RoB | Probably high RoB | Probably low RoB | Probably low RoB | Definitely high RoB | Definitely high RoB | Probably high |
| Rationale | *No controls* | *No matched controls, influencing factors accounted, no covariates adjusted* | *No info on non-measures* | *Biological measurement but no appropriate study design* | *No info on diagnosis* | *No info on diagnosis* |  |
| Fahmy, 2014 (kidney disease & male reproductive disease) | Definitely low RoB | Probably low RoB | Probably low RoB | Defintely low RoB | Probably low RoB | Probably low RoB | Definitely low |
| Rationale | *Normal tissue from the negative surgical margins of each tumour served as a control* | *Control and case sample from the same participant, influencing factors considered but no adjustment of covariates* | *No info on non-measures* | *Biological measure and appropriate study design* | *Probably hospital, diagnosis via biopsy and histopathological* | *Info on biopsy* |  |
| Niknejad, 2021 (oesophageal disease) | Definitely high RoB | Definitely high RoB | Probably low RoB | Probably low RoB | Definitely high RoB | Definitely high RoB | Definitely high |
| Rationale | *No matching* | *No matching, no influencing factors and no covariates included* | *No info on non-measures* | *Biological measurement but no appropriate study design (CaCo but not nested)* | *No info on diagnosis – pilot study method paper* | *No info on diagnosis – pilot study method paper* |  |
| Nelson, 1980 (neurologic disease) | Definitely low RoB | Probably high RoB | Probably low RoB | Probably low RoB | Definitely low RoB | Definitely low RoB | Probably low |
| Rationale | *Matched for age, gender & ethnicity and residence* | *Matching, no influencing factors nor covariates considered* | *Non-measures adequately addressed* | *Biological measurement but no appropriate study design* | *Hospital setting with clinical parameters and diagnosis mentioned* | *Hospital records* |  |
| Ortiz-Cuaran, 2013 (liver disease) | Definitely low RoB | Definitely low RoB | Probably low RoB | Probably low RoB | Definitely low RoB | Definitely low RoB | Definitely low |
| Rationale | *Matched for age, gender & ethnicity* | *Matching, influencing factors accounted for and adjusted for appropriate covariates (e.g., hepatitis antibodies)* | *No info on non-measures* | *Biological measurement but no appropriate study design (CaCo but not nested)* | Hospital setting. Differential diagnosis of HCC and cholangiocarcinoma was based on clinical examination, alpha-fetoprotein (AFP) detection and abdominal imaging, with histological confirmation in a subset of the cases. Presence of cirrhosis at HCC diagnosis was assessed by imaging and was combined with information on symptom duration and on pre-existing clinical information to classify HCC patients into two groups, HCC/cirrhosis (with clinical and imaging evidence of cirrhosis) and HCC/no cirrhosis (with no evidence of cirrhosis at diagnosis or prior to the onset of first tumour symptoms). | *Hospital records* |  |
| Mizrak, 2009 (liver disease) | Probably high RoB | Probably high RoB | Probably low RoB | Definitely low RoB | Definitely low RoB | Definitely low RoB | Probably low |
| Rationale | *No info on controls* | *No matching, no influencing factors nor covariates adjusted for* | *No info on non-measures* | *Biological measurement with appropriate study design* | *Hospital setting with viral hepatitis levels* | *Hospital records* |  |
| Alvarez, 2022 (metabolic disease) | Probably high RoB | Probably low RoB | Definitely low RoB | Probably low RoB | Probably low RoB | Probably low RoB | Definitely low |
| Rationale | *Controls were selected based on the residence of cases* | *Control selection only on residence, influencing factors accounted for and adjustment for appropriate covariates (e.g., alcohol, hepatitis)* | *Non-measure adequately addressed* | *Biological measurement but no appropriate study design (CaCo but not nested)* | *Hospital setting and cirrhosis was diagnosed by abdominal ultrasonography using a quantitative scoring system, including morphological appearance of the liver surface, liver parenchymal texture, intrahepatic vascular structure and spleen size.* | *Medical records – trained personnel* |  |
| Wangia-Dixon, 2020 (underweight) | Definitely high RoB | Probably low RoB | Probably low RoB | Definitely low RoB | Probably low RoB | Probably low RoB | Definitely low |
| Rationale | *No controls* | *No controls, influencing factors accounted for and appropriate covariates adjustment (e.g., material status, education of mother)* | *No info on non-measures* | *Biological measurement and appropriate study design* | *Trained personnel on-site using WHO standards* | *Medical records/questionnaires* |  |
| Lin, 2020 (oesophageal disease) | Probably high RoB | Probably high RoB | Probably low RoB | *Definitely low RoB* | *Definitely low RoB* | *Definitely low RoB* | Prbably low |
| Rationale | *No info on controls* | *No matching, no influencing factors considered but adjustment for appropriate covariates (e.g., age and sex)* | *No info on non-measures* | *Biological measurement and appropriate study design* | *Hospital setting with the diagnosis of achalasia was based on the absence of peristalsis and the presence of impaired LES relaxation on established tests (barium swallow, manometry or esophagogastroduodenoscopy).* | *Hospital records including clinical and diagnostic information* |  |
| Hsieh, 2004 (kidney disease & metabolic disease) | Definitely high | Probably high RoB | Probably low RoB | Defintely low RoB | Probably high RoB | Definitely high RoB | Probably high |
| Rationale | *No controls* | *No controls, influencing factors considered but no adjustment of covariates* | *No info on non-measures* | *Biological measurement and appropriate study design* | *Info on biomarker analyses for outcome measure* | *No info recorded* |  |
| Shirabe, 2011 (liver disease) | Definitely high | Probably high RoB | Definitely low RoB | Definitely low RoB | Probably low RoB | Definitely low RoB | Probably low |
| Rationale | *No controls* | *No controls, influencing factors accounted for but no covariates* | *Exclusion adequately addressed* | *Biological measurement and appropriate prospective design* | *Hospital setting but lacks info on diagnosis criteria* | *Hospital records with clinicopathological characteristics* |  |
| Rivera-Nunez, 2019 (premature puberty) | Definitely high | Probably low RoB | Probably high RoB | Definitely low RoB | Probably high RoB | Probably low RoB | Probably low |
| Rationale | *No controls* | *No matched controls, accounted for influencing factors, adjusted for appropriate (e.g., BMI at menarche)* | *Not adequately addressed* | *Biological measurement (Golden standard) and appropriate study design* | *Trained personnel on-site but lacks info on criteria* | *Medical records/questionnaires* |  |
| Berry, 1986 (neurologic disease) | Definitely high | Probably high RoB | Probably high RoB | Probably low RoB | Definitely low RoB | Definitely low RoB | Probably low |
| Rationale | *No controls* | *No controls, influencing factors but not adjusted for covariates* | *Not adequately addressed* | *Food analysis and appropriate study design* | *Hospital setting with clinical symptoms* | *Hospital records* |  |
| Lei, 2021 (blood diseases) | Definitely high | Probably low RoB | Definitly low RoB | Definitely low RoB | Definitely low RoB | Definitely low RoB | Definitely low |
| Rationale | *No controls* | *No matched controls, adjusted for influencing factors and adjusted for appropriate covariates (e.g., maternal age, folic acid)* | *Adequately addressed reasons for exclusion* | *Biological measurement and appropriate study design* | *Hospital setting using WHO Child growth standards* | *Hospital records* |  |
| Mahfuz, 2022 (neurologic disease) | Definitly high RoB | Probably high RoB | Definitely low RoB | Definitely low RoB | Probably low RoB | Probably low RoB | Definitely low |
| Rationale | *No controls* | *No controls, accounted for influencing factors but not adjusted for covariates* | *Adequately addressed, with all cases measured* | *Biological measurement (golden standard) and appropriate study design* | *Trained personnel on-site using WHO Child growth standards* | *Medical records/questionnaires* |  |
| Wu, 2009 (liver disease) | Definitely low RoB | Probably low RoB | Definitely low RoB | Definitely low RoB | Definitely low RoB | Probably low RoB | Definitely low |
| Rationale | *Matched for age, gender & ethnicity* | *Matched design, influencing factors accounted for and adjusted for appropriate covariates (e.g., alcohol, hepatitis)* | *Exclusion adequately addressed* | *Biological measurement and appropriate study design* | *Hospital setting, with suspected HCC cases were referred to teaching medical enters for confirmatory diagnosis by computerized tomography, digital subtracted angiogram, aspiration cytology, and pathologic examination. The criteria for HCC diagnosis included a histopathologic examination and a positive lesion detected by at least two different imaging techniques (abdominal ultrasonography, angiogram, or computed tomography) or by one imaging technique and a serum AFP level of >400 ng/mL* | *Reported on liver biomarkers* |  |
| Mahfuz, 2021 (stunting) | Definitely high | Probably low RoB | Probably low RoB | Definitely low RoB | Probably low RoB | Probably low RoB | Definitely low |
| Rationale | *No controls* | *No controls, influencing factors accounted for, adjusted for appropriate covariates* | *loss of follow up explained* | *Biological measurement (golden standard) and appropriate study design* | *Trained personnel on-site using WHO standards* | *Medical records/questionnaires* |  |
| Abdulrazzaq, 2002 (adverse birth outcomes) | Definitely high RoB | Definitely high RoB | Probably high RoB | Definitely low RoB | Probably low RoB | Probably low RoB | Probably low |
| Rationale | *No controls* | *No golden standard, no influencing factors and not adjusted for covariates* | *Not Adequately addressed* | *Biological measurement and appropriate study design* | *Hospital setting but lacks info on outcome* | *Hospital records* |  |
| Chan, 1990 (liver disease) | Probably high RoB | Definitely high RoB | Probably high RoB | Probably high RoB | Definitely high RoB | Definitely high RoB | Definitely high |
| Rationale | *Matched for ethnicity only* | *Not fully matched, no influence factors and not adjusted for covariates* | *Not Adequately addressed* | *Food analysis but no info on method, no appropriate study design for cancer* | *No info on diagnosis* | *No info on diagnosis* |  |
| Hendrickse, 1984 (kwashiorkor) | Probably high RoB | Definitely high RoB | Probably high RoB | Probably low RoB | Definitely low RoB | Definitely low RoB | Probably low |
| Rationale | *Matched for age only* | *Not fully matched, no influencing factors, no covariates included* | *Not Adequately addressed* | *Biological measurement but no longitudinal design* | *Hospital setting using WHO standards* | *Hospital records* |  |
| Yadgiri, 1970 (liver disease) | Definitely high RoB | Definitely high RoB | Definitely low RoB | Probably low RoB | Definitely low RoB | Definitely low RoB | Probably low |
| Rationale | *Case series* | *No matched controls, no influence factors nor covariates considered* | *Case series* | *Biological measurement but no appropriate study design* | *Hospital setting with histological proven liver cirrhosis* | *Hospital records* |  |
| DeSantis, 2017 (neurologic disease) | Probably high RoB | Probably high RoB | Definitely low RoB | Probably low RoB | Probably low RoB | Probably low RoB | Probably low |
| Rationale | *Matched for age only* | *No full matching, no influencing factors nor covariates considered* | *Exclusion adequately addressed* | *Biological measurement (golden standard) but no appropriate study design (CaCo but not nested)* | *Trained personnel on-site, clinical symtopmps, chemokines and biomarker analyses mentioned* | *Medical records/questionnaires* |  |
| Cirlini, 2016 (intestinal disease) | Probably low RoB | Probably high RoB | Probably low RoB | Definitely low RoB | Probably low Rob | Definitely high Rob | Probably low |
| Rationale | *Controls had similar energy intake, sex, BMI and age* | *Matching, no influencing factors, no covariates adjusted* | *Exclusion explained* | *Biological measurement (golden standard) and appropriate study design* | *Diagnosis was conducted at Centre* for Prevention and Diagnosis of Celiac Disease at the University of Milan. | *No info reported* |  |
| Wood, 2017 (immunomodulation) | Probably high RoB | Definitely high RoB | Probably high RoB | Probably low RoB | Definitely low RoB | Definitely low RoB | Probably low |
| Rationale | *No info on controls* | *No matching, no influencing factors, no covariates* | *Not Adequately addressed* | *Biological measurement but no appropriate study design* | *Hospital setting with assessment of T cell activation, cytokines and chemokines* | *Hospital records* |  |
| Amla, 1971 (liver disease) | definitely high | Probably high RoB | Definitely low RoB | Definitely low RoB | Definitely low RoB | Definitely low RoB | Probably low |
| Rationale | *Case series* | *No matched controls, little influencing factors (focus on peanuts) considered and no covariates adjusted for* | *Case series* | *Biological measurement and ideal study design* | *Hospital setting with liver histology mentioned* | *Hospital record* |  |
| Yordanova, 2010 (kidney disease) | Probably high RoB | Probably high RoB | Probably high RoB | Probably low RoB | Definitely high Rob | Definitely high Rob | Definitely high |
| Rationale | *Not matched for age or gender, but only for origin* | *Not matched, influencing factors accounted for and no covariates considered* | *Not Adequately addressed* | *Biological measurement but no appropriate study design* | *No info on diagnosis* | *No info on diagnosis* |  |
| HmaissiaKhlifa , 2012 (kidney disease) | Probably high RoB | Definitely high RoB | Probably high RoB | Probably low RoB | Probably low RoB | Probably high Rob | Probably high |
| Rationale | *No info on controls* | *No matching design, no influencing factors and not adjustment* | *Not Adequately addressed* | *Biological measurement but no appropriate study design* | *Hospital setting but no info on diagnosis* | *Although in hospital, very little info recorded* |  |
| Gadzała-Kopciuch, 2011 (female reproductive disease) | definitely high | Definitely high RoB | Probably high RoB | Probably low RoB | Definitely high RoB | Definitely high RoB | Definitely high |
| Rationale | *No controls* | *No matched controls, no influencing factors and no covariates considered* | *Not Adequately addressed* | *Biological measurement but no appropriate study design* | *No info on hospital nor diagnosis* | *No info* |  |
| Turner, 2007 (underweight & stunting) | *Definitely high RoB* | *Definitely high RoB* | *Probably high RoB* | *Definitely low RoB* | *Probably low RoB* | *Probably low RoB* | *Definitely low* |
| Rationale | *No controls* | *No matched controls, influence factors considered and appropriate covariates (e.g., placental weight) adjusted for* | *Not Adequately addressed* | *Biological measurement and appropriate study design* | *Hospital and WHO standards* | *Medical reports* |  |
| Gajecki, 2004 (female reproductive disease) | definitely high RoB | Definitely high RoB | Probably high RoB | Definitely low RoB | Definitely high Rob | Definitely high Rob | Probably high |
| Rationale | *No controls* | *No matched controls, no influencing factors and no consideration of covariates* | *Not Adequately addressed* | *Biological measurement and appropriate study design* | *No info on diagnosis* | *No info recorded* |  |
| Sun, 1999 (liver disease) | definitely high RoB | Probably high RoB | Probably high RoB | Probably low RoB | Definitely low RoB | Probably high Rob | Probably high |
| Rationale | *No controls* | *No matched controls, Influencing factors considered and no covariates considered* | *Not Adequately addressed* | *Biological measurement but no appropriate study design* | *Hospital setting and info on diagnosis* | *Although in hospital, very little info recorded* |  |
| El-Shahat, 2012 (liver disease) | Probably low RoB | Probably high RoB | Probably high RoB | Probably low RoB | Definitely high Rob | Definitely high Rob | Probably high |
| Rationale | *Matched for age, gender* | *Not fully matched, no influencing factors considered and no adjustment of covariates* | *Not Adequately addressed* | *Biological measurement but no appropriate study design* | *No info on diagnosis* | *No info on diagnosis* |  |
| Parkin, 1991 (liver disease) | Probably low RoB | Probably low RoB | Probably low RoB | Probably low RoB | Definitely low RoB | Definitely low RoB | Definitely low |
| Rationale | *Matched for age, gender and origin (but different inclusion criteria)* | *Matched, accounted for influencing factors but no adjustment of covariates* | *Adequately addressed* | *Biological measurement but no appropriate study design (CaCo but not nested)* | *Hospital setting and c*holangiocarcinoma diagnosed either by histology, or with typical findings on ultrasound examination or by percutaneous cholangiography with or without an elevated titre (5=40 units/ml) of CA19-9 | *Hospital records* |  |
| Chen, 2022 (blood diseases) | Definitely high RoB | Definitely low RoB | Probably low RoB | Probably low RoB | Probably low RoB | Probably low RoB | Definitely low |
| Rationale | *No controls* | *No matched controls, accounted for influencing factors and adjusted for appropriate covariates (e.g., alcohol during pregnancy, BMI)* | *adequately addressed* | *Biological measurement and appropriate study design* | *Trained personnel and laboratory testing for outcome* | *Medical records* |  |
| Lu, 2010 (liver disease) | Definitely high RoB | Definitely high RoB | Probably high RoB | Definitely low RoB | Probably low RoB | Definitely high Rob | Probably low |
| Rationale | *No controls* | *No matched controls, no influencing factors nor adjusted for covariates* | *Not Adequately addressed* | *Biological measurement and appropriate study design* | *Hospital setting but lacks info on diagnostic* | *No info* |  |
| Ahmed, 1995 (liver disease) | Probably low RoB | Probably high RoB | Probably high RoB | Definitely low RoB | Definitely low RoB | Probably low Rob | Probably low |
| Rationale | *Matched for age, gender* | *Not fully matched, influencing factors considered but no covariates considered* | *Not Adequately addressed* | *Biological measurement (golden standard) and appropriate study design* | *Hospital setting and info on clinical diagnosis* | *Hospital records* |  |
| Soini, 1996 (liver disease) | Definitely high RoB | Probably low RoB | Probably high RoB | Probably low RoB | Probably low RoB | Probably low RoB | Probably low |
| Rationale | *No controls* | *No matched design, influencing factors accounted for and no info on covariates* | *Not Adequately addressed* | *Biological measurement but no appropriate study design* | *Hospital setting but lacks info on diagnosis* | *Hospital records* |  |
| Lieonart, 2005 (liver disease) | Definitely low RoB | Definitely low RoB | Probably high RoB | Definitely low RoB | Definitely low RoB | Definitely low RoB | Definitely low |
| Rationale | *Matched for age, gender, ethnicity* | *Matched, golden standard, influencing factors accounted for and adjusted for covariates (e.g., alcohol)* | *Not Adequately addressed* | *Biological measurement (golden standard) but no appropriate study design (Caco but not nested)* | *Hospital setting. Evaluation of all participants included a detailed interviewer-administered questionnaire, a physical exam, collection of biological specimens, and a standardized ultrasound examination in the case of hepatocellular carcinoma and cirrhotic participants.* | *Hospital records* |  |
| Matchado, 2023 (wasting & underweight & stunting & adverse birth outcomes) | Definitely high RoB | Definitely low RoB | Probably low RoB | Definitely low RoB | Definitely low RoB | Probably low RoB | Definitely low |
| Rationale | *No controls* | *No matched design, influencing factors accounted for, not golden standard and appropriate covariates considered (e.g., pregnancy BMI)* | *Adequately addressed* | *Biological measurement and appropriate study design* | *Sub study from hospital with WHO standards* | *Medical records* |  |
| Kosicki, 2021 (kidney disease) | Probably low RoB | Probably high RoB | Probably high RoB | Probably low RoB | Probably low RoB | Probably high Rob | Probably low |
| Rationale | *No statistical difference between two groups* | *No difference control versus patients, no influencing factors accounted for and not adjusted for covariates* | *Not Adequately addressed* | *Biological measurement but no appropriate study design* | *Medical centre but no more information* | *Little info reported* |  |
| Zheng, 2017 (liver disease) | Definitely low RoB | Definitely low RoB | Probably high RoB | Probably low RoB | Definitely high Rob | Probably high RoB | Probably high |
| Rationale | *Matched for age, gender, origin* | *Matched, accounted for influencing factors and adjusted for appropriate covariates (e.g., alcohol)* | *Not Adequately addressed* | *Biological measurement but no appropriate study design* | *No info on diagnosis* | *Only liver function reported* |  |
| DeVries, 1998 (liver disease) | Definitely high RoB | Definitely high RoB | Probably high RoB | Definitely low RoB | Probably low RoB | Probably high Rob | Probably high |
| Rationale | *No controls* | *No matched design, nor accounted for influencing factors and adjusted of covariates* | *Not Adequately addressed* | *Biological measurement and appropriate study design* | *Hospital setting and some symptoms before death mentioned* | *Although in hospital, very little info recorded* |  |
| Bhowate, 2021 (mucous fibrosis) | Probably low RoB | Probably high RoB | Probably low RoB | Definitely low RoB | Probably low RoB | Probably low RoB | Probably low |
| Rationale | *Matched for ethnicity, similar inclusion criteria* | *Matched design, not accounted for influencing factors and no covariates adjusted* | *Adequately addressed* | *Biological measurement and appropriate study design* | *Medical centre or hospital and outcome measured via liver biomarkers* | *Medical reports* |  |
| Gong, 2004 (stunting) | Definitely high RoB | Probably low RoB | Probably high RoB | Definitely low RoB | Probably low RoB | Probably high RoB | Probably low |
| Rationale | *no controls* | *No matched controls, influencing factors considered and covariate adjustment* | *Not Adequately addressed* | *Biological measurement and appropriate study design* | *Trained personnel on-site using WHO standards* | *Medical reports but misses info* |  |
| Tan, 1995 (liver disease) | Probably high RoB | Probably high RoB | Probably high RoB | Probably low RoB | Probably low RoB | Probably high RoB | Probably high |
| Rationale | *No info on controls* | *No info on selection, no influencing factors and covariates not considered* | *Not Adequately addressed* | *Biological measurement but no appropriate study design* | *Trained personnel – who criteria* | *Very little info* |  |
| Eom, 2013 (gastric disease) | Probably low RoB | Definitely low RoB | Probably high RoB | Probably low RoB | Probably low RoB | Probably low Rob | Definitely low |
| Rationale | *Matched for age, gender* | *Partially matched, influencing factors accounted for and appropriate covariates included (e.g., calorie intake, alcohol)* | *Not Adequately addressed* | *Via food intake questionnaires but no appropriate study design (CaCo but not nested)* | *Hospital setting but lacks info on diagnosis* | *Hospital records but lacks info* |  |
| Chu, 2018 (liver disease) | Definitely low RoB | Definitely low RoB | Probably low RoB | Defintely ow RoB | Definitely low RoB | Definitely low Rob | Definitely low |
| Rationale | *Matched for age, gender, ethnicity, etc.* | *Matched design, influencing factors considered and adjusted for appropriate covariates (e.g., alcohol)* | *Reason for exclusion mentioned* | *Biological measurement and appropriate study design* | *Hospital setting and diagnosis with liver testing and* abdominal ultrasonography and serological tests. | *Hospital records* |  |
| El-Kafraw, 2005 (liver disease) | Definitely high RoB | Probably high RoB | Definitely low RoB | Probably low RoB | Probably low RoB | Probably low RoB | Probably low |
| Rationale | *No controls* | *No matched design, influencing factors considered but no covariates* | *Info on inclusion and exclusion mentioned well* | *Biological measurement but not appropriate study design* | *Hospital setting but lacks info on diagnosis* | *Hospital records* |  |
| Kirk, 2000 (liver disease) | Defintely low RoB | Definitely low RoB | Definitely low RoB | Probably low RoB | Definitely low RoB | Definitely low Rob | Definitely low |
| Rationale | *Matched for age, gender, recruitment site* | *Matched design, influencing factors accounted for and adjusted for appropriate covariates (e.g., hepatitis)* | *Reasons exclusion adequately addressed* | *Biological measurement but no appropriate study design (CaCo but not nested)* | *Hospital setting and diagnosis mentioned:* HCC had to have compatible clinical and ultrasonographic findings and serum a-fetoprotein levels of 100 ng/ mL or more. | *Hospital records* |  |
| Wu, 2007 (liver disease) | Definitely low Rob | Definitely low RoB | Probably high RoB | Definitely low RoB | Defintely low RoB | Probably low RoB | Definitely low |
| Rationale | *Matched for age, gender, origin* | *Matched design, influencing factors considered, matched for appropriate covariates (e.g., alcohol)* | *Not Adequately addressed* | *Biological measurement and appropriate study design* | *Medical centre and the criteria for HCC diagnosis included: a histopathological examination; a positive lesion detected by at least two different imaging techniques (abdominal ultrasonography, angiogram or computed tomography); or by one imaging technique and a serum AFP level >400 ng/ml.* | *Medical records/questionnaires* |  |
| Magoha, 2014 (wasting & underweight & stunting) | Definitely high RoB | Probably high RoB | Probably low RoB | Probably low RoB | Probably low RoB | Probably low RoB | Probably low |
| Rationale | *No controls* | *No matched controls, some influencing factors considered but no covariates adjusted* | *Adequately addressed* | *Biological measurement and appropriate study design* | *Trained personnel on-site using WHO standards* | *Medical records/questionnaires* |  |
| Hendrickse, 1983 (kwashiorkor) | Probably high RoB | Probably high RoB | Probably high RoB | Probably low RoB | Pobably low Rob | Probably high RoB | Probably high |
| Rationale | *Matched for age* | *Not fully matched, no influencing factors and no covariates adjusted* | *Not Adequately addressed* | *Biological measurement and appropriate study design* | *No info on diagnosis but data on autopsy* | *Little info recorded* |  |
| Oyelami, 1997 (kidney disease & neurologic disease & intestinal disease & kwashiorkor & blood diseases & lung diseases) | Definitely high RoB | Probably high RoB | Definitely low RoB | Probably low RoB | Definitely low RoB | Probably low RoB | Probably low |
| Rationale | *Case series* | *No influencing factors and no covariates considered* | *Case series* | *Biological measurement and appropriate study design* | *Hospital setting with autopsy data* | *Autopsy data* |  |
| Zhou, 1996 (liver disease) | Definitely high RoB | Definitely high RoB | Probably high RoB | Probably low RoB | Probably low RoB | Probably high RoB | Probably high |
| Rationale | *No info* | *No info on diagnosis, no influencing factors considered and no covariates adjusted* | *Lacks info on selection* | *Biological measurement but no appropriate study design* | *Hospital setting but lacks info on diagnosis* | *Although hospital, no info on diagnosis recorded* |  |
| Wang, 2020 (oesophageal disease) | Probably low RoB | Definitely low RoB | Probably low RoB | Definitely low RoB | Probably low RoB | Probably low RoB | Definitely low |
| Rationale | *Matched for age, gender* | *Matched design, influencing factors considered and adjusted for appropriate covariates (e.g., smoking, alcohol)* | *Reason for exclusion mentioned* | *Biological measurement and appropriate study design* | *Trained personnel on-site and* routine endoscopy examination | *Medical records/questionnaires* |  |
| Rogan, 1985 (neurologic disease) | Probably high RoB | Probably high RoB | Probably high RoB | Probably low RoB | Definitely low RoB | Definitely low Rob | Probably low |
| Rationale | *No info* | *No matching, no influencing factors nor covariates considered* | *Not Adequately addressed* | *Biological measurement but no appropriate study design* | *Hospital setting* | *Hospital records* |  |
| Voth-Gaeddert, 2018 (stunting) | Definitely high RoB | Definitely high RoB | Probably high RoB | Probably low RoB | Probably low RoB | Probably high RoB | Probably high |
| Rationale | *No controls* | *No matched controls, no influencing factors and no covariates considered* | *Not Adequately addressed* | *Food analysis and appropriate study design* | *Medical centre using WHO standards* | *Very little info* |  |
| Ming, 2002 (liver disease) | Definitely high RoB | Probably high RoB | Probably high RoB | Probably low RoB | Probably low RoB | Probably low RoB | Probably low |
| Rationale | *No controls* | *No matched controls, influencing factors considered but no covariates adjusted* | *Not Adequately addressed* | *Biological measurement but no appropriate study design* | *Hospital setting but lacks info on the diagnosis* | *Hospital records* |  |
| Gong, 2012 (liver disease) | Definitely high RoB | Probably high RoB | Probably high RoB | Probably low RoB | Definitely low RoB | Definitely low RoB | Probably low |
| Rationale | *No controls* | *No matched controls, accounting for influencing factors, only village was considered as covariate* | *Excluded when > 17 year, no other info* | *Biological measurement but no appropriate study design (Cs)* | *Hospital setting, enlarged liver and spleen* | *Hospital records* |  |
| Wafa, 1998 (kidney disease) | Definitely high RoB | Definitely high RoB | Probably high RoB | Probably low RoB | Definitely low RoB | Definitely low Rob | Probably high |
| Rationale | *No matching* | *No matching, no influencing factors, no covariates considered* | *No info on follow-up* | *Biological measurement but no appropriate study design* | *Hospital setting with laboratory, radiological and histopathology evaluation of kidney status* | *Hospital records* |  |
| Gichohi-Wainaina, 2023 (underweight & stunting) | Definitely high RoB | Probably high RoB | Probably high RoB | Probably low RoB | Probably low RoB | Probably high RoB | Probably high |
| Rationale | *No controls* | *No matching, some influencing factors considered but no adjustment of covariates* | *Not Adequately addressed* | *Food analysis and ideal study design* | *Trained personnel on-site using WHO standards* | *Medical records/questionnaires* |  |
| Afum, 2016 (liver disease) | Definitely high RoB | Probably high RoB | Probably low RoB | Probably low RoB | Probably low RoB | Probably low RoB | Probably low |
| Rationale | *No matching* | *No matching, influencing factors considered but covariates not adjusted* | *Reason for exclusion mentioned* | *Biological measurement but no appropriate study design* | *Hospital setting but lacks info on diagnosis* | *Hospital records but lacks info* |  |
| Ning, 2023 (musculoskeletal disease) | Probably low RoB | Probably low RoB | Definitely low RoB | Definitely low RoB | Probably low RoB | Probably high RoB | Probably low |
| Rationale | *Matched for age and gender* | *Matched, influencing factors accounted but no covariates adjusted* | *Adequately addressed inclusion and exclusion* | *Biological measurement (golden standard) and appropriate design* | *Governmental research on-site – trained personnel* | *Little information recorded* |  |
| Jolly, 2007 (acute food intoxication) | Definitely high RoB | Probably high RoB | Probably high RoB | Probably low RoB | Probably low RoB | Probably high RoB | Probably high |
| Rationale | *No controls* | *No matched design, influencing factors considered but no adjustment of covariates* | *Not Adequately addressed* | *Biological measurement but no appropriate study design (Cs)* | *Trained personnel on-site with laboratory testing for diagnosis* | *Medical records/questionnaires* |  |
| Jiang, 2005 (immunomodulation) | Definitely high RoB | Definitely high RoB | Probably high RoB | Probably low RoB | Definitely high Rob | Probably low RoB | Probably high |
| Rationale | *No controls* | *No matched controls, no influencing factors and covariates included* | *Not Adequately addressed* | *Biological measurement and no appropriate study design* | *No info on diagnosis* | *Many parameters recorded* |  |
| Massart, 2008 (premature puberty) | Probably low RoB | Probably high RoB | Probably high RoB | Probably low RoB | Definitely low RoB | Definitely low RoB | Probably low |
| Rationale | *Matched for age and gender* | *Not fully matched, some influencing factors considered but no adjustment of covariates* | *Not Adequately addressed* | *Biological measurement but no ideal study design (CaCo but not nested)* | *Hospital setting and diagnostic parameters well documented* | *Hospital records* |  |
| Kimanya, 2010 (stunting) | Definitely high RoB | Probably high RoB | Probably high RoB | Probably low RoB | Probably high RoB | Probably high RoB | Probably high |
| Rationale | *No controls* | *No influencing factors* | *Not Adequately addressed* | *Food intake questionnaires and ideal study design (cohort)* | *Trained personnel on-site using questionnaires* | *Medical records with questionnaires* |  |
| Hoffmann, 2018 (stunting) | Definitely high RoB | Probably high RoB | Probably high RoB | Probably low RoB | Probably low RoB | Probably low RoB | Probably low |
| Rationale | *Not matched* | *Not matched, accounted for influencing factors but no adjustment of covariates* | *Indirect evidence that not all was included* | *Biological measurement but no ideal study design (Cs)* | *Trained personnel on-site using WHO standards & guidelines* | *WHO validated reports* |  |
| Ngindu, 1982 (liver disease) | Definitely high RoB | Probably high RoB | Definitely low RoB | Definitely low RoB | Definitely low RoB | Definitely low RoB | Probably low |
| Rationale | *Case series* | *No matched controls, influencing factors considered but no covariates adjusted* | *Adequately addressed* | *Biological and food measurement and appropriate study design* | *Hospital setting, clinical features, lab analyses of liver disease and pathophysiological examination* | *Hospital records* |  |
| deVries, 1990 (kwashiorkor) | Definitely high RoB | Probably high RoB | Probably high RoB | Probably low RoB | Definitely low RoB | Definitely low RoB | Probably low |
| Rationale | *No controls* | *No matched controls, no influencing factors nor covariates considered* | *Not Adequately addressed* | *Biological measurement but no appropriate design for kwashiorkor and stunting* | *Hospital setting using WHO standards* | *Hospital records* |  |
| Anitha, 2014 (liver disease) | Probably high RoB | Probably high RoB | Probably high RoB | Defintely low RoB | Definitely low RoB | Definitely low RoB | Probably low |
| Rationale | *No matching of age, gender but living in same household* | *No matching, some influencing factors considered, no adjustment of covariates* | *Not Adequately addressed* | *Biological measurement and appropriate study design* | *Hospital setting and clinical profiling of liver disease mentioned* | *Hospital records* |  |
| Yu, 1997 (liver disease) | Probably low RoB | Definitely low RoB | Probably low RoB | Defintely low RoB | Definitely low RoB | Definitely low RoB | Definitely low |
| Rationale | *Matched for age and gender* | *Matched, influence factors considered, adjusted for appropriate covariates (e.g., alcohol consumption)* | *Reason for exclusion mentioned* | *Biological measurement and appropriate study design (NCC)* | *Hospital setting and diagnosis via fetoprotein measurement, ultrasonographic images* | *Hospital records* |  |
| Buetow, 1992 (liver disease) | Definitely high RoB | Probably high RoB | Definitely low RoB | Probably low RoB | Probably low RoB | Probably low RoB | Probably low |
| Rationale | *Case report* | *No influencing factors accounted for, no covariates* | *Case report* | *Biological measurement* | *Hospital setting but no further info* | *Although hospital setting, only focused on mutations* |  |
| Zeng, 2017 (liver disease & metabolic disease) | Probably high RoB | Probably low RoB | Probably high RoB | Probably low RoB | Probably high RoB | Probably low RoB | Probably low |
| Rationale | *No info on matching of controls* | *No info matching, accounted for influencing factors and appropriative covariates (e.g., alcohol consumption)* | *Not Adequately addressed* | *Food analysis but no appropriate design (Cs)* | *Trained personnel on-site with questionnaire* | *Medical records* |  |
| Nasir, 2021 (liver disease & wasting & underweight & stunting) | Definitely high RoB | Probably high RoB | Probably high RoB | Definitely low RoB | Probably low RoB | Probably low RoB | Probably low |
| Rationale | *No controls* | *No matched controls, adjusted for influencing factors but lacks info on covariates* | *Not Adequately addressed* | *Biological measurement and appropriate study design (Cs)* | *Trained personnel using WHO standards* | *Medical records* |  |
| Shuaib, 2010 (adverse birth outcomes & blood diseases) | Definitely high RoB | Probably low RoB | Probably low RoB | Definitely low RoB | Probably low RoB | Probably low RoB | Definitely low |
| Rationale | *No controls* | *No matched controls, adjusted for influencing factors and covariates* | *Reasons mentioned* | *Biological measurement and appropriate study design* | *Trained personnel* | *Medical records* |  |
| Tessema, 2021 (stunting) | Probably high RoB | Probably low RoB | Probably high RoB | Definitely low RoB | Probably low RoB | Probably low RoB | Probably low |
| Rationale | *No info* | *No info on matching, influencing factors and covariates were considered (e.g., harvest period)* | *Not Adequately addressed* | *Biological measurement (golden standard) and appropriate design (longitudinal)* | *Trained personnel on-site with questionnaire using WHO standards* | *Medical records* |  |
| Brera, 2013 (intestinal disease) | Probably high RoB | Definitely high RoB | Probably high RoB | Probably low RoB | Definitely high Rob | Probably high RoB | Probably high |
| Rationale | *No info on matched controls* | *No info on matching, no influencing factors, no info on covariates* | *Not Adequately addressed* | *Biological measurement but no appropriate study design (CaCo but not nested)* | *No info on diagnosis* | *Outcomes not mentioned for all mycotoxin exposure* |  |
| Peers, 1987 (liver disease) | Definitely high RoB | Probably high RoB | Probably high RoB | Probably low RoB | Definitely high Rob | Probably high RoB | Definitely high |
| Rationale | *No controls* | *No matched controls, influencing factors considered but no covariates considered* | *Not Adequately addressed* | *Food analysis and no appropriate design for cancer* | *No info on diagnosis* | *Outcomes not mentioned for all mycotoxin exposure* |  |
| Tesfamariam, 2022 (adverse birth outcomes) | Definitely high RoB | Probably high RoB | Definitely low RoB | Definitely low RoB | Probably high RoB | Probably low RoB | Probably high |
| Rationale | *No controls* | *No controls, adjusted for covariates and some influencing factors* | *Reasons exclusion adequately addressed* | *Biological measurement with golden standard and appropriate study design (Cohort)* | *Trained personnel on-site with questionnaire* | *Medical records* |  |
| Maaroufi, 1995 (kidney disease) | Definitely high RoB | Definitely high RoB | Probably high RoB | Probably low RoB | Probably low RoB | Probably high RoB | Probably high |
| Rationale | *No matching* | *No matching, not accounted for covariates or influencing factors* | *Not Adequately addressed* | *Biological measurement but no appropriate study design (CaCo but not nested)* | *Hospital setting but no info on diagnosis* | *Only outcomes for positive/high mycotoxin detected* |  |
| Campbell, 1990 (liver disease) | Definitely high RoB | Definitely high RoB | Probably high RoB | Probably low RoB | Definitely high Rob | Probably low RoB | Definitely high |
| Rationale | *No controls* | *No matched design, not accounted for covariates or influencing factors* | *Not Adequately addressed* | *Biological measurement but no appropriate study design (Cs)* | *No info on diagnosis* | *Medical records* |  |
| Nogueira, 2009 (liver disease) | Definitely high RoB | Probably high RoB | Definitely low RoB | Probably low RoB | Probably low RoB | Definitely low RoB | Probably high |
| Rationale | *No matching* | *No matching, not accounted for covariates but taken into account influencing factors* | *Exclusion adequately addressed* | *Biological measurement but no appropriate study design (CaCo but not nested)* | *Hospital setting but diagnosis not specified* | *Hospital records* |  |
| Kamala, 2018 (acute food intoxication & underweight) | Definitely high RoB | Definitely high RoB | Probably high RoB | Probably low RoB | Probably low RoB | Probably high RoB | Probably high |
| Rationale | *No controls* | *No matched design, not accounted for covariates nor influencing factors* | *Not Adequately addressed* | *Food analysis* | *Hospital setting but no further info* | *Although in hospital, little recorded for outcomes* |  |
| Aslam, 2012 (bladder cancer) | Probably low RoB | Probably high RoB | Probably high RoB | Probably low RoB | Probably low RoB | Probably high RoB | Probaby high |
| Rationale | *Not matched for gender, but for age & origin* | *Not fully matched, no influencing factors considered and lacks info on covariates* | *Not Adequately addressed* | *Biological measurement but no appropriate study design (CaCo but not nested)* | *Probably hospital setting but further info lacks* | *Little info recorded* |  |
| Lino-silva, 2022 (liver disease) | Definitely high RoB | Probably low RoB | Probably high RoB | Definitely low RoB | Probably low RoB | Probably low RoB | Probably low |
| Rationale | *No controls* | *No matched controls, influence factors were considered but covariates were not considered* | *Not Adequately addressed* | *Biological measurement (golden standard) and appropriate study design (cohort)* | *Medical centre and HCC diagnosis was confirmed by central review* | *Medical reports* |  |
| Yeh, 2010 (liver disease) | Definitely high RoB | Definitely high RoB | Definitely low RoB | Definitely low RoB | Definitely low RoB | Definitely low RoB | Probably low |
| Rationale | *Case series– no controls* | *No matched controls, no influence factors considered and not adjusted for covariates* | *Case series* | *Biological measurement and appropriate study design* | *Hospital setting with pathological features evaluated by two pathologists* | *Hospital records* |  |
| Jolly, 2011 (immunomodulation & liver disease) | Definitely high RoB | Probably low RoB | Probably high RoB | Probably low RoB | Probably low RoB | Probably low RoB | Probably low |
| Rationale | *No matching* | *No matching, accounted for some influencing factors and adjusted for appropriate covariates (e.g., education)* | *Not Adequately addressed* | *Biological measurement but no appropriate study design (Cs)* | *Hospital setting but lacks specific on the outcome assessment* | *Hospital records* |  |
| Shimizu, 1999 (liver disease) | Definitely high RoB | Probably high RoB | Probably high oB | Probably low RoB | Probably low RoB | Probably high RoB | Probably high |
| Rationale | *No controls* | *No matched controls, influencing factors considered but no covariates mentioned* | *Not Adequately addressed* | *Biological measurement but no appropriate study design* | *Hospital setting but lacks info on the diagnosis* | *Although in hospital, very little info on outcome reported* |  |
| Smpokou, 2019 (kidney disease) | Definitely low RoB | Probably low RoB | Definitely high RoB | Definitely low RoB | Probably low Rob | Probably low RoB | Probably low |
| Rationale | *Age, gender & community matched* | *Matched, influencing factors considered but no info in adjustment* | *Not Adequately addressed* | *Biological measurement (golden standard) and appropriate study design (NCC)* | *Probably hospital setting and outcome measures were mentioned* | *Medical records* |  |
| Tchana, 2010 (liver disease & kwashiorkor) | Definitely low RoB | Probably low RoB | Probably high RoB | Definitely low RoB | Probably low RoB | Probably low RoB | Probably low |
| Rationale | *Age, gender & residence matched* | *Matched, influencing factors considered, no info on covariates* | *Not Adequately addressed* | *Biological measurement and appropriate study design (CaCo)* | *Hospital setting but lacks info on the outcome* | *Hospital records but lacks details in the article* |  |
| Tomerak, 2011 (liver disease) | Definitely high RoB | Probably high RoB | Probably high RoB | Probably low RoB | Probably low RoB | Probably high RoB | Probably high |
| Rationale | *No controls* | *No controls, no influencing factors and little info on covariates* | *Not Adequately addressed* | *Biological measurement but no appropriate study design (Cs)* | *Hospital setting but lacks outcome info* | *Although in hospital, very little info recorded* |  |
| Okoth, 2004 (kwashiorkor) | Definitely high RoB | Definitely high RoB | Probably high RoB | Probably low RoB | Probably high RoB | Definitely high Rob | Definitely high |
| Rationale | *No controls* | *No controls, no influencing factors and no covariates considered* | *Not Adequately addressed* | *Food analysis and no appropriate study design (Cs)* | *Trained personnel on-site with questionnaire using WHO standards* | *No info recorded on outcome* |  |
| deCarvalho, 2013 (liver disease) | Definitely high RoB | Probably high RoB | Definitely low RoB | Probably low RoB | Probably low RoB | Probably high RoB | Probably low |
| Rationale | *No controls* | *No controls, influencing factors were accounted but covariates not considered* | *Exclusion adequately addressed* | *Biological measurement but the study design was not appropriate (Cs)* | *Hospital setting but lacks info on the diagnosis* | *Although in hospital very little info recorded* |  |
| Díaz deLeón-Martínez, 2019 (kidney disease) | Definitely high RoB | Probably high RoB | Probably high RoB | Probably low RoB | Probably high RoB | Probably high RoB | Definitely high |
| Rationale | *No controls* | *No matched controls, no influencing factors and adjusted covariates* | *Not Adequately addressed* | *Biological measurement but small pilot study* | *Trained personnel on-site with questionnaire* | *Little info recorded* |  |
| Karamkhani, 2020 (kidney disease & liver disease) | Probably high RoB | Definitely high RoB | Probably high RoB | Definitely low RoB | Definitely high Rob | Probably high RoB | Probably high |
| Rationale | *No info* | *No info, no influencing factors, lacks covariate inclusion* | *Not Adequately addressed* | *Biological measurement and appropriate study design* | *No info on diagnosis* | *Little info recorded* |  |
| Fry, 2022 (male reproductive disease) | Definitely high RoB | Definitely high RoB | Probably high RoB | Probably low RoB | Probably low RoB | Probably low RoB | Probably low |
| Rationale | *No controls* | *No matching, no influencing factors, lacks info on covariates* | *Not Adequately addressed* | *Biological measurement but no appropriate study design (post-diagnosis)* | *Medical procedure – probably in hospital using prostatectomy* | *Medical records* |  |
| Shank, 1971 (acute food intoxication) | Probably high RoB | Definitely high RoB | Probably high RoB | Definitely low RoB | Probably low RoB | Probably high RoB | Probably high |
| Rationale | *Matched for ethnicity/residence* | *Not fully matched, no influencing factors and no covariate adjustment* | *Not Adequately addressed* | *Biological measurement and appropriate study design* | *Hospital setting and autopsy samples, but no clinical parameters reported* | *Although in hospital, limited clinical outcomes reported* |  |
| Abedi-Ardekani, 2011 (liver disease) | Definitely high RoB | Probably high RoB | Probably high RoB | Probably low RoB | Definitely low RoB | Definitely low RoB | Probably low |
| Rationale | *No controls* | *No matching design, influencing factors accounted for but no covariates adjustment* | *Not Adequately addressed* | *Biological measurement but no appropriate study design (Cs)* | *Hospital setting and histological and immunohistochemical confirmed diagnosis* | *Hospital records* |  |
| Andrews-Trevino, 2021 (stunting) | Definitely high RoB | Probably low RoB | Probably high RoB | Definitely low RoB | Probably high RoB | Probably low RoB | Probably low |
| Rationale | *no controls* | *No matching, accounted for influencing factors and covariates included* | *Not Adequately addressed* | *Biological measurement and appropriate study design (longitudinal cohort)* | *Trained personnel on-site with questionnaire* | *Relevant outcomes reported by trained personnel* |  |
| Mcmillan, 2018 (kwashiorkor) | Probably high RoB | Probably low RoB | Probably high RoB | Definitely low RoB | Probably low RoB | Definitely low RoB | Probably low |
| Rationale | *No info on controls* | *No info on matching, accounted for influencing factors and only adjusted for age* | *Not Adequately addressed* | *Biological measurement (golden standard) and appropriate study design (cohort)* | *Hospital setting and using WHO standards* | *Hospital records* |  |
| Watson, 2018 (underweight & stunting) | Definitely high RoB | Probably low RoB | Definitely low RoB | Probably low RoB | Probably high RoB | Probably low RoB | Probably low |
| Rationale | *no controls* | *No matched design, accounted for influencing factors and appropriate covariates (e.g., season of sampling, mother’s household quality)* | *Adequately addressed* | *Biological measurement (no golden standard) and appropriate study design (cohort)* | *Questionnaires* | *Relevant outcomes reported by trained personnel* |  |
| Tesfamariam, 2022 (adverse birth outcomes) | Definitely high RoB | Probably low RoB | Definitely low RoB | Definitely low RoB | Probably high RoB | Probably low RoB | Probably low |
| Rationale | *No controls* | *No matching, accounted for influencing factors and covariates* | *Adequately addressed* | *Biological measurement (golden standard) and appropriate study design (cohort)* | *Questionnaires* | *Relevant outcomes reported* |  |
| Liu, 2008 (liver disease) | Definitely low RoB | Definitely low RoB | Definitely low RoB | Definitely low RoB | Definitely low RoB | Probably low RoB | Definitely low |
| Rationale | *Age, gender & serology-status matched* | *Matched design, accounted for influencing factors and adjusted for covariates* | *Adequately addressed* | *Biological measurement (no golden standard) and ideal study design* | *Hospital setting and reference to cancer diagnosis (national diagnostic criteria)* | *Relevant outcomes reported* |  |
| Wild, 1993 (liver disease) | Definitely high RoB | Probably high RoB | Definitely low RoB | Probably low RoB | Definitely high Rob | Probably low RoB | Probably high |
| Rationale | *cohort* | *No matching, accounted for influencing factors but no covariates included* | *Adequately addressed* | *Biological measurement (no golden standard) and appropriate study design* | *No info on diagnosis* | *Relevant outcomes reported* |  |
| El-Shanawani, 2006 (liver disease) | Probably low RoB | Probably low RoB | Probably high RoB | Probably low RoB | Probably low RoB | Probably high RoB | Probably low |
| Rationale | *Described that there was matching but not specified how* | *Matched, influencing factors accounted for but no adjustment* | *Exclusion criteria not mentioned* | *Biological measurement (no golden standard) but no appropriate study design* | *Examination mentioned* | *Little info recorded* |  |
| Brewer, 2013 (neurologic disease) | Probably high RoB | Definitely high RoB | Probably high RoB | Probably low RoB | Probably low RoB | Probably low RoB | Probably low |
| Rationale | *Matching not described* | *No matching, not all influencing factors accounted for nor covariates* | *Exclusion criteria not mentioned* | *Biological measurement (no golden standard) but no appropriate study design* | *In the clinic and diagnosis mentioned* | *In the clinic with medical reports* |  |
| Mokhles, 2007 (liver disease) | Probably low RoB | Probably high RoB | Probably low RoB | Probably low RoB | Definitely low RoB | Definitely low RoB | Probably low |
| Rationale | *Age & gender matched* | *Matched design, not all influencing factors accounted for and no adjustment* | *Exclusion criteria well mentioned* | *Biological measurement (no golden standard) but no appropriate study design (CaCo but not nested)* | *Hospital setting and all factors of diagnosis mentioned (full history of clinical examination)* | *Relevant outcomes reported with medical report* |  |
| Yu, 1992 (liver disease) | Definitely high RoB | Probably high RoB | Probably high RoB | Probably low RoB | Probably low RoB | Probably low RoB | Probably low |
| Rationale | *No controls* | *No matched design, influencing factors accounted for but no adjustment of covariates* | *Exclusion criteria not mentioned* | *Biological measurement (no golden standard) but no appropriate design* | *Hospital setting but diagnosis not specified* | *Relevant outcomes reported* |  |
| Manda, 2018 (liver disease) | Definitely low RoB | Probably low RoB | Definitely low RoB | Probably low RoB | Definitely low RoB | Definitely low RoB | Definitely low |
| Rationale | *Age, gender & residence matched* | *Matched design, influencing factors accounted for but no appropriate covariates* | *Adequately addressed* | *Biological measurement (no golden standard) but no appropriate study design (CaCo but not nested)* | *Hospital setting with medical dossier containing the clinical diagnosis via biomarker analyses (e.g., fetoprotein)* | *Hospital records* |  |
| Huţanaşu, 2011 (liver disease) | Definitely low RoB | Probably low RoB | Probably low RoB | Probably low RoB | Definitely high RoB | Probably low RoB | Probably low |
| Rationale | *Age, gender & residence matched* | *Matched design, influencing factors accounted for* | *Adequately addressed* | *Biological measurement (no golden standard) but no appropriate study design (CaCo but not nested)* | *Patients but not clear from hospital and no info on diagnosis* | *Patients but not clear from hospital* |  |
| Krishnamachari, 1975 (acute food intoxication) | Definitely high RoB | Probably high RoB | Definitely high RoB | Probably low RoB | Probably high RoB | Probably high RoB | Probably high |
| Rationale | *Outbreak report* | *No matching design, influencing factors accounted for but no covariates adjusted* | *Not all cases measured* | *Biological measurement (no golden standard) and appropriate design* | *Little info recorded* | *Little info recorded* |  |
| Lauer, 2019 (underweight & stunting & lung diseases) | Definitely high RoB | Definitely low RoB | Definitely low RoB | Definitely low RoB | Probably low RoB | Probably low RoB | Definitely low |
| Rationale | *no controls* | *No matched design, covariates considered (e.g., weight mother, education), and influencing factors accounted for* | *Adequately addressed* | *Biological measurement (no golden standard) and appropriate design* | *District heath centre* | *Relevant outcomes reported* |  |
| Leon, 1998 (neurologic disease) | Definitely high RoB | Definitely high RoB | Probably high RoB | Definitely low RoB | Probably low RoB | Probably low RoB | Probably low |
| Rationale | *No controls* | *No matching design, no covariates considered, not all influencing factors accounted for* | Little info recorded | *Biological measurement (no golden standard) and appropriate study design* | *Info on diagnosis but no hospital setting* | *Info on diagnosis but no hospital setting* |  |
| Szymańska, 2009 (liver disease) | Probably low RoB | Probably low RoB | Definitely high RoB | Probably low RoB | Definitely low RoB | Probably low RoB | Definitely low |
| Rationale | *Age & gender matched* | *Matched design, no covariates adjusted all influencing factors accounted for* | *No info recorded* | *Biological measurement (no golden standard) and appropriate study design (cohort)* | *Hospital setting and diagnosis via alfa-protein, alanine aminotransferase and ultrasound* | *Relevant outcomes reported* |  |
| Lamplugh, 1982 (kwashiorkor) | Definitely high RoB | Probably high RoB | Probably high RoB | Probably low RoB | Definitely high RoB | Definitely high RoB | Definitely high |
| Rationale | *No controls* | *Little info recorded* | Little info recorded | *Biological measurement (no golden standard) and no appropriate study design (Cs)* | *No info on diagnosis* | *Little info recorded* |  |
| Andrews-Trevino, 2019 (adverse birth outcomes) | Definitely high RoB | Probably low RoB | Definitely high RoB | Probably high RoB | Definitely high RoB | Definitely high RoB | Probably high |
| Rationale | *No controls* | *No matching, adjusted for appropriate covariates (e.g., maternal education, wealth index) all influencing factors accounted for* | *No information on missing data* | *Little info on analysis but appropriate study design (cohort)* | *Little info on diagnosis* | *Little info on diagnosis* |  |
| Jiang, 1998 (liver disease) | Definitely high RoB | Definitely high RoB | Definitely low RoB | Probably low RoB | Probably low RoB | Probably high RoB | Probably high |
| Rationale | *No controls* | *No matching, no covariates included, no influencing factors accounted for* | *Case report* | *Biological measurement (no golden standard) but case report* | *Most probably in hospital setting* | *Little info on diagnosis* |  |
| Sayed, 2005 (liver disease) | Definitely high RoB | Probably high RoB | Definitely high RoB | Probably high RoB | Probably high RoB | Probably high RoB | Definitely high |
| Rationale | *No controls* | *No mathcing design, appropriate covariates not adjusted but influencing factors accounted for* | *Missing data* | *Biological measurement (no golden standard) but no appropriate study design (Cs)* | *Questionnaire* | *Little info recorded* |  |
| Memiş, 2020 (stunting) | Definitely high RoB | Probably low RoB | Probably low RoB | Probably low RoB | Definitely low RoB | Definitely low RoB | Definitely low |
| Rationale | *No controls* | *No matching design, appropriate covariates not adjusted, but influencing factors accounted for* | *Exclusion mentioned* | *Biological measurement (no golden standard) and appropriate study design* | *Hospital setting and WHO standards* | *Hospital records* |  |
| Simon, 2003 (neurologic disease) | Definitley high RoB | Definitely high RoB | Probably low RoB | Definitely low RoB | Probably low RoB | Probably low RoB | Probably low |
| Rationale | *No matching of control* | *No matching design, influencing factors accounted for* | *Exclusion mentioned* | *Biological measurement (no golden standard) and appropriate study design (CaCo)* | Specialized health centre and outcome assessment mentioned | Trained personnel and outcomes reported |  |
| Huybrechts, 2024 (liver disease) | Definitely low RoB | Probably low RoB | Probably low RoB | Probably low RoB | Probably low RoB | Probably low RoB | Definitely low |
| Rationale | *Age, gender & residence matched* | *Matched design, adjusted for appropriate covariates (e.g., processed meat) not all influencing factors accounted for* | *Exclusion mentioned* | *Dietary food questionnaire but appropriate study design (NCC)* | *Multi-centre with international diagnostic criteria* | *Relevant outcomes reported* |  |
| Koshiol, 2024 (gallbladder cancer) | Definitely low RoB | Definitely low RoB | Probably low RoB | Definitely low RoB | Definitely low RoB | Definitely low RoB | Definitely low |
| Rationale | *Age, gender & residence matched* | *Matched design, adjustment for appropriate covariates (e.g., education, smoking) and influencing factors accounted for* | *Exclusion mentioned* | *Biological measurement with golden standard and appropriate study design (NCC)* | Shanghai Cancer registry and routing follow up for diagnosis of gallbladder cancer | Medical chart review, Shanghai Cancer registry |  |
| Yadav, 2025 (gallbladder cancer) | Definitely low RoB | Probably low RoB | Probably low RoB | Probably low RoB | Definitely low RoB | Definitely low RoB | Definitely low |
| Rationale | *Age, gender & residence matched* | *Matched design, adjustment was performed but not for appropriate covariates influencing factors accounted for* | *Exclusion mentioned* | *Biological measurement (no golden standard) but no appropriate design (CaCo but not nested)* | *Hospital setting and diagnosis mentioned (radiological evidence of gallbladder mass or mural thickening)* | *Hospital records* |  |
| Rodriguez, 2025 (pancreatic cancer) | Probably high RoB | Probably high RoB | Probably low RoB | Probably low RoB | Probably low RoB | Probably low RoB | Probably low |
| Rationale | *Only age matched* | *No covariates adjusted but influencing factors accounted for* | *Exclusion mentioned* | *Biological measurement with golden standard but retrospective design* | *Cancer centrum and medical records* | *Relevant outcomes reported* |  |
| Zhong, 2024 (adverse birth outcomes) | Definitely high RoB | Probablyl low RoB | Probably high RoB | Definitly low RoB | Probably low RoB | Probably low RoB | Definitely low |
| Rationale | *no controls* | *Not all covariates included but influencing factors accounted for* | *Little info recorded* | *Biological measurement (no golden standard) and appropriate study design (cohort)* | *Probably hospital setting and outcome assessment mentioned* | *Hospital records* |  |
| Tan, 2023 (adverse birth outcomes) | Definitely high RoB | Probably low RoB | Probably high RoB | Definitely low RoB | Probably low RoB | Probably low RoB | Definitely low |
| Rationale | *no controls* | *Adjusted for appropriate covariates (e.g., maternal age, BMI) and influencing factors accounted for* | Little info recorded | *Biological measurement with golden standard and appropriate study design (Cohort)* | *Probably hospital setting and criteria for outcome assessment described* | *Hospital records* |  |
| Lefèvre-Arbogast, 2024 (neurologic disease) | Definitely high RoB | Probably low RoB | Probably low RoB | Probably low RoB | Probably low RoB | Probably low RoB | Definitely low |
| Rationale | *no controls* | *Adjusted for appropriate covariates (e.g., lipid metabolism), influencing factors accounted for* | *Exclusion mentioned* | *Dietary food questionnaires and reliable study design (cohort)* | *Trained personnel and diagnostic criteria are well documented* | *Relevant outcomes reported* |  |
| Murashiki, 2025 (blood diseases) | Definitely high RoB | Probably low RoB | Probably low RoB | Definetly low RoB | Probably low RoB | Definity high RoB | Probably low |
| Rationale | *no controls* | *Adjusted for appropriate covariates (e.g., hepatitis status, season) influencing factors accounted for* | Exclusion mentioned | *Biological measurement (no golden standard) and appropriate study design (cohort)* | *Probably hospital setting but lacks info* | No info recorded |  |
| Pratiwi, 2024 (blood diseases) | Probably high RoB | Probably high RoB | Probably high RoB | Probably low RoB | Probably high RoB | Probably low RoB | Probably high |
| Rationale | *No info recorded* | *No covariates included, and not all influencing factors accounted for* | *Little info recorded* | *Biological measurement (no golden standard) but not appropriate study design (Cs)* | *Probably hospital setting but lacks info* | *Hospital records* |  |
| Osoro, 2024 (adverse birth outcomes) | Definitely low RoB | Definitely low RoB | Probably high RoB | Definitely low RoB | Probably low RoB | Probably low RoB | Definitely low |
| Rationale | *Age, gender, gestational weight & residence matched* | *Matched design, appropriate covariates were included, influencing factors accounted for* | *Little info recorded* | *Biological measurement with golden standard but appropriate study design (NCC)* | *Trained personnel* | *Relevant outcomes reported* |  |
| Ning, 2024 (male reproductive disease) | Probably high RoB | Probably low RoB | Probably low RoB | Probably low RoB | Probably low RoB | Probably high RoB | Probably low |
| Rationale | *Little info recorded* | *Adjusted for reliable covariates (e.g., partner ever pregnant) and influencing factors considered* | *Exclusion mentioned* | *Biological measurement with golden standard but no appropriate study design (Cs)* | *Probably hospital setting and infertility based on semen quality* | *Little info record* |  |
| Yang, 2024 (male reproductive disease) | Probably high RoB | Probably low RoB | Probably low RoB | Probably low RoB | Probably low RoB | Probably low RoB | Probably low |
| Rationale | *Little info recorded* | *Adjusted for reliable covariates (e.g., partner ever pregnant) and influencing factors considered* | *Exclusion mentioned* | *Biological measurement with golden standard but no appropriate study design (Cs)* | *Probably hospital setting and infertility based on semen quality* | *Relevant outcomes reported* |  |
| Okonofua, 2024 (male reproductive disease) | Probably high RoB | Definitely high RoB | Probably low RoB | Probably low RoB | Probably low RoB | Probably low RoB | Probably low |
| Rationale | *Little info recorded* | *No matching design, lacks info on confounders, not all influencing factors accounted for* | *Exclusion mentioned* | *Biological measurement with golden standard but no appropriate study design (CaCo without nested design)* | *Probably hospital setting* | *Relevant outcomes reported* |  |
| Ayeni, 2024 (intestinal disease) | Definitely high RoB | Definitely high RoB | Probably low RoB | Definitely low RoB | Probably high RoB | Probably high RoB | Probably high |
| Rationale | *no controls* | *No matching design, lacks info on confounders, not all influencing factors accounted for* | *Exclusion mentioned* | *Biological measurement with golden standard and appropriate study design (Cohort)* | *Probably hospital setting but lacks info on clinical diagnosis* | Lacks info |  |
| Lisieska-Żołnierczyk, 2024 (intestinal disease) | Definitely low RoB | Probably high RoB | Definitely low RoB | Probably low RoB | Definitely low RoB | Probably low RoB | Probably low |
| Rationale | *Age, gender & residence matched* | *Matched design, lacks info on confounders, not all influencing factors accounted for* | *Exclusion mentioned, no loss follow-up* | *Biological measurement (no golden standard) but less appropriate study design (Cs)* | *Hospital setting and histologically confirmed diagnosis* | *Hospital records* |  |
| Oesterle, 2024 (intestinal disease) | Definitely high RoB | Definitely high RoB | Probably low RoB | Definitely low RoB | Probably low RoB | Definitely low RoB | Probably low |
| Rationale | *no controls* | *No matching design, and no influencing factors accounted for* | *Exclusion mentioned* | *Biological measurement with golden standard and appropriate study design (Cohort)* | *Probably hospital setting* | Hospital records |  |
| Mulisa, 2025 (oesophageal disease) | Definitely low RoB | Definitely low RoB | Probably low Rob | Probably low RoB | Definitely high RoB | Probably low RoB | Probably low |
| Rationale | *Age, gender & residence matched* | *Matched design, adjusted for reliable confounders (e.g., smoking, hot drinks), most influencing factors accounted for* | *Exclusion mentioned* | *Biological measurement with golden standard but no appropriate study design (CaCo without nested design)* | *Lacks info on the diagnosis* | Hospital records |  |
| Ashraf, 2022 (wasting & underweight & stunting) | Definitely high RoB | Probably high RoB | Probably low Rob | Definitely low RoB | Probably low RoB | Probably high RoB | Probably low |
| Rationale | *on controls* | *No matched design, lacks info on reliable confounding, not all influencing factors accounted for* | *Exclusion mentioned* | *Biological measurement (no golden standard) and appropriate study design (Cohort)* | *Probably hospital setting and WHO Child Growth Standard used* | *Little info record* |  |
| Unicsovics, 2024 (female reproductive disease) | Definitely high RoB | Probably high RoB | Probably low Rob | Definitely low RoB | Probably low RoB | Probably high RoB | Probably low |
| Rationale | *no controls* | *Matched design, lacks info on reliable confounding, not all influencing factors accounted for* | *Exclusion mentioned* | *Biological measurement (no golden standard) and appropriate study design (Cohort)* | *Probably hospital setting and histologically diagnosed endometrial cancer* | *Little info record* |  |
| Kinkade, 2024 (adverse birth outcomes) | Definitely high RoB | Probably low RoB | Probably high RoB | Definitely low RoB | Probably high RoB | Probably high RoB | Probably low |
| Rationale | *no controls* | *No matched design, adjusted for reliable confounders (e.g., maternal age, education), most influencing factors accounted for* | *Little info recorded* | *Biological measurement with golden standard and appropriate study design (Cohort)* | *Little info recorded* | *Little info record* |  |
| Bastos-Moreira, 2025 (wasting & adverse birth outcomes) | Definitely high RoB | Probably low RoB | Probably high RoB | Definitely low RoB | Probably low RoB | Probably low RoB | Probably low |
| Rationale | *no controls* | *No matched design, adjusted for reliable confounders (e.g., maternal age, wealth index score), most influence factors accounted for* | *Little info recorded* | *Biological measurement with golden standard and appropriate study design (Cohort)* | *Probably hospital setting and WHO Child Growth Standard used* | *Relevant outcomes reported* |  |
| Chu, 2017 (liver disease) | Probably low RoB | Definitely low RoB | Probably low RoB | Definitely low RoB | Definitely low RoB | Probably low RoB | Definitely low |
| Rationale | *Age & gender matched* | *Matching was performed, adjustment for age, alcohol drinking, gender, and serum alanine, hepatitis was accounted for* | *Exclusion mentioned* | *Biological measurement (no golden standard) AND appropriate study design (NCC)* | *Cirrhosis was determined by a quantitative scoring system deriving from (i) morphological appearance of the liver surface (normal, irregular, undulated); (ii) liver parenchymal texture (normal, heterogeneous, coarse); (iii) intrahepatic vascular structure (normal, obscure, narrowing) and (iv) spleen size (normal, enlarged). diagnosis of HCC according to the following criteria: (i) histopathological confirmed HCC lesion; (ii) detection of HCC lesion by two imaging techniques (abdominal ultrasonography, angiogram, or computed tomography or (iii) detection of HCC lesion by one imaging techniques in combination with elevated serum AFP level (≥400 ng/mL)* | *Relevant outcomes reported* |  |

Note: n.a. = not applicable, MYC = mycotoxin.

# **Table 29 Detailed overview, including the criteria & rationale for decisions of the GRADE assessment of the overall body of evidence of this SR for acute food intoxication.**

|  | **CRITERIA** | **Judgement** | **Rationale** |
| --- | --- | --- | --- |
| **Initial certainty based on study design** | Assigned from study design features (Morgan et al., 2016):  1. investigator-controlled exposure?  2. exposure demonstrably prior to outcome?  3. outcome data at individual level?  4. presence of a comparison group? | Lowᵃ | 1. Not met – exposure was environmental/dietary and not investigator-controlled.  2. Met – outbreak investigations document consumption of contaminated food preceding illness, and the biomarker window coincides with the acute episode.  3. Met – exposure and outcome data were reported at the individual level.  4. Not met – most studies reported no comparison group.  2 of 4 features met → initial certainty low. |
| **Downgrade: RoB** | 1. Is the RoB considered serious? | Serious | √ Yes, most studies had high RoB (see Table 28) |
| **Downgrade: Inconsistency** | Are there different subgroups of patients with different effects? | Not serious | Х No, all ORs positive. |
| **Downgrade: Indirectness** | Are there differences that could seriously impact the magnitude of effect in:  1. populations?  2. exposure?  3. outcome?  4. comparator?  5. study design? | Not serious | Х No, little differences in studied populations: all ages, all low-income countries.  X No, mostly high aflatoxin exposure via food.  X No, all studied outcomes are related to acute aflatoxicosis.  Most studies without controls.  X No, appropriate study design for acute outbreaks  Note: item 5 (study design) is reflected in the initial certainty rating and does not contribute an additional downgrade here.ᵇ |
| **Downgrade: Imprecision** | 1. Is there an inadequate sample size (suboptimal information size, no sample size calculation, sample sizes < 100) ?  2. Is the reported 95% confidence interval broad? | Serious | √ Yes, only one study provided the population sample size able to calculate incidence, and the number of cases reported in most studies was generally low (≤ 68 cases, except for one study which reported 317 cases). None of the studies reported a sample size calculation.  √ Yes, although only two studies reported 95% confidence intervals: RR = 6.6 (95% CI: 1.2 to 16) and OR = 13.5 (95% CI: 1.5 to 165.3) most probably due to rare event and small sample size. |
| **Downgrade: Publication bias** | Are results prone to bias due to file-drawing effect? | Serious | √ Yes. Only outbreaks severe enough to trigger a public health response are investigated and published; milder or unconfirmed episodes are not. This likely inflates apparent severity and case-fatality while underestimating true burden. |
| **Upgrade: Large magnitude of effect** | Upgrade with 1 level if risk reduction ± 50% or OR ≥ 2?  Upgrade with 2 levels if risk reduction ± 80% or OR between 5 and 10? | Not applicableᶜ | Criterion met, but not applied.ᶜ Observation recorded: Calculated odds ratios indicated a higher risk of acute aflatoxicosis symptoms with values ranging from 2.2 to 5.1, at elevated aflatoxin levels (≥250 pg/mg albumin in serum). |
| **Upgrade: presence of a clear dose–response gradient** | Presence of a clear dose-response gradient? | Not applicableᶜ | Criterion not met.ᶜ Symptoms of aflatoxicosis were observed across a wide range of AF concentrations: 10 to 51,100 µg/kg in food, 36 to 209,000 pg/mg albumin in serum, and 19 to 18,521 pg/g in tissue. |
| **Upgrade: Residual confounding** | Is it plausible that all important residual confounding would reduce the observed effect, or create an effect where none is observed? | Not applicableᶜ | Criterion not met.ᶜ Except for one study, no studies controlled for confounders. |
| **Overall certainty of evidence** | High: very confident that the true effect lies close to the effect estimate.  Moderate: moderately confident that the true effect lies close to the effect estimate.  Low: confidence in the effect estimate is limited.  Very low: very little confidence in the effect estimate. | **⊕○○○**  **Very low** | Starting from low certainty on the basis of study design features, the body of evidence was downgraded for the following domains: RoB (serious); Imprecision (serious); Publication bias (serious). No serious concerns were identified in the remaining domains. Upgrading criteria were not applied because serious concerns were identified in one or more downgrading domains. The overall certainty of evidence for acute food intoxication is therefore very low: very little confidence in the effect estimates, and the true effect is likely to be substantially different from the estimate. |

*Note: AF = aflatoxin; RoB = Risk of bias.*

*ᵃ Initial certainty was assigned on the basis of study design features rather than a uniform starting point for all observational evidence, following the adaptation of GRADE for observational exposure data by Morgan et al. (2016). Bodies of evidence meeting all four features (investigator-controlled exposure; exposure demonstrably preceding the outcome; outcome data at the individual level; presence of a comparison group) begin at high certainty, with one level deducted for each feature not met. Because exposure was not investigator-controlled in any included study, the maximum attainable initial certainty in this review was moderate.*

*ᵇ To avoid double counting, the appropriateness of the study design relative to the time course of the outcome is captured in the initial certainty rating; it is therefore reported descriptively under indirectness but does not contribute an additional downgrade.*

*ᶜ In accordance with GRADE guidance, upgrading criteria are considered only where no serious concerns are identified in any downgrading domain. The observations recorded here are retained for transparency but were not applied.*

# **Table 30 Detailed overview, including the criteria & rationale for decisions of the GRADE assessment of the overall body of evidence of this SR for liver disease.**

|  | **CRITERIA** | **Judgement** | **Rationale** |
| --- | --- | --- | --- |
| **Initial certainty based on study design** | Assigned from study design features (Morgan et al., 2016):  1. investigator-controlled exposure?  2. exposure demonstrably prior to outcome?  3. outcome data at individual level?  4. presence of a comparison group? | Very lowᵃ | 1. Not met – exposure was environmental/dietary and not investigator-controlled.  2. Not met – predominantly cross-sectional studies, in which exposure and outcome were ascertained concurrently.  3. Met – exposure and outcome data were reported at the individual level.  4. Not met – most studies reported no comparison group.  1 of 4 features met → initial certainty very low. |
| **Downgrade: RoB** | 1. Is the RoB considered serious? | Not serious | X No, most studies had low RoB (see Table 28) |
| **Downgrade: Inconsistency** | Are there different subgroups of patients with different effects? | Very serious | √ Yes, ORs vary widely and there was moderate to high statistical heterogeneity for liver cirrhosis and HCC determined via plasma analysis (I2 = 45.5-82.0%). |
| **Downgrade: Indirectness** | Are there differences that could seriously impact the magnitude of effect in:  1. populations?  2. exposure?  3. outcome?  4. comparator?  5. study design? | Very serious | √ Yes, low-income versus European countries, only 36 studies matched, often no controls.  √ Yes, AFs both as parent but also as DNA mutational signature + highly different techniques, matrices, & sensitivity  X No, mostly diagnosed in hospital or medical center.  Most studies without controls.  √ Yes, mostly cross-sectional studies which are inappropriate for (mostly) chronic liver disease.  Note: item 5 (study design) is reflected in the initial certainty rating and does not contribute an additional downgrade here.ᵇ |
| **Downgrade: Imprecision** | Is there an inadequate sample size (suboptimal information size, no sample size calculation, sample sizes < 100) ?  Are the reported 95% confidence interval broad? | Serious | √ Yes, 51% has a small sample size (<100), never is a power calculation performed.  X No, the 95% confidence width varied by study, with small sample sized study having broader interval but were not broad overall. |
| **Downgrade: Publication bias** | Are results prone to bias due to file-drawering effect? | Serious | √Yes, many studies without controls (51%), only few studies report non-significant results, many studies without statistics (53 out of 102). |
| **Upgrade: Large magnitude of effect** | Upgrade with 1 level if risk reduction ± 50% or OR ≥ 2?  Upgrade with 2 levels if risk reduction ± 80% or OR between 5 and 10? | Not applicableᶜ | Criterion met, but not applied.ᶜ Observation recorded: Calculated odds ratios indicated a higher risk of liver cancer (between 1 and 20.3). |
| **Upgrade: presence of a clear dose–response gradient** | Presence of a clear dose-response gradient? | Not applicableᶜ | Criterion not met.ᶜ Large variation in measured concentrations. |
| **Upgrade: Residual confounding** | Is it plausible that all important residual confounding would reduce the observed effect, or create an effect where none is observed? | Not applicableᶜ | Criterion not met.ᶜ Only 6 studies adjusted for smoking and/or alcohol consumption and/or hepatitis antigen, and only 5 studies adjusted for confounding not specified or limited to gender or residence. |
| **Overall certainty of evidence** | High: very confident that the true effect lies close to the effect estimate.  Moderate: moderately confident that the true effect lies close to the effect estimate.  Low: confidence in the effect estimate is limited.  Very low: very little confidence in the effect estimate. | **⊕○○○**  **Very low** | Starting from very low certainty on the basis of study design features, the body of evidence was downgraded for the following domains: Inconsistency (very serious); Indirectness (very serious); Imprecision (serious); Publication bias (serious). No serious concerns were identified in the remaining domains. Upgrading criteria were not applied because serious concerns were identified in one or more downgrading domains. The overall certainty of evidence for liver disease is therefore very low: very little confidence in the effect estimates, and the true effect is likely to be substantially different from the estimate. |

*Note: AF = aflatoxin; RoB = Risk of bias.*

*ᵃ Initial certainty was assigned on the basis of study design features rather than a uniform starting point for all observational evidence, following the adaptation of GRADE for observational exposure data by Morgan et al. (2016). Bodies of evidence meeting all four features (investigator-controlled exposure; exposure demonstrably preceding the outcome; outcome data at the individual level; presence of a comparison group) begin at high certainty, with one level deducted for each feature not met. Because exposure was not investigator-controlled in any included study, the maximum attainable initial certainty in this review was moderate.*

*ᵇ To avoid double counting, the appropriateness of the study design relative to the time course of the outcome is captured in the initial certainty rating; it is therefore reported descriptively under indirectness but does not contribute an additional downgrade.*

*ᶜ In accordance with GRADE guidance, upgrading criteria are considered only where no serious concerns are identified in any downgrading domain. The observations recorded here are retained for transparency but were not applied.*

# **Table 31 Detailed overview, including the criteria & rationale for decisions of the GRADE assessment of the overall body of evidence of this SR for gallbladder cancer.**

|  | **CRITERIA** | **Judgement** | **Rationale** |
| --- | --- | --- | --- |
| **Initial certainty based on study design** | Assigned from study design features (Morgan et al., 2016):  1. investigator-controlled exposure?  2. exposure demonstrably prior to outcome?  3. outcome data at individual level?  4. presence of a comparison group? | Moderateᵃ | 1. Not met – exposure was environmental/dietary and not investigator-controlled.  2. Met – case–control studies in which exposure status was related to a previously ascertained diagnosis.  3. Met – exposure and outcome data were reported at the individual level.  4. Met – all studies included controls matched for age, sex and/or origin.  3 of 4 features met → initial certainty moderate. |
| **Downgrade: RoB** | 1. Is the RoB considered serious? | Not serious | X No, most studies had low RoB (see Table 28) |
| **Downgrade: Inconsistency** | Are there different subgroups of patients with different effects? | Very serious | √ Yes, OR vary widely and there was substantial statistical heterogeneity (I2 = 63.5%). |
| **Downgrade: Indirectness** | Are there differences that could seriously impact the magnitude of effect in:  1. populations?  2. exposure?  3. outcome?  4. comparator?  5. study design? | Not serious | X No, all adults, no low-income countries, all controlled.  X No, all AFB1-lysine in serum/plasma.  X No, all diagnosed in hospital setting.  X No, all studies had controls that were matched for at age, sex and/or origin.  X No, most studies had appropriate study design  Note: item 5 (study design) is reflected in the initial certainty rating and does not contribute an additional downgrade here.ᵇ |
| **Downgrade: Imprecision** | Is there an inadequate sample size (suboptimal information size, no sample size calculation, sample sizes < 100) ?  Are the reported 95% confidence interval broad? | Serious | X No, 2 out of 3 studies with a sample size >100.  √ Yes, RR reported ranges of 0.6–194.5 and 2.1–653.3 |
| **Downgrade: Publication bias** | Are results prone to bias due to file-drawering effect? | Not serious | X No, controlled studies and non-significant results are mentioned. |
| **Upgrade: Large magnitude of effect** | Upgrade with 1 level if risk reduction ± 50% or OR ≥ 2?  Upgrade with 2 levels if risk reduction ± 80% or OR between 5 and 10? | Not applicableᶜ | Criterion met, but not applied.ᶜ Observation recorded: Calculated odds ratios indicated a higher risk of liver cancer (between 3 and 13). |
| **Upgrade: presence of a clear dose–response gradient** | Presence of a clear dose-response gradient? | Not applicableᶜ | Criterion met, but not applied.ᶜ Observation recorded: Comparable concentrations. |
| **Upgrade: Residual confounding** | Is it plausible that all important residual confounding would reduce the observed effect, or create an effect where none is observed? | Not applicableᶜ | Criterion not met.ᶜ Although controlled for age and gender, not for nutrition or other potential variations. |
| **Overall certainty of evidence** | High: very confident that the true effect lies close to the effect estimate.  Moderate: moderately confident that the true effect lies close to the effect estimate.  Low: confidence in the effect estimate is limited.  Very low: very little confidence in the effect estimate. | **⊕○○○**  **Very low** | Starting from moderate certainty on the basis of study design features, the body of evidence was downgraded for the following domains: Inconsistency (very serious); Imprecision (serious). No serious concerns were identified in the remaining domains. Upgrading criteria were not applied because serious concerns were identified in one or more downgrading domains. The overall certainty of evidence for gallbladder cancer is therefore very low: very little confidence in the effect estimates, and the true effect is likely to be substantially different from the estimate. |

*Note: AF = aflatoxin; RoB = Risk of bias; LC-MS/MS = liquid chromatography tandem mass spectrometry; ELISA = Enzyme-Linked Immunosorbent Assay; HPLC-FLD = High-Performance Liquid Chromatography with Fluorescence Detection.*

*ᵃ Initial certainty was assigned on the basis of study design features rather than a uniform starting point for all observational evidence, following the adaptation of GRADE for observational exposure data by Morgan et al. (2016). Bodies of evidence meeting all four features (investigator-controlled exposure; exposure demonstrably preceding the outcome; outcome data at the individual level; presence of a comparison group) begin at high certainty, with one level deducted for each feature not met. Because exposure was not investigator-controlled in any included study, the maximum attainable initial certainty in this review was moderate.*

*ᵇ To avoid double counting, the appropriateness of the study design relative to the time course of the outcome is captured in the initial certainty rating; it is therefore reported descriptively under indirectness but does not contribute an additional downgrade.*

*ᶜ In accordance with GRADE guidance, upgrading criteria are considered only where no serious concerns are identified in any downgrading domain. The observations recorded here are retained for transparency but were not applied.*

# **Table 32 Detailed overview, including the criteria & rationale for decisions of the GRADE assessment of the overall body of evidence of this SR for metabolic disease.**

|  | **CRITERIA** | **Judgement** | **Rationale** |
| --- | --- | --- | --- |
| **Initial certainty based on study design** | Assigned from study design features (Morgan et al., 2016):  1. investigator-controlled exposure?  2. exposure demonstrably prior to outcome?  3. outcome data at individual level?  4. presence of a comparison group? | Very lowᵃ | 1. Not met – exposure was environmental/dietary and not investigator-controlled.  2. Not met – most studies applied a design that does not establish temporality.  3. Met – exposure and outcome data were reported at the individual level.  4. Not met – most studies reported no comparison group.  1 of 4 features met → initial certainty very low. |
| **Downgrade: RoB** | 1. Is the RoB considered serious? | Not serious | X No, most studies had low RoB (see Table 28) |
| **Downgrade: Inconsistency** | Are there different subgroups of patients with different effects? | Serious | √ Yes, OR vary widely. |
| **Downgrade: Indirectness** | Are there differences that could seriously impact the magnitude of effect in:  1. populations?  2. exposure?  3. outcome?  4. comparator?  5. study design? | Very serious | √ Yes, all adults, low-, middle and high-income countries.  √ Yes, AF or OTA in serum/plasma/urine/cord blood.  √ Yes, only few in hospital setting.  Most studies without controls.  √ Yes, most studies had inappropriate study design.  Note: item 5 (study design) is reflected in the initial certainty rating and does not contribute an additional downgrade here.ᵇ |
| **Downgrade: Imprecision** | Is there an inadequate sample size (suboptimal information size, no sample size calculation, sample sizes < 100) ?  Are the reported 95% confidence interval broad? | Not serious | X No, most studies sample size >100.  X No, broadest 95% CI was 0.53 - 0.67 |
| **Downgrade: Publication bias** | Are results prone to bias due to file-drawering effect? | Serious | √ Yes, only few controlled, no mention of non-significant results. |
| **Upgrade: Large magnitude of effect** | Upgrade with 1 level if risk reduction ± 50% or OR ≥ 2?  Upgrade with 2 levels if risk reduction ± 80% or OR between 5 and 10? | Not applicableᶜ | Criterion met, but not applied.ᶜ Observation recorded: Calculated odds ratios indicated a higher risk of diabetes (between 1 and 10), but lower for metabolic syndrome. |
| **Upgrade: presence of a clear dose–response gradient** | Presence of a clear dose-response gradient? | Not applicableᶜ | Criterion met, but not applied.ᶜ Observation recorded: Comparable concentrations. |
| **Upgrade: Residual confounding** | Is it plausible that all important residual confounding would reduce the observed effect, or create an effect where none is observed? | Not applicableᶜ | Criterion not met.ᶜ Only few controlled and not taking other factors such as nutrition into account. |
| **Overall certainty of evidence** | High: very confident that the true effect lies close to the effect estimate.  Moderate: moderately confident that the true effect lies close to the effect estimate.  Low: confidence in the effect estimate is limited.  Very low: very little confidence in the effect estimate. | **⊕○○○**  **Very low** | Starting from very low certainty on the basis of study design features, the body of evidence was downgraded for the following domains: Inconsistency (serious); Indirectness (very serious); Publication bias (serious). No serious concerns were identified in the remaining domains. Upgrading criteria were not applied because serious concerns were identified in one or more downgrading domains. The overall certainty of evidence for metabolic disease is therefore very low: very little confidence in the effect estimates, and the true effect is likely to be substantially different from the estimate. |

*Note: AF = aflatoxin; OTA = ochratoxin A; RoB = Risk of bias; ELISA = Enzyme-Linked Immunosorbent Assay; HPLC-FLD = High-Performance Liquid Chromatography with Fluorescence Detection; IDMS = Isotope Dilution Mass Spectrometry.*

*ᵃ Initial certainty was assigned on the basis of study design features rather than a uniform starting point for all observational evidence, following the adaptation of GRADE for observational exposure data by Morgan et al. (2016). Bodies of evidence meeting all four features (investigator-controlled exposure; exposure demonstrably preceding the outcome; outcome data at the individual level; presence of a comparison group) begin at high certainty, with one level deducted for each feature not met. Because exposure was not investigator-controlled in any included study, the maximum attainable initial certainty in this review was moderate.*

*ᵇ To avoid double counting, the appropriateness of the study design relative to the time course of the outcome is captured in the initial certainty rating; it is therefore reported descriptively under indirectness but does not contribute an additional downgrade.*

*ᶜ In accordance with GRADE guidance, upgrading criteria are considered only where no serious concerns are identified in any downgrading domain. The observations recorded here are retained for transparency but were not applied.*

# **Table 33 Detailed overview, including the criteria & rationale for decisions of the GRADE assessment of the overall body of evidence of this SR for neurological disease.**

|  | **CRITERIA** | **Judgement** | **Rationale** |
| --- | --- | --- | --- |
| **Initial certainty based on study design** | Assigned from study design features (Morgan et al., 2016):  1. investigator-controlled exposure?  2. exposure demonstrably prior to outcome?  3. outcome data at individual level?  4. presence of a comparison group? | Lowᵃ | 1. Not met – exposure was environmental/dietary and not investigator-controlled.  2. Not met – most studies applied a design that does not establish temporality.  3. Met – exposure and outcome data were reported at the individual level.  4. Met – most studies included controls matched for age, sex and/or origin.  2 of 4 features met → initial certainty low. |
| **Downgrade: RoB** | 1. Is the RoB considered serious? | Not serious | X No, most studies had low RoB (see Table 28) |
| **Downgrade: Inconsistency** | Are there different subgroups of patients with different effects? | Not serious | X No, all CCs are negative. |
| **Downgrade: Indirectness** | Are there differences that could seriously impact the magnitude of effect in:  1. populations?  2. exposure?  3. outcome?  4. comparator?  5. study design? | Very serious | √ Yes, children and adults, low-, middle and high-income countries.  √ Yes, multiple mycotoxins in serum/plasma/urine/tissue.  √ Yes, only few in hospital setting.  X No, most studies with controls matched for age, sex and/or origin.  √ Yes, mostly inappropriate study design  Note: item 5 (study design) is reflected in the initial certainty rating and does not contribute an additional downgrade here.ᵇ |
| **Downgrade: Imprecision** | Is there an inadequate sample size (suboptimal information size, no sample size calculation, sample sizes < 100) ?  Are the reported 95% confidence interval broad? | Serious | √ Yes, most studies sample size <100.  X No, only one study reported 95% confidence interval which was not broad. |
| **Downgrade: Publication bias** | Are results prone to bias due to file-drawering effect? | Serious | √ Yes, half of studies controlled but also no mention of non-significant results. |
| **Upgrade: Large magnitude of effect** | Upgrade with 1 level if risk reduction ± 50% or OR ≥ 2?  Upgrade with 2 levels if risk reduction ± 80% or OR between 5 and 10? | Not applicableᶜ | Criterion not met.ᶜ CC are negative. |
| **Upgrade: presence of a clear dose–response gradient** | Presence of a clear dose-response gradient? | Not applicableᶜ | Criterion not met.ᶜ Highly variable concentrations. |
| **Upgrade: Residual confounding** | Is it plausible that all important residual confounding would reduce the observed effect, or create an effect where none is observed? | Not applicableᶜ | Criterion not met.ᶜ Only few controlled and not taking other factors such as nutrition into account. |
| **Overall certainty of evidence** | High: very confident that the true effect lies close to the effect estimate.  Moderate: moderately confident that the true effect lies close to the effect estimate.  Low: confidence in the effect estimate is limited.  Very low: very little confidence in the effect estimate. | **⊕○○○**  **Very low** | Starting from low certainty on the basis of study design features, the body of evidence was downgraded for the following domains: Indirectness (very serious); Imprecision (serious); Publication bias (serious). No serious concerns were identified in the remaining domains. Upgrading criteria were not applied because serious concerns were identified in one or more downgrading domains. The overall certainty of evidence for neurological disease is therefore very low: very little confidence in the effect estimates, and the true effect is likely to be substantially different from the estimate. |

*Note: AF = aflatoxin; RoB = Risk of bias; LC-MS/MS = liquid chromatography tandem mass spectrometry; ELISA = Enzyme-Linked Immunosorbent Assay; HPLC-FLD = High-Performance Liquid Chromatography with Fluorescence Detection.*

*ᵃ Initial certainty was assigned on the basis of study design features rather than a uniform starting point for all observational evidence, following the adaptation of GRADE for observational exposure data by Morgan et al. (2016). Bodies of evidence meeting all four features (investigator-controlled exposure; exposure demonstrably preceding the outcome; outcome data at the individual level; presence of a comparison group) begin at high certainty, with one level deducted for each feature not met. Because exposure was not investigator-controlled in any included study, the maximum attainable initial certainty in this review was moderate.*

*ᵇ To avoid double counting, the appropriateness of the study design relative to the time course of the outcome is captured in the initial certainty rating; it is therefore reported descriptively under indirectness but does not contribute an additional downgrade.*

*ᶜ In accordance with GRADE guidance, upgrading criteria are considered only where no serious concerns are identified in any downgrading domain. The observations recorded here are retained for transparency but were not applied.*

# **Table 34 Detailed overview, including the criteria & rationale for decisions of the GRADE assessment of the overall body of evidence of this SR for male reproductive disease.**

|  | **CRITERIA** | **Judgement** | **Rationale** |
| --- | --- | --- | --- |
| **Initial certainty based on study design** | Assigned from study design features (Morgan et al., 2016):  1. investigator-controlled exposure?  2. exposure demonstrably prior to outcome?  3. outcome data at individual level?  4. presence of a comparison group? | Very lowᵃ | 1. Not met – exposure was environmental/dietary and not investigator-controlled.  2. Not met – most studies applied a design that does not establish temporality.  3. Met – exposure and outcome data were reported at the individual level.  4. Not met – most studies reported no comparison group.  1 of 4 features met → initial certainty very low. |
| **Downgrade: RoB** | 1. Is the RoB considered serious? | Not serious | X No, most studies had low RoB (see Table 28) |
| **Downgrade: Inconsistency** | Are there different subgroups of patients with different effects? | Serious | √ Yes, detects versus non-detects. |
| **Downgrade: Indirectness** | Are there differences that could seriously impact the magnitude of effect in:  1. populations?  2. exposure?  3. outcome?  4. comparator?  5. study design? | Very serious | √ Yes, differential age, low-, middle and high-income countries.  √ Yes, multiple mycotoxins in serum/plasma/urine/tissue/semen.  √ Yes, only 1 in hospital setting.  Most studies without controls.  √ Yes, mostly inappropriate study design (def. for infertility)  Note: item 5 (study design) is reflected in the initial certainty rating and does not contribute an additional downgrade here.ᵇ |
| **Downgrade: Imprecision** | Is there an inadequate sample size (suboptimal information size, no sample size calculation, sample sizes < 100) ?  Are the reported 95% confidence interval broad? | Serious | √ Yes, most studies sample size <100.  X No, none reported. |
| **Downgrade: Publication bias** | Are results prone to bias due to file-drawering effect? | Not serious | X No, only a few controlled but there is mention of non-significant results. |
| **Upgrade: Large magnitude of effect** | Upgrade with 1 level if risk reduction ± 50% or OR ≥ 2?  Upgrade with 2 levels if risk reduction ± 80% or OR between 5 and 10? | Not applicableᶜ | Criterion not met.ᶜ Prevalences vary widely. |
| **Upgrade: presence of a clear dose–response gradient** | Presence of a clear dose-response gradient? | Not applicableᶜ | Criterion not met.ᶜ Highly variable concentrations. |
| **Upgrade: Residual confounding** | Is it plausible that all important residual confounding would reduce the observed effect, or create an effect where none is observed? | Not applicableᶜ | Criterion not met.ᶜ Only few controlled and not taking other factors such as nutrition into account. |
| **Overall certainty of evidence** | High: very confident that the true effect lies close to the effect estimate.  Moderate: moderately confident that the true effect lies close to the effect estimate.  Low: confidence in the effect estimate is limited.  Very low: very little confidence in the effect estimate. | **⊕○○○**  **Very low** | Starting from very low certainty on the basis of study design features, the body of evidence was downgraded for the following domains: Inconsistency (serious); Indirectness (very serious); Imprecision (serious). No serious concerns were identified in the remaining domains. Upgrading criteria were not applied because serious concerns were identified in one or more downgrading domains. The overall certainty of evidence for male reproductive disease is therefore very low: very little confidence in the effect estimates, and the true effect is likely to be substantially different from the estimate. |

*Note: AF = aflatoxin; RoB = Risk of bias; FM = ; LC-MS/MS = liquid chromatography tandem mass spectrometry; ELISA = Enzyme-Linked Immunosorbent Assay; IHC = Immunohistochemistry; FM = Fluorescence measurement.*

*ᵃ Initial certainty was assigned on the basis of study design features rather than a uniform starting point for all observational evidence, following the adaptation of GRADE for observational exposure data by Morgan et al. (2016). Bodies of evidence meeting all four features (investigator-controlled exposure; exposure demonstrably preceding the outcome; outcome data at the individual level; presence of a comparison group) begin at high certainty, with one level deducted for each feature not met. Because exposure was not investigator-controlled in any included study, the maximum attainable initial certainty in this review was moderate.*

*ᵇ To avoid double counting, the appropriateness of the study design relative to the time course of the outcome is captured in the initial certainty rating; it is therefore reported descriptively under indirectness but does not contribute an additional downgrade.*

*ᶜ In accordance with GRADE guidance, upgrading criteria are considered only where no serious concerns are identified in any downgrading domain. The observations recorded here are retained for transparency but were not applied.*

# **Table 35 Detailed overview, including the criteria & rationale for decisions of the GRADE assessment of the overall body of evidence of this SR for female reproductive disease.**

|  | **CRITERIA** | **Judgement** | **Rationale** |
| --- | --- | --- | --- |
| **Initial certainty based on study design** | Assigned from study design features (Morgan et al., 2016):  1. investigator-controlled exposure?  2. exposure demonstrably prior to outcome?  3. outcome data at individual level?  4. presence of a comparison group? | Very lowᵃ | 1. Not met – exposure was environmental/dietary and not investigator-controlled.  2. Not met – most studies applied a design that does not establish temporality.  3. Met – exposure and outcome data were reported at the individual level.  4. Not met – most studies reported no comparison group.  1 of 4 features met → initial certainty very low. |
| **Downgrade: RoB** | 1. Is the RoB considered serious? | Not serious | X No, most studies had low RoB (see Table 28) |
| **Downgrade: Inconsistency** | 1. Are there different subgroups of patients with different effects? | Serious | √ Yes, prevalence varies. |
| **Downgrade: Indirectness** | Are there differences that could seriously impact the magnitude of effect in:  1. populations?  2. exposure?  3. outcome?  4. comparator?  5. study design? | Not serious | X No mostly adults in high-income countries.  X No, mostly ZEN + metabolites in blood.  X No, mostly in hospital setting.  Mostly without controls.  √ Yes, mostly inappropriate study design  Note: item 5 (study design) is reflected in the initial certainty rating and does not contribute an additional downgrade here.ᵇ |
| **Downgrade: Imprecision** | Is there an inadequate sample size (suboptimal information size, no sample size calculation, sample sizes < 100) ?  Are the reported 95% confidence interval broad? | Serious | √ Yes, most studies sample size <100.  X No, none reported. |
| **Downgrade: Publication bias** | Are results prone to bias due to file-drawering effect? | Not serious | X No, also reports of non-detects + some controlled. |
| **Upgrade: Large magnitude of effect** | Upgrade with 1 level if risk reduction ± 50% or OR ≥ 2?  Upgrade with 2 levels if risk reduction ± 80% or OR between 5 and 10? | Not applicableᶜ | Criterion met, but not applied.ᶜ Observation recorded: Between 1 and 10 for breast cancer. |
| **Upgrade: presence of a clear dose–response gradient** | Presence of a clear dose-response gradient? | Not applicableᶜ | Criterion not met.ᶜ Variable concentrations. |
| **Upgrade: Residual confounding** | Is it plausible that all important residual confounding would reduce the observed effect, or create an effect where none is observed? | Not applicableᶜ | Criterion not met.ᶜ Only few controlled and not taking other factors such as nutrition into account. |
| **Overall certainty of evidence** | High: very confident that the true effect lies close to the effect estimate.  Moderate: moderately confident that the true effect lies close to the effect estimate.  Low: confidence in the effect estimate is limited.  Very low: very little confidence in the effect estimate. | **⊕○○○**  **Very low** | Starting from very low certainty on the basis of study design features, the body of evidence was downgraded for the following domains: Inconsistency (serious); Imprecision (serious). No serious concerns were identified in the remaining domains. Upgrading criteria were not applied because serious concerns were identified in one or more downgrading domains. The overall certainty of evidence for female reproductive disease is therefore very low: very little confidence in the effect estimates, and the true effect is likely to be substantially different from the estimate. |

*Note: ZEN = zearalenone; RoB = Risk of bias.*

*ᵃ Initial certainty was assigned on the basis of study design features rather than a uniform starting point for all observational evidence, following the adaptation of GRADE for observational exposure data by Morgan et al. (2016). Bodies of evidence meeting all four features (investigator-controlled exposure; exposure demonstrably preceding the outcome; outcome data at the individual level; presence of a comparison group) begin at high certainty, with one level deducted for each feature not met. Because exposure was not investigator-controlled in any included study, the maximum attainable initial certainty in this review was moderate.*

*ᵇ To avoid double counting, the appropriateness of the study design relative to the time course of the outcome is captured in the initial certainty rating; it is therefore reported descriptively under indirectness but does not contribute an additional downgrade.*

*ᶜ In accordance with GRADE guidance, upgrading criteria are considered only where no serious concerns are identified in any downgrading domain. The observations recorded here are retained for transparency but were not applied.*

# **Table 36 Detailed overview, including the criteria & rationale for decisions of the GRADE assessment of the overall body of evidence of this SR for premature puberty.**

|  | **CRITERIA** | **Judgement** | **Rationale** |
| --- | --- | --- | --- |
| **Initial certainty based on study design** | Assigned from study design features (Morgan et al., 2016):  1. investigator-controlled exposure?  2. exposure demonstrably prior to outcome?  3. outcome data at individual level?  4. presence of a comparison group? | Lowᵃ | 1. Not met – exposure was environmental/dietary and not investigator-controlled.  2. Not met – most studies applied a design that does not establish temporality.  3. Met – exposure and outcome data were reported at the individual level.  4. Met – most studies included well-matched controls.  2 of 4 features met → initial certainty low. |
| **Downgrade: RoB** | 1. Is the RoB considered serious? | Not serious | X No, most studies had low RoB (see Table 28) |
| **Downgrade: Inconsistency** | 1. Are there different subgroups of patients with different effects?  2. Was the outcome differently defined between studies? | Not serious | X No, ORs >5. |
| **Downgrade: Indirectness** | Are there differences that could seriously impact the magnitude of effect in:  1. populations?  2. exposure?  3. outcome?  4. comparator?  5. study design? | Not serious | X No, female children from high-income countries.  X No, mostly ZEN + metabolites in blood/urine.  X No, all in-house/study center.  X No, mostly with well-matched controls.  √ Yes, mostly inappropriate study design.  Note: item 5 (study design) is reflected in the initial certainty rating and does not contribute an additional downgrade here.ᵇ |
| **Downgrade: Imprecision** | Is there an inadequate sample size (suboptimal information size, no sample size calculation, sample sizes < 100) ?  Are the reported 95% confidence interval broad? | Serious | √ Yes, most studies sample size <100.  X No, all reported 95% confidence intervals were not broad. |
| **Downgrade: Publication bias** | Are results prone to bias due to file-drawering effect? | Not serious | X No, also reports of non-detects + some controlled. |
| **Upgrade: Large magnitude of effect** | Upgrade with 1 level if risk reduction ± 50% or OR ≥ 2?  Upgrade with 2 levels if risk reduction ± 80% or OR between 5 and 10? | Not applicableᶜ | Criterion met, but not applied.ᶜ Observation recorded: ORs between 6 and 9. |
| **Upgrade: presence of a clear dose–response gradient** | Presence of a clear dose-response gradient? | Not applicableᶜ | Criterion met, but not applied.ᶜ Observation recorded: Similar detected concentrations. |
| **Upgrade: Residual confounding** | Is it plausible that all important residual confounding would reduce the observed effect, or create an effect where none is observed? | Not applicableᶜ | Criterion met, but not applied.ᶜ Observation recorded: Adjusted for confounding and study design allowed only one gender and age range. |
| **Overall certainty of evidence** | High: very confident that the true effect lies close to the effect estimate.  Moderate: moderately confident that the true effect lies close to the effect estimate.  Low: confidence in the effect estimate is limited.  Very low: very little confidence in the effect estimate. | **⊕○○○**  **Very low** | Starting from low certainty on the basis of study design features, the body of evidence was downgraded for the following domains: Imprecision (serious). No serious concerns were identified in the remaining domains. Upgrading criteria were not applied because serious concerns were identified in one or more downgrading domains. The overall certainty of evidence for premature puberty is therefore very low: very little confidence in the effect estimates, and the true effect is likely to be substantially different from the estimate. |

*Note: ZEN = zearalenone; OR = odds ratio; RoB = Risk of bias, HPLC-MS/MS = ultrahigh performance liquid chromatography tandem mass spectrometry.*

*ᵃ Initial certainty was assigned on the basis of study design features rather than a uniform starting point for all observational evidence, following the adaptation of GRADE for observational exposure data by Morgan et al. (2016). Bodies of evidence meeting all four features (investigator-controlled exposure; exposure demonstrably preceding the outcome; outcome data at the individual level; presence of a comparison group) begin at high certainty, with one level deducted for each feature not met. Because exposure was not investigator-controlled in any included study, the maximum attainable initial certainty in this review was moderate.*

*ᵇ To avoid double counting, the appropriateness of the study design relative to the time course of the outcome is captured in the initial certainty rating; it is therefore reported descriptively under indirectness but does not contribute an additional downgrade.*

*ᶜ In accordance with GRADE guidance, upgrading criteria are considered only where no serious concerns are identified in any downgrading domain. The observations recorded here are retained for transparency but were not applied.*

# **Table 37 Detailed overview, including the criteria & rationale for decisions of the GRADE assessment of the overall body of evidence of this SR for kidney disease.**

|  | **CRITERIA** | **Judgement** | **Rationale** |
| --- | --- | --- | --- |
| **Initial certainty based on study design** | Assigned from study design features (Morgan et al., 2016):  1. investigator-controlled exposure?  2. exposure demonstrably prior to outcome?  3. outcome data at individual level?  4. presence of a comparison group? | Lowᵃ | 1. Not met – exposure was environmental/dietary and not investigator-controlled.  2. Not met – most studies applied a design that does not establish temporality.  3. Met – exposure and outcome data were reported at the individual level.  4. Met – most studies included controls matched for age, sex and/or origin.  2 of 4 features met → initial certainty low. |
| **Downgrade: RoB** | 1. Is the RoB considered serious? | Serious | √ Yes, most studies had high RoB (see Table 28) |
| **Downgrade: Inconsistency** | Are there different subgroups of patients with different effects? | Serious | √ Yes, prevalence varies widely. |
| **Downgrade: Indirectness** | Are there differences that could seriously impact the magnitude of effect in:  1. populations?  2. exposure?  3. outcome?  4. comparator?  5. study design? | Very serious | √ Yes, children and adults, from low-, middle- and high-income countries.  √ Yes, OTA, AFs, multiple in urine, blood, tissue.  X No, mostly diagnosed in hospital.  X No, mostly controlled and matched for age, sex and/or origin.  √ Yes, mostly inappropriate study design.  Note: item 5 (study design) is reflected in the initial certainty rating and does not contribute an additional downgrade here.ᵇ |
| **Downgrade: Imprecision** | Is there an inadequate sample size (suboptimal information size, no sample size calculation, sample sizes < 100) ?  Are the reported 95% confidence interval broad? | Not serious | X No, most studies sample size >100.  X No, none reported. |
| **Downgrade: Publication bias** | Are results prone to bias due to file-drawering effect? | Not serious | X No, also reports of non-detects + some controlled. |
| **Upgrade: Large magnitude of effect** | Upgrade with 1 level if risk reduction ± 50% or OR ≥ 2?  Upgrade with 2 levels if risk reduction ± 80% or OR between 5 and 10? | Not applicableᶜ | Criterion not met.ᶜ ORs and CC <1. |
| **Upgrade: presence of a clear dose–response gradient** | Presence of a clear dose-response gradient? | Not applicableᶜ | Criterion not met.ᶜ Concentrations varies within studies and within-matrices. |
| **Upgrade: Residual confounding** | Is it plausible that all important residual confounding would reduce the observed effect, or create an effect where none is observed? | Not applicableᶜ | Criterion not met.ᶜ Often no controls and only 1 study matched for confounding. |
| **Overall certainty of evidence** | High: very confident that the true effect lies close to the effect estimate.  Moderate: moderately confident that the true effect lies close to the effect estimate.  Low: confidence in the effect estimate is limited.  Very low: very little confidence in the effect estimate. | **⊕○○○**  **Very low** | Starting from low certainty on the basis of study design features, the body of evidence was downgraded for the following domains: RoB (serious); Inconsistency (serious); Indirectness (very serious). No serious concerns were identified in the remaining domains. Upgrading criteria were not applied because serious concerns were identified in one or more downgrading domains. The overall certainty of evidence for kidney disease is therefore very low: very little confidence in the effect estimates, and the true effect is likely to be substantially different from the estimate. |

*Note: AF = aflatoxin; OTA = ochratoxin A; OR = odds ratio; RoB = Risk of bias; LC-MS/MS = liquid chromatography tandem mass spectrometry; ELISA = Enzyme-Linked Immunosorbent Assay; HPLC-FLD = High-Performance Liquid Chromatography with Fluorescence Detection.*

*ᵃ Initial certainty was assigned on the basis of study design features rather than a uniform starting point for all observational evidence, following the adaptation of GRADE for observational exposure data by Morgan et al. (2016). Bodies of evidence meeting all four features (investigator-controlled exposure; exposure demonstrably preceding the outcome; outcome data at the individual level; presence of a comparison group) begin at high certainty, with one level deducted for each feature not met. Because exposure was not investigator-controlled in any included study, the maximum attainable initial certainty in this review was moderate.*

*ᵇ To avoid double counting, the appropriateness of the study design relative to the time course of the outcome is captured in the initial certainty rating; it is therefore reported descriptively under indirectness but does not contribute an additional downgrade.*

*ᶜ In accordance with GRADE guidance, upgrading criteria are considered only where no serious concerns are identified in any downgrading domain. The observations recorded here are retained for transparency but were not applied.*

# **Table 38 Detailed overview, including the criteria & rationale for decisions of the GRADE assessment of the overall body of evidence of this SR for bladder cancer.**

|  | **CRITERIA** | **Judgement** | **Rationale** |
| --- | --- | --- | --- |
| **Initial certainty based on study design** | Assigned from study design features (Morgan et al., 2016):  1. investigator-controlled exposure?  2. exposure demonstrably prior to outcome?  3. outcome data at individual level?  4. presence of a comparison group? | Lowᵃ | 1. Not met – exposure was environmental/dietary and not investigator-controlled.  2. Not met – the designs applied do not establish that exposure preceded diagnosis.  3. Met – exposure and outcome data were reported at the individual level.  4. Met – all studies included a comparison group, although matching was frequently inadequate.  2 of 4 features met → initial certainty low. |
| **Downgrade: RoB** | 1. Is the RoB considered serious? | Serious | √ Yes, most studies had high RoB (see Table 28) |
| **Downgrade: Inconsistency** | Are there different subgroups of patients with different effects? | Serious | √ Yes, prevalence varies. |
| **Downgrade: Indirectness** | Are there differences that could seriously impact the magnitude of effect in:  1. populations?  2. exposure?  3. outcome?  4. comparator?  5. study design? | Serious | X No, mostly lower-income countries, adults.  X No, OTA in blood.  X No, mostly diagnosed in hospital.  √ Yes, most controls not well-matched.  √ Yes, overall inappropriate study design.  Note: item 5 (study design) is reflected in the initial certainty rating and does not contribute an additional downgrade here.ᵇ |
| **Downgrade: Imprecision** | Is there an inadequate sample size (suboptimal information size, no sample size calculation, sample sizes < 100) ?  Are the reported 95% confidence interval broad? | Not serious | X No, most studies sample size >100.  X No, none reported. |
| **Downgrade: Publication bias** | Are results prone to bias due to file-drawering effect? | Not serious | X No, also reports of non-detects + all controlled studies. |
| **Upgrade: Large magnitude of effect** | Upgrade with 1 level if risk reduction ± 50% or OR ≥ 2?  Upgrade with 2 levels if risk reduction ± 80% or OR between 5 and 10? | Not applicableᶜ | Criterion not met.ᶜ No effect sizes and prevalence between 40 and 77%. |
| **Upgrade: presence of a clear dose–response gradient** | Presence of a clear dose-response gradient? | Not applicableᶜ | Criterion met, but not applied.ᶜ Observation recorded: Found concentrations are comparable. |
| **Upgrade: Residual confounding** | Is it plausible that all important residual confounding would reduce the observed effect, or create an effect where none is observed? | Not applicableᶜ | Criterion not met.ᶜ Almost no matching, few controls. |
| **Overall certainty of evidence** | High: very confident that the true effect lies close to the effect estimate.  Moderate: moderately confident that the true effect lies close to the effect estimate.  Low: confidence in the effect estimate is limited.  Very low: very little confidence in the effect estimate. | **⊕○○○**  **Very low** | Starting from low certainty on the basis of study design features, the body of evidence was downgraded for the following domains: RoB (serious); Inconsistency (serious); Indirectness (serious). No serious concerns were identified in the remaining domains. Upgrading criteria were not applied because serious concerns were identified in one or more downgrading domains. The overall certainty of evidence for bladder cancer is therefore very low: very little confidence in the effect estimates, and the true effect is likely to be substantially different from the estimate. |

*Note: AF = aflatoxin; OTA = ochratoxin A; RoB = Risk of bias; HPLC-FLD = High-Performance Liquid Chromatography with Fluorescence Detection.*

*ᵃ Initial certainty was assigned on the basis of study design features rather than a uniform starting point for all observational evidence, following the adaptation of GRADE for observational exposure data by Morgan et al. (2016). Bodies of evidence meeting all four features (investigator-controlled exposure; exposure demonstrably preceding the outcome; outcome data at the individual level; presence of a comparison group) begin at high certainty, with one level deducted for each feature not met. Because exposure was not investigator-controlled in any included study, the maximum attainable initial certainty in this review was moderate.*

*ᵇ To avoid double counting, the appropriateness of the study design relative to the time course of the outcome is captured in the initial certainty rating; it is therefore reported descriptively under indirectness but does not contribute an additional downgrade.*

*ᶜ In accordance with GRADE guidance, upgrading criteria are considered only where no serious concerns are identified in any downgrading domain. The observations recorded here are retained for transparency but were not applied.*

# **Table 39 Detailed overview, including the criteria & rationale for decisions of the GRADE assessment of the overall body of evidence of this SR for lung disease.**

|  | **CRITERIA** | **Judgement** | **Rationale** |
| --- | --- | --- | --- |
| **Initial certainty based on study design** | Assigned from study design features (Morgan et al., 2016):  1. investigator-controlled exposure?  2. exposure demonstrably prior to outcome?  3. outcome data at individual level?  4. presence of a comparison group? | Very lowᵃ | 1. Not met – exposure was environmental/dietary and not investigator-controlled.  2. Not met – most studies applied a design that does not establish temporality.  3. Met – exposure and outcome data were reported at the individual level.  4. Not met – most studies reported no comparison group.  1 of 4 features met → initial certainty very low. |
| **Downgrade: RoB** | 1. Is the RoB considered serious? | Not serious | X No, most studies had low RoB (see Table 28) |
| **Downgrade: Inconsistency** | Are there different subgroups of patients with different effects? | Serious | √ Yes, prevalence varies. |
| **Downgrade: Indirectness** | Are there differences that could seriously impact the magnitude of effect in:  1. populations?  2. exposure?  3. outcome?  4. comparator?  5. study design? | Very serious | X No, mostly lower-income countries, adults.  √ Yes, AFs but in blood and tissue.  √ Yes, outbreaks not in a hospital setting.  Mostly without controls.  √ Yes, mostly inappropriate study design for lung cancer.  Note: item 5 (study design) is reflected in the initial certainty rating and does not contribute an additional downgrade here.ᵇ |
| **Downgrade: Imprecision** | Is there an inadequate sample size (suboptimal information size, no sample size calculation, sample sizes < 100) ?  Are the reported 95% confidence interval broad? | Serious | √ Yes, most studies sample size <100.  X No, none reported. |
| **Downgrade: Publication bias** | Are results prone to bias due to file-drawering effect? | Serious | √ Yes, hospital admission biases, favoring individuals with the financial means, typical in outbreak settings in low-income countries. |
| **Upgrade: Large magnitude of effect** | Upgrade with 1 level if risk reduction ± 50% or OR ≥ 2?  Upgrade with 2 levels if risk reduction ± 80% or OR between 5 and 10? | Not applicableᶜ | Criterion not met.ᶜ Prevalence between 0.7 and 25%. |
| **Upgrade: presence of a clear dose–response gradient** | Presence of a clear dose-response gradient? | Not applicableᶜ | Criterion not met.ᶜ Concentrations varied widely. |
| **Upgrade: Residual confounding** | Is it plausible that all important residual confounding would reduce the observed effect, or create an effect where none is observed? | Not applicableᶜ | Criterion not met.ᶜ No matching, few controls. |
| **Overall certainty of evidence** | High: very confident that the true effect lies close to the effect estimate.  Moderate: moderately confident that the true effect lies close to the effect estimate.  Low: confidence in the effect estimate is limited.  Very low: very little confidence in the effect estimate. | **⊕○○○**  **Very low** | Starting from very low certainty on the basis of study design features, the body of evidence was downgraded for the following domains: Inconsistency (serious); Indirectness (very serious); Imprecision (serious); Publication bias (serious). No serious concerns were identified in the remaining domains. Upgrading criteria were not applied because serious concerns were identified in one or more downgrading domains. The overall certainty of evidence for lung disease is therefore very low: very little confidence in the effect estimates, and the true effect is likely to be substantially different from the estimate. |

*Note: AF = aflatoxin; RoB = Risk of bias.*

*ᵃ Initial certainty was assigned on the basis of study design features rather than a uniform starting point for all observational evidence, following the adaptation of GRADE for observational exposure data by Morgan et al. (2016). Bodies of evidence meeting all four features (investigator-controlled exposure; exposure demonstrably preceding the outcome; outcome data at the individual level; presence of a comparison group) begin at high certainty, with one level deducted for each feature not met. Because exposure was not investigator-controlled in any included study, the maximum attainable initial certainty in this review was moderate.*

*ᵇ To avoid double counting, the appropriateness of the study design relative to the time course of the outcome is captured in the initial certainty rating; it is therefore reported descriptively under indirectness but does not contribute an additional downgrade.*

*ᶜ In accordance with GRADE guidance, upgrading criteria are considered only where no serious concerns are identified in any downgrading domain. The observations recorded here are retained for transparency but were not applied.*

# **Table 40 Detailed overview, including the criteria & rationale for decisions of the GRADE assessment of the overall body of evidence of this SR for immunomodulation.**

|  | **CRITERIA** | **Judgement** | **Rationale** |
| --- | --- | --- | --- |
| **Initial certainty based on study design** | Assigned from study design features (Morgan et al., 2016):  1. investigator-controlled exposure?  2. exposure demonstrably prior to outcome?  3. outcome data at individual level?  4. presence of a comparison group? | Lowᵃ | 1. Not met – exposure was environmental/dietary and not investigator-controlled.  2. Met – most studies applied a design appropriate to the time course of the outcome.  3. Met – exposure and outcome data were reported at the individual level.  4. Not met – most studies reported no comparison group.  2 of 4 features met → initial certainty low. |
| **Downgrade: RoB** | 1. Is the RoB considered serious? | Not serious | X No, most studies had low RoB (see Table 28) |
| **Downgrade: Inconsistency** | Are there different subgroups of patients with different effects? | Serious | √ Yes, CCs varies widely from positive to negative. |
| **Downgrade: Indirectness** | Are there differences that could seriously impact the magnitude of effect in:  1. populations?  2. exposure?  3. outcome?  4. comparator?  5. study design? | Not serious | X No, all African countries, children and adults.  X No, OTA & AFs in blood.  X No, diagnosed in hospital setting.  Mostly without controls.  X No, mostly appropriate study designs.  Note: item 5 (study design) is reflected in the initial certainty rating and does not contribute an additional downgrade here.ᵇ |
| **Downgrade: Imprecision** | Is there an inadequate sample size (suboptimal information size, no sample size calculation, sample sizes < 100) ?  Are the reported 95% confidence interval broad? | Not serious | X No, most studies sample size >100.  X No, reported 95% confidence intervals were small. |
| **Downgrade: Publication bias** | Are results prone to bias due to file-drawering effect? | Not serious | X No, non-significant effects mentioned. |
| **Upgrade: Large magnitude of effect** | Upgrade with 1 level if risk reduction ± 50% or OR ≥ 2?  Upgrade with 2 levels if risk reduction ± 80% or OR between 5 and 10? | Not applicableᶜ | Criterion met, but not applied.ᶜ Observation recorded: OR of 2.8 for T cell activation. |
| **Upgrade: presence of a clear dose–response gradient** | Presence of a clear dose-response gradient? | Not applicableᶜ | Criterion not met.ᶜ Concentrations varied widely. |
| **Upgrade: Residual confounding** | Is it plausible that all important residual confounding would reduce the observed effect, or create an effect where none is observed? | Not applicableᶜ | Criterion not met.ᶜ No matching, few controls. |
| **Overall certainty of evidence** | High: very confident that the true effect lies close to the effect estimate.  Moderate: moderately confident that the true effect lies close to the effect estimate.  Low: confidence in the effect estimate is limited.  Very low: very little confidence in the effect estimate. | **⊕○○○**  **Very low** | Starting from low certainty on the basis of study design features, the body of evidence was downgraded for the following domains: Inconsistency (serious). No serious concerns were identified in the remaining domains. Upgrading criteria were not applied because serious concerns were identified in one or more downgrading domains. The overall certainty of evidence for immunomodulation is therefore very low: very little confidence in the effect estimates, and the true effect is likely to be substantially different from the estimate. |

*Note: AF = aflatoxin; OTA = ochratoxin A; OR = odds ratio; RoB = Risk of bias; ELISA = Enzyme-Linked Immunosorbent Assay; RIA = radioimmunoassay; OR = odds ratio; CC = correlation coefficient.*

*ᵃ Initial certainty was assigned on the basis of study design features rather than a uniform starting point for all observational evidence, following the adaptation of GRADE for observational exposure data by Morgan et al. (2016). Bodies of evidence meeting all four features (investigator-controlled exposure; exposure demonstrably preceding the outcome; outcome data at the individual level; presence of a comparison group) begin at high certainty, with one level deducted for each feature not met. Because exposure was not investigator-controlled in any included study, the maximum attainable initial certainty in this review was moderate.*

*ᵇ To avoid double counting, the appropriateness of the study design relative to the time course of the outcome is captured in the initial certainty rating; it is therefore reported descriptively under indirectness but does not contribute an additional downgrade.*

*ᶜ In accordance with GRADE guidance, upgrading criteria are considered only where no serious concerns are identified in any downgrading domain. The observations recorded here are retained for transparency but were not applied.*

# **Table 41 Detailed overview, including the criteria & rationale for decisions of the GRADE assessment of the overall body of evidence of this SR for kwashiorkor.**

|  | **CRITERIA** | **Judgement** | **Rationale** |
| --- | --- | --- | --- |
| **Initial certainty based on study design** | Assigned from study design features (Morgan et al., 2016):  1. investigator-controlled exposure?  2. exposure demonstrably prior to outcome?  3. outcome data at individual level?  4. presence of a comparison group? | Lowᵃ | 1. Not met – exposure was environmental/dietary and not investigator-controlled.  2. Not met – most studies applied a design that does not establish temporality.  3. Met – exposure and outcome data were reported at the individual level.  4. Met – most studies included controls matched for age, sex and/or origin.  2 of 4 features met → initial certainty low. |
| **Downgrade: RoB** | 1. Is the RoB considered serious? | Not serious | X No, most studies had low RoB (see Table 28) |
| **Downgrade: Inconsistency** | Are there different subgroups of patients with different effects? | Serious | √ Yes, prevalence varied widely. |
| **Downgrade: Indirectness** | Are there differences that could seriously impact the magnitude of effect in:  1. populations?  2. exposure?  3. outcome?  4. comparator?  5. study design? | Not serious | X No, all low-income countries, children.  X No, all AFs, always in urine, tissue and blood.  X No, all diagnosed in hospital setting.  X No, mostly controls matched for age, sex and/or origin.  √ Yes, mostly inappropriate study designs.  Note: item 5 (study design) is reflected in the initial certainty rating and does not contribute an additional downgrade here.ᵇ |
| **Downgrade: Imprecision** | Is there an inadequate sample size (suboptimal information size, no sample size calculation, sample sizes < 100) ?  Are the reported 95% confidence interval broad? | Serious | √ Yes, most studies sample size <100.  X No, none reported. |
| **Downgrade: Publication bias** | Are results prone to bias due to file-drawering effect? | Serious | √ Yes, hospital admission biases, favoring individuals with the financial means, typical in outbreak settings in low-income countries. |
| **Upgrade: Large magnitude of effect** | Upgrade with 1 level if risk reduction ± 50% or OR ≥ 2?  Upgrade with 2 levels if risk reduction ± 80% or OR between 5 and 10? | Not applicableᶜ | Criterion not met.ᶜ No effect size and varying prevalences. |
| **Upgrade: presence of a clear dose–response gradient** | Presence of a clear dose-response gradient? | Not applicableᶜ | Criterion not met.ᶜ Concentrations varied widely. |
| **Upgrade: Residual confounding** | Is it plausible that all important residual confounding would reduce the observed effect, or create an effect where none is observed? | Not applicableᶜ | Criterion not met.ᶜ No matching, few controls. |
| **Overall certainty of evidence** | High: very confident that the true effect lies close to the effect estimate.  Moderate: moderately confident that the true effect lies close to the effect estimate.  Low: confidence in the effect estimate is limited.  Very low: very little confidence in the effect estimate. | **⊕○○○**  **Very low** | Starting from low certainty on the basis of study design features, the body of evidence was downgraded for the following domains: Inconsistency (serious); Imprecision (serious); Publication bias (serious). No serious concerns were identified in the remaining domains. Upgrading criteria were not applied because serious concerns were identified in one or more downgrading domains. The overall certainty of evidence for kwashiorkor is therefore very low: very little confidence in the effect estimates, and the true effect is likely to be substantially different from the estimate. |

*Note: AF = aflatoxin; OR = odds ratio; RoB = Risk of bias; HPLC-FLD = High-Performance Liquid Chromatography with Fluorescence Detection.*

*ᵃ Initial certainty was assigned on the basis of study design features rather than a uniform starting point for all observational evidence, following the adaptation of GRADE for observational exposure data by Morgan et al. (2016). Bodies of evidence meeting all four features (investigator-controlled exposure; exposure demonstrably preceding the outcome; outcome data at the individual level; presence of a comparison group) begin at high certainty, with one level deducted for each feature not met. Because exposure was not investigator-controlled in any included study, the maximum attainable initial certainty in this review was moderate.*

*ᵇ To avoid double counting, the appropriateness of the study design relative to the time course of the outcome is captured in the initial certainty rating; it is therefore reported descriptively under indirectness but does not contribute an additional downgrade.*

*ᶜ In accordance with GRADE guidance, upgrading criteria are considered only where no serious concerns are identified in any downgrading domain. The observations recorded here are retained for transparency but were not applied.*

# **Table 42 Detailed overview, including the criteria & rationale for decisions of the GRADE assessment of the overall body of evidence of this SR for wasting.**

|  | **CRITERIA** | **Judgement** | **Rationale** |
| --- | --- | --- | --- |
| **Initial certainty based on study design** | Assigned from study design features (Morgan et al., 2016):  1. investigator-controlled exposure?  2. exposure demonstrably prior to outcome?  3. outcome data at individual level?  4. presence of a comparison group? | Very lowᵃ | 1. Not met – exposure was environmental/dietary and not investigator-controlled.  2. Not met – most studies applied a design that does not establish temporality.  3. Met – exposure and outcome data were reported at the individual level.  4. Not met – most studies reported no comparison group.  1 of 4 features met → initial certainty very low. |
| **Downgrade: RoB** | 1. Is the RoB considered serious? | Not serious | X No, most studies had low RoB (see Table 28) |
| **Downgrade: Inconsistency** | 1. Are there different subgroups of patients with different effects? | Serious | √ Yes, ORs varied. |
| **Downgrade: Indirectness** | Are there differences that could seriously impact the magnitude of effect in:  1. populations?  2. exposure?  3. outcome?  4. comparator?  5. study design? | Very serious | √ Yes, all low-income countries, however different ages.  √ Yes, AFs & multiple mycotoxins, in blood or milk.  √ Yes, often not in hospital setting.  Mostly without controls.  √ Yes, mostly inappropriate study designs.  Note: item 5 (study design) is reflected in the initial certainty rating and does not contribute an additional downgrade here.ᵇ |
| **Downgrade: Imprecision** | Is there an inadequate sample size (suboptimal information size, no sample size calculation, sample sizes < 100) ?  Are the reported 95% confidence interval broad? | Not serious | X No, most studies sample size >100.  X No, reported 95% confidence intervals were small. |
| **Downgrade: Publication bias** | Are results prone to bias due to file-drawering effect? | Not serious | X No, reports of non-significance. |
| **Upgrade: Large magnitude of effect** | Upgrade with 1 level if risk reduction ± 50% or OR ≥ 2?  Upgrade with 2 levels if risk reduction ± 80% or OR between 5 and 10? | Not applicableᶜ | Criterion not met.ᶜ ORs <1. |
| **Upgrade: presence of a clear dose–response gradient** | Presence of a clear dose-response gradient? | Not applicableᶜ | Criterion not met.ᶜ Concentrations varied widely. |
| **Upgrade: Residual confounding** | Is it plausible that all important residual confounding would reduce the observed effect, or create an effect where none is observed? | Not applicableᶜ | Criterion not met.ᶜ No matching, almost no controls. |
| **Overall certainty of evidence** | High: very confident that the true effect lies close to the effect estimate.  Moderate: moderately confident that the true effect lies close to the effect estimate.  Low: confidence in the effect estimate is limited.  Very low: very little confidence in the effect estimate. | **⊕○○○**  **Very low** | Starting from very low certainty on the basis of study design features, the body of evidence was downgraded for the following domains: Inconsistency (serious); Indirectness (very serious). No serious concerns were identified in the remaining domains. Upgrading criteria were not applied because serious concerns were identified in one or more downgrading domains. The overall certainty of evidence for wasting is therefore very low: very little confidence in the effect estimates, and the true effect is likely to be substantially different from the estimate. |

*Note: AF = aflatoxin; RoB = Risk of bias; OR = odds ratio; LC-MS/MS = liquid chromatography tandem mass spectrometry; ELISA = Enzyme-Linked Immunosorbent Assay; HPLC-FLD = High-Performance Liquid Chromatography with Fluorescence Detection; IDMS = Isotope Dilution Mass Spectrometry.*

*ᵃ Initial certainty was assigned on the basis of study design features rather than a uniform starting point for all observational evidence, following the adaptation of GRADE for observational exposure data by Morgan et al. (2016). Bodies of evidence meeting all four features (investigator-controlled exposure; exposure demonstrably preceding the outcome; outcome data at the individual level; presence of a comparison group) begin at high certainty, with one level deducted for each feature not met. Because exposure was not investigator-controlled in any included study, the maximum attainable initial certainty in this review was moderate.*

*ᵇ To avoid double counting, the appropriateness of the study design relative to the time course of the outcome is captured in the initial certainty rating; it is therefore reported descriptively under indirectness but does not contribute an additional downgrade.*

*ᶜ In accordance with GRADE guidance, upgrading criteria are considered only where no serious concerns are identified in any downgrading domain. The observations recorded here are retained for transparency but were not applied.*

# **Table 43 Detailed overview, including the criteria & rationale for decisions of the GRADE assessment of the overall body of evidence of this SR for underweight.**

|  | **CRITERIA** | **Judgement** | **Rationale** |
| --- | --- | --- | --- |
| **Initial certainty based on study design** | Assigned from study design features (Morgan et al., 2016):  1. investigator-controlled exposure?  2. exposure demonstrably prior to outcome?  3. outcome data at individual level?  4. presence of a comparison group? | Very lowᵃ | 1. Not met – exposure was environmental/dietary and not investigator-controlled.  2. Not met – most studies applied a design that does not establish temporality.  3. Met – exposure and outcome data were reported at the individual level.  4. Not met – most studies reported no comparison group.  1 of 4 features met → initial certainty very low. |
| **Downgrade: RoB** | 1. Is the RoB considered serious? | Not serious | X No, most studies had low RoB (see Table 28) |
| **Downgrade: Inconsistency** | Are there different subgroups of patients with different effects? | Serious | √ Yes, although all ORs are around 1, there is high statistical heterogeneity (I2 = 82.5%). |
| **Downgrade: Indirectness** | Are there differences that could seriously impact the magnitude of effect in:  1. populations?  2. exposure?  3. outcome?  4. comparator?  5. study design? | Very serious | √ Yes, mostly low-income countries, however different ages.  √ Yes, different mycotoxins, different matrices.  √ Yes, often not in hospital setting.  Mostly without controls.  √ Yes, mostly inappropriate study designs.  Note: item 5 (study design) is reflected in the initial certainty rating and does not contribute an additional downgrade here.ᵇ |
| **Downgrade: Imprecision** | Is there an inadequate sample size (suboptimal information size, no sample size calculation, sample sizes < 100) ?  Are the reported 95% confidence interval broad? | Not serious | X No, most studies sample size >100.  X No, if reported it was small. |
| **Downgrade: Publication bias** | Are results prone to bias due to file-drawering effect? | Not serious | X No, reports of non-significance. |
| **Upgrade: Large magnitude of effect** | Upgrade with 1 level if risk reduction ± 50% or OR ≥ 2?  Upgrade with 2 levels if risk reduction ± 80% or OR between 5 and 10? | Not applicableᶜ | Criterion not met.ᶜ ORs ≤ 1. |
| **Upgrade: presence of a clear dose–response gradient** | Presence of a clear dose-response gradient? | Not applicableᶜ | Criterion not met.ᶜ Concentrations varied widely. |
| **Upgrade: Residual confounding** | Is it plausible that all important residual confounding would reduce the observed effect, or create an effect where none is observed? | Not applicableᶜ | Criterion not met.ᶜ Mainly no matching and almost no controls. |
| **Overall certainty of evidence** | High: very confident that the true effect lies close to the effect estimate.  Moderate: moderately confident that the true effect lies close to the effect estimate.  Low: confidence in the effect estimate is limited.  Very low: very little confidence in the effect estimate. | **⊕○○○**  **Very low** | Starting from very low certainty on the basis of study design features, the body of evidence was downgraded for the following domains: Inconsistency (serious); Indirectness (very serious). No serious concerns were identified in the remaining domains. Upgrading criteria were not applied because serious concerns were identified in one or more downgrading domains. The overall certainty of evidence for underweight is therefore very low: very little confidence in the effect estimates, and the true effect is likely to be substantially different from the estimate. |

*Note: RoB = Risk of bias; OR = odds ratio; LC-MS/MS = liquid chromatography tandem mass spectrometry; ELISA = Enzyme-Linked Immunosorbent Assay; HPLC-FLD = High-Performance Liquid Chromatography with Fluorescence Detection; IDMS = Isotope Dilution Mass Spectrometry.*

*ᵃ Initial certainty was assigned on the basis of study design features rather than a uniform starting point for all observational evidence, following the adaptation of GRADE for observational exposure data by Morgan et al. (2016). Bodies of evidence meeting all four features (investigator-controlled exposure; exposure demonstrably preceding the outcome; outcome data at the individual level; presence of a comparison group) begin at high certainty, with one level deducted for each feature not met. Because exposure was not investigator-controlled in any included study, the maximum attainable initial certainty in this review was moderate.*

*ᵇ To avoid double counting, the appropriateness of the study design relative to the time course of the outcome is captured in the initial certainty rating; it is therefore reported descriptively under indirectness but does not contribute an additional downgrade.*

*ᶜ In accordance with GRADE guidance, upgrading criteria are considered only where no serious concerns are identified in any downgrading domain. The observations recorded here are retained for transparency but were not applied.*

# **Table 44 Detailed overview, including the criteria & rationale for decisions of the GRADE assessment of the overall body of evidence of this SR for stunting.**

|  | **CRITERIA** | **Judgement** | **Rationale** |
| --- | --- | --- | --- |
| **Initial certainty based on study design** | Assigned from study design features (Morgan et al., 2016):  1. investigator-controlled exposure?  2. exposure demonstrably prior to outcome?  3. outcome data at individual level?  4. presence of a comparison group? | Very lowᵃ | 1. Not met – exposure was environmental/dietary and not investigator-controlled.  2. Not met – most studies applied a design that does not establish temporality.  3. Met – exposure and outcome data were reported at the individual level.  4. Not met – most studies reported no comparison group.  1 of 4 features met → initial certainty very low. |
| **Downgrade: RoB** | 1. Is the RoB considered serious? | Not serious | X No, most studies had low RoB (see Table 28) |
| **Downgrade: Inconsistency** | Are there different subgroups of patients with different effects? | Serious | √ Yes, although ORs mostly around 1, there is substantial statistical heterogeneity (I2 = 65.1%). |
| **Downgrade: Indirectness** | Are there differences that could seriously impact the magnitude of effect in:  1. populations?  2. exposure?  3. outcome?  4. comparator?  5. study design? | Very serious | X No, mostly low-income countries, mostly children.  √ Yes, different mycotoxins, different matrices.  √ Yes, often not in hospital setting.  Mostly without controls.  √ Yes, mostly inappropriate study designs.  Note: item 5 (study design) is reflected in the initial certainty rating and does not contribute an additional downgrade here.ᵇ |
| **Downgrade: Imprecision** | Is there an inadequate sample size (suboptimal information size, no sample size calculation, sample sizes < 100) ?  Are the reported 95% confidence interval broad? | Not serious | X No, most studies sample size >100.  X No, most confidence intervals were small. |
| **Downgrade: Publication bias** | Are results prone to bias due to file-drawering effect? | Not serious | X No, reports of non-significance. |
| **Upgrade: Large magnitude of effect** | Upgrade with 1 level if risk reduction ± 50% or OR ≥ 2?  Upgrade with 2 levels if risk reduction ± 80% or OR between 5 and 10? | Not applicableᶜ | Criterion not met.ᶜ ORs mostly around 1. |
| **Upgrade: presence of a clear dose–response gradient** | Presence of a clear dose-response gradient? | Not applicableᶜ | Criterion not met.ᶜ Concentrations varied widely. |
| **Upgrade: Residual confounding** | Is it plausible that all important residual confounding would reduce the observed effect, or create an effect where none is observed? | Not applicableᶜ | Criterion not met.ᶜ Mainly no matching and almost no controls. |
| **Overall certainty of evidence** | High: very confident that the true effect lies close to the effect estimate.  Moderate: moderately confident that the true effect lies close to the effect estimate.  Low: confidence in the effect estimate is limited.  Very low: very little confidence in the effect estimate. | **⊕○○○**  **Very low** | Starting from very low certainty on the basis of study design features, the body of evidence was downgraded for the following domains: Inconsistency (serious); Indirectness (very serious). No serious concerns were identified in the remaining domains. Upgrading criteria were not applied because serious concerns were identified in one or more downgrading domains. The overall certainty of evidence for stunting is therefore very low: very little confidence in the effect estimates, and the true effect is likely to be substantially different from the estimate. |

*Note: RoB = Risk of bias; OR = odds ratio; LC-MS/MS = liquid chromatography tandem mass spectrometry; ELISA = Enzyme-Linked Immunosorbent Assay; HPLC-FLD = High-Performance Liquid Chromatography with Fluorescence Detection; IDMS = Isotope Dilution Mass Spectrometry.*

*ᵃ Initial certainty was assigned on the basis of study design features rather than a uniform starting point for all observational evidence, following the adaptation of GRADE for observational exposure data by Morgan et al. (2016). Bodies of evidence meeting all four features (investigator-controlled exposure; exposure demonstrably preceding the outcome; outcome data at the individual level; presence of a comparison group) begin at high certainty, with one level deducted for each feature not met. Because exposure was not investigator-controlled in any included study, the maximum attainable initial certainty in this review was moderate.*

*ᵇ To avoid double counting, the appropriateness of the study design relative to the time course of the outcome is captured in the initial certainty rating; it is therefore reported descriptively under indirectness but does not contribute an additional downgrade.*

*ᶜ In accordance with GRADE guidance, upgrading criteria are considered only where no serious concerns are identified in any downgrading domain. The observations recorded here are retained for transparency but were not applied.*

# **Table 45 Detailed overview, including the criteria & rationale for decisions of the GRADE assessment of the overall body of evidence of this SR for blood disease.**

|  | **CRITERIA** | **Judgement** | **Rationale** |
| --- | --- | --- | --- |
| **Initial certainty based on study design** | Assigned from study design features (Morgan et al., 2016):  1. investigator-controlled exposure?  2. exposure demonstrably prior to outcome?  3. outcome data at individual level?  4. presence of a comparison group? | Very lowᵃ | 1. Not met – exposure was environmental/dietary and not investigator-controlled.  2. Not met – most studies applied a design that does not establish temporality.  3. Met – exposure and outcome data were reported at the individual level.  4. Not met – most studies reported no comparison group.  1 of 4 features met → initial certainty very low. |
| **Downgrade: RoB** | 1. Is the RoB considered serious? | Not serious | X No, most studies had low RoB (see Table 28) |
| **Downgrade: Inconsistency** | Are there different subgroups of patients with different effects? | Serious | √ Yes, ORs vary widely. |
| **Downgrade: Indirectness** | Are there differences that could seriously impact the magnitude of effect in:  1. populations?  2. exposure?  3. outcome?  4. comparator?  5. study design? | Very serious | √ Yes, low- and high-income countries, children & adults.  √ Yes, different mycotoxins, different matrices.  √ Yes, often not in hospital setting.  Mostly without controls.  √ Yes, mostly inappropriate study designs.  Note: item 5 (study design) is reflected in the initial certainty rating and does not contribute an additional downgrade here.ᵇ |
| **Downgrade: Imprecision** | Is there an inadequate sample size (suboptimal information size, no sample size calculation, sample sizes < 100) ?  Are the reported 95% confidence interval broad? | Serious | X No, most studies sample size >100.  √ Yes, with broadest OR = 13.72 (0.92 – 203.55) and OR = 3.08 (3.08 – 67.02) |
| **Downgrade: Publication bias** | Are results prone to bias due to file-drawering effect? | Not serious | X No, reports of non-significance. |
| **Upgrade: Large magnitude of effect** | Upgrade with 1 level if risk reduction ± 50% or OR ≥ 2?  Upgrade with 2 levels if risk reduction ± 80% or OR between 5 and 10? | Not applicableᶜ | Criterion met, but not applied.ᶜ Observation recorded: ORs between 1 and 112. |
| **Upgrade: presence of a clear dose–response gradient** | Presence of a clear dose-response gradient? | Not applicableᶜ | Criterion not met.ᶜ Concentrations varied widely. |
| **Upgrade: Residual confounding** | Is it plausible that all important residual confounding would reduce the observed effect, or create an effect where none is observed? | Not applicableᶜ | Criterion not met.ᶜ Mainly no matching and almost no controls. |
| **Overall certainty of evidence** | High: very confident that the true effect lies close to the effect estimate.  Moderate: moderately confident that the true effect lies close to the effect estimate.  Low: confidence in the effect estimate is limited.  Very low: very little confidence in the effect estimate. | **⊕○○○**  **Very low** | Starting from very low certainty on the basis of study design features, the body of evidence was downgraded for the following domains: Inconsistency (serious); Indirectness (very serious); Imprecision (serious). No serious concerns were identified in the remaining domains. Upgrading criteria were not applied because serious concerns were identified in one or more downgrading domains. The overall certainty of evidence for blood disease is therefore very low: very little confidence in the effect estimates, and the true effect is likely to be substantially different from the estimate. |

*Note: RoB = Risk of bias; OR = odds ratio; ELISA = Enzyme-Linked Immunosorbent Assay; HPLC-FLD = High-Performance Liquid Chromatography with Fluorescence Detection; IDMS = Isotope Dilution Mass Spectrometry.*

*ᵃ Initial certainty was assigned on the basis of study design features rather than a uniform starting point for all observational evidence, following the adaptation of GRADE for observational exposure data by Morgan et al. (2016). Bodies of evidence meeting all four features (investigator-controlled exposure; exposure demonstrably preceding the outcome; outcome data at the individual level; presence of a comparison group) begin at high certainty, with one level deducted for each feature not met. Because exposure was not investigator-controlled in any included study, the maximum attainable initial certainty in this review was moderate.*

*ᵇ To avoid double counting, the appropriateness of the study design relative to the time course of the outcome is captured in the initial certainty rating; it is therefore reported descriptively under indirectness but does not contribute an additional downgrade.*

*ᶜ In accordance with GRADE guidance, upgrading criteria are considered only where no serious concerns are identified in any downgrading domain. The observations recorded here are retained for transparency but were not applied.*

# **Table 46 Detailed overview, including the criteria & rationale for decisions of the GRADE assessment of the overall body of evidence of this SR for oesophageal disease.**

|  | **CRITERIA** | **Judgement** | **Rationale** |
| --- | --- | --- | --- |
| **Initial certainty based on study design** | Assigned from study design features (Morgan et al., 2016):  1. investigator-controlled exposure?  2. exposure demonstrably prior to outcome?  3. outcome data at individual level?  4. presence of a comparison group? | Lowᵃ | 1. Not met – exposure was environmental/dietary and not investigator-controlled.  2. Not met – most studies applied a design that does not establish temporality.  3. Met – exposure and outcome data were reported at the individual level.  4. Met – all studies included well-matched controls.  2 of 4 features met → initial certainty low. |
| **Downgrade: RoB** | 1. Is the RoB considered serious? | Not serious | X No, most studies had low RoB (see Table 28) |
| **Downgrade: Inconsistency** | Are there different subgroups of patients with different effects? | Not serious | X No, ORs similar. |
| **Downgrade: Indirectness** | Are there differences that could seriously impact the magnitude of effect in:  1. populations?  2. exposure?  3. outcome?  4. comparator?  5. study design? | Very serious | √ Yes, low- and high-income countries, children & adults.  √ Yes, different mycotoxins, different matrices.  X No, diagnosed in hospital setting.  X No, all with well-matched controls.  √ Yes, mostly inappropriate study designs.  Note: item 5 (study design) is reflected in the initial certainty rating and does not contribute an additional downgrade here.ᵇ |
| **Downgrade: Imprecision** | Is there an inadequate sample size (suboptimal information size, no sample size calculation, sample sizes < 100) ?  Are the reported 95% confidence interval broad? | Not serious | X No, most studies sample size >100.  X No, if reported the 95% confidence intervals were small. |
| **Downgrade: Publication bias** | Are results prone to bias due to file-drawering effect? | Not serious | X No, reports of non-significance. |
| **Upgrade: Large magnitude of effect** | Upgrade with 1 level if risk reduction ± 50% or OR ≥ 2?  Upgrade with 2 levels if risk reduction ± 80% or OR between 5 and 10? | Not applicableᶜ | Criterion met, but not applied.ᶜ Observation recorded: ORs between 1 and 7. |
| **Upgrade: presence of a clear dose–response gradient** | Presence of a clear dose-response gradient? | Not applicableᶜ | Criterion not met.ᶜ Concentrations varied widely. |
| **Upgrade: Residual confounding** | Is it plausible that all important residual confounding would reduce the observed effect, or create an effect where none is observed? | Not applicableᶜ | Criterion met, but not applied.ᶜ Observation recorded: All controlled and often matched. |
| **Overall certainty of evidence** | High: very confident that the true effect lies close to the effect estimate.  Moderate: moderately confident that the true effect lies close to the effect estimate.  Low: confidence in the effect estimate is limited.  Very low: very little confidence in the effect estimate. | **⊕○○○**  **Very low** | Starting from low certainty on the basis of study design features, the body of evidence was downgraded for the following domains: Indirectness (very serious). No serious concerns were identified in the remaining domains. Upgrading criteria were not applied because serious concerns were identified in one or more downgrading domains. The overall certainty of evidence for oesophageal disease is therefore very low: very little confidence in the effect estimates, and the true effect is likely to be substantially different from the estimate. |

*Note: RoB = Risk of bias; OR = odds ratio.*

*ᵃ Initial certainty was assigned on the basis of study design features rather than a uniform starting point for all observational evidence, following the adaptation of GRADE for observational exposure data by Morgan et al. (2016). Bodies of evidence meeting all four features (investigator-controlled exposure; exposure demonstrably preceding the outcome; outcome data at the individual level; presence of a comparison group) begin at high certainty, with one level deducted for each feature not met. Because exposure was not investigator-controlled in any included study, the maximum attainable initial certainty in this review was moderate.*

*ᵇ To avoid double counting, the appropriateness of the study design relative to the time course of the outcome is captured in the initial certainty rating; it is therefore reported descriptively under indirectness but does not contribute an additional downgrade.*

*ᶜ In accordance with GRADE guidance, upgrading criteria are considered only where no serious concerns are identified in any downgrading domain. The observations recorded here are retained for transparency but were not applied.*

# **Table 47 Detailed overview, including the criteria & rationale for decisions of the GRADE assessment of the overall body of evidence of this SR for gastric disease.**

|  | **CRITERIA** | **Judgement** | **Rationale** |
| --- | --- | --- | --- |
| **Initial certainty based on study design** | Assigned from study design features (Morgan et al., 2016):  1. investigator-controlled exposure?  2. exposure demonstrably prior to outcome?  3. outcome data at individual level?  4. presence of a comparison group? | Very lowᵃ | 1. Not met – exposure was environmental/dietary and not investigator-controlled.  2. Not met – most studies applied a design that does not establish temporality.  3. Met – exposure and outcome data were reported at the individual level.  4. Not met – most studies reported no comparison group.  1 of 4 features met → initial certainty very low. |
| **Downgrade: RoB** | 1. Is the RoB considered serious? | Not serious | X No, studies had more def lower RoB than higher RoB (see Table 28) |
| **Downgrade: Inconsistency** | Are there different subgroups of patients with different effects? | Serious | √ Prevalence varied widely. |
| **Downgrade: Indirectness** | Are there differences that could seriously impact the magnitude of effect in:  1. populations?  2. exposure?  3. outcome?  4. comparator?  5. study design? | Serious | √ Yes, low- and high-income countries, children & adults.  X No, mostly AFs in tissue.  X No, diagnosed in hospital setting.  Mostly no controls.  √ Yes, mostly inappropriate study designs.  Note: item 5 (study design) is reflected in the initial certainty rating and does not contribute an additional downgrade here.ᵇ |
| **Downgrade: Imprecision** | Is there an inadequate sample size (suboptimal information size, no sample size calculation, sample sizes < 100) ?  Are the reported 95% confidence interval broad? | Serious | √ Yes, 2 out of 4 studies with sample size <100.  X No, if reported the 95% confidence intervals were small. |
| **Downgrade: Publication bias** | Are results prone to bias due to file-drawering effect? | Not serious | X No, reports of non-significance and case studies. |
| **Upgrade: Large magnitude of effect** | Upgrade with 1 level if risk reduction ± 50% or OR ≥ 2?  Upgrade with 2 levels if risk reduction ± 80% or OR between 5 and 10? | Not applicableᶜ | Criterion met, but not applied.ᶜ Observation recorded: ORs between 1.4 and 2.6. |
| **Upgrade: presence of a clear dose–response gradient** | Presence of a clear dose-response gradient? | Not applicableᶜ | Criterion not met.ᶜ Only one time was a low concentration reported in a case study. |
| **Upgrade: Residual confounding** | Is it plausible that all important residual confounding would reduce the observed effect, or create an effect where none is observed? | Not applicableᶜ | Criterion not met.ᶜ Mostly not controlled or matched. |
| **Overall certainty of evidence** | High: very confident that the true effect lies close to the effect estimate.  Moderate: moderately confident that the true effect lies close to the effect estimate.  Low: confidence in the effect estimate is limited.  Very low: very little confidence in the effect estimate. | **⊕○○○**  **Very low** | Starting from very low certainty on the basis of study design features, the body of evidence was downgraded for the following domains: Inconsistency (serious); Indirectness (serious); Imprecision (serious). No serious concerns were identified in the remaining domains. Upgrading criteria were not applied because serious concerns were identified in one or more downgrading domains. The overall certainty of evidence for gastric disease is therefore very low: very little confidence in the effect estimates, and the true effect is likely to be substantially different from the estimate. |

*Note: AF = aflatoxin; RoB = Risk of bias; OR = odds ratio.*

*ᵃ Initial certainty was assigned on the basis of study design features rather than a uniform starting point for all observational evidence, following the adaptation of GRADE for observational exposure data by Morgan et al. (2016). Bodies of evidence meeting all four features (investigator-controlled exposure; exposure demonstrably preceding the outcome; outcome data at the individual level; presence of a comparison group) begin at high certainty, with one level deducted for each feature not met. Because exposure was not investigator-controlled in any included study, the maximum attainable initial certainty in this review was moderate.*

*ᵇ To avoid double counting, the appropriateness of the study design relative to the time course of the outcome is captured in the initial certainty rating; it is therefore reported descriptively under indirectness but does not contribute an additional downgrade.*

*ᶜ In accordance with GRADE guidance, upgrading criteria are considered only where no serious concerns are identified in any downgrading domain. The observations recorded here are retained for transparency but were not applied.*

# **Table 48 Detailed overview, including the criteria & rationale for decisions of the GRADE assessment of the overall body of evidence of this SR for intestinal disease.**

|  | **CRITERIA** | **Judgement** | **Rationale** |
| --- | --- | --- | --- |
| **Initial certainty based on study design** | Assigned from study design features (Morgan et al., 2016):  1. investigator-controlled exposure?  2. exposure demonstrably prior to outcome?  3. outcome data at individual level?  4. presence of a comparison group? | Lowᵃ | 1. Not met – exposure was environmental/dietary and not investigator-controlled.  2. Not met – most studies applied a design that does not establish temporality.  3. Met – exposure and outcome data were reported at the individual level.  4. Met – most studies included controls matched for age, sex or origin.  2 of 4 features met → initial certainty low. |
| **Downgrade: RoB** | 1. Is the RoB considered serious? | Not serious | X No, most studies had low RoB (see Table 28) |
| **Downgrade: Inconsistency** | 1. Are there different subgroups of patients with different effects? | Serious | √ Prevalence varied widely. |
| **Downgrade: Indirectness** | Are there differences that could seriously impact the magnitude of effect in:  1. populations?  2. exposure?  3. outcome?  4. comparator?  5. study design? | Very serious | √ Yes, low- and high-income countries, children & adults.  √ Yes, different mycotoxins in different matrices.  X No, mostly diagnosed in hospital setting.  X No, most studies with controls, matched for either age, sex or origin.  √ Yes, mostly inappropriate study designs.  Note: item 5 (study design) is reflected in the initial certainty rating and does not contribute an additional downgrade here.ᵇ |
| **Downgrade: Imprecision** | Is there an inadequate sample size (suboptimal information size, no sample size calculation, sample sizes < 100) ?  Are the reported 95% confidence interval broad? | Serious | √ Yes, most with sample size <100.  X No, if reported, 95% confidence intervals were small. |
| **Downgrade: Publication bias** | Are results prone to bias due to file-drawering effect? | Not serious | X No, reports of non-significance and case studies. |
| **Upgrade: Large magnitude of effect** | Upgrade with 1 level if risk reduction ± 50% or OR ≥ 2?  Upgrade with 2 levels if risk reduction ± 80% or OR between 5 and 10? | Not applicableᶜ | Criterion not met.ᶜ Prevalence varied, no ORs calculated. |
| **Upgrade: presence of a clear dose–response gradient** | Presence of a clear dose-response gradient? | Not applicableᶜ | Criterion not met.ᶜ Concentrations varied widely. |
| **Upgrade: Residual confounding** | Is it plausible that all important residual confounding would reduce the observed effect, or create an effect where none is observed? | Not applicableᶜ | Criterion not met.ᶜ Mostly controlled and matched. |
| **Overall certainty of evidence** | High: very confident that the true effect lies close to the effect estimate.  Moderate: moderately confident that the true effect lies close to the effect estimate.  Low: confidence in the effect estimate is limited.  Very low: very little confidence in the effect estimate. | **⊕○○○**  **Very low** | Starting from low certainty on the basis of study design features, the body of evidence was downgraded for the following domains: Inconsistency (serious); Indirectness (very serious); Imprecision (serious). No serious concerns were identified in the remaining domains. Upgrading criteria were not applied because serious concerns were identified in one or more downgrading domains. The overall certainty of evidence for intestinal disease is therefore very low: very little confidence in the effect estimates, and the true effect is likely to be substantially different from the estimate. |

*Note: RoB = Risk of bias; OR = odds ratio.*

*ᵃ Initial certainty was assigned on the basis of study design features rather than a uniform starting point for all observational evidence, following the adaptation of GRADE for observational exposure data by Morgan et al. (2016). Bodies of evidence meeting all four features (investigator-controlled exposure; exposure demonstrably preceding the outcome; outcome data at the individual level; presence of a comparison group) begin at high certainty, with one level deducted for each feature not met. Because exposure was not investigator-controlled in any included study, the maximum attainable initial certainty in this review was moderate.*

*ᵇ To avoid double counting, the appropriateness of the study design relative to the time course of the outcome is captured in the initial certainty rating; it is therefore reported descriptively under indirectness but does not contribute an additional downgrade.*

*ᶜ In accordance with GRADE guidance, upgrading criteria are considered only where no serious concerns are identified in any downgrading domain. The observations recorded here are retained for transparency but were not applied.*

# **Table 49 Detailed overview, including the criteria & rationale for decisions of the GRADE assessment of the overall body of evidence of this SR for adverse birth outcomes.**

|  | **CRITERIA** | **Judgement** | **Rationale** |
| --- | --- | --- | --- |
| **Initial certainty based on study design** | Assigned from study design features (Morgan et al., 2016):  1. investigator-controlled exposure?  2. exposure demonstrably prior to outcome?  3. outcome data at individual level?  4. presence of a comparison group? | Very lowᵃ | 1. Not met – exposure was environmental/dietary and not investigator-controlled.  2. Not met – most studies applied a design that does not establish temporality.  3. Met – exposure and outcome data were reported at the individual level.  4. Not met – most studies reported no comparison group.  1 of 4 features met → initial certainty very low. |
| **Downgrade: RoB** | 1. Is the RoB considered serious? | Not serious | X No, most studies had low RoB (see Table 28) |
| **Downgrade: Inconsistency** | 1. Are there different subgroups of patients with different effects? | Serious | √ ORs varied widely and there is substantial statistical heterogeneity for pregnancy aneamia (I2 = 72.8%). |
| **Downgrade: Indirectness** | Are there differences that could seriously impact the magnitude of effect in:  1. populations?  2. exposure?  3. outcome?  4. comparator?  5. study design? | Very serious | √ Yes, low- and high-income countries, children & adults.  √ Yes, different mycotoxins in different matrices.  √ Yes, often not diagnosed in hospital setting.  Most studies without controls.  √ Yes, mostly inappropriate study designs.  Note: item 5 (study design) is reflected in the initial certainty rating and does not contribute an additional downgrade here.ᵇ |
| **Downgrade: Imprecision** | Is there an inadequate sample size (suboptimal information size, no sample size calculation, sample sizes < 100) ?  Are the reported 95% confidence interval broad? | Not serious | X No, most with sample size >100.  X No, if reported the 95% confidence intervals were small. |
| **Downgrade: Publication bias** | Are results prone to bias due to file-drawering effect? | Not serious | X No, reports of non-significance. |
| **Upgrade: Large magnitude of effect** | Upgrade with 1 level if risk reduction ± 50% or OR ≥ 2?  Upgrade with 2 levels if risk reduction ± 80% or OR between 5 and 10? | Not applicableᶜ | Criterion not met.ᶜ ORs mostly <2, and for higher values the confidence intervals contain 1. |
| **Upgrade: presence of a clear dose–response gradient** | Presence of a clear dose-response gradient? | Not applicableᶜ | Criterion not met.ᶜ Concentrations varied widely. |
| **Upgrade: Residual confounding** | Is it plausible that all important residual confounding would reduce the observed effect, or create an effect where none is observed? | Not applicableᶜ | Criterion not met.ᶜ Mostly not controlled or matched. |
| **Overall certainty of evidence** | High: very confident that the true effect lies close to the effect estimate.  Moderate: moderately confident that the true effect lies close to the effect estimate.  Low: confidence in the effect estimate is limited.  Very low: very little confidence in the effect estimate. | **⊕○○○**  **Very low** | Starting from very low certainty on the basis of study design features, the body of evidence was downgraded for the following domains: Inconsistency (serious); Indirectness (very serious). No serious concerns were identified in the remaining domains. Upgrading criteria were not applied because serious concerns were identified in one or more downgrading domains. The overall certainty of evidence for adverse birth outcomes is therefore very low: very little confidence in the effect estimates, and the true effect is likely to be substantially different from the estimate. |

*Note: RoB = Risk of bias; OR = odds ratio.*

*ᵃ Initial certainty was assigned on the basis of study design features rather than a uniform starting point for all observational evidence, following the adaptation of GRADE for observational exposure data by Morgan et al. (2016). Bodies of evidence meeting all four features (investigator-controlled exposure; exposure demonstrably preceding the outcome; outcome data at the individual level; presence of a comparison group) begin at high certainty, with one level deducted for each feature not met. Because exposure was not investigator-controlled in any included study, the maximum attainable initial certainty in this review was moderate.*

*ᵇ To avoid double counting, the appropriateness of the study design relative to the time course of the outcome is captured in the initial certainty rating; it is therefore reported descriptively under indirectness but does not contribute an additional downgrade.*

*ᶜ In accordance with GRADE guidance, upgrading criteria are considered only where no serious concerns are identified in any downgrading domain. The observations recorded here are retained for transparency but were not applied.*

# **Table 50 Leave-one-out meta-analysis on all outcomes without metric transformation.**

| **Study omitted** | **RR** | **95% CI** | **Tau^2^** | **Tau** | **I^2^ (%)** | **95% PI** |
| --- | --- | --- | --- | --- | --- | --- |
| **HCC_urine_** | | | | | | |
| Ross, 1992 | 2.63 | [1.29 , 5.36] | 0.0 | 0.0 | 0.0% | [1.04 , 6.67] |
| Qian, 1994 | 2.50 | [1.56 ,4.01] | 0.0 | 0.0 | 0.0% | [1.02 , 6.16] |
| **Lu, 2010** | **3.26** | **[2.10 , 5.06]** | **0.0** | **0.0** | **0.0%** | **[0.95 , 11.20]** |
| Sun, 1999 | 2.56 | [1.35 , 4.83] | 0.0 | 0.0 | 0.0% | [1.03 , 6.36] |
| None | 2.66 | [1.80 , 3.93] | 0.0 | 0.0 | 0.0% | [1.43 , 4.95] |
| **Study omitted** | **OR** | **95% CI** | **Tau^2^** | **Tau** | **I^2^ (%)** | **95% PI** |
| **HCC_plasma_** | | | | | | |
| Chu, 2017 | 2.25 | [1.64; 3.09] | 0.1213 | 0.3483 | 46.0% | [1.02; 4.98] |
| **Kirk, 2005** | **2.20** | **[1.61; 3.01]** | **0.1076** | **0.3280** | **43.6%** | **[1.04; 4.67]** |
| Aydin, 2015 | 2.37 | [1.71; 3.30] | 0.1458 | 0.3818 | 48.1% | [1.00; 5.65] |
| Leonart, 2005 | 2.36 | [1.69; 3.29] | 0.1479 | 0.3846 | 48.5% | [0.99; 5.64] |
| Kirk, 2000 | 2.17 | [1.64; 2.87] | 0.0921 | 0.3035 | 36.7% | [1.08; 4.37] |
| Chen, 1996 | 2.24 | [1.63; 3.07] | 0.1193 | 0.3454 | 45.6% | [1.02; 4.93] |
| Rahman El-Zayadi, 2001 | 2.38 | [1.69; 3.34] | 0.1615 | 0.4019 | 48.6% | [0.96; 5.91] |
| Wu, 2009 | 2.44 | [1.75; 3.41] | 0.1435 | 0.3788 | 45.0% | [1.03; 5.79] |
| Zheng, 2017 | 2.39 | [1.70; 3.37] | 0.1631 | 0.4038 | 48.5% | [0.96; 5.98] |
| **Lu, 1998** | **2.25** | **[1.62; 3.13]** | **0.1292** | **0.3594** | **46.1%** | **[0.99; 5.11]** |
| Chen, 2007 | 2.35 | [1.67; 3.31] | 0.1613 | 0.4016 | 48.3% | [0.94; 5.84] |
| Wu, 2007 | 2.30 | [1.65; 3.20] | 0.1397 | 0.3737 | 47.9% | [0.98; 5.37] |
| **Mizrak, 2009** | **2.24** | **[1.62; 3.11]** | **0.1257** | **0.3545** | **46.0%** | **[1.00; 5.04]** |
| Sun, 2001 | 2.38 | [1.69; 3.35] | 0.1629 | 0.4036 | 48.6% | [0.95; 5.95] |
| Chu, 2018 | 2.25 | [1.63; 3.12] | 0.1276 | 0.3572 | 46.3% | [1.00; 5.09] |
| **Kimbi, 2005** | **2.22** | **[1.65; 2.99]** | **0.1100** | **0.3317** | **42.3%** | **[1.04; 4.74]** |
| Szymańska, 2009 | 2.37 | [1.81; 3.09] | 0.0304 | 0.1743 | 25.7% | [1.52; 3.69] |
| Srivatanakul, 1991 | 2.38 | [1.73; 3.28] | 0.1378 | 0.3713 | 46.7% | [1.03; 5.54] |
| None | 2.31 | [1.69; 3.15] | 0.1285 | 0.3585 | 45.4% | [1.03; 5.19] |
| **Liver cirrhosis** | | | | | | |
| Chu, 2017 | 4.48 | [0.90; 22.28] | 1.4055 | 1.1855 | 82.7% | [0.11; 179.48] |
| Alvarez, 2020 | 3.19 | [0.75; 13.57] | 1.1336 | 1.0647 | 82.1% | [0.12; 88.14] |
| **Kirk, 2005** | **2.77** | **[0.89; 8.57]** | **0.5573** | **0.7465** | **64.0%** | **[0.26; 29.79]** |
| Aydin, 2015 | 5.27 | [1.40; 19.86] | 0.9197 | 0.9590 | 81.8% | [0.26; 106.03] |
| Leonart, 2005 | 4.61 | [0.99; 21.46] | 1.2955 | 1.1382 | 84.9% | [0.13; 158.20] |
| Kirk, 2000 | 4.09 | [0.86; 19.50] | 1.3347 | 1.1553 | 85.6% | [0.12; 144.34] |
| None | 3.98 | [1.17; 13.55] | 1.1175 | 1.0571 | 82.0% | [0.20; 79.69] |
| **Underweight** | | | | | | |
| Andrews-Trevino, 2022 | 1.39 | [0.09, 21.49] | 1.06 | 1.03 | 88.1% | [0.01, 250.73] |
| Alamu, 2020 | 1.39 | [0.09, 21.07] | 1.05 | 1.03 | 88.3% | [0.01, 240.86] |
| **Ashraf, 2022** | **1.04** | **[0.41, 2.65]** | **0.05** | **0.23** | **56.8%** | **[0.31, 3.55]** |
| Shouman, 2012 | 1.74 | [0.27, 11.20] | 0.46 | 0.68 | 83.2% | [0.06, 53.01] |
| None | 1.33 | [0.33, 5.35] | 0.62 | 0.79 | 82.5% | [0.08, 22.74] |
| **Gallbladder cancer** | | | | | | |
| Koshiol, 2017 | 4.74 | [0.44, 51.43] | 0.69 | 0.83 | 74.1% | [0.06, 361.41] |
| Koshiol, 2024 | 5.04 | [0.69, 36.86] | 0.45 | 0.67 | 68.1% | [0.15, 174.76] |
| Yadav, 2025 | 3.68 | [0.35, 38.43] | 0.62 | 0.79 | 72.6% | [0.06, 209.01] |
| **Nogueira, 2015** | **2.64** | **[1.19, 5.88]** | **0.00** | **0.00** | **0%** | **[1.18, 5.91]** |
| None | 3.82 | [1.08, 13.53] | 0.39 | 0.62 | 63.5% | [0.37, 39.01] |
| **Pregnancy anaemia** | | | | | | |
| Shuaib, 2010 | 1.90 | [0.00, 10002.28] | 0.78 | 0.88 | 85.6% | [0.00, 2590359.76] |
| Lei, 2021 | 1.39 | [0.03, 64.59] | 0.12 | 0.34 | 64.0% | [0.00, 459.31] |
| Osoro, 2024 | 2.46 | [0.02, 245.70] | 0.16 | 0.39 | 56.0% | [0.00, 2224.83] |
| None | 1.83 | [0.36, 9.17] | 0.29 | 0.54 | 72.8% | [0.11, 30.31] |
| **Stunting** | | | | | | |
| **Andrews-Trevino, 2019** | **1.13** | **[0.99, 1.29]** | **0.01** | **0.11** | **75.0%** | **[0.80, 1.60]** |
| Andrews-Trevino, 2021 | 1.08 | [0.93, 1.25] | 0.01 | 0.11 | 54.6% | [0.77, 1.53] |
| Andrews-Trevino, 2022 | 1.06 | [0.95, 1.19] | 0.01 | 0.09 | 56.0% | [0.81, 1.38] |
| Alamu, 2020 | 1.09 | [0.96, 1.23] | 0.01 | 0.11 | 71.8% | [0.79, 1.48] |
| Mahfuz, 2021 | 1.11 | [0.97, 1.26] | 0.01 | 0.11 | 75.0% | [0.81, 1.52] |
| McMillan, 2018 | 1.10 | [0.96, 1.26] | 0.01 | 0.10 | 75.0% | [0.81, 1.50] |
| **Ashraf, 2022** | **1.15** | **[1.01, 1.31]** | **0.01** | **0.08** | **18.1%** | **[0.88, 1.50]** |
| None | 1.10 | [0.98, 1.24] | 0.01 | 0.10 | 70.0% | [0.82, 1.47] |
| **Study omitted** | **CC** | **95% CI** | **Tau^2^** | **Tau** | **I^2^ (%)** | **95% PI** |
| **ALT** | | | | | | |
| Farag, 2018 | 0.51 | [0.20; 0.73] | 0.0122 | 0.1104 | 45.6% | [-0.06; 0.83] |
| Wild, 1993 | 0.61 | [0.42; 0.75] | 0.0 | 0.0 | 0% | [0.37; 0.78] |
| Raafat, 2021 | 0.56 | [0.11; 0.82] | 0.0310 | 0.1761 | 71.5% | [-0.28; 0.91] |
| Abdel-Wahab, 2018 | 0.54 | [0.06; 0.82] | 0.0327 | 0.1808 | 66.1% | [-0.34; 0.92] |
| None | 0.55 | [0.33; 0.72] | 0.0193 | 0.1389 | 57.2% | [0.09; 0.82] |
| **AST** | | | | | | |
| Farag, 2018 | -0.10 | [-0.12; -0.09] | 0 | 0 | 0% | [-0.84; 0.77] |
| Raafat, 2021 | 0.21 | [-1.00; 1.00] | 0.2005 | 0.4478 | 92.7% | [-1.00; 1.00] |
| Abdel-Wahab, 2018 | 0.23 | [-1.00; 1.00] | 0.1918 | 0.4380 | 88.1% | [-1.00; 1.00] |
| None | 0.11 | [-0.68; 0.78] | 0.1238 | 0.3518 | 86.6% | [-0.93; 0.96] |
| **Bilirubin** | | | | | | |
| Wu, 2022 | 0.15 | [-1.00; 1.00] | 1.603 | 4.003 | 88.8% | [-1.00; 1.00] |
| Raafat, 2021 | 0.11 | [-0.99; 1.00] | 975 | 3.123 | 92.0% | [-1.00; 1.00] |
| Abdel-Wahab, 2008 | 0.34 | [-0.32; 0.78] | 0 | 0 | 0% | [-0.52; 0.86] |
| None | 0.21 | [-0.51; 0.76] | 831 | 2.882 | 87.1% | [-0.85; 0.93] |

Note: OR = odds ratio, CC = correlation coefficient, CI = confidence interval, PI = prediction interval, AST = aspartate aminotransferase, ALT = alanine transaminase. In bolt studies with high or uncertain RoB.

# **Table 51. Evidential basis of each health outcome assessed with GRADE: availability of a study design appropriate to the outcome, and supporting biological plausibility.**

| **Outcome** | **Appropriate study design** | **Biological plausibility** | **Evidential basis** |
| --- | --- | --- | --- |
| Acute food intoxication | Yes – outbreak investigation, case series | Direct acute toxicity, well characterised | Both |
| Liver disease | Yes – cohort (HCC) | AFB1-induced TP53 mutation | Both |
| Gallbladder cancer | Yes – cohort (2 of 4 studies) | AFB1 inflammation and DNA mutation; synergy with gallstones | Both |
| Metabolic disease | No – predominantly cross-sectional, mostly without controls | Limited; hepatic oxidative stress and impaired insulin signalling proposed for AFB1/OTA, not established | Neither |
| Neurological disease | No – cross-sectional | Fumonisins neurotoxic in vitro; DON crosses blood–brain barrier | Plausibility only |
| Male reproductive disease | No – cross-sectional | ZEN binds ER-α/ER-β as estrogen mimic | Plausibility only |
| Female reproductive disease | No – cross-sectional | ZEN binds ER-α/ER-β as estrogen mimic | Plausibility only |
| Premature puberty | No – predominantly cross-sectional | ZEN binds ER-α/ER-β as estrogen mimic | Plausibility only |
| Kidney disease | Yes – cohort (nephropathy) | OTA/CIT accumulates and is reabsorbed in the kidney | Both |
| Bladder cancer | No – cross-sectional | OTA concentrated in urine; prolonged urothelial exposure | Plausibility only |
| Lung disease | Yes – cohort (lung cancer) | Partial; unclear for bronchopneumonia and TB | Design only |
| Immunomodulation | Yes – cross-sectional, appropriate for subacute response | Documented immunotoxicity of T2 and DON | Both |
| Kwashiorkor | Yes – cohort and RCT | AFB1 inhibits hepatic protein and albumin synthesis | Both |
| Wasting | Yes – cohort and RCT | Gastrointestinal damage; suppression of IGF-1 | Both |
| Underweight | Yes – cohort and RCT | Gastrointestinal damage; suppression of IGF-1 | Both |
| Stunting | Yes – cohort | Suppression of IGF-1; impaired linear growth | Both |
| Blood disease | No – cross-sectional | T2, DON and PAT target haematopoietic cells | Plausibility only |
| Oesophageal disease | No – cross-sectional | FB1 inhibits ceramide synthase; sphingoid base accumulation | Plausibility only |
| Gastric disease | No – cross-sectional | T2, DON and PAT damage gastric mucosa | Plausibility only |
| Intestinal disease | Yes – cohort (colorectal cancer) | Cytotoxic and genotoxic damage to GI mucosa | Both |
| Adverse birth outcomes | Yes – cohort | Aflatoxins cross the placenta; fumonisins disrupt folate uptake | Both |

Note: "Appropriate study design" denotes a design suited to the time course of the outcome; for acute and subacute outcomes this is not necessarily a longitudinal design. AFB1 = aflatoxin B1; DON = deoxynivalenol; ER = estrogen receptor; FB1 = fumonisin B1; GI = gastrointestinal; HCC = hepatocellular carcinoma; IGF-1 = insulin-like growth factor 1; OTA = ochratoxin A; CIT = citrinin; PAT = patulin; T2 = T-2 toxin; TB = tuberculosis; ZEN = zearalenone.

# **Figure 1. Traffic light plot and Risk of bias (RoB) summary plot for acute food intoxication.**

**Left.** **Traffic light plot** of the RoB assessment created using robvis and the colourblind palette. **Right.** **RoB summary plot** created using robvis and the standard cochrane palette, using the Robis Visualisation Tool (2025).

# **Figure 2. Traffic light plot and Risk of bias (RoB) summary plot for adverse birth outcomes.**

**Left.** **Traffic light plot** of the RoB assessment created using robvis and the colourblind palette. **Right.** **RoB summary plot** created using robvis and the standard cochrane palette, using the Robis Visualisation Tool (2025).

# **Figure 3. Traffic light plot and Risk of bias (RoB) summary plot for female reproductive disease.**

**Left.** **Traffic light plot** of the RoB assessment created using robvis and the colourblind palette. **Right.** **RoB summary plot** created using robvis and the standard cochrane palette, using the Robis Visualisation Tool (2025).

# **Figure 4. Traffic light plot and Risk of bias (RoB) summary plot for oesophageal disease.**

**Left.** **Traffic light plot** of the RoB assessment created using robvis and the colourblind palette. **Right.** **RoB summary plot** created using robvis and the standard cochrane palette, using the Robis Visualisation Tool (2025).

# **Figure 5. Traffic light plot and Risk of bias (RoB) summary plot for gallbladder cancer.**

**Left.** **Traffic light plot** of the RoB assessment created using robvis and the colourblind palette. **Right.** **RoB summary plot** created using robvis and the standard cochrane palette, using the Robis Visualisation Tool (2025).

# **Figure 6. Traffic light plot and Risk of bias (RoB) summary plot for gastric disease.**

**Left.** **Traffic light plot** of the RoB assessment created using robvis and the colourblind palette. **Right.** **RoB summary plot** created using robvis and the standard cochrane palette, using the Robis Visualisation Tool (2025).

# **Figure 7. Traffic light plot and Risk of bias (RoB) summary plot for liver disease.**

**Left.** **Traffic light plot** of the RoB assessment created using robvis and the colourblind palette. **Right.** **RoB summary plot** created using robvis and the standard cochrane palette, using the Robis Visualisation Tool (2025).

.

# **Figure 8. Traffic light plot and Risk of bias (RoB) summary plot for immunomodulation.**

**Left.** **Traffic light plot** of the RoB assessment created using robvis and the colourblind palette. **Right.** **RoB summary plot** created using robvis and the standard cochrane palette, using the Robis Visualisation Tool (2025).

# **Figure 9. Traffic light plot and Risk of bias (RoB) summary plot for male infertility.**

**Left.** **Traffic light plot** of the RoB assessment created using robvis and the colourblind palette. **Right.** **RoB summary plot** created using robvis and the standard cochrane palette, using the Robis Visualisation Tool (2025).

# **Figure 10. Traffic light plot and Risk of bias (RoB) summary plot for intestinal disease.**

**Left.** **Traffic light plot** of the RoB assessment created using robvis and the colourblind palette. **Right.** **RoB summary plot** created using robvis and the standard cochrane palette, using the Robis Visualisation Tool (2025).

# **Figure 11. Traffic light plot and Risk of bias (RoB) summary plot for kidney disease and bladder cancer.**

**Left.** **Traffic light plot** of the RoB assessment created using robvis and the colourblind palette. **Right.** **RoB summary plot** created using robvis and the standard cochrane palette, using the Robis Visualisation Tool (2025).

# **Figure 12. Traffic light plot and Risk of bias (RoB) summary plot for kwashiorkor and marasmus.**

**Left.** **Traffic light plot** of the RoB assessment created using robvis and the colourblind palette. **Right.** **RoB summary plot** created using robvis and the standard cochrane palette, using the Robis Visualisation Tool (2025).

# **Figure 13. Traffic light plot and Risk of bias (RoB) summary plot for lung disease, musculoskeletal disease, pancreatic cancer, male reproductive disease, and mucous fibrosis.**

**Left.** **Traffic light plot** of the RoB assessment created using robvis and the colourblind palette. **Right.** **RoB summary plot** created using robvis and the standard cochrane palette, using the Robis Visualisation Tool (2025).

# **Figure 14. Traffic light plot and Risk of bias (RoB) summary plot for neurological disease.**

**Left.** **Traffic light plot** of the RoB assessment created using robvis and the colourblind palette. **Right.** **RoB summary plot** created using robvis and the standard cochrane palette, using the Robis Visualisation Tool (2025).

# **Figure 15. Traffic light plot and Risk of bias (RoB) summary plot for blood disease.**

**Left.** **Traffic light plot** of the RoB assessment created using robvis and the colourblind palette. **Right.** **RoB summary plot** created using robvis and the standard cochrane palette, using the Robis Visualisation Tool (2025).

# **Figure 16. Traffic light plot and Risk of bias (RoB) summary plot for premature puberty and metabolic disease.**

**Left.** **Traffic light plot** of the RoB assessment created using robvis and the colourblind palette. **Right.** **RoB summary plot** created using robvis and the standard cochrane palette, using the Robis Visualisation Tool (2025).

# **Figure 16. Traffic light plot and Risk of bias (RoB) summary plot for underweight.**

**Left.** **Traffic light plot** of the RoB assessment created using robvis and the colourblind palette. **Right.** **RoB summary plot** created using robvis and the standard Cochrane palette, using the Robis Visualisation Tool (2025).

# **Figure 17. Traffic light plot and Risk of bias (RoB) summary plot for stunting**

**Left.** **Traffic light plot** of the RoB assessment created using robvis and the colourblind palette. **Right.** **RoB summary plot** created using robvis and the standard Cochrane palette, using the Robis Visualisation Tool (2025).

# **Figure 18. Forest plot with random effects model of aflatoxin exposure detected in plasma or serum samples in relation to underweight in children**.

The pooled odds ratio (OR) and [95% confidence interval] was 1.33 [0.33; 5.35], with a prediction interval of [0.08;22.74]. Stratification was performed on study design (cohort and cross-sectional).

# **Figure 19. Forest plot with random effects model of aflatoxin exposure detected in plasma or serum samples in relation to liver cirrhosis.**

The pooled odds ratio (OR) and [95% confidence interval] was 3.98 [1.17; 13.55], with a prediction interval of [0.20;79.69]. Stratification was performed on study design (case-control, cohort and cross-sectional). Chu, 2017 was adjusted for age, gender and serum AST; Alvarez, 2020 was adjusted for sex, ethnicity, hepatitis B status and heavy alcohol consumption; Leonart, 2005 was adjusted but without specification, Kirk, 2000 was adjusted for hepatitis B status, sex, recruitment site.

# **Figure 20. Forest plot with random effects model of aflatoxin exposure detected in plasma or serum samples in relation to gallbladder cancer.**

The pooled odds ratio (OR) and [95% confidence interval] was 3.82 [1.08; 13.53], with a prediction interval of [0.37;39.01]. Stratification was performed on study design (case-control and cross-sectional).

# **Figure 21. Forest plot with random effects model of aflatoxin exposure detected in plasma or serum samples in relation to stunting.**

The pooled odds ratio (OR) and [95% confidence interval] was 1.10 [0.98; 1.24], with a prediction interval of [0.82;1.47]. Stratification was performed on study design (cohort and cross-sectional).

# **Figure 22. Forest plot with random effects model of aflatoxin exposure detected in plasma or serum samples in relation to pregnancy anaemia.**

The pooled odds ratio (OR) and [95% confidence interval] was 1.83 [0.39; 9.17], with a prediction interval of [0.11;30.31]. Stratification was performed on study design (case-control, cohort and cross-sectional).

# **Figure 23. Forest plot with random effects model of aflatoxin exposure detected in plasma or serum samples in relation to hepatocellular carcinoma.**

The pooled odds ratio (OR) and [95% confidence interval] was 2.31 [1.69; 3.15], with a prediction interval of [1.03;5.19]. Stratification was performed on study design (case-control, cohort and cross-sectional). Chu, 2017 was adjusted for age, gender, alcohol consumption and serum AST; Leonart, 2005 was adjusted but without specification, Kirk, 2000 was adjusted for hepatitis B status, sex, recruitment site and age; and Kirk, 2005 was adjusted for age, gender, recruitment site, residence, ethnicity, socio-economic status, hepatitis antigens, and anti- hepatitis C status; Chen, 1996 was adjusted for sex and gender, Rahman El-Zayadi, 2001and Zheng, 2017 were adjusted but not specified; Kimbi, 2005 was adjusted for sex; Sun, 2001 was adjusted for sex, age and residence; Chu,2018 was adjusted for hepatitis C status and gender; Wu, 2007 was adjusted for smoking status, alcohol consumption and hepatitis B status; Wu, 2009 was adjusted for anti-hepatitis C virus, assay batch, smoking, alcohol consumption, body-mass-index and hepatitis B antigen. Other studies did not mention adjustment.

# **Figure 24. Forest plot with random effects model of aflatoxin exposure detected in urine in relation to hepatocellular carcinoma.**

The pooled relative risk (RR) and [95% confidence interval] was 2.66 [1.80; 3.93], with a prediction interval of [1.43;4.95]. Stratification was performed on study design (case-control, cohort and cross-sectional). Sun, 1999 was adjusted for age and hepatitis C status, and Ross,1992 was adjusted for hepatitis B antigen seropositivity, level of education, cigarette smoking and alcohol consumption. Other studies did not mention adjustment.

# **Figure 25. Forest plot with random effects model of aflatoxin exposure detected in plasma or serum samples in relation to AST/ALT/Bilirubin increase.**

The pooled correlation coefficients (cc) and [95% confidence intervals] were estimated for each biomarker, with corresponding prediction intervals. Stratification was performed by study design (case-control, cohort, and cross-sectional). Squares represent individual study estimates weighted by inverse variance, and diamonds represent the pooled estimates. Where reported, studies adjusted for potential confounders; however, adjustment strategies varied across studies and were not consistently specified.

| **Acute food intoxication** | | | | | | |
| --- | --- | --- | --- | --- | --- | --- |
| Study | Mycotoxin | Study design | Acute hepatic failure  or Aflatoxicosis | Alimentary Toxic Leukaemia | Acute Encephalopathy | Acute vomiting |
| **Berry, 1986** | Aflatoxins | **Cross-sectional** | **↔** | **↔** |  |  |
| **Berry, 1986** | Citrinin | **Cross-sectional** | **↔** | **↔** |  |  |
| **Berry, 1986** | Deoxynivalenol | **Cross-sectional** | **↔** | **↔** |  |  |
| **Berry, 1986** | Diacetoxyscirpenol | **Cross-sectional** | **↔** | **↔** |  |  |
| **Berry, 1986** | Fusarium-X | **Cross-sectional** | **↔** | **↔** |  |  |
| **Berry, 1986** | Ochratoxin A | **Cross-sectional** | **↔** | **↔** |  |  |
| **Jolly, 2007** | Aflatoxins | **Cross-sectional** | ▲ |  |  |  |
| **Kamala, 2018** | Aflatoxins | **Outbreak report** | ▲ |  |  |  |
| **Krishnamachari, 1975** | Aflatoxins | **Outbreak report** | ▲ |  |  |  |
| **Lye, 1995** | Aflatoxins | **Case-report** | **↔** |  |  |  |
| **Nyikal, 2004** | Aflatoxins | **Outbreak report** | **↔** |  |  |  |
| **Ruan, 2020** | Deoxynivalenol | **Outbreak report** |  |  |  | ▲ |
| **Samuel, 2009** | Aflatoxins | **Case-report** | **↔** |  |  |  |
| **Shank, 1971** | Aflatoxins | **Cross-sectional** |  |  | ▲ |  |
| **Tzee-Cheng, 1991** | Aflatoxins | **Outbreak report** | **↔** |  |  |  |
| **Wang, 1993** | T_2_HT_2_ | **Case-report** |  |  |  | ▲ |

# **Figure 26. Effect direction plot for acute food intoxication.**

Symbols: ▲ indicates a positive association within the studies. ↔ indicates a non-association or requires a review of the association. ▼ indicates an inverse association. ▲↔▼ indicates total sample size <50. ▲↔▼ indicates total sample size >50 but <300. ▲↔▼indicates total sample size >300.

| **Metabolic disease** | | | | | | |
| --- | --- | --- | --- | --- | --- | --- |
| Study | Mycotoxin | Study design | Type II diabetes | Metabolic syndrome | Elevated hepatic index | Gestational diabetes |
| **Alvarez, 2022** | Aflatoxins | **Cross-sectional** | ▲ | **↔** | **↔** |  |
| **Hamid Akash, 2021** | Aflatoxins | **Cross-sectional** | ▲ |  |  |  |
| **Hseih, 2004** | Ochratoxin A | **Cohort** | ▲ |  |  |  |
| **Wu, 2021** | Aflatoxins | **Cross-sectional** | ▼ |  |  |  |
| **Xie, 2018** | Aflatoxins | **Cross-sectional** |  |  |  | **↔** |
| **Zeng, 2017** | Aflatoxins | **Cross-sectional** | ▲ |  |  |  |

# **Figure 27. Effect direction plot for metabolic disease.**

Symbols: ▲ indicates a positive association within the studies. ↔ indicates a non-association or requires a review of the association. ▼ indicates an inverse association. ▲↔▼ indicates total sample size <50. ▲↔▼ indicates total sample size >50 but <300. ▲↔▼indicates total sample size >300.

| **Neurological diseases** | | | | | | | | | | | |
| --- | --- | --- | --- | --- | --- | --- | --- | --- | --- | --- | --- |
| Study | Mycotoxin | Study design | Alzheimer  Parkinson | Autism | Brain functionality | Dementia | Chronic fatigue syndrome | CISA | Meningitis | Nodding syndrome | Reye’s  Syndrome |
| **Arce-López, 2021** | Aflatoxins | **Cross-sectional** | **↔** |  |  |  |  |  |  |  |  |
| **Arce-López, 2021** | Diacetoxyscirpenol | **Cross-sectional** | **↔** |  |  |  |  |  |  |  |  |
| **Arce-López, 2021** | Deoxynivalenol | **Cross-sectional** | **↔** |  |  |  |  |  |  |  |  |
| **Arce-López, 2021** | Citrinin | **Cross-sectional** | **↔** |  |  |  |  |  |  |  |  |
| **Arce-López, 2021** | Fumonisins | **Cross-sectional** | **↔** |  |  |  |  |  |  |  |  |
| **Arce-López, 2021** | Fusarium-X | **Cross-sectional** | **↔** |  |  |  |  |  |  |  |  |
| **Arce-López, 2021** | Neosolaniol | **Cross-sectional** | **↔** |  |  |  |  |  |  |  |  |
| **Arce-López, 2021** | Nivalenol | **Cross-sectional** | **↔** |  |  |  |  |  |  |  |  |
| **Arce-López, 2021** | Ochratoxin A | **Cross-sectional** | ▲ |  |  |  |  |  |  |  |  |
| **Arce-López, 2021** | Sterigmatocystin | **Cross-sectional** | ▲ |  |  |  |  |  |  |  |  |
| **Arce-López, 2021** | T_2_HT_2_ | **Cross-sectional** | **↔** |  |  |  |  |  |  |  |  |
| **Arce-López, 2021** | Zearalenone | **Cross-sectional** | **↔** |  |  |  |  |  |  |  |  |
| **Berry, 1986** | Aflatoxins | **Cross-sectional** |  |  |  |  |  |  |  |  | **↔** |
| **Berry, 1986** | Citrinin | **Cross-sectional** |  |  |  |  |  |  |  |  | **↔** |
| **Berry, 1986** | Diacetoxyscirpenol | **Cross-sectional** |  |  |  |  |  |  |  |  | **↔** |
| **Berry, 1986** | Deoxynivalenol | **Cross-sectional** |  |  |  |  |  |  |  |  | **↔** |
| **Berry, 1986** | Fusarium-X | **Cross-sectional** |  |  |  |  |  |  |  |  | **↔** |
| **Berry, 1986** | Ochratoxin A | **Cross-sectional** |  |  |  |  |  |  |  |  | **↔** |
| **Becroft, 1972** | Aflatoxins | **Case-report** |  |  |  |  |  |  |  |  | **↔** |
| **Brewer, 2012** | Aflatoxins | **Cross-sectional** |  |  |  |  | ▲ |  |  |  |  |
| **Brewer, 2012** | Ochratoxin A | **Cross-sectional** |  |  |  |  | ▲ |  |  |  |  |
| **Brewer, 2012** | T_2_HT_2_ | **Cross-sectional** |  |  |  |  | ▲ |  |  |  |  |
| **DeSantis, 2017** | Aflatoxins | **Cross-sectional** |  | ▲ |  |  |  |  |  |  |  |
| **DeSantis, 2017** | Deoxynivalenol | **Cross-sectional** |  | ▲ |  |  |  |  |  |  |  |
| **DeSantis, 2017** | Fumonisins | **Cross-sectional** |  | ▲ |  |  |  |  |  |  |  |
| **DeSantis, 2017** | Ochratoxin A | **Cross-sectional** |  | ▲ |  |  |  |  |  |  |  |
| **Duringer, 2021** | Aflatoxins | **Cross-sectional** |  |  |  |  |  |  |  | **↔** |  |
| **Duringer, 2021** | T_2_HT_2_ | **Cross-sectional** |  |  |  |  |  |  |  | **↔** |  |
| **Duringer, 2021** | Zearalenone | **Cross-sectional** |  |  |  |  |  |  |  | **↔** |  |
| **Duringer, 2016** | Zearalenone | **Cross-sectional** |  | **↔** |  |  |  |  |  |  |  |
| **Lefèvre-Arbogast, 2024** | Ochratoxin B | **Cohort** |  |  |  | **↔** |  |  |  |  |  |
| **León, 1998** | Aflatoxins | **Cross-sectional** |  |  |  |  |  | **↔** |  |  |  |
| **León, 1998** | Deoxynivalenol | **Cross-sectional** |  |  |  |  |  | ▲ |  |  |  |
| **León, 1998** | Fumonisins | **Cross-sectional** |  |  |  |  |  | ▲ |  |  |  |
| **León, 1998** | Nivalenol | **Cross-sectional** |  |  |  |  |  | ▲ |  |  |  |
| **León, 1998** | Ochratoxin A | **Cross-sectional** |  |  |  |  |  | **↔** |  |  |  |
| **Mahfuz, 2022** | Aflatoxins | **Cohort** |  |  | ▼ |  |  |  |  |  |  |
| **Nelson, 1980** | Aflatoxins | **Cross-sectional** |  |  |  |  |  |  |  |  | **↔** |
| **Oyelami, 1997** | Aflatoxins | **Case-report** |  |  |  |  |  |  | **↔** |  |  |
| **Rogan, 1985** | Aflatoxins | **Cross-sectional** |  |  |  |  |  |  |  |  | **↔** |
| **Simon, 2003** | Aflatoxins | **Cross-sectional** |  |  | ▼ |  |  |  |  |  |  |
| **Stora, 1983** | Aflatoxins | **Cross-sectional** |  |  |  |  |  |  |  |  | **↔** |
| **Wu, 2022** | Aflatoxins | **Cross-sectional** |  |  |  |  | **↔** |  |  |  |  |
| **Wu, 2022** | Ochratoxin A | **Cross-sectional** |  |  |  |  | **↔** |  |  |  |  |

# **Figure 28. Effect direction plot for neurologic disease.**

Symbols: ▲ indicates a positive association within the studies. ↔ indicates a non-association or requires a review of the association. ▼ indicates an inverse association. ▲↔▼ indicates total sample size <50. ▲↔▼ indicates total sample size >50 but <300. ▲↔▼indicates total sample size >300.

| **Female reproductive disease and cancer** | | | | | | | | |
| --- | --- | --- | --- | --- | --- | --- | --- | --- |
| Study | Mycotoxin | Study design | Breast cancer | Uterus cancer | Ovarian cancer | Endometrial cancer | Polycystic Ovary  Syndrome | Idiopathic Anovulation |
| **Belhassen, 2014** | Zearalenone | **Cohort** | ▲ |  |  |  |  |  |
| **El-Azab, 2010** | Aflatoxins | **Cross-sectional** |  |  |  |  | **↔** | **↔** |
| **Gajecki, 2004** | Zearalenone | **Cross-sectional** |  | **↔** | **↔** |  |  |  |
| **Gadzała-Kopciuch, 2011** | Zearalenone | **Cohort** |  | **↔** |  |  |  |  |
| **Kuciel-Lisieska, 2008** | Zearalenone | **Cross-sectional** | **↔** |  |  |  |  |  |
| **Pillay, 2002** | Zearalenone | **Cross-sectional** | **↔** |  |  |  |  |  |
| **Unicsovics, 2024** | Aflatoxins | **Cohort** |  |  |  | ▲ |  |  |
| **Unicsovics, 2024** | Deoxynivalenol | **Cohort** | **↔** |  |  |  |  |  |
| **Unicsovics, 2024** | Fumonisins | **Cohort** | **↔** |  |  |  |  |  |
| **Unicsovics, 2024** | Ochratoxin A | **Cohort** | **↔** |  |  |  |  |  |
| **Unicsovics, 2024** | T_2_/HT_2_ | **Cohort** | **↔** |  |  |  |  |  |
| **Unicsovics, 2024** | Zearalenone | **Cohort** |  |  |  | ▲ |  |  |

# **Figure 29. Effect direction plot for female reproductive disease and cancer.**

Symbols: ▲ indicates a positive association within the studies. ↔ indicates a non-association or requires a review of the association. ▼ indicates an inverse association. ▲↔▼ indicates total sample size <50. ▲↔▼ indicates total sample size >50 but <300. ▲↔▼indicates total sample size >300.

| **Male infertility** | | | | |
| --- | --- | --- | --- | --- |
| Study | Mycotoxin | Study design | Male infertility | Low semen quality |
| **Ning, 2024** | Beauvericin | **Cross-sectional** | **↔** |  |
| **Ning, 2024** | Citrinin | **Cross-sectional** | ▲ |  |
| **Ning, 2024** | Cyclopiazonic acid | **Cross-sectional** | **↔** |  |
| **Ning, 2024** | Enniatin B | **Cross-sectional** | **↔** |  |
| **Ning, 2024** | Ochratoxin A/B | **Cross-sectional** | ▲ |  |
| **Okonofua, 2024** | Deoxynivalenol | **Cross-sectional** |  | ▲ |
| **Okonofua, 2024** | Ochratoxin A | **Cross-sectional** |  | ▲ |
| **Okonofua, 2024** | Zearalenone | **Cross-sectional** |  | ▲ |
| **Yang, 2024** | Beauvericin | **Cross-sectional** |  | ▲ |
| **Yang, 2024** | Citrinin | **Cross-sectional** |  | ▲ |
| **Yang, 2024** | Enniatin | **Cross-sectional** |  | ▼ |
| **Yang, 2024** | Ochratoxin A | **Cross-sectional** |  | ▲ |
| **Yang, 2024** | Ochratoxin B | **Cross-sectional** |  | ▼ |

# **Figure 30. Effect direction plot for male infertility.**

Symbols: ▲ indicates a positive association within the studies. ↔ indicates a non-association or requires a review of the association. ▼ indicates an inverse association. ▲↔▼ indicates total sample size <50. ▲↔▼ indicates total sample size >50 but <300. ▲↔▼indicates total sample size >300.

| **Premature puberty** | | | | | | | | |
| --- | --- | --- | --- | --- | --- | --- | --- | --- |
| Study | Mycotoxin | Study design | Premature  Thelarche | Precocious Puberty  (central) | Precocious Puberty  (Idiopathic) | Height/weight z-score  at menarche | Time to menarche | Maternal menarche |
| **Asci, 2024** | Zearalenone | **Cross-sectional** | ▲ | ▲ |  |  |  |  |
| **Deng, 2012** | Zearalenone | **Cross-sectional** |  |  | ▲ |  |  | ▲ |
| **Massart, 2008** | Zearalenone | **Cross-sectional** |  | **↔** |  |  |  |  |
| **Rivera-Núñez, 2019** | Zearalenone | **Cohort** |  |  | ▼ | ▼ | ▼ |  |

# **Figure 31. Effect direction plot for premature puberty.**

Symbols: ▲ indicates a positive association within the studies. ↔ indicates a non-association or requires a review of the association. ▼ indicates an inverse association. ▲↔▼ indicates total sample size <50. ▲↔▼ indicates total sample size >50 but <300. ▲↔▼indicates total sample size >300.

| **Testicular and prostate cancer** | | | | |
| --- | --- | --- | --- | --- |
| Study | Mycotoxin | Study design | Testicular cancer | Prostate cancer |
| Fahmy, 2014 | Ochratoxin A | **Cohort** | **↔** |  |
| Ning, 2024 | Ochratoxin A | **Cohort** |  | **↔** |

# **Figure 32. Effect direction plot for testicular and prostate cancer.**

Symbols: ▲ indicates a positive association within the studies. ↔ indicates a non-association or requires a review of the association. ▼ indicates an inverse association. ▲↔▼ indicates total sample size <50. ▲↔▼ indicates total sample size >50 but <300. ▲↔▼indicates total sample size >300.

| **Oesophageal disease** | | | | | | |
| --- | --- | --- | --- | --- | --- | --- |
| Study | Mycotoxin | Study design | Oesophageal squamous cell carcinoma | Oesophageal precancerous lesions | oesophageal cancer | Achalasia |
| Lin, 2020 | Aflatoxins | **Cross-sectional** |  |  |  | **↔** |
| Niknejad, 2021 | Deoxynivalenol | **Cross-sectional** |  |  | **↔** |  |
| Niknejad, 2021 | Fusarium-X | **Cross-sectional** |  |  | **↔** |  |
| Niknejad, 2021 | Neosolaniol | **Cross-sectional** |  |  | **↔** |  |
| Niknejad, 2021 | Nivalenol | **Cross-sectional** |  |  | **↔** |  |
| Niknejad, 2021 | HT2/T2 | **Cross-sectional** |  |  | **↔** |  |
| Niknejad, 2021 | Zearalenone | **Cross-sectional** |  |  | **↔** |  |
| Mulisa, 2025 | Aflatoxins | **Cross-sectional** |  |  | **↔** |  |
| Mulisa, 2025 | Citrinin | **Cross-sectional** |  |  | **↔** |  |
| Mulisa, 2025 | Cyclopiazonic acid | **Cross-sectional** |  |  | **↔** |  |
| Mulisa, 2025 | Deoxynivalenol | **Cross-sectional** |  |  | **↔** |  |
| Mulisa, 2025 | Enniatin | **Cross-sectional** |  |  | **↔** |  |
| Mulisa, 2025 | Multiple-mycotoxin | **Cross-sectional** |  |  | ▲ |  |
| Mulisa, 2025 | Nivalenol | **Cross-sectional** |  |  | **↔** |  |
| Mulisa, 2025 | Ochratoxin A | **Cross-sectional** |  |  | **↔** |  |
| Mulisa, 2025 | Tenuazonic acid | **Cross-sectional** |  |  | ▲ |  |
| Mulisa, 2025 | Zearalenone | **Cross-sectional** |  |  | **↔** |  |
| Wang, 2020 | Aflatoxins | **Cross-sectional** |  | ▲ |  |  |
| Xue, 2019 | Aflatoxins | **Cross-sectional** | ▲ |  |  |  |
| Xue, 2019 | Fumonisins | **Cross-sectional** | ▲ |  |  |  |

# **Figure 33. Effect direction plot for testicular and oesophageal disease.**

Symbols: ▲ indicates a positive association within the studies. ↔ indicates a non-association or requires a review of the association. ▼ indicates an inverse association. ▲↔▼ indicates total sample size <50. ▲↔▼ indicates total sample size >50 but <300. ▲↔▼indicates total sample size >300.

| **Gastro-intestinal diseases** | | | | | | | | | | |
| --- | --- | --- | --- | --- | --- | --- | --- | --- | --- | --- |
| Study | Mycotoxin | Study design | Gastric cancer | Pyloric stenosis | Colorectal cancer | Coeliac disease | Enteropathy | Hirschsprung disease | Typhoid enteritis | Microbiome alternations |
| Ayeni, 2024 | Multiple mycotoxins | **Cross-sectional** |  |  |  |  |  |  |  | **↔** |
| Brera, 2013 | Aflatoxins | **Cross-sectional** |  |  |  | **↔** |  |  |  |  |
| Brera, 2013 | Ochratoxin A | **Cross-sectional** |  |  |  | **↔** |  |  |  |  |
| Brera, 2013 | Aflatoxins | **Cross-sectional** |  |  |  | **↔** |  |  |  |  |
| Chen, 2022 | Aflatoxins | **Cohort** |  |  |  |  | **↔** |  |  |  |
| Chen, 2022 | Fumonisins | **Cohort** |  |  |  |  | **↔** |  |  |  |
| Cirlini, 2016 | Deoxynivalenol | **Cross-sectional** |  |  |  | **↔** |  |  |  |  |
| Cirlini, 2016 | Fumonisins | **Cross-sectional** |  |  |  | **↔** |  |  |  |  |
| Cirlini, 2016 | Zearalenone | **Cross-sectional** |  |  |  | **↔** |  |  |  |  |
| Eom, 2013 | Aflatoxins | **Cross-sectional** | ▲ |  |  |  |  |  |  |  |
| Lisieska-Żołnierczyk, 2025 | Zearalanone | **Cross-sectional** |  |  | ▲ |  |  |  |  |  |
| Liu, 2022 | Aflatoxins | **Cohort** | **↔** |  |  |  |  |  |  |  |
| Oesterle, 2024 | Fumonisins | **Cross-sectional** |  |  |  |  |  |  |  | ▲ |
| Oesterle, 2024 | Nivalenol | **Cross-sectional** |  |  |  |  |  |  |  | **↔** |
| Ouhibi, 2020 | Citrinin | **Cross-sectional** |  |  | **↔** |  |  |  |  |  |
| Ouhibi, 2020 | Patulin | **Cross-sectional** |  |  | **↔** |  |  |  |  |  |
| Oyelami, 1995 | Aflatoxins | **Case-report** |  | **↔** |  |  |  |  |  |  |
| Oyelami, 1997 | Aflatoxins | **Case-report** |  |  |  |  |  |  | **↔** |  |
| Oyelami, 1998 | Aflatoxins | **Case-report** |  | **↔** |  |  |  | **↔** |  |  |
| Roy, 1992 | Aflatoxins | **Cross-sectional** |  |  |  | **↔** |  |  |  |  |

# **Figure 34. Effect direction plot for gastro-intestinal disease.**

Symbols: ▲ indicates a positive association within the studies. ↔ indicates a non-association or requires a review of the association. ▼ indicates an inverse association. ▲↔▼ indicates total sample size <50. ▲↔▼ indicates total sample size >50 but <300. ▲↔▼indicates total sample size >300.

| **Gallbladder, cholangiocarcinoma and pancreatic cancer** | | | | |  |
| --- | --- | --- | --- | --- | --- |
| Study | Mycotoxin | Study design | Gallbladder cancer | Pancreatic cancer | Cholangiocarcinoma |
| **Koshiol, 2017** | Aflatoxins | **Cross-sectional** | ▲ |  |  |
| **Koshiol, 2024** | Aflatoxins | **Cross-sectional** | **↔** |  |  |
| **Nogueira, 2015** | Aflatoxins | **Cross-sectional** | ▲ |  |  |
| **Parkin, 1991** | Aflatoxins | **Cross-sectional** |  |  | **↔** |
| **Rodriguez, 2025** | Aflatoxins | **Cross-sectional** |  | **↔** |  |
| **Rodriguez, 2025** | Citrinin | **Cross-sectional** |  | **↔** |  |
| **Rodriguez, 2025** | Ochratoxin A | **Cross-sectional** |  | **↔** |  |
| **Rodriguez, 2025** | Sterigmatocystin | **Cross-sectional** |  | **↔** |  |
| **Rodriguez, 2025** | Zearalenone | **Cross-sectional** |  | **↔** |  |
| **Yadav, 2025** | Aflatoxins | **Cross-sectional** | ▲ |  |  |

# **Figure 35. Effect direction plot for gallbladder, cholangiocarcinoma and pancreatic cancer.**

Symbols: ▲ indicates a positive association within the studies. ↔ indicates a non-association or requires a review of the association. ▼ indicates an inverse association. ▲↔▼ indicates total sample size <50. ▲↔▼ indicates total sample size >50 but <300. ▲↔▼indicates total sample size >300.

| **Lung diseases** | | | | |
| --- | --- | --- | --- | --- |
| Study | Mycotoxin | Study design | Lung cancer | Bronchopneumonia |
| **Cusumano, 1991** | Aflatoxins | **Cohort** | **↔** | **↔** |
| **Dvorackova, 1981** | Aflatoxins | **Case-report** | ▲ |  |
| **Oyelami, 1997** | Aflatoxins | **Case-series** |  | **↔** |

# **Figure 36. Effect direction plot for lung diseases.**

Symbols: ▲ indicates a positive association within the studies. ↔ indicates a non-association or requires a review of the association. ▼ indicates an inverse association. ▲↔▼ indicates total sample size <50. ▲↔▼ indicates total sample size >50 but <300. ▲↔▼indicates total sample size >300.

| **Kwashiorkor and marasmus** | | | | | |
| --- | --- | --- | --- | --- | --- |
| Study | Mycotoxin | Study design | Kwashiorkor | Marasmic kwashiorkor | Marasmus |
| Adhikari,1994 | Aflatoxins | **Cross-sectional** | **↔** |  |  |
| Apeagyei,1986 | Aflatoxins | **Case-series** | **▲** |  |  |
| Coulter, 1986 | Aflatoxins | **Cross-sectional** | **↔** |  | **↔** |
| Devries, 1987 | Aflatoxins | **Cross-sectional** | **↔** | **↔** | **↔** |
| Devries, 1990 | Aflatoxins | **Cross-sectional** | **↔** | **↔** | **↔** |
| Hatem, 2005 | Aflatoxins | **Cross-sectional** | **↔** |  | **↔** |
| Hendrickse,1982 | Aflatoxins | **Cross-sectional** | **▲** | **▲** | **▲** |
| Hendrickse,1983 | Aflatoxins | **Cross-sectional** | **▲** | **▲** | **▲** |
| Househam, 1991 | Aflatoxins | **Cross-sectional** | **↔** |  | **↔** |
| Lamplugh, 1982 | Aflatoxins | **Cohort** | **↔** | **↔** | **↔** |
| McMillan, 2018 | Aflatoxins | **Cross-sectional** | **▲** |  | **▲** |
| Okoth, 2004 | Aflatoxins | **Cross-sectional** | **↔** |  |  |
| Oyelami, 1995 | Aflatoxins | **Cross-sectional** | **↔** |  | **↔** |
| Oyelami, 1997 | Aflatoxins | **Cross-sectional** | **↔** |  |  |
| Oyelami, 1998 | Aflatoxins | **Cross-sectional** | **↔** |  |  |
| Ramjee, 1992 | Aflatoxins | **Cross-sectional** | **↔** |  | **↔** |
| Tchana, 2010 | Aflatoxins | **Cross-sectional** | **↔** | **↔** |  |

# **Figure 37. Effect direction plot for kwashiorkor and marasmus.**

Symbols: ▲ indicates a positive association within the studies. ↔ indicates a non-association or requires a review of the association. ▼ indicates an inverse association. ▲↔▼ indicates total sample size <50. ▲↔▼ indicates total sample size >50 but <300. ▲↔▼indicates total sample size >300.

| **Kidney disease and bladder cancer** | | | | | | | | | |
| --- | --- | --- | --- | --- | --- | --- | --- | --- | --- |
| Study | Mycotoxin | Study design | Chronic kidney disease | Bladder  Cancer | Nephrotic syndrome | Renal  failure | Renal  impairment | Renal stones | Renal cell carcinoma |
| Abid, 2003 | Ochratoxin A | **Cross-sectional** | **▲** |  | ↔ |  |  |  |  |
| Al-Mhanaa, 2020 | Ochratoxin A | **Cross-sectional** |  |  |  | **▲** |  |  |  |
| Aslam, 2005 | Ochratoxin A | **Cross-sectional** |  | ↔ |  |  |  |  |  |
| Aslam, 2012 | Ochratoxin A | **Cross-sectional** |  | ▼ |  |  |  |  |  |
| Claeys, 2022 | Multiple mycotoxins | **Cross-sectional** |  |  |  |  |  |  | **↔** |
| Desalegn, 2011 | Aflatoxins | **Cross-sectional** | **↔** |  |  |  |  |  |  |
| Desalegn, 2011 | Fumonisins | **Cross-sectional** | **↔** |  |  |  |  |  |  |
| Desalegn, 2011 | Ochratoxin A | **Cross-sectional** | **↔** |  |  |  |  |  |  |
| DiazdelLéon, 2019 | Aflatoxins | **Cross-sectional** |  |  | **▲** |  |  |  |  |
| Dinis, 2007 | Ochratoxin A | **Cohort** | **↔** |  |  |  |  |  |  |
| Fahmy, 2014 | Ochratoxin A | **Cohort** |  |  |  |  |  |  | **↔** |
| Grosso, 2003 | Ochratoxin A | **Cross-sectional** |  | **↔** |  |  |  |  |  |
| Hassan, 2004 | Ochratoxin A | **Cross-sectional** | **↔** |  |  |  |  |  |  |
| Hassan, 2006 | Ochratoxin A | **Cross-sectional** |  |  | **▲** |  |  |  |  |
| Hmaissia Khalifa, 2008 | Ochratoxin A | **Cross-sectional** |  |  | **↔** |  |  |  |  |
| Hmaissia Khalifa, 2012 | Ochratoxin A | **Cross-sectional** |  |  | **▲** |  |  |  |  |
| Hseih, 2004 | Ochratoxin A | **Cohort** | **▲** |  |  |  |  |  |  |
| Karamkhani, 2020 | Aflatoxins | **Cross-sectional** | **▲** |  |  |  | **↔** |  |  |
| Kareem, 2021 | Aflatoxins | **Cross-sectional** | **↔** |  |  |  |  |  |  |
| Kosicki, 2021 | Ochratoxin A | **Cross-sectional** |  |  |  | ↔ |  |  |  |
| Lin, 2016 | Aflatoxins | **Cross-sectional** | **↔** |  |  |  |  |  |  |
| Maaroufi, 1995 | Ochratoxin A | **Cross-sectional** |  |  | **▲** |  |  |  |  |
| Malir, 2019 | Citrinin | **Cross-sectional** |  |  |  |  |  |  | ↔ |
| Malir, 2019 | Ochratoxin A | **Cross-sectional** |  |  |  |  |  |  | ↔ |
| MohRedzwan, 2014 | Aflatoxins | **Cross-sectional** |  |  | **▲** |  |  |  |  |
| Oyelami, 1995 | Aflatoxins | **Cross-sectional** |  |  | **↔** | **↔** |  |  |  |
| Oyelami, 1997 | Aflatoxins | **Case-report** | **↔** |  |  |  |  |  |  |
| Oyelami, 1998 | Aflatoxins | **Cross-sectional** |  |  |  | **↔** |  |  |  |
| Özçelik,2011 | Ochratoxin A | **Cross-sectional** | **↔** | **↔** | **↔** | **↔** |  | **↔** |  |
| Sangare-tirogri, 2006 | Ochratoxin A | **Cross-sectional** |  |  | **↔** |  |  |  |  |
| Schwerdt, 2001 | Ochratoxin A | **Cross-sectional** | **↔** |  | **↔** |  |  |  |  |
| Smpokou, 2019 | Ochratoxin A | **Cross-sectional** |  |  | **↔** |  |  |  |  |
| Thé, 2015 | Ochratoxin A | **Cross-sectional** | **↔** | ↔ |  |  |  |  |  |
| Wafa, 1998 | Ochratoxin A | **Cross-sectional** |  |  | **▲** |  |  |  |  |
| Yordanova, 2012 | Ochratoxin A | **Cross-sectional** |  |  | **▲** |  |  |  |  |
| Zaied, 2011 | Ochratoxin A | **Cross-sectional** |  |  | **▲** |  |  |  |  |

# **Figure 38. Effect direction plot for kidney disease and bladder cancer.**

Symbols: ▲ indicates a positive association within the studies. ↔ indicates a non-association or requires a review of the association. ▼ indicates an inverse association. ▲↔▼ indicates total sample size <50. ▲↔▼ indicates total sample size >50 but <300. ▲↔▼indicates total sample size >300.

| **Adverse birth outcomes** | | | | | | |
| --- | --- | --- | --- | --- | --- | --- |
| Study | Mycotoxin | Study design | Low birth weight | Preterm birth | Small-for-gestational age | Microcephaly |
| Abdulrazzaq, 2004 | Aflatoxins | **Cross-sectional** | **↔** |  |  |  |
| Andrews-Trevino 2019 | Aflatoxins | **Cohort** |  | **▲** |  |  |
| Andrews-Trevino 2022 | Aflatoxins | **Cohort** |  |  |  | **↔** |
| Andrews-Trevino 2022 | Deoxynivalenol | **Cohort** |  |  |  | **↔** |
| Andrews-Trevino 2022 | Fumonisins | **Cohort** |  |  |  | **↔** |
| Andrews-Trevino 2022 | Ochratoxin A | **Cohort** |  |  |  | **↔** |
| Bastos-Moreira 2025 | Ochratoxin A | **Cohort** | **↔** |  |  | **▲** |
| Kinkade 2024 | Zearalenone | **Cohort** | **↔** |  |  |  |
| Lauer, 2019 | Aflatoxins | **Cohort** |  |  |  | **▲** |
| Raafat, 2021 | Aflatoxins | **Cross-sectional** | **↔** |  |  |  |
| Shuaib, 2010 | Aflatoxins | **Cross-sectional** | **▲** | **↔** | **↔** |  |
| Tan 2023 | Deoxynivalenol | **Cohort** | **▲** |  | **▲** |  |
| Tesfamariam, 2022 | Multiple mycotoxin | **Cross-sectional** |  | **↔** | **↔** |  |
| Zhong, 2024 | Aflatoxins | **Cohort** | **↔** | **↔** |  |  |

# **Figure 39. Effect direction plot for adverse birth outcomes.**

Symbols: ▲ indicates a positive association within the studies. ↔ indicates a non-association or requires a review of the association. ▼ indicates an inverse association. ▲↔▼ indicates total sample size <50. ▲↔▼ indicates total sample size >50 but <300. ▲↔▼indicates total sample size >300.

| **Wasting** | | | |
| --- | --- | --- | --- |
| Study | Mycotoxin | Study design | Wasting |
| Alamu, 2020 | Aflatoxins | **Cross-sectional** | **▲** |
| Bastos-Moreira 2025 | Ochratoxin A | **Cohort** | **↔** |
| Magoha, 2014 | Aflatoxins | **Cross-sectional** | **↔** |
| Matchado 2023 | Aflatoxins | **Cross-sectional** | **▲** |
| Nasir, 2021 | Aflatoxins | **Cross-sectional** | **↔** |

# **Figure 40. Effect direction plot for wasting.**

Symbols: ▲ indicates a positive association within the studies. ↔ indicates a non-association or requires a review of the association. ▼ indicates an inverse association. ▲↔▼ indicates total sample size <50. ▲↔▼ indicates total sample size >50 but <300. ▲↔▼indicates total sample size >300.

| **Musculoskeletal and mucous fibrosis disease** | | | | |
| --- | --- | --- | --- | --- |
| Study | Mycotoxin | Study design | Kashin-Beck disease | Oral Mucous Fibrosis |
| **Bhowate, 2021** | Aflatoxins | **Cross-sectional** |  | **▲** |
| **Ning, 2023** | T_2_HT_2_ | **Case-control** | **▲** |  |

# **Figure 41. Effect direction plot for musculoskeletal and mucous fibrosis disease.**

Symbols: ▲ indicates a positive association within the studies. ↔ indicates a non-association or requires a review of the association. ▼ indicates an inverse association. ▲↔▼ indicates total sample size <50. ▲↔▼ indicates total sample size >50 but <300. ▲↔▼indicates total sample size >300.

| **Immunomodulation** | | | | |
| --- | --- | --- | --- | --- |
| Study | Mycotoxin | Study design | Immune activation | Immune decrease |
| Turner, 2003 | Aflatoxin | Cross-sectional |  | ▲ |
| Xu, 2021 | Aflatoxin | Cohort |  | **↔** |
| Wood ,2017 | Ochratoxin A | case-control | ▲ |  |
| Jiang, 2005 | Aflatoxin | Cross-sectional |  | ▲ |
| Jolly, 2011 | Aflatoxin | Cross-sectional | ▲ |  |

# **Figure 42. Effect direction plot for immunomodulation.**

Symbols: ▲ indicates a positive association within the studies. ↔ indicates a non-association or requires a review of the association. ▼ indicates an inverse association. ▲↔▼ indicates total sample size <50. ▲↔▼ indicates total sample size >50 but <300. ▲↔▼indicates total sample size >300.

| **Stunting and underweight** | | | | |
| --- | --- | --- | --- | --- |
| Study | Mycotoxin | Study design | Stunting | Underweight |
| Alamu, 2020 | Aflatoxin | Cross-sectional | ▲ | ↔ |
| Andrews-Trevino, 2019 | Aflatoxin | Cohort | ▲ | ↔ |
| Andrews-Trevino, 2021 | Aflatoxin | cohort | ▲ |  |
| Andrews-Trevino 2022 | Aflatoxin | Cohort | ▲ | ▲ |
| Andrews-Trevino 2022 | Ochratoxin A | Cohort | ↔ | ↔ |
| Andrews-Trevino 2022 | Fumonisins | Cohort | ↔ | ▲ |
| Andrews-Trevino 2022 | Deoxynivalenol | Cohort | ↔ | ↔ |
| Bastos-Moreira 2025 | Ochratoxin A | Cohort | ↔ | ↔ |
| Chen, 2018 | Aflatoxin | Cross-sectional | ↔ | ↔ |
| Chen, 2018 | Fumonisins | Cross-sectional | ↔ | ▲ |
| Gichohi-Wainaina, 2023 | Aflatoxin | Cross-sectional | ▲ | ▲ |
| Gong, 2002 | Aflatoxin | Cross-sectional | ▲ | ▲ |
| Gong, 2004 | Aflatoxin | Cohort | ▲ |  |
| Hoffmann, 2018 | Aflatoxin | Cross-sectional | ↔ |  |
| Huong, 2019 | Aflatoxin | Cross-sectional | ▲ |  |
| Huong, 2019 | Fumonisins | Cross-sectional | ▲ |  |
| Huong, 2019 | Ochratoxin A | Cross-sectional | ▲ |  |
| Huong, 2019 | Multiple mycotoxin | Cross-sectional | ▲ |  |
| Kamala, 2018 | Aflatoxin | RCT |  | ▲ |
| Kamala, 2018 | Fumonisins | RCT |  | ▲ |
| Kimanya, 2010 | Fumonisins | Cross-sectional | ▲ |  |
| Lauer, 2019 | Aflatoxin | Cross-sectional | ↔ | ↔ |
| Lauer, 2020 | Aflatoxin | Cohort |  | ↔ |
| Leroy, 2018 | Aflatoxin | Cohort | ▲ |  |
| Magoha, 2014 | Aflatoxin | Cross-sectional | ▲ | ▲ |
| Mahdavi, 2010 | Aflatoxin | Cross-sectional | ▲ | ↔ |
| Mahfuz,2021 | Aflatoxin | Cohort | ▲ |  |
| Matchado, 2023 | Aflatoxin | Cross-sectional | ▲ | ↔ |
| Memiş, 2020 | Ochratoxin A | Cross-sectional | ↔ |  |
| Memiş, 2020 | Aflatoxin | Cross-sectional | ↔ |  |
| Memiş, 2020 | Deoxynivalenol | Cross-sectional | ↔ |  |
| Memiş, 2020 | Zearalanone | Cross-sectional | ↔ |  |
| Mitchell, 2017 | Aflatoxin | Cohort | ▲ |  |
| NabwireWangia-Dixon 2020 | Aflatoxin | Cross-sectional | ↔ | ↔ |
| Nasir, 2021 | Aflatoxin | Cross-sectional | ↔ | ▲ |
| NjumbeEdiage, 2013 | Ochratoxin A | Cross-sectional | ↔ | ↔ |
| NjumbeEdiage, 2013 | Aflatoxin | Cross-sectional | ↔ | ↔ |
| NjumbeEdiage, 2013 | Deoxynivalenol | Cross-sectional | ↔ | ↔ |
| NjumbeEdiage, 2013 | Fumonisins | Cross-sectional | ↔ | ↔ |
| NjumbeEdiage, 2013 | Zearalanone | Cross-sectional | ↔ | ↔ |
| Shirima, 2015 | Aflatoxin | Cohort | ▲ | ▲ |
| Shirima, 2015 | Fumonisins | Cohort | ▲ | ▲ |
| Shouman, 2012 | Aflatoxin | Cross-sectional | ▲ | ↔ |
| Tesfamariam, 2022 | Aflatoxin | Cross-sectional |  | ▲ |
| Tessema, 2021 | Aflatoxin | Cross-sectional | ↔ |  |
| Turner, 2007 | Aflatoxin | Cross-sectional | ▲ | ▲ |
| Voth-Gaeddert 2018 | Aflatoxin | Cross-sectional | ▲ |  |
| Watson, 2018 | Aflatoxin | Cross-sectional | ▲ | ▲ |

# **Figure 43. Effect direction plot for stunting and underweight.**

Symbols: ▲ indicates a statistically significant association within the studies. ↔ indicates a non-significant association or requires a review of the association. ▼ Symbols: ▲ indicates a positive association within the studies. ↔ indicates a non-association or requires a review of the association. ▼ indicates an inverse association. ▲↔▼ indicates total sample size <50. ▲↔▼ indicates total sample size >50 but <300. ▲↔▼indicates total sample size >300.

| **Liver diseases** | | | | | | | | | |
| --- | --- | --- | --- | --- | --- | --- | --- | --- | --- |
| **Liver pathologies** | | | | | | | | | |
| Study | Mycotoxin | Study design | Liver enzyme increase | Hepatocellular carcinoma | Neonatal jaundice | Liver cirrhosis | Miscellaneous liver disease | Chronic Hepatomegaly | Fatty or bilharzial liver |
| Anitha, 2014 | Aflatoxins | Cross-sectional |  |  |  | ▲ |  |  |  |
| Abdel-Wahab,2008 | Aflatoxins | Cross-sectional |  | ▲ |  |  |  |  |  |
| Abedi-Ardekani, 2011 | R249 TP53 | Case-series |  | ↔ |  |  |  |  |  |
| Afum,2016 | Aflatoxins | Cross-sectional |  | ↔ |  |  |  |  |  |
| Ahmed, 1995 | Aflatoxins | Cross-sectional |  |  | ▲ |  |  |  |  |
| Alvarez, 2020 | Aflatoxins | Cross-sectional |  |  |  | ▲ |  |  |  |
| Amla 1971 | Aflatoxins | Case series |  |  |  | ▲ |  |  |  |
| Ankrah, 1994 | Aflatoxins | Cross-sectional | ↔ |  |  |  |  |  |  |
| Asim, 2011 | Aflatoxins | Cross-sectional |  | ▲ |  |  |  |  |  |
| Aydın, 2015 | Aflatoxins | Cross-sectional |  | ↔ |  |  |  |  |  |
| Bessard, 1990 | Aflatoxins | Case report |  | ↔ |  |  |  |  |  |
| Buetow, 1992 | R249 TP53 | Case series |  | ▲ |  |  |  |  |  |
| Bulatao-Jayme, 1982 | Aflatoxins | Case-control |  | ▲ |  |  |  |  |  |
| Campbell, 1990 | Aflatoxins | Cross-sectional |  | ↔ |  |  |  |  |  |
| Chan, 1990 | Aflatoxins | Cross-sectional |  | ↔ |  |  |  |  |  |
| Chen, 1996 | Aflatoxins | Cohort |  | ▲ |  |  |  |  |  |
| Chen, 2007 | Aflatoxins | Cohort |  | ▲ |  |  |  |  |  |
| Chu, 2017 | Aflatoxins | Cross-sectional |  | ▲ |  | ▲ |  |  |  |
| Chu, 2018 | Aflatoxins | Cross-sectional |  | ▲ |  |  |  |  |  |
| Coulter,1986 | Aflatoxins | Cross-sectional |  |  |  |  | ▲ |  |  |
| deCarvalho, 2013 | R249 TP53 | Case series |  | ▲ |  | ▲ |  |  |  |
| deCarvalho, 2013 | Aflatoxins | Case series |  | ▲ |  | ▲ |  |  |  |
| Deng, 1998 | Aflatoxins | Cross-sectional |  | ↔ |  |  |  |  |  |
| Devries, 1989 | Aflatoxins | Cohort |  | ▲ |  |  |  |  |  |
| El-Kafrawy, 2005 | R249 TP53 | Case report |  | ↔ |  |  |  |  |  |
| El-Shahat,2012 | Aflatoxins | Case-control |  | ▲ |  |  |  |  |  |
| El-Shanawani, 2006 | Aflatoxins | Cross-sectional |  | ▲ |  | ▲ |  |  |  |
| El-Shanawani, 2006 | R249 TP53 | Cross-sectional |  | ▲ |  | ▲ |  |  |  |
| Farag, 2018 | Aflatoxins | Case-control | ▲ |  |  |  |  |  |  |
| Gong,2012 | Aflatoxins | Cross-sectional |  |  |  |  |  | ▲ |  |
| Hollstein, 1993 | R249 TP53 | Case series |  | ▲ |  |  |  |  |  |
| Hoque, 1999 | Aflatoxins | Cross-sectional |  | ▲ |  |  |  |  |  |
| Hosny, 2008 | R249 TP53 | Cross-sectional |  | ↔ |  |  |  |  |  |
| Huţanaşu,2011 | Sterigmatocystin | Cross-sectional |  | ▲ |  |  |  |  |  |
| Huybrechts 2024 | Aflatoxins | Cross-sectional |  | ↔ |  |  |  |  |  |
| Huybrechts 2024 | Deoxynivalenol | Cross-sectional |  | ▲ |  |  |  |  |  |
| Huybrechts 2024 | Citrinin | Cross-sectional |  | ↔ |  |  |  |  |  |
| Huybrechts 2024 | Enniatins | Cross-sectional |  | ↔ |  |  |  |  |  |
| Huybrechts 2024 | Fumonisins | Cross-sectional |  | ↔ |  |  |  |  |  |
| Huybrechts 2024 | Fusarium-X | Cross-sectional |  | ↔ |  |  |  |  |  |
| Huybrechts 2024 | Multiple mycotoxin | Cross-sectional |  | ▲ |  |  |  |  |  |
| Huybrechts 2024 | Ochratoxins | Cross-sectional |  | ↔ |  |  |  |  |  |
| Huybrechts 2024 | Sterigmatocystin | Cross-sectional |  | ↔ |  |  |  |  |  |
| Huybrechts 2024 | HT2/T2 | Cross-sectional |  | ↔ |  |  |  |  |  |
| Huybrechts 2024 | Zearalenone | Cross-sectional |  | ↔ |  |  |  |  |  |
| Ibrahim, 2013 | Ochratoxin A | Cross-sectional |  | ↔ |  |  |  |  |  |
| Ilić, 2016 | Aflatoxins | Case report |  | ↔ |  |  |  |  |  |
| Jiang, 1998 | R249 TP53 | Cross-sectional |  | ▲ |  |  |  |  |  |
| Jolly, 2011 | Aflatoxins | Cross-sectional | ↔ |  |  |  |  |  |  |
| Karamkhani, 2020 | Aflatoxins | Cross-sectional | ▲ |  |  |  |  |  |  |
| Kimbi, 2005 | R249 TP53 | Cross-sectional |  | ▲ |  |  |  |  |  |
| Kirk, 2000 | R249 TP53 | Cross-sectional |  | ▲ |  | ↔ |  |  |  |
| Kirk, 2005 | R249 TP53 | Cross-sectional |  | ↔ |  | ↔ |  |  |  |
| Lino-Silva, 2022 | Aflatoxins | Cross-sectional |  | ▲ |  |  |  |  |  |
| Liu, 2008 | Aflatoxins | Cross-sectional |  | ▲ |  |  |  |  |  |
| Liu, 2017 | Aflatoxins | Cross-sectional | ▲ |  |  |  |  |  |  |
| Lleonart, 2005 | R249 TP53 | Cross-sectional |  | ▲ |  | ↔ |  |  |  |
| Long, 2008 | R249 TP53 | Cross-sectional |  | ↔ |  |  |  |  |  |
| López, 2002 | Aflatoxins | Cross-sectional |  |  |  | ↔ |  |  |  |
| Lu, 2010 | Aflatoxins | Cross-sectional |  | ▲ |  |  |  |  |  |
| Lu,1998 | Aflatoxins | Cross-sectional |  | ▲ |  |  |  |  |  |
| Lunn, 1997 | R249 TP53 | Cross-sectional |  | ▲ |  |  |  |  |  |
| Manda 2018 | Aflatoxins | Cross-sectional |  | ↔ |  |  |  |  |  |
| McGlynn, 1995 | Aflatoxins | Cross-sectional |  | ▲ |  |  |  |  |  |
| Ming,2002 | Aflatoxins | Cross-sectional |  | ↔ |  |  |  |  |  |
| Mizrak,2009 | Aflatoxins | Cross-sectional |  | ▲ |  | ▲ |  |  |  |
| MohdRedzwan, 2014 | Aflatoxins | Cross-sectional | ▲ |  |  |  |  |  |  |
| Mokhles,2007 | Aflatoxins | Cross-sectional |  | ▲ |  | ▲ |  |  |  |
| Murugavel, 2007 | Aflatoxins | Cross-sectional |  | ▲ |  |  |  |  |  |
| Nasir, 2021 | Aflatoxins | Cross-sectional | ▲ |  |  |  |  |  |  |
| Ng, 1994 | Aflatoxins | Cross-sectional |  | ↔ |  |  |  |  |  |
| Ngindu, 1982 | Aflatoxins | Cohort |  |  |  | ↔ |  |  |  |
| Nogueira, 2009 | R249 TP53 | Cohort |  | ▲ |  |  |  |  |  |
| Olubuyide, 1992 | Aflatoxins | Cross-sectional |  | ▲ |  |  |  |  |  |
| Onyemelukwe,1982 | Aflatoxins | Cross-sectional |  | ▲ |  |  |  |  |  |
| Ortiz-Cuaran, 2013 | R249 TP53 | Cross-sectional |  | ▲ |  | ↔ |  |  |  |
| Ozdemir, 2010 | R249 TP53 | Cross-sectional |  | ↔ |  | ▲ |  |  |  |
| Ozturk, 1991 | Aflatoxins | Cross-sectional |  | ↔ |  |  |  |  |  |
| Peers, 1973 | Aflatoxins | Case series |  | ▲ |  |  |  |  |  |
| Peers,1987 | Aflatoxins | Cross-sectional |  | ▲ |  |  |  |  |  |
| Persson, 2012 | Aflatoxins | Cross-sectional |  | ↔ |  |  |  |  |  |
| Qian, 1994 | Aflatoxins | Cross-sectional |  | ▲ |  |  |  |  |  |
| Raafat, 2021 | Aflatoxins | Cross-sectional | ▲ |  |  |  |  |  |  |
| RahmanEl-Zayadi, 2001 | Aflatoxins | Cross-sectional |  | ↔ |  |  |  |  |  |
| Ramalho, 2018 | Aflatoxins | Cross-sectional |  | ▲ |  | ▲ |  |  |  |
| Ross,1992 | Aflatoxins | Cross-sectional |  | ▲ |  |  |  |  |  |
| Sayed, 2005 | Aflatoxins | Cross-sectional |  |  |  |  |  |  | ↔ |
| Shimizu, 1999 | R249 TP53 | Case series |  | ↔ |  |  |  |  |  |
| Shirabe, 2011 | Aflatoxins | Case series |  | ▲ |  |  |  |  |  |
| Sodeinde, 1995 | Aflatoxins | Cross-sectional |  | ↔ | ▲ |  |  |  |  |
| Soini,1996 | R249 TP53 | Case series |  | ▲ |  |  |  |  |  |
| Srivatanakul, 1991 | Aflatoxins | Cross-sectional |  | ↔ |  |  |  |  |  |
| Stora, 1981 | Aflatoxins | Cross-sectional |  | ↔ |  |  |  |  |  |
| Stora, 1987 | Aflatoxins | Caser series |  | ▲ |  |  |  |  |  |
| Sun,  1999 | Aflatoxins | Cohort |  | ▲ |  |  |  |  |  |
| Sun, 2001 | Aflatoxins | Cross-sectional |  | ▲ |  |  |  |  |  |
| Szymañska, 2009 | Aflatoxins | Cohort |  | ↔ |  |  |  |  |  |
| Tan, 1995 | Aflatoxins | Case-control |  | ▲ |  |  |  |  |  |
| Tchana, 2010 | Aflatoxins | Cohort |  | ↔ |  |  |  |  |  |
| Tian, 1995 | Sterigmatocystin | Cross-sectional |  | ▲ |  |  |  |  |  |
| Tomerak,2011 | Aflatoxins | Cross-sectional |  | ↔ |  |  |  |  |  |
| Wild, 1993 | Aflatoxins | Cohort |  | ▲ |  |  |  |  |  |
| Wray, 1980 | Aflatoxins | Case report |  | ▲ |  |  |  |  |  |
| Wu, 2007 | Aflatoxins | Cross-sectional |  | ↔ |  |  |  |  |  |
| Wu, 2009 | Aflatoxins | Cross-sectional |  | ▲ |  |  |  |  |  |
| Wu, 2022 | Aflatoxins | Cross-sectional | ▲ | ▲ |  |  |  |  |  |
| Yadgiri, 1970 | Aflatoxins | Cross-sectional |  |  |  | ▲ |  |  |  |
| Yeh, 2210 | R249 TP53 | Case series |  | ↔ |  |  |  |  |  |
| Yu, 1992 | Aflatoxins | Cross-sectional |  | ▲ |  |  |  |  |  |
| Yu, 1997 | Aflatoxins | Cross-sectional |  | ▲ |  |  |  |  |  |
| Zeng, 2017 | Aflatoxins | Cross-sectional |  | ▲ |  |  |  |  |  |
| Zhou 1996 | Aflatoxins | Cross-sectional |  | ▲ |  |  |  |  |  |

# **Figure 44. Effect direction plot for liver diseases.**

Symbols: ▲ indicates a positive association within the studies. ↔ indicates a non-association or requires a review of the association. ▼ indicates an inverse association. ▲↔▼ indicates total sample size <50. ▲↔▼ indicates total sample size >50 but <300. ▲↔▼indicates total sample size >300.

| **Blood diseases** | | | | | | |
| --- | --- | --- | --- | --- | --- | --- |
| Study | Mycotoxin | Study design | Burkit Lymphoma | Pregnancy haemorrhage | Pregnancy anaemia | Pre-eclampsia |
| Chen, 2022 | Aflatoxins | **Cross-sectional** |  | ▲ |  |  |
| Lei, 2021 | Aflatoxins | **Cohort** |  |  | ▲ |  |
| Moodley, 2001 | Fumonisins | **Cross-sectional** |  |  |  | ↔ |
| Murashiki, 2025 | Aflatoxins | **Cohort** |  |  | **↔** |  |
| Murashiki, 2025 | Fumonisins | **Cohort** |  |  | ▲ |  |
| Osoro, 2024 | Aflatoxins | **Cohort** |  |  | **↔** |  |
| Oyelami, 1995 | Aflatoxins | **Case report** | ▲ |  |  |  |
| Oyelami, 1995 | Aflatoxins | **Case report** | ▲ |  |  |  |
| Oyelami, 1997 | Aflatoxins | **Outbreak report** | ▲ |  |  |  |
| Pratiwi, 2024 | Aflatoxins | **Cohort** |  |  | ▲ |  |
| Shuaib, 2010 | Aflatoxins | **Cross-sectional** |  |  | ▲ |  |

# **Figure 45. Effect direction plot for blood diseases.**

Symbols: ▲ indicates a positive association within the studies. ↔ indicates a non-association or requires a review of the association. ▼ indicates an inverse association. ▲↔▼ indicates total sample size <50. ▲↔▼ indicates total sample size >50 but <300. ▲↔▼indicates total sample size >300.

**References**

1	Schamann A, Schmidt-Heydt M, Geisen R, Kulling SE, Soukup ST, Afb A. Formation of B-and M-group aflatoxins and precursors by Aspergillus flavus on maize and its implication for food safety. *Mycotoxin Res* 2022; **1**: 3.

2	Schrenk D, Bignami M, Bodin L, *et al.* Risk assessment of aflatoxins in food. *EFSA J* 2020; **18**: 1–112.

3	Chrevatidis A. MYCOTOXINS | Occurrence and Determination. *Encycl Food Sci Nutr* 2003; : 4089–96.

4	Frisvad JC, Møller LLH, Larsen TO, Kumar R, Arnau J. Safety of the fungal workhorses of industrial biotechnology: update on the mycotoxin and secondary metabolite potential of Aspergillus niger, Aspergillus oryzae, and Trichoderma reesei. *Appl Microbiol Biotechnol 2018 10222* 2018; **102**: 9481–515.

5	Martins LM, Sant’Ana AS, Fungaro MHP, *et al.* The biodiversity of Aspergillus section Flavi and aflatoxins in the Brazilian peanut production chain. *Food Res Int* 2017; **94**: 101–7.

6	Kagot V, De Boevre M, Landschoot S, Obiero G, Okoth S, De Saeger S. Comprehensive analysis of multiple mycotoxins and Aspergillus flavus metabolites in maize from Kenyan households. *Int J Food Microbiol* 2022; **363**: 109502.

7	Polak-Sliwinska M, Paszczyck B. Trichothecenes in Food and Feed, Relevance to Human and Animal Health and Methods of Detection: A Systematic Review. *Molecules* 2021; **454**: 1–22.

8	Pierron A, Alassane-Kpembi I, Oswald IP, *et al.* Fumonisins: Impact on Agriculture, Food, and Human Health and their Management Strategies Madhu. *Porc Heal Manag* 2016; **29**: 1–8.

9	EFSA. Scientific Opinion on the risks to human and animal health related to the presence of beauvericin and enniatins in food and feed. *EFSA J* 2014; **12**. DOI:10.2903/j.efsa.2014.3802.

10	Ropejko K, Twarużek M. Zearalenone and Its Metabolites—General Overview, Occurrence, and Toxicity. *Toxins (Basel)* 2021; **13**. DOI:10.3390/TOXINS13010035.

11	Knutsen HK, Alexander J, Barregård L, *et al.* Risk to human and animal health related to the presence of 4,15-diacetoxyscirpenol in food and feed. *EFSA J* 2018; **16**: e05367.

12	Streit E, Schwab C, Sulyok M, Naehrer K, Krska R, Schatzmayr G. Multi-mycotoxin screening reveals the occurrence of 139 different secondary metabolites in feed and feed ingredients. *Toxins (Basel)* 2013; **5**: 504–23.

13	Ismaiel AA, Papenbrock J. Mycotoxins: Producing Fungi and Mechanisms of Phytotoxicity. *Agriculture* 2015; **5**: 492–537.

14	Han Z, Tangni EK, Huybrechts B, *et al.* Screening survey of co-production of fusaric acid, fusarin C, and fumonisins B 1 , B 2 and B 3 by Fusarium strains grown in maize grains. *Mycotoxin Res* 2014; **30**: 231–40.

15	EFSA. Risks to human and animal health related to the presenceof moniliformin in food and feed. *EFSA J* 2017; **16**: 5082.

16	Zorzete P, Baquião AC, Atayde DD, Reis TA, Gonçalez E, Corrêa B. Mycobiota, aflatoxins and cyclopiazonic acid in stored peanut cultivars. *Food Res Int* 2013; **52**: 380–6.

17	Ostry V, Malir F, Ruprich J. Producers and Important Dietary Sources of Ochratoxin A and Citrinin. *Toxins (Basel)* 2013; **5**: 1574.

18	Patriarca A. Alternaria in food products. *Curr Opin Food Sci* 2016; **11**: 1–9.

19	Otero C, Arredondo C, Echeverriá-Vega A, Gordillo-Fuenzalida F. Penicillium spp. mycotoxins found in food and feed and their health effects. *World Mycotoxin J* 2020; **13**: 323–43.

20	Mizutani K, Kumagai S, Mochizuki N, Kitagawa Y, Sugita-Konishi Y. Determination of a yellow rice toxin, luteoskyrin, in rice by using liquid chromatography-tandem mass spectrometry with electrospray ionization. *J Food Prot* 2009; **72**: 1321–6.

21	Mahato DK, Kamle M, Sharma B, *et al.* Patulin in food: A mycotoxin concern for human health and its management strategies. *Toxicon* 2021; **198**: 12–23.

22	Nicholson P. Mycotoxins in food. Cornwall, England: Woodhead Publishing, 2004 www.woodhead-publishing.com (accessed Jan 3, 2020).

23	Krska R, Crews C. Food Additives and Contaminants Significance, chemistry and determination of ergot alkaloids: A review R. Krska & C. Crews. *Food additves Contam* 2008; **25**: 722–31.

24	EFSA. Scientific Opinion on the risks for animal and public health related to the presence of phomopsins in feed and food. *EFSA J* 2012; **10**: 1–52.

25	Peers FG. Aflatoxin in relation to the epidemiology of human liver cancer. *Zentralblatt fur Bakteriol Mikrobiol und Hyg - Abt 1 Orig A* 1980; **246**: 279–89.

26	Godin M, JP F, Simon P, Francois A, FL R, JP M. Is ochratoxin a nephrotoxic in human beings? *Adv Nephrol Necker Hosp* 1997; **26**: 181–206.

27	Munsterer O. [Ergot poisoning, a national epidemic in the Middle Ages]. *Munch Med Wochenschr* 1955; **97**: 1035–6.

28	Dvorackova I, Vesely D, Kusak V. Aflatoxin in the pathology of early childhood. *Ces Pediatr* 1979; **34**: 80–3.

29	Amla I, Kumari S, Murthy V, Jayaraj P, Parpia H. Role of aflatoxin in Indian childhood cirrhosis. (A preliminary communication). *Indian Pediatr* 1970; **7**: 262–70.

30	Ribarova FT, Yardanova P. Ochratoxin a - Risk factor for human health. *Acta Medica Bulg* 2003; **30**: 24–35.

31	Payet M. [Aflatoxins and cancer of the liver]. *Med Chir Dig* 1975; **4**: 57.

32	Butler WH. Liver injury induced by aflatoxin. *Prog Liver Dis* 1970; **3**: 408–18.

33	Naqvi SH. A flatoxin and liver disease. *J Pak Med Assoc* 1980; **30**: 23.

34	Asgaonkar D, Jagose J, Jaykar VV, Vanjani CV, Joshi VR. Ergot poisoning: a report of two cases. *J Assoc Physicians India* 1987; **35**: 603–5.

35	Huţanaşu C, Sfarti C, Trifan A, *et al.* [High blood and urine concentration of hepatotoxic mycotoxins (aflatoxin B1, sterigmatocystin) in patients with liver cirrhosis]. *Rev Med Chir Soc Med Nat Iasi* 2009; **113**: 59–63.

36	Nizami F, HM N, Ahmad M. Urinary excretion of aflatoxin and liver cancer in Karachi. *J Pak Med Assoc* 1986; **36**: 112–4.

37	Olubuyide I, Maxwell S, Hood H, Neal G, Hendrickse R. HBsAg, aflatoxins and primary hepatocellular carcinoma. *Afr J Med Med Sci* 1993; **22**: 89–91.

38	Abulu E, Uriah N, Aigbefo H, Oboh P, Agbonlahor D. Preliminary investigation on aflatoxin in cord blood of jaundiced neonates. *West Afr J Med* 1998; **17**: 184–7.

39	Dhatt PS, Parida NK, Das Chaudhury PK, Singh H. Aflatoxins and Indian childhood cirrhosis. *Indian Pediatr* 1982; **19**: 407–8.

40	Petkova-Bocharova T, IN C, Castegnaro M. Ochratoxin A in human blood in relation to Balkan endemic nephropathy and urinary system tumours in Bulgaria. *Food Addit Contam* 1988; **5**: 299–301.

41	Burguera JA. Presence of aflatoxin B1 in human liver referred to as Reye’s syndrome in Venezuela. *Acta Cient Venez* 1986; **37**: 325–6.

42	Andrássy K, Horváth I, Lakos T, Töke Z. [Outbreak of mass mycotoxicosis in Hajdú-Bihar County]. *Orv Hetil* 1979; **120**: 1763–4.

43	Le Breton E. [Hepatic cancer and mycotoxins]. *Ann Biol Clin* 1970; **28**: 203–8.

44	Breitholtz-Emanuelsson A, Minervini F, Hult K, Visconti A. Ochratoxin A in human serum samples collected in southern Italy from healthy individuals and individuals suffering from different kidney disorders. *Nat Toxins* 1994; **2**: 366–70.

45	Nguyen H. [Risk of liver cancer due to peanut butter?]. *Dtsch Med Wochenschr* 1999; **124**: 299.

46	Terao K. Mycotoxicosis caused by aflatoxin B1 and related compounds (Japanese). *CHIBA MEDJ* 1974; **50**: 159–65.

47	De Vries H, Lamplugh S. Aflatoxins in liver biopsies from Kenya. *Trop Geogr Med* 1989; **41**: 26–30.

48	Serck-Hanssen A. Aflatoxin-induced fatal hepatitis? A case report from Uganda. *Arch Env Heal* 1970; **20**: 729–31.

49	Krishnamachari KA, Bhat VR, Nagarajan V, Tilak TB, Tulpule PG. The problem of aflatoxic human disease in parts of India-epidemiological and ecological aspects. *Ann Nutr Aliment* 1977; **31**: 991–6.

50	Dvorácková I, Zilková J, Brodský F, Cerman J. Aflatoxin and liver damage with encephalopathy. *Sb Ved Pr Lek Fak Karlov Univerzity Hradci Kral* 1972; **15**: 521–4.

51	Dvorackova I, Brodsky F, Cerman J. Aflatoxin: possible factor of liver damage in children (Czech). *Cesk Hyg* 1974; **19**: 211–6.

52	Karvonen M. [Buerger’s disease and ergotism]. *Duodecim* 1951; **67**: 406–14.

53	Plestina R, Ceović S, Gatenbeck S, *et al.* Human exposure to ochratoxin A in areas of Yugoslavia with endemic nephropathy. *J Env Pathol Toxicol Oncol* 1990; **10**: 145–8.

54	Onyemelukwe C, Nirodi C, West C. Aflatoxin B1 in hepatocellular carcinoma. *Trop Geogr Med* 1980; **32**: 237–40.

55	Alvarez M, Castañeda C, Escobar A, Fragoso T. [Aflatoxin detection in urine in liver diseases in childhood]. *G E N* 1991; **45**: 205–9.

56	Aljicevic M, Hamzic S. Aflatoxin in the urine of patients suffering fom HCC and HBV. *HEALTHMED* 2010; **4**: 852–6.

57	Peers F, Linsell C. Dietary aflatoxins and human primary liver cancer. *Ann Nutr Aliment* 1977; **31**: 1005–17.

58	RV B, PH S, RP A, RV S. A foodborne disease outbreak due to the consumption of moldy sorghum and maize containing fumonisin mycotoxins. *J Toxicol Clin Toxicol* 1997; **35**: 249–55.

59	Dvorackova I, Kusak V. Hepatocellular carcinoma (a 28-year necropsy review). *J Env Pathol Toxicol Oncol* 1990; **10**: 220–4.

60	Shank RC, Bourgeois CH, Keschamras N, Chandavimol P. Aflatoxins in autopsy specimens from Thai children with an acute disease of unknown aetiology. *Food Cosmet Toxicol* 1971; **9**: 501–7.

61	Lye MS, Ghazali AA, Mohan J, Alwin N, Nair RC. An outbreak of acute hepatic encephalopathy due to severe aflatoxicosis in Malaysia. *Am J Trop Med Hyg* 1995; **53**: 68–72.

62	Wang ZG, Feng JN, Tong Z. Human toxicosis caused by moldy rice contaminated with fusarium and T-2 toxin. *Biomed Env Sci* 1993; **6**: 65–70.

63	Ruan F, Chen JG, Chen L, *et al.* Food Poisoning Caused by Deoxynivalenol at a School in Zhuhai, Guangdong, China, in 2019. *Foodborne Pathog Dis* 2020; **17**: 429–33.

64	Perduri R, Gobba S. Unexpected aflatoxin exposure in a woman in northern Italy: a case report. *Cases J* 2009; **2**: 7736.

65	Probst C, Njapau H, PJ C. Outbreak of an acute aflatoxicosis in Kenya in 2004: identification of the causal agent. *Appl Env Microbiol* 2007; **73**: 2762–4.

66	Krishnamachari KAVR, Nagarajan V, Bhat R, Tilak TBG. Hepatitis Due To Aflatoxicosis. *Lancet* 1975; **305**: 1061–3.

67	Hadidane R, Roger-Regnault C, H. B, *et al.* Correlation between alimentary mycotoxin contamination and specific diseases. *Hum Toxicol* 1985; **4**: 491–501.

68	Tzee‐Cheng C, Maxwell SM, Wong S ‐Y. An outbreak of aflatoxicosis and boric acid poisoning in Malaysia: A clinicopathological study. *J Pathol* 1991; **164**: 225–33.

69	Nyikal et al. Outbreak of Aflatoxin Poisoning --- Eastern and Central Provinces, Kenya, January--July 2004. *Morb Mortal Wkly Rep* 2004; **53**: 790–3.

70	Jolly PE, Jiang Y, Ellis WO, *et al.* Association between aflatoxin exposure and health characteristics, liver function, hepatitis and malaria infections in Ghanaians. *J Nutr Environ Med* 2007; **16**: 242–57.

71	Samuel N, Ezri Y, Farah R, *et al.* Acute Aflatoxicosis Resulting in Fulminant Hepatic Failure and Rhabdomyolysis. *Gastroenterol Res* 2009; **2**: 48–50.

72	Kamala A, Shirima C, Jani B, *et al.* Outbreak of an acute aflatoxicosis in Tanzania during 2016. *World Mycotoxin J* 2018; **11**: 311–20.

73	Koshiol J, Zhu B, Wang R, *et al.* Association of aflatoxin with gallbladder cancer in a case-control study nested within a Chinese cohort. *Int J Cancer* 2024; **154**: 801–6.

74	Yadav A, Gupta P, Gupta P, *et al.* Aflatoxin exposure is associated with an increased risk of gallbladder cancer. *Int J Cancer* 2025; **156**: 322–30.

75	Koshiol J, YT G, Dean M, *et al.* Association of Aflatoxin and Gallbladder Cancer. *Gastroenterology* 2017; **153**: 488-494.e1.

76	Nogueira L, Foerster C, Groopman J, Egner P, Koshiol J, Ferreccio C. Association of aflatoxin with gallbladder cancer in Chile. *JAMA - J Am Med Assoc* 2015; **313**: 2075–7.

77	Grosso F, Saíd S, Mabrouk I, *et al.* New data on the occurrence of ochratoxin A in human sera from patients affected or not by renal diseases in Tunisia. *Food Chem Toxicol* 2003; **41**: 1133–40.

78	Thé Y, Manda P, Elleingand E, *et al.* The role of ochratoxin A in the development of bladder tumorsin Ivorian patients. *Toxicol Anal Clin* 2015; **27**: 66–71.

79	Ozçelik N, Koşar A, Soysal D. Ochratoxin A in human serum samples collected in Isparta-Turkey from healthy individuals and individuals suffering from different urinary disorders. *Toxicol Lett* 2001; **121**: 9–13.

80	Aslam M, AE B, Blaszkewicz M, GH D, Golka K. Ochratoxin A blood concentration in healthy subjects and bladder cancer cases from Pakistan. *Mycotoxin Res* 2005; **21**: 164–7.

81	Aslam M, Rivzi S, Beg A, Blaszkewicz M, Golka K, Degen GH. Analysis of Ochratoxin a Blood Levels in Bladder Cancer Cases and Healthy Persons from Pakistan. *J Toxicol Environ Health* 2012; **75**: 1176–84.

82	Fahmy N, Woo M, Alameldin M, *et al.* Ochratoxin A is not detectable in renal and testicular tumours. *Can Urol Assoc J* 2014; **8**: 40–6.

83	Claeys L, S DS, Scelo G, *et al.* Mycotoxin Exposure and Renal Cell Carcinoma Risk: An Association Study in the EPIC European Cohort. *Nutrients* 2022; **14**: 3581.

84	Malir F, Louda M, Ostry V, *et al.* Analyses of biomarkers of exposure to nephrotoxic mycotoxins in a cohort of patients with renal tumours. *Mycotoxin Res* 2019; **35**: 391–403.

85	Oyelami OA, Maxwell SM, Adelusola KA, Aladekoma TA, Oyelese AO. Aflatoxins in autopsy kidney specimens from children in Nigeria. *J Toxicol Env Heal A* 1995; **55**: 317–23.

86	Wafa EW, Yahya RS, Sobh MA, *et al.* Human ochratoxicosis and nephropathy in Egypt: a preliminary study. *Hum Exp Toxicol* 1998; **17**: 124–9.

87	Yordanova P, Wilfried K, Tsolova S, Dimitrov P. Ochratoxin A and β2-microglobulin in BEN patients and controls. *Toxins (Basel)* 2010; **2**: 780–92.

88	Oyelami OA, Maxwell SM, Adelusola KA, Aladekoma TA, Oyelese AO. Aflatoxins in the autopsy brain tissue of children in Nigeria. *Mycopathologia* 1995; **132**: 35–8.

89	Kareem SA, Hameed RM, Al-Redha SA, Al-Khatt H. Aflatoxin b1 as a threshold concept of uncertain etiology of chronic kidney diseases. *Indian J Forensic Med Toxicol* 2021; **15**: 737–44.

90	Mohd Redzwan S, Rosita J, Mohd Sokhini AM, *et al.* Detection of serum AFB1-lysine adduct in Malaysia and its association with liver and kidney functions. *Int J Hyg Env Heal* 2014; **217**: 443–51.

91	Schwerdt G, Schuster C, Silbernagl S, Gareis M, Gekle M. Preliminary results on Ochratoxin A concentrations in blood of patients with various kidney diseases in Germany. *Mycotoxin Res* 2001; **17 Suppl 2**: 146–9.

92	Desalegn B, Nanayakkara S, KH H, *et al.* Mycotoxin detection in urine samples from patients with chronic kidney disease of uncertain etiology in Sri Lanka. *Bull Env Contam Toxicol* 2011; **87**: 6–10.

93	Lin H, Liu W, Zeng H, *et al.* Determination of Environmental Exposure to Microcystin and Aflatoxin as a Risk for Renal Function Based on 5493 Rural People in Southwest China. *Environ Sci Technol* 2016; **50**: 5346–56.

94	Al-Mhanaa HAH, Al-Obaidi AB. The evaluation of ochratoxin A in patients suffer from renal failure. *Indian J Forensic Med Toxicol* 2020; **14**: 1293–7.

95	Oyelami OA, Maxwell SM, Adelusola KA, Aladekoma TA, Oyelese AO. Aflatoxins in the lungs of children with kwashiorkor and children with miscellaneous diseases in Nigeria. *J Toxicol Env Heal* 1997; **51**: 623–8.

96	Kosicki R, Buharowska-Donten J, Twarużek M. Ochratoxin A levels in serum of Polish dialysis patients with chronic renal failure. *Toxicon* 2021; **200**: 183–8.

97	Hassen W, Abid S, Achour A, Creppy E, Bacha H. Ochratoxin A and β2-microglobulinuria in healthy individuals and in chronic interstitial nephropathy patients in the Centre of Tunisia: A hot spot of Ochratoxin A exposure. *Toxicology* 2004; **199**: 185–93.

98	Karamkhani M, Asilian-Mahabadi H, Daraei B, Seidkhani-Nahal A, Noori-Zadeh A. Liver and kidney serum profile abnormalities in workers exposed to aflatoxin B1 in urban solid waste management centers. *Env Monit Assess* 2020; **192**: 472.

99	Zaied C, Bouaziz C, Azizi I, *et al.* Presence of ochratoxin A in Tunisian blood nephropathy patients. Exposure level to OTA. *Exp Toxicol Pathol* 2011; **63**: 613–8.

100	Sangare-Tigori B, Moukha S, JH K, *et al.* Ochratoxin A in human blood in Abidjan, Côte d’Ivoire. *Toxicon* 2006; **47**: 894–900.

101	Abid S, Hassen W, Achour A, *et al.* Ochratoxin A and human chronic nephropathy in Tunisia: is the situation endemic? *Hum Exp Toxicol* 2003; **22**: 77–84.

102	Maaroufi K, Achour A, Hammami M, *et al.* Ochratoxin A in human blood in relation to nephropathy in Tunisia. *Hum Exp Toxicol* 1995; **14**: 609–14.

103	Hmaissia Khlifa K, Ghali R, Mezigh C, *et al.* Serum levels of ochratoxin A in healthy subjects and in nephropathic patients in Tunisia. *Ann Biol Clin* 2008; **66**: 631–6.

104	Hmaissia Khlifa K, Ghali R, Mazigh C, Aouni Z, Machgoul S, Hedhili A. Ochratoxin A levels in human serum and foods from nephropathy patients in Tunisia: where are you now? *Exp Toxicol Pathol* 2012; **64**: 509–12.

105	Dinis AM, Lino CM, Pena AS. Ochratoxin A in nephropathic patients from two cities of central zone in Portugal. *J Pharm Biomed Anal* 2007; **44**: 553–7.

106	Hsieh MF, Chiu HY, Lin-Tan D, Lin JL. Does human ochratoxin A aggravate proteinuria in patients with chronic renal disease? *Ren Fail* 2004; **26**: 311–6.

107	Díaz de León-Martínez L, Díaz-Barriga F, Barbier O, *et al.* Evaluation of emerging biomarkers of renal damage and exposure to aflatoxin-B 1 in Mexican indigenous women: a pilot study. *Environ Sci Pollut Res* 2019; **26**: 12205–16.

108	Maaroufi K, Achour A, AM B, *et al.* Foodstuffs and human blood contamination by the mycotoxin ochratoxin A: correlation with chronic interstitial nephropathy in Tunisia. *Arch Toxicol* 1995; **69**: 552–8.

109	Smpokou ET, González-Quiroz M, Martins C, *et al.* Environmental exposures in young adults with declining kidney function in a population at risk of Mesoamerican nephropathy. *Occup Env Med* 2019; **76**: 920–6.

110	Hassan AM, Sheashaa HA, Fattah M, Ibrahim A, Gaber O, Sobh M. Study of ochratoxin A as an environmental risk that causes renal injury in breast-fed Egyptian infants. *Pediatr Nephrol* 2006; **21**: 102–5.

111	Xu Y, Moore S, Chen G, *et al.* Impact of dietary aflatoxin on immune development in Gambian infants: a cohort study. *BMJ Open* 2021; **11**: e048688.

112	Wood L, Wood M, Fisher B, Jaspan H, Sodora D. T Cell Activation in South African HIV-Exposed Infants Correlates with Ochratoxin A Exposure. *Front Immunol* 2017; **8**: 1857.

113	Jiang Y, Jolly P, Ellis W, Wang J, Phillips T, Williams J. Aflatoxin B1 albumin adduct levels and cellular immune status in Ghanaians. *Int Immunol* 2005; **17**: 807–14.

114	Jolly P, Shuaib F, Jiang Y, *et al.* Association of high viral load and abnormal liver function with high aflatoxin B1-albumin adduct levels in HIV-positive Ghanaians: preliminary observations. *Food Addit Contam Part A Chem Anal Control Expo Risk Assess* 2011; **28**: 1224–34.

115	Turner P, Moore S, Hall A, Prentice A, Wild C. Modification of immune function through exposure to dietary aflatoxin in Gambian children. *Env Heal Perspect* 2003; **111**: 217–20.

116	Huybrechts I, Jacobs I, Biessy C, *et al.* Associations between dietary mycotoxins exposures and risk of hepatocellular carcinoma in a European cohort. *PLoS One* 2024; **19**: 1–18.

117	Stora C, Dvorackova I, Ayraud N. Characterization of aflatoxin B1 (AFB) in human liver cancer. *Res Commun Chem Pathol Pharmacol* 1981; **31**: 77–84.

118	El-Kafrawy S, Abdel-Hamid M, El-Daly M, *et al.* P53 mutations in hepatocellular carcinoma patients in Egypt. *Int J Hyg Env Heal* 2005; **208**: 263–70.

119	Huţanaşu C, Sfarti C, Trifan A, *et al.* [High levels of sterigmatocystin in patients with chronic liver diseases]. *Rev Med Chir Soc Med Nat Iasi* 2011; **115**: 33–7.

120	Onyemelukwe G, Ogbadu G, Salifu A. Aflatoxins, B1, B2, G1, G2 in primary liver cell carcinoma. *Toxicol Lett* 1982; **10**: 309–12.

121	Lunn R, Zhang Y, Wang L, *et al.* p53 mutations, chronic hepatitis B virus infection, and aflatoxin exposure in hepatocellular carcinoma in Taiwan. *Cancer Res* 1997; **57**: 3471–7.

122	Nogueira J, Ono-Nita S, Nita M, *et al.* 249 TP53 mutation has high prevalence and is correlated with larger and poorly differentiated HCC in Brazilian patients. *BMC Cancer* 2009; **9**: 204.

123	Abedi-Ardekani B, Gouas D, Villar S, Sotoudeh M, Hainaut P. TP53 Mutations and HBX Status Analysis in Hepatocellular Carcinomas from Iran: Evidence for Lack of Association between HBV Genotype D and TP53 R249S Mutations. *Hepat Res Treat* 2011; **2011**: 475965.

124	Jiang W, Lu Q, Pan G. [p53 gene mutation in hepatocellular carcinoma]. *Zhonghua Wai Ke Za Zhi* 1998; **36**: 531–2.

125	Zhou Y, Peng W, Yao J. [Synergism of three risk factors in hepatocellular carcinogenicity]. *Zhonghua Yu Fang Yi Xue Za Zhi* 1996; **30**: 289–91.

126	Yu M, Lien J, Chiu Y, Santella R, Liaw Y, Chen C. Effect of aflatoxin metabolism and DNA adduct formation on hepatocellular carcinoma among chronic hepatitis B carriers in Taiwan. *J Hepatol* 1997; **27**: 320–30.

127	Yu SZ. [The aflatoxins and liver cancer in Guangxi, China]. *Zhonghua Yu Fang Yi Xue Za Zhi* 1992; **26**: 162–4.

128	El-Shanawani F, Abdel-Hadi A, Abu Zikri N, Ismail A, El-Ansary M, El-Raai A. Clinical significance of aflatoxin, mutant P53 gene and sIL-2 receptor in liver cirrhosis and hepatocellular carcinoma. *J Egypt Soc Parasitol* 2006; **36**: 221–39.

129	de Carvalho FM, de Almeida Pereira T, Gonçalves PL, Jarske RD, Pereira FE, Louro ID. Hepatocellular carcinoma and liver cirrhosis TP53 mutation analysis reflects a moderate dietary exposure to aflatoxins in Espírito Santo State, Brazil. *Mol Biol Rep* 2013; **40**: 4883–7.

130	Kimbi GC, Kew MC, Yu MC, Arakawa K, Hodkinson J. 249ser p53 mutation in the serum of black southern African patients with hepatocellular carcinoma. *J Gastroenterol Hepatol* 2005; **20**: 1185–90.

131	Hollstein M, Wild C, Bleicher F, *et al.* p53 mutations and aflatoxin B1 exposure in hepatocellular carcinoma patients from Thailand. *Int J Cancer* 1993; **53**: 51–5.

132	Hoque A, YZ P, Yoffe B, *et al.* Does aflatoxin B1 play a role in the etiology of hepatocellular carcinoma in the United States? *Nutr Cancer* 1999; **35**: 27–33.

133	Bessard G, Soubeyrand J, Chauvier C, Pollet A. [Dietary contamination and hepatic presence of aflatoxins in an individual with primitive liver cancer (PLC) in Ivory Coast]. *J Toxicol Clin Exp* 1990; **10**: 41–4.

134	Buetow K, Sheffield V, Zhu M, *et al.* Low frequency of p53 mutations observed in a diverse collection of primary hepatocellular carcinomas. *Proc Natl Acad Sci U S A* 1992; **89**: 9622–6.

135	Shirabe K, Toshima T, Taketomi A, *et al.* Hepatic aflatoxin B1-DNA adducts and TP53 mutations in patients with hepatocellular carcinoma despite low exposure to aflatoxin B1 in southern Japan. *Liver Int* 2011; **31**: 1366–72.

136	Sun Z, Lu P, MH G, *et al.* Increased risk of hepatocellular carcinoma in male hepatitis B surface antigen carriers with chronic hepatitis who have detectable urinary aflatoxin metabolite M1. *Hepatology* 1999; **30**: 379–83.

137	Szymañska K, JG C, Cui Y, *et al.* TP53 R249S mutations, exposure to aflatoxin, and occurrence of hepatocellular carcinoma in a cohort of chronic hepatitis B virus carriers from Qidong, China. *Cancer Epidemiol Biomarkers Prev* 2009; **18**: 1638–43.

138	Lino-Silva L, Lajous M, Brochier M, *et al.* Aflatoxin levels and prevalence of TP53 aflatoxin-mutations in hepatocellular carcinomas in Mexico. *Salud Publica Mex* 2022; **64**: 35–40.

139	Manda P, Adépo AJ-B, Kouassi M’bengue A, *et al.* Assessment of the role of aflatoxin B1 in the development of hepatocellular carcinoma in Côte d’Ivoire (Ivory Coast): Preliminary study. *Toxicol Anal Clin* 2018; **30**: 85–95.

140	Yeh MM, Daniel HDJ, Torbenson M. Hepatitis C-associated hepatocellular carcinomas in non-cirrhotic livers. *Mod Pathol* 2010; **23**: 276–83.

141	Long X, Ma Y, Huang H, Yao J, Qu D, Lu Y. Polymorphism of XRCC1 and the frequency of mutation in codon 249 of the p53 gene in hepatocellular carcinoma among Guangxi population, China. *Mol Carcinog* 2008; **47**: 295–300.

142	Shimizu Y, JJ Z, Han F, Ishikawa T, Oda H. Different frequencies of p53 codon-249 hot-spot mutations in hepatocellular carcinomas in Jiang-su province of China. *Int J Cancer* 1999; **82**: 187–90.

143	Wu H, Shen J, Siegel A, Santella R. Environmental exposure and clinical correlates of hepatocellular carcinoma in New York City: a case only study. *Cancer Causes Control* 2022; **33**: 153–9.

144	Hosny G, Farahat N, Tayel H, Hainaut P. Ser-249 TP53 and CTNNB1 mutations in circulating free DNA of Egyptian patients with hepatocellular carcinoma versus chronic liver diseases. *Cancer Lett* 2008; **264**: 201–8.

145	Ross R, Yuan J, Yu M, *et al.* Urinary aflatoxin biomarkers and risk of hepatocellular carcinoma. *Lancet* 1992; **339**: 943–6.

146	Ibrahim A, Zaghloul H, Badria F. Case report evidence of relationships between hepatocellular carcinoma and ochratoxicosis. *PLoS One* 2013; **8**: e71423.

147	Bulatao-Jayme J, EM A, MC C, MT J, LA S. A case-control dietary study of primary liver cancer risk from aflatoxin exposure. *Int J Epidemiol* 1982; **11**: 112–9.

148	Ramalho L, Porta L, Rosim R, *et al.* Aflatoxin B(1) residues in human livers and their relationship with markers of hepatic carcinogenesis in São Paulo, Brazil. *Toxicol Rep* 2018; **5**: 777–84.

149	Wray B, Hayes A. Aflatoxin B1 in the serum of a patient with primary hepatic carcinoma. *Env Res* 1980; **22**: 400–3.

150	Olubuyide IO. The natural history of primary liver cell carcinoma - a study of 89 untreated adult nigerians. *Cent Afr J Med* 1992; **38**: 25–30.

151	Srivatanakul P, DM P, Khlat M, *et al.* Liver cancer in Thailand. II. A case-control study of hepatocellular carcinoma. *Int J Cancer* 1991; **48**: 329–32.

152	Chen C, Wang L, Lu S, *et al.* Elevated aflatoxin exposure and increased risk of hepatocellular carcinoma. *Hepatology* 1996; **24**: 38–42.

153	Rahman El-Zayadi A, Abaza H, Shawky S, Mohamed M, Selim O, Badran H. Prevalence and epidemiological features of hepatocellular carcinoma in Egypt-a single center experience. *Hepatol Res* 2001; **19**: 170–9.

154	Murugavel K, Naranatt P, Shankar E, *et al.* Prevalence of aflatoxin B1 in liver biopsies of proven hepatocellular carcinoma in India determined by an in-house immunoperoxidase test. *J Med Microbiol* 2007; **56**: 1455–9.

155	Asim M, MP S, Thayumanavan L, Kar P. Role of aflatoxin B1 as a risk for primary liver cancer in north Indian population. *Clin Biochem* 2011; **44**: 1235–40.

156	Qian G, Ross M, Yu M, *et al.* A follow-up study of urinary markers of aflatoxin exposure and liver cancer risk in Shanghai, People’s Republic of China. *Cancer Epidemiol Biomarkers Prev* 1994; **3**: 3–10.

157	Peers F, Linsell C. Dietary aflatoxins and liver cancer-a population based study in Kenya. *Br J Cancer* 1973; **27**: 473–84.

158	Kirk G, Lesi O, Mendy M, *et al.* 249(ser) TP53 mutation in plasma DNA, hepatitis B viral infection, and risk of hepatocellular carcinoma.1 Kirk G, Lesi O, Mendy M, et al. 249(ser) TP53 mutation in plasma DNA, hepatitis B viral infection, and risk of hepatocellular carcinoma. Oncogene 200. *Oncogene* 2005; **24**: 5858–67.

159	Ozdemir F, Tiftikci A, Sancak S, *et al.* The prevalence of the mutation in codon 249 of the P53 gene in patients with hepatocellular carcinoma (HCC) in Turkey. *J Gastrointest Cancer* 2010; **41**: 185–9.

160	McGlynn K, Rosvold E, Lustbader E, *et al.* Susceptibility to hepatocellular carcinoma is associated with genetic variation in the enzymatic detoxification of aflatoxin B1. *Proc Natl Acad Sci U S A* 1995; **92**: 2384–7.

161	Afum C, Cudjoe L, Hills J, *et al.* Association between aflatoxin M1 and liver disease in HBV/HCV infected persons in Ghana. *Int J Environ Res Public Health* 2016; **13**: 1–11.

162	Deng Z, Ma Y. Aflatoxin sufferer and p53 gene mutation in hepatocellular carcinoma. *World J Gastroenterol* 1998; **4**: 28–9.

163	Ng I, Srivastava G, Chung L, Tsang S, Ng M. Overexpression and point mutations of p53 tumor suppressor gene in hepatocellular carcinomas in Hong Kong Chinese people. *Cancer* 1994; **74**: 30–7.

164	Ozturk M. p53 mutation in hepatocellular carcinoma after aflatoxin exposure. *Lancet* 1991; **338**: 1356–9.

165	Wu H, Wang Q, Yang H, *et al.* Aflatoxin B1 exposure, hepatitis B virus infection, and hepatocellular carcinoma in Taiwan. *Cancer Epidemiol Biomarkers Prev* 2009; **18**: 846–53.

166	Zeng H, Lin H, Liu W, *et al.* Prevalence of metabolic syndrome among adults with liver function injury in rural area of Southwest China: A cross-sectional study. *Sci Rep* 2017; **7**: 5518.

167	Peers F, Bosch X, Kaldor J, Linsell A, Pluijmen M. Aflatoxin exposure, hepatitis B virus infection and liver cancer in Swaziland. *Int J Cancer* 1987; **39**: 545–53.

168	Lu P, Kuang S, Wang J. [Hepatitis B virus infection and aflatoxin exposure in the development of primary liver cancer]. *Zhonghua Yi Xue Za Zhi* 1998; **78**: 340–2.

169	Tian H, Lou J, Du C. [Determination of sterigmatocystin in cancerous tissues, blood and urine in patients with liver and stomach cancer]. *Zhonghua Yu Fang Yi Xue Za Zhi* 1995; **29**: 276–8.

170	Chan V, Fonseca J, Sittenfeld A, *et al.* Content and human consumption of aflatoxins and hepatocellolar cancer in Costa Rica. *Rev Med Costa Rica* 1990; **47**: 25–9.

171	Mizrak D, Engin B, FO O, *et al.* Aflatoxin exposure in viral hepatitis patients in Turkey. *Turk J Gastroenterol* 2009; **20**: 192–7.

172	Wild C, Fortuin M, Donato F, *et al.* Aflatoxin, liver enzymes, and hepatitis B virus infection in Gambian children. *Cancer Epidemiol Biomarkers Prev* 1993; **2**: 555–61.

173	Persson E, Sewram V, Evans A, *et al.* Fumonisin B1 and risk of hepatocellular carcinoma in two Chinese cohorts. *Food Chem Toxicol* 2012; **50**: 679–83.

174	Lu P, Wang J, Zhang Q, Wu Y, Sun Y, Chen T. Longitudinal study of aflatoxin exposure in the development of primary liver cancer in patients with chronic hepatitis. *Zhonghua Yi Xue Za Zhi* 2010; **90**: 1665–9.

175	Liu Z, Li L, Peng M, *et al.* Hepatitis B virus infection contributes to oxidative stress in a population exposed to aflatoxin B1 and high-risk for hepatocellular carcinoma. *Cancer Lett* 2008; **263**: 212–22.

176	Aydin M, Aydin S, Bacanli M, Basaran N. Aflatoxin levels in chronic hepatitis B patients with cirrhosis or hepatocellular carcinoma in Balkesir, Turkey. *J Viral Hepat* 2015; **22**: 926–35.

177	Sun CA, Wang LY, Chen CJ, *et al.* Genetic polymorphisms of glutathione S-transferases M1 and TI associated with susceptibility to aflatoxin-related hepatocarcinogenesis among chronic hepatitis B carriers: a nested case-control study in Taiwan. *Carcinogenesis* 2001; **22**: 1289–94.

178	Wu H, Wang Q, Wang L, *et al.* Polycyclic aromatic hydrocarbon- and aflatoxin-albumin adducts, hepatitis B virus infection and hepatocellular carcinoma in Taiwan. *Cancer Lett* 2007; **252**: 104–14.

179	Stora C, Dvorackova I. Aflatoxin, viral hepatitis and primary liver cancer. *J Med* 1987; **18**: 23–41.

180	Chu Y, Yang H, Wu H, *et al.* Aflatoxin B(1) exposure increases the risk of hepatocellular carcinoma associated with hepatitis C virus infection or alcohol consumption. *Eur J Cancer* 2018; **94**: 37–46.

181	Tan C, Lo D, Law N, Ng H, Chao T. Blood aflatoxin levels in patients with hepatocellular carcinoma in Singapore. *Singapore Med J* 1995; **36**: 612–4.

182	Ilić M, SS P, Prvulović-Bunović N, *et al.* Hepatocellular carcinoma and impact of aflatoxin difuranocoumarin derivative system – A case report. *Srp Arh Celok Lek* 2016; **144**: 661–3.

183	El-Shahat E-SA, Swelim MA, Mohamed AF, Abdel-Wahhab MA. Correlation study between aflatoxin M1 and hepatitis C virus in Egyptian patients with chronic liver disease. *World J Med Sci* 2012; **7**: 224–31.

184	Campbell TC, Chen JS, Liu CB, Li JI, Parpia B. Nonassociation of aflatoxin with primary liver cancer in a cross-sectional ecological survey in the People’s Republic of China. *Cancer Res* 1990; **50**: 6882–93.

185	Lleonart ME, Kirk GD, Villar S, *et al.* Quantitative analysis of plasma TP53 249Ser-mutated DNA by electrospray ionization mass spectrometry. *Cancer Epidemiol Biomarkers Prev* 2005; **14**: 2956–62.

186	Tchana AN, Moundipa PF, Tchouanguep FM. Aflatoxin contamination in food and body fluids in relation to malnutrition and cancer status in Cameroon. *Int J Env Res Public Heal* 2010; **7**: 178–88.

187	Tomerak RH, Shaban HH, Khalafallah OA, El Shazly MN. Assessment of exposure of Egyptian infants to aflatoxin M1 through breast milk. *J Egypt Public Heal Assoc* 2011; **86**: 51–5.

188	Ortiz-Cuaran S, Villar S, Gouas D, *et al.* Association between HBX status, aflatoxin-induced R249S TP53 mutation and risk of hepatocellular carcinoma in a case-control study from Thailand. *Cancer Lett* 2013; **331**: 46–51.

189	Soini Y, SC C, WP B, *et al.* An aflatoxin-associated mutational hotspot at codon 249 in the p53 tumor suppressor gene occurs in hepatocellular carcinomas from Mexico. *Carcinogenesis* 1996; **17**: 1007–12.

190	Mokhles M, MA AEW, Tawfik M, Ezzat W, Gamil K, Ibrahim M. Detection of aflatoxin among hepatocellular carcinoma patients in Egypt. *Pak J Biol Sci* 2007; **10**: 1422–9.

191	Kirk GD, Camus-Randon AM, Mendy M, *et al.* Ser-249 p53 mutations in plasma DNA of patients with hepatocellular carcinoma from The Gambia. *J Natl Cancer Inst* 2000; **92**: 148–53.

192	Chu Y, Yang H, Wu H, *et al.* Aflatoxin B(1) exposure increases the risk of cirrhosis and hepatocellular carcinoma in chronic hepatitis B virus carriers. *Int J Cancer* 2017; **141**: 711–20.

193	Mandishona E, AP M, VR G, *et al.* Dietary iron overload as a risk factor for hepatocellular carcinoma in Black Africans. *Hepatology* 1998; **27**: 1563–6.

194	Ngindu A, BK J, PR K, *et al.* Outbreak of acute hepatitis caused by aflatoxin poisoning in Kenya. *Lancet* 1982; **1**: 1346–8.

195	López C, Ramos L, Bulacio L, Ramadán S, Rodríguez F. Aflatoxin B1 content in patients with hepatic diseases. *Med (B Aires)* 2002; **62**: 313–6.

196	Ahmed H, RG H, SM M, AM Y. Neonatal jaundice with reference to aflatoxins: an aetiological study in Zaria, northern Nigeria. *Ann Trop Paediatr* 1995; **15**: 11–20.

197	Raafat N, WA E, AF G, OE N, Zakaria M. Assessment of serum aflatoxin B(1) levels in neonatal jaundice with glucose-6-phosphate dehydrogenase deficiency: a preliminary study. *Mycotoxin Res* 2021; **37**: 109–16.

198	Sodeinde O, MC C, SM M, JB F, RG H. Neonatal jaundice, aflatoxins and naphthols: report of a study in Ibadan, Nigeria. *Ann Trop Paediatr* 1995; **15**: 107–13.

199	Parkin D, Srivatanakul P, Khlat M, *et al.* Liver cancer in Thailand - a case-control study of cholangiocarcinoma. *Int J Cancer* 1991; **48**: 323–8.

200	Coulter JB, Suliman GI, Lamplugh SM, Mukhtar BI, Hendrickse RG. Aflatoxins in liver biopsies from Sudanese children. *Am J Trop Med Hyg* 1986; **35**: 360–5.

201	Abdel-Wahab M, Mostafa M, Sabry M, el-Farrash M, Yousef T. Aflatoxins as a risk factor for hepatocellular carcinoma in Egypt, Mansoura Gastroenterology Center study. *Hepatogastroenterology* 2008; **55**: 1754–9.

202	Ankrah NA, Rikimaru T, Ekuban FA. Observations on aflatoxins and the liver status of ghanaian subjects. *East Afr Med J* 1994; **71**: 739–41.

203	Nasir U, Naeem I, Asif M, *et al.* Assessment of aflatoxins exposure through urinary biomarker approach and the evaluation of the impacts of aflatoxins exposure on the selected health parameters of the children of Multan city of Pakistan. *FOOD Control* 2021; **123**. DOI:10.1016/j.foodcont.2021.107863.

204	Farag RMMA, AlAyobi D, Kwon HJ, EL-Ansary A. Relationship between aflatoxin B1 exposure and etiology of liver disease in Saudi Arabian patients. *J Pure Appl Microbiol* 2018; **12**: 1147–53.

205	Liu W, Wang L, Yang X, *et al.* Environmental Microcystin Exposure Increases Liver Injury Risk Induced by Hepatitis B Virus Combined with Aflatoxin: A Cross-Sectional Study in Southwest China. *Env Sci Technol* 2017; **51**: 6367–78.

206	Amla I, Parpia H, Jayaraj A. Cirrhosis in children after consumption of aflatoxin-contaminated peanut meal. *J Pathol* 1971; **103**: Pxix.

207	Alvarez C, Hernández E, Escobar K, *et al.* Aflatoxin B(1) exposure and liver cirrhosis in Guatemala: a case-control study. *BMJ Open Gastroenterol* 2020; **7**. DOI:10.1136/bmjgast-2020-000380.

208	Chen CH, Wang M, Wang JH, *et al.* Aflatoxin exposure and hepatitis C virus in advanced liver disease in a hepatitis C virus endemic area in Taiwan. *Am J Trop Med Hyg* 2007; **77**: 747–52.

209	Yadgiri B, Reddy V, PG T, SG S, Gopalan C. Aflatoxin and Indian childhood cirrhosis. *Am J Clin Nutr* 1970; **23**: 94–8.

210	Anitha S, Raghunadharao D, Waliyar F, *et al.* The association between exposure to aflatoxin, mutation in TP53, infection with hepatitis B virus, and occurrence of liver disease in a selected population in Hyderabad, India. *Mutat Res Genet Toxicol Env Mutagen* 2014; **766**: 23–8.

211	YY G, Wilson S, JK M, *et al.* Aflatoxin exposure may contribute to chronic hepatomegaly in Kenyan school children. *Env Heal Perspect* 2012; **120**: 893–6.

212	Sayed HA, El Ayyat A, El Dusoki H, *et al.* A cross sectional study of hepatitis B, C, some trace elements, heavy metals, aflatoxin B1 and schistosomiasis in a rural population, Egypt. *J Egypt Public Heal Assoc* 2005; **80**: 355–88.

213	De Santis B, Raggi M, Moretti G, *et al.* Study on the Association among Mycotoxins and other Variables in Children with Autism. *Toxins (Basel)* 2017; **9**. DOI:10.3390/toxins9070203.

214	Duringer J, Fombonne E, Craig M. No Association between Mycotoxin Exposure and Autism: A Pilot Case-Control Study in School-Aged Children. *Toxins (Basel)* 2016; **8**. NS -.

215	León F, Carpintero M, Bayona J, Gaffga N, Ocampo L, Del Castillo N. Lack of air conditioning and mycotoxin poisoning are basic co-factors in tropical spastic paraparesis. *Rev Neurol* 1998; **26**: 564–7.

216	Mahfuz M, MS H, MA A, *et al.* Chronic Aflatoxin Exposure and Cognitive and Language Development in Young Children of Bangladesh: A Longitudinal Study. *Toxins (Basel)* 2022; **14**. DOI:10.3390/toxins14120855.

217	Simon T, Rea W. Use of functional brain imaging in the evaluation of exposure to mycotoxins and toxins encountered in Desert Storm/Desert Shield. *Arch Env Heal* 2003; **58**: 406–9.

218	Wu T, Khorramshahi T, Taylor L, Bansal N, Rodriguez B, Rey I. Prevalence of Aspergillus-Derived Mycotoxins (Ochratoxin, Aflatoxin, and Gliotoxin) and Their Distribution in the Urinalysis of ME/CFS Patients. *Int J Env Res Public Heal* 2022; **19**. DOI:10.3390/ijerph19042052.

219	Brewer J, Thrasher J, Straus D, Madison R, Hooper D. Detection of mycotoxins in patients with chronic fatigue syndrome. *Toxins (Basel)* 2013; **5**: 605–17.

220	Arce-López B, Alvarez-Erviti L, B DS, *et al.* Biomonitoring of Mycotoxins in Plasma of Patients with Alzheimer’s and Parkinson’s Disease. *Toxins (Basel)* 2021; **13**. DOI:10.3390/toxins13070477.

221	Duringer J, Mazumder R, Palmer V, AM C, Spencer P. Case-Control Study of Nodding Syndrome in Acholiland: Urinary Multi-Mycotoxin Screening. *Toxins (Basel)* 2021; **13**. DOI:10.3390/toxins13050313.

222	Stora C, Dvorackova I, Ayraud N. Aflatoxin and Reye’s syndrome. *J Med* 1983; **14**: 47–54.

223	Nelson D, Kimbrough R, Landrigan P, Hayes A, Yang G, Benanides J. Aflatoxin and Reye’s syndrome: a case control study. *Pediatrics* 1980; **66**: 865–9.

224	Berry C. Correlation between alimentary mycotoxin contamination and specific diseases. *Hum Toxicol* 1986; **5**: 213–5.

225	Rogan W, Yang G, Kimbrough R. Aflatoxin and Reye’s syndrome: a study of livers from deceased cases. *Arch Env Heal* 1985; **40**: 91–5.

226	Becroft D, Webster D. Aflatoxins and Reye’s disease. *Br Med J* 1972; **4**: 117.

227	Lefèvre-Arbogast S, Duquenne P, Helmer C, Auriacombe S, Sirot V, Samieri C. Association between dietary exposure to chemical contaminants and risk of dementia in older persons. *Environ Int* 2024; **192**. DOI:10.1016/j.envint.2024.109033.

228	Akash MSH, Haq ME ul, Qader A, Rehman K. Biochemical investigation of human exposure to aflatoxin M1 and its association with risk factors of diabetes mellitus. *Environ Sci Pollut Res* 2021; **28**: 62907–18.

229	Alvarez CS, Rivera-Andrade A, Kroker-Lobos MF, *et al.* Associations between aflatoxin B1-albumin adduct levels with metabolic conditions in Guatemala: A cross-sectional study. *Heal Sci Reports* 2022; **5**: 1–10.

230	Xie G, Liu M, Xia C, Li R. Characterization of cord blood interleukin 10 on aflatoxinB1-exposed patients with gestational diabetes. *Clin Chim Acta* 2018; **487**: 46–7.

231	El-Azab SM, Abdelhamid AM, Shalaby HA, Mehrim AI, Ibrahim AH. Study of aflatoxin B1 as a risk factor that impairs the reproductive performance in females - Egypt. *Toxicol Environ Chem* 2010; **92**: 383–9.

232	Asci A, Durmaz E, Erkekoglu P, Pasli D, Bircan I, Kocer-Gumusel B. Urinary zearalenone levels in girls with premature thelarche and idiopathic central precocious puberty. *Minerva Pediatr* 2014; **66**: 571–8.

233	Massart F, Meucci V, Saggese G, Soldani G. High Growth Rate of Girls with Precocious Puberty Exposed to Estrogenic Mycotoxins. *J Pediatr* 2008; **152**. DOI:10.1016/j.jpeds.2007.10.020.

234	Deng F, FB T, DY L, *et al.* Effects of growth environments and two environmental endocrine disruptors on children with idiopathic precocious puberty. *Eur J Endocrinol* 2012; **166**: 803–9.

235	Rivera-Núñez Z, Barrett ES, Szamreta EA, *et al.* Urinary mycoestrogens and age and height at menarche in New Jersey girls. *Environ Heal A Glob Access Sci Source* 2019; **18**. DOI:10.1186/S12940-019-0464-8.

236	Gajecki M, Przybyłowicz M, Zielonka L, *et al.* Preliminary results of monitoring research on zearalenone presence in blood of women with neoplastic lesions in reproductive system. *Pol J Vet Sci* 2004; **7**: 153–6.

237	Kuciel-Lisieska G, Obremski K, Stelmachow J, *et al.* Presence of zearalenone in blood plasma in women with neoplastic lesions in the mammary gland. *Bull Vet Inst pulawyBULLETIN Vet Inst PULAWY* 2008; **52**: 671–4.

238	Pillay D, Chuturgoon AA, Nevines E, Manickum T, Deppe W, Dutton MF. The Quantitative Analysis of Zearalenone and Its Derivatives in Plasma of Patients with Breast and Cervical Cancer. *Clin Chem Lab Med* 2002; **40**: 946–51.

239	Belhassen H, Jiménez-Díaz I, Arrebola JP, *et al.* Zearalenone and its metabolites in urine from Tunisian women with breast cancer. A case-control study. *Clin Chem Lab Med* 2014; **52**: S792.

240	Unicsovics M, Molnár Z, Mézes M, *et al.* The Possible Role of Mycotoxins in the Pathogenesis of Endometrial Cancer. *Toxins (Basel)* 2024; **16**: 1–15.

241	Gadzała-Kopciuch R, Cendrowski K, Cesarz A, Kiełbasa P, Buszewski B. Determination of zearalenone and its metabolites in endometrial cancer by coupled separation techniques. *Anal Bioanal Chem* 2011; **401**: 2069–78.

242	Fry S, Kaye M, Missan D, *et al.* Microbial community profiling by next-generation DNA sequencing of adenocarcinoma of the prostate with evidence of ochratoxin A producing fungi. *Exp Mol Pathol* 2022; **128**: 104831.

243	Ibeh IN, Uraih N, Ogonar JI. Dietary exposure to aflatoxin in human male infertility in Benin City, Nigeria. *Int J Fertil Menopausal Stud* 1994; **39**: 208–14.

244	Ning X, Wang L, Wang JS, *et al.* High-Coverage UHPLC-MS/MS Analysis of 67 Mycotoxins in Plasma for Male Infertility Exposure Studies. *Toxics* 2024; **12**. DOI:10.3390/toxics12060395.

245	Yang C, Ning X, Wang B, *et al.* Association between spectrum of mycotoxins and semen quality : A cross-sectional study in Beijing , China. 2024; **476**. DOI:10.1016/j.jhazmat.2024.135124.

246	Okonofua FE, Ntoimo LFC, Unuabonah EI, *et al.* Association of Urinary Mycotoxins with Sperm Quality: A Case-Control Study in Southern Nigeria. *Toxins (Basel)* 2024; **16**: 1–15.

247	Ning Y, Chen S, Zhang F, *et al.* The alteration of urinary metabolomics profiles in Kashin-Beck disease in a three consecutive year study. *Mol Omi* 2023; **19**: 137–49.

248	Bhowate R, Lohe V, Meshram M, Dangore S. Serum aflatoxin B1 antibody titer, percent hemolysis and transaminases in oral submucous fibrosis. *J Oral Maxillofac Pathol* 2021; **25**: 110–7.

249	Lin S, Gao P, Li Q, *et al.* Aflatoxin influences achalasia symptomatology. *Mol Med Rep* 2020; **21**: 1276–84.

250	Niknejad F, Escrivá L, KB AR, Khoshnia M, FJ B, Berrada H. Biomonitoring of Multiple Mycotoxins in Urine by GC-MS/MS: A Pilot Study on Patients with Esophageal Cancer in Golestan Province, Northeastern Iran. *Toxins (Basel)* 2021; **13**. DOI:10.3390/toxins13040243.

251	Mulisa G, Pero-Gascon R, McCormack V, *et al.* Multiple mycotoxin exposure assessment through human biomonitoring in an esophageal cancer case-control study in the Arsi-Bale districts of Oromia region of Ethiopia. *Int J Hyg Environ Health* 2025; **263**. DOI:10.1016/j.ijheh.2024.114466.

252	Xue K, Tang L, Sun G, Wang S, Hu X, Wang J. Mycotoxin exposure is associated with increased risk of esophageal squamous cell carcinoma in Huaian area, China. *BMC Cancer* 2019; **19**: 1218.

253	Wang S, Pan D, Zhang T, *et al.* Corn Flour Intake, Aflatoxin B(1) Exposure, and Risk of Esophageal Precancerous Lesions in a High-Risk Area of Huai’an, China: A Case-Control Study. *Toxins (Basel)* 2020; **12**. DOI:10.3390/toxins12050299.

254	Liu Z, Zhang X, Zhao Q, *et al.* Whole-Exome Sequencing Among Chinese Patients With Hereditary Diffuse Gastric Cancer. *JAMA Netw Open* 2022; **5**: e2245836.

255	Eom S, Yim D, Zhang Y, *et al.* Dietary aflatoxin B1 intake, genetic polymorphisms of CYP1A2, CYP2E1, EPHX1, GSTM1, and GSTT1, and gastric cancer risk in Korean. *Cancer Causes Control* 2013; **24**: 1963–72.

256	Cirlini M, Mazzeo T, Roncoroni L, *et al.* Are Treated Celiac Patients at Risk for Mycotoxins? An Italian Case-Study. *Toxins (Basel)* 2016; **9**. NS -.

257	Brera C, Valitutti F, Nigri A, *et al.* Assessment of mycotoxins in human milk of celiac mothers: A preliminary outline. *Dig Liver Dis* 2013; **45**: e305.

258	Roy RN, Russell RI. Crohn’s disease and aflatoxins. *J R Soc Health* 1992; **112**: 277–9.

259	Ouhibi S, Vidal A, Martins C, *et al.* LC-MS/MS methodology for simultaneous determination of patulin and citrinin in urine and plasma applied to a pilot study in colorectal cancer patients. *Food Chem Toxicol* 2020; **136**: 110994.

260	Lisieska-Żołnierczyk S, Gajęcka M, Zielonka Ł, Dąbrowski M, Gajęcki MT. Blood levels of zearalenone, thyroid-stimulating hormone, and thyroid hormones in patients with colorectal cancer. *Toxicon* 2024; **251**. DOI:10.1016/j.toxicon.2024.108125.

261	Chen C, Patil CL, Mduma ER, Groopman JD, Riley RT, Wu F. Mycotoxins were not associated with environmental enteropathy in a cohort of Tanzanian children. *RISK Anal* 2023; **43**: 860–6.

262	Ayeni KI, Seki D, Pjevac P, *et al.* Biomonitoring of Dietary Mycotoxin Exposure and Associated Impact on the Gut Microbiome in Nigerian Infants. *Environ Sci Technol* 2024; **58**: 2236–46.

263	Oesterle I, Ayeni KI, Ezekiel CN, Berry D, Rompel A, Warth B. Insights into the early-life chemical exposome of Nigerian infants and potential correlations with the developing gut microbiome. *Environ Int* 2024; **188**: 108766.

264	Ashraf W, Rehman A, Ahmad M ud D, *et al.* Assessment of aflatoxin B1-lysine adduct in children and its effect on child growth in Lahore, Pakistan. *Food Addit Contam - Part A* 2022; **39**: 1463–73.

265	Alamu E, Gondwe T, Akello J, Maziya-Dixon B, Mukanga M. Relationship between serum aflatoxin concentrations and the nutritional status of children aged 6-24 months from Zambia. *Int J Food Sci Nutr* 2020; **71**: 593–603.

266	Bastos-moreira Y, Argaw A, Palma G Di, *et al.* Ochratoxin A Status at Birth Is Associated with Reduced Birth Weight and Ponderal Index in Rural Burkina Faso. 2025; **155**: 260–9.

267	Magoha H, Kimanya M, De Meulenaer B, Roberfroid D, Lachat C, Kolsteren P. Association between aflatoxin M exposure through breast milk and growth impairment in infants from northern Tanzania. *World Mycotoxin J* 2014; **7**: 277–84.

268	Matchado A, Smith JW, Schulze KJ, *et al.* Child Aflatoxin Exposure is Associated with Poor Child Growth Outcomes: A Prospective Cohort Study in Rural Malawi. *Curr Dev Nutr* 2023; **7**. DOI:10.1016/j.cdnut.2023.101962.

269	Andrews-Trevino J, Webb P, Shrestha R, *et al.* Exposure to multiple mycotoxins, environmental enteric dysfunction and child growth: Results from the AflaCohort Study in Banke, Nepal. *Matern Child Nutr* 2022; **18**: e13315.

270	Gong Y, Cardwell K, Hounsa A, *et al.* Dietary aflatoxin exposure and impaired growth in young children from Benin and Togo: cross sectional study. *BMJ* 2002; **325**: 20–1.

271	Mahdavi R, Nikniaz L, Arefhosseini S, Vahed Jabbari M. Determination of aflatoxin M(1) in breast milk samples in Tabriz-Iran. *Matern Child Heal J* 2010; **14**: 141–5.

272	Shouman B, El Morsi D, Shabaan S, Abdel-Hamid A, Mehrim A. Aflatoxin B1 level in relation to child’s feeding and growth. *Indian J Pediatr* 2012; **79**: 56–61.

273	Turner P, Collinson A, Cheung Y, *et al.* Aflatoxin exposure in utero causes growth faltering in Gambian infants. *Int J Epidemiol* 2007; **36**: 1119–25.

274	Ramjee G, Berjak P, Adhikari M, Dutton MF. Aflatoxins and kwashiorkor in Durban, South-Africa. *Ann Trop Paediatr* 1992; **12**: 241–7.

275	Chen C, NJ M, Gratz J, *et al.* Exposure to aflatoxin and fumonisin in children at risk for growth impairment in rural Tanzania. *Env Int* 2018; **115**: 29–37.

276	Lauer J, Natamba B, Ghosh S, Webb P, Wang J, Griffiths J. Aflatoxin exposure in pregnant women of mixed status of human immunodeficiency virus infection and rate of gestational weight gain: a Ugandan cohort study. *Trop Med Int Heal* 2020; **25**: 1145–54.

277	Njumbe Ediage E, Diana Di Mavungu J, Song S, Sioen I, De Saeger S. Multimycotoxin analysis in urines to assess infant exposure: a case study in Cameroon. *Env Int* 2013; **57**–**58**: 50–9.

278	Nabwire Wangia-Dixon R, Xue KS, Alcala J, *et al.* Nutrition and growth outcomes are affected by aflatoxin exposures in Kenyan children. *Food Addit Contam Part A Chem Anal Control Expo Risk Assess* 2020; **37**: 2123–34.

279	Watson S, SE M, MK D, *et al.* Impaired growth in rural Gambian infants exposed to aflatoxin: a prospective cohort study. *BMC Public Health* 2018; **18**: 1247.

280	Andrews-Trevino JY, Webb P, Shively G, *et al.* Relatively Low Maternal Aflatoxin Exposure Is Associated with Small-for-Gestational-Age but Not with Other Birth Outcomes in a Prospective Birth Cohort Study of Nepalese Infants. *J Nutr* 2019; **149**: 1818–25.

281	Lauer JM, Duggan CP, Ausman LM, *et al.* Maternal aflatoxin exposure during pregnancy and adverse birth outcomes in Uganda. *Matern Child Nutr* 2019; **15**: e12701.

282	Gichohi-Wainaina WN, Kimanya M, Muzanila YC, *et al.* Aflatoxin Contamination, Exposure among Rural Smallholder Farming Tanzanian Mothers and Associations with Growth among Their Children. *Toxins (Basel)* 2023; **15**. DOI:10.3390/toxins15040257.

283	Leroy JL, Sununtnasuk C, García-Guerra A, Wang JS. Low level aflatoxin exposure associated with greater linear growth in southern Mexico: A longitudinal study. *Matern Child Nutr* 2018; **14**: e12619.

284	Shirima CP, Kimanya ME, Routledge MN, *et al.* A prospective study of growth and biomarkers of exposure to aflatoxin and fumonisin during early childhood in Tanzania. *Env Heal Perspect* 2015; **123**: 173–8.

285	Mahfuz M, SMT H, MA A, *et al.* Aflatoxin exposure was not associated with childhood stunting: results from a birth cohort study in a resource-poor setting of Dhaka, Bangladesh. *Public Heal Nutr* 2021; **24**: 3361–70.

286	Huong BTM, Tuyen LD, Madsen H, Brimer L, Friis H, Dalsgaard A. Total Dietary Intake and Health Risks Associated with Exposure to Aflatoxin B(1), Ochratoxin A and Fuminisins of Children in Lao Cai Province, Vietnam. *Toxins (Basel)* 2019; **11**. DOI:10.3390/toxins11110638.

287	Voth-Gaeddert L, Stoker M, Torres O, Oerther DB. Association of aflatoxin exposure and height-for-age among young children in Guatemala. *Int J Env Heal Res* 2018; **28**: 280–92.

288	Gong Y, Hounsa A, Egal S, *et al.* Postweaning exposure to aflatoxin results in impaired child growth: A longitudinal study in Benin, West Africa. *Environ Health Perspect* 2004; **112**: 1334–8.

289	Mitchell NJ, Hsu HH, Chandyo RK, *et al.* Aflatoxin exposure during the first 36 months of life was not associated with impaired growth in Nepalese children: An extension of the MAL-ED study. *PLoS One* 2017; **12**: e0172124.

290	Memiş EY, Yalçın SS, Yalçın S. Mycotoxin carry-over in breast milk and weight of infant in exclusively-breastfed infants. *Arch Environ Occup Heal* 2020. DOI:10.1080/19338244.2020.1828242.

291	Tessema M, H DG, ID B, *et al.* Exposure to aflatoxins and fumonisins and linear growth of children in rural Ethiopia: a longitudinal study. *Public Heal Nutr* 2021; **24**: 3662–73.

292	ME K, B DM, Roberfroid D, Lachat C, Kolsteren P. Fumonisin exposure through maize in complementary foods is inversely associated with linear growth of infants in Tanzania. *Mol Nutr Food Res* 2010; **54**: 1659–67.

293	Andrews-Trevino JY, Webb P, Shively G, *et al.* Aflatoxin exposure and child nutrition: Measuring anthropometric and long-bone growth over time in Nepal. *Am J Clin Nutr* 2021; **113**: 874–83.

294	Hoffmann V, Jones K, JL L. The impact of reducing dietary aflatoxin exposure on child linear growth: a cluster randomised controlled trial in Kenya. *BMJ Glob Heal* 2018; **3**: e000983.

295	Tesfamariam K, Argaw A, Hanley-Cook GT, *et al.* Multiple mycotoxin exposure during pregnancy and risks of adverse birth outcomes: a prospective cohort study in rural Ethiopia. *Environ Int* 2022; **160**: 107052.

296	FM S, PE J, JE E, *et al.* Association between birth outcomes and aflatoxin B1 biomarker blood levels in pregnant women in Kumasi, Ghana. *Trop Med Int Heal* 2010; **15**: 160–7.

297	Kinkade CW, Núñez ZR, Brinker A, *et al.* Urinary mycoestrogens and gestational weight gain in the UPSIDE pregnancy cohort. *Environ Heal* 2024. DOI:10.1186/s12940-024-01141-8.

298	Osoro E, Awuor AO, Inwani I, *et al.* Association between low maternal serum aflatoxin B1 exposure and adverse pregnancy outcomes in Mombasa , Kenya , 2017 – 2019 : A nested matched case – control study. 2024; : 1–10.

299	Tan T, Chen T, Zhu W, *et al.* Adverse associations between maternal deoxynivalenol exposure and birth outcomes : a prospective cohort study in China. *BMC Med* 2023; : 1–11.

300	Zhong Y, Lu H, Lu X, *et al.* Correlations of Aflatoxin Exposure from Cooking Oil and Dietary Foods During Pregnancy with Birth Weight and Gestational Age at Birth in Guangxi , China. 2024; : 1171–84.

301	Abdulrazzaq YM, Osman N, Yousif ZM, Trad O. Morbidity in neonates of mothers who have ingested aflatoxins. *Ann Trop Paediatr* 2004; **24**: 145–51.

302	Abdulrazzaq YM, Osman N, Ibrahim A. Fetal exposure to aflatoxins in the United Arab Emirates. *Ann Trop Paediatr* 2002; **22**: 3–9.

303	De Vries HR, Maxwell SM, Hendrickse RG. Foetal and neonatal exposure to aflatoxins. *Acta Paediatr Scand* 1989; **78**: 373–8.

304	Tesfamariam K, Gebreyesus SH, Lachat C, *et al.* Chronic aflatoxin exposure during pregnancy is associated with lower fetal growth trajectories: a prospective cohort from the BUNMAP study in rural Ethiopia. *Am J Clin Nutr* 2022. DOI:10.1093/ajcn/nqac280.

305	Hendrickse RG, Coulter JB, Lamplugh SM, *et al.* Aflatoxins and kwashiorkor: a study in Sudanese children. *Br Med J (Clin Res Ed)* 1982; **285**: 843–6.

306	de Vries HR, Maxwell SM, Hendrickse RG. Aflatoxin excretion in children with kwashiorkor or marasmic kwashiorkor--a clinical investigation. *Mycopathologia* 1990; **110**: 1–9.

307	Hatem NL, Hassab HMA, Abd Al-Rahman EM, El-Deeb SA, El-Sayed Ahmed RL. Prevalence of aflatoxins in blood and urine of Egyptian infants with protein-energy malnutrion. *Food Nutr Bull* 2005; **26**: 49–56.

308	Coulter JB, Hendrickse RG, Lamplugh SM, *et al.* Aflatoxins and kwashiorkor: clinical studies in Sudanese children. *Trans R Soc Trop Med Hyg* 1986; **80**: 945–51.

309	Apeagyei F, Lamplugh SM, Hendrickse RG, Affram K, Lucas S. Aflatoxins in the livers of children with kwashiorkor in Ghana. *Trop Geogr Med* 1986; **38**: 273–6.

310	Adhikari M, Gita-Ramjee, Berjak P. Aflatoxin, kwashiorkor, and morbidity. *Nat Toxins* 1994; **2**: 1–3.

311	de Vries HR, Lamplugh SM, Hendrickse RG. Aflatoxins and kwashiorkor in Kenya: a hospital based study in a rural area of Kenya. *Ann Trop Paediatr* 1987; **7**: 249–51.

312	Househam K, Hundt H. Aflatoxin exposure and its relationship to kwashiorkor in African children. *J Trop Pediatr* 1991; **37**: 300–2.

313	Lamplugh SM, Hendrickse RG. Aflatoxins in the livers of children with kwashiorkor. *Ann Trop Paediatr* 1982; **2**: 101–4.

314	McMillan A, Renaud JB, Burgess KMN, *et al.* Aflatoxin exposure in Nigerian children with severe acute malnutrition. *Food Chem Toxicol* 2018; **111**: 356–62.

315	Hendrickse RG, Coulter JB, Lamplugh SM, *et al.* Aflatoxins and kwashiorkor. Epidemiology and clinical studies in Sudanese children and findings in autopsy liver samples from Nigeria and South Africa. *Bull Soc Pathol Exot Fil* 1983; **76**: 559–66.

316	Hendrickse RG. The influence of aflatoxins on child health in the tropics with particular reference to kwashiorkor. *Trans R Soc Trop Med Hyg* 1984; **78**: 427–35.

317	Okoth SA, Ohingo M. Dietary aflatoxin exposure and impaired growth in young children from Kisumu District, Kenya: Cross sectional study. *Afr J Heal Sci* 2004; **11**: 43–54.

318	Lei L, Liu S, Ye Y, *et al.* Associations between Serum Aflatoxin-B1 and Anemia in Pregnant Women: Evidence from Guangxi Zhuang Birth Cohort in China. *Toxins (Basel)* 2021; **13**. DOI:10.3390/toxins13110806.

319	Shuaib FM, Jolly PE, Ehiri JE, *et al.* Association between anemia and aflatoxin B1 biomarker levels among pregnant women in Kumasi, Ghana. *Am J Trop Med Hyg* 2010; **83**: 1077–83.

320	Pratiwi N, Sunarno I, Lotisna D, Arifuddin S, Mailoa J. The association between aflatoxin and iron deficiency anemia in pregnant women: a cross-sectional study. *Gazz Med Ital* 2024; **183**: 419–22.

321	Murashiki T, Mazhandu J, Zinyama-Gutsire R, Mutingwende I, Mazengera L, Duri K. Association between anaemia and aflatoxin ­ B 1 and fumonisin ­ B 1 exposure in HIV ‑ infected and HIV ‑ uninfected pregnant women from Harare , Zimbabwe. *Mycotoxin Res* 2025; : 147–61.

322	Chen J, Ye Y, Huang D, *et al.* The mediating role of coagulation function on the association of prenatal exposure to aflatoxin B1 and postpartum hemorrhage in Guangxi, China. *Env Sci Pollut Res Int* 2022; **29**: 37543–55.

323	Moodley J, Moodley D, Reddy L, Dutton MF, Chuturgoon AA. Fumonisin B1: An aetiological role in pre-eclampsia. *J Obstet Gynaecol (Lahore)* 2001; **21**: 599–600.

324	Ali S, Malik A, Shahid M, Bhargava R. Pulmonary aspergillosis and aflatoxins in chronic lung diseases. *Mycopathologia* 2013; **176**: 287–94.

325	Dvorackova I, Stora C, Ayraud N. Evidence for aflatoxin B1 in two cases of lung cancer in man. *J Cancer Res Clin Oncol* 1981; **100**: 221–4.

326	Cusumano V. Aflatoxins in serum from patients with lung-cancer. *Oncology* 1991; **48**: 194–5.

327	Rodriguez VI, Mammadova J, Permuth JB, *et al.* Elevated Urinary Levels of Fungal and Environmental Toxins in Patients with Pancreatic Ductal Adenocarcinoma. *J Gastrointest Cancer* 2025. DOI:10.1007/s12029-024-01125-4.

328	Truong NN, Hadush KT, Visintin L, *et al.* Associating Multiple Mycotoxin Exposure and Health Outcomes: Current Statistical Approaches and Challenges. *World Mycotoxin J* 2022; : 1–8.

329	World Cancer Research Fund. Diet, Nutrition, Physical Activity and Cancer: a Global Perspective. 2018 https://www.wcrf.org/diet-and-cancer/ (accessed Jan 14, 2022).

330	Kimani PM. Factors associated with prevalence of liver disease among seropositive HBV and AFB1 individuals in selected health facilities in Kitui and Makueni Counties, Kenya. 2022.

331	Mahfuz M, Gazi A, Hossain M, Islam MR, Fahim SM, Ahmed T. General and advanced methods for the detection and measurement of aflatoxins and aflatoxin metabolites: a review. *Toxin Rev* 2020; **39**: 123–37.

332	Morgan RL, Thayer KA, Bero L, *et al.* GRADE: Assessing the quality of evidence in environmental and occupational health. *Environ Int* 2016; **92**–**93**: 611–6.
